# A Systematic Review and Meta-Analysis of Preoperative Biliary Drainage Methods in Periampullary Tumors

**DOI:** 10.3390/jcm14197097

**Published:** 2025-10-08

**Authors:** Septimiu Alex Moldovan, Emil Ioan Moiș, Florin Graur, Ion Cosmin Puia, Iulia Vlad, Vlad Ionuț Nechita, Luminiţa Furcea, Florin Zaharie, Călin Popa, Daniel Corneliu Leucuța, Simona Mirel, Mihaela Ştefana Moldovan, Tudor Mocan, Andrada Seicean, Andra Ciocan, Nadim Al Hajjar

**Affiliations:** 1 Department of Surgery, “Octavian Fodor” Regional Institute of Gastroenterology and Hepatology, Croitorilor Str., No. 19–21, 400162 Cluj-Napoca, Romania; septimiu1995@yahoo.com (S.A.M.); graurf@yahoo.com (F.G.); drpuia@yahoo.fr (I.C.P.); iulia.vlad94@yahoo.com (I.V.); nechita.vlad@umfcluj.ro (V.I.N.); luminita.furcea@yahoo.com (L.F.); florinzaharie@yahoo.com (F.Z.); calinp2003@yahoo.com (C.P.); andra.ciocan10@gmail.com (A.C.);; 2 Department of Surgery, “Iuliu Hațieganu” University of Medicine and Pharmacy, Croitorilor Str., No. 19–21, 400162 Cluj-Napoca, Romania; 3 Department of Medical Informatics and Biostatistics, “Iuliu Hațieganu” University of Medicine and Pharmacy, Louis Pasteur Str., No. 6, 400349 Cluj-Napoca, Romania; dleucuta@umfcluj.ro; 4 Department of Medical Devices, “Iuliu Hațieganu” University of Medicine and Pharmacy, Louis Pasteur Str., No. 4, 400349 Cluj-Napoca, Romania; smirel@umfcluj.ro; 5 Department of Endocrinology, County Emergency Hospital, Endocrinology Clinic, Louis Pasteur Str., No. 3–5, 400349 Cluj-Napoca, Romania; mihaelamirel@gmail.com; 6 3rd Medical Department, “Iuliu Hațieganu” University of Medicine and Pharmacy, 400162 Cluj-Napoca, Romania; mocan_tudor@yahoo.com; 7 UBBMed Department, Babeș-Bolyai University, 400349 Cluj-Napoca, Romania; 8 Department of Gastroenterology, “Octavian Fodor” Regional Institute of Gastroenterology and Hepatology, Croitorilor Str., No. 19–21, 400162 Cluj-Napoca, Romania; andradaseicean@yahoo.com; 9 Department of Gastroenterology, “Iuliu Hațieganu” University of Medicine and Pharmacy, Croitorilor Str., No. 19–21, 400162 Cluj-Napoca, Romania

**Keywords:** periampullary neoplasms, preoperative biliary drainage, postprocedural complications, postoperative complications, endoscopic retrograde biliary drainage, endoscopic nasobiliary drainage, percutaneous transhepatic biliary drainage, plastic stent, self-expandable metallic stent

## Abstract

**Background**: Pancreatic and hepatobiliary tumors continue to rank among the deadliest cancers worldwide. Due to a low response rate to treatment, these tumors continue to have a high death rate, a poor prognosis and survival rate, and an overall poor patient outcome. The multimodal strategy used in current treatment includes systemic therapy, radiation therapy, and surgery. However, surgery remains the only treatment with curative intent. Preoperative biliary drainage has a direct impact on the perioperative prognosis of patients with obstructive jaundice and significantly compromised liver function due to hepato-bilio-pancreatic malignancies. Our study’s goal was to determine the safest and most efficient preoperative biliary drainage technique by conducting a systematic review and meta-analysis of resectable periampullary cancers. **Methods**: Our approach consisted of searching PubMed, BMC Medicine, and Scopus databases using keywords with a result of 1104 articles from 2010 to 2023. The remaining 24 articles that met our inclusion criteria were subjected to meta-analysis using R Commander 4.3.2. **Results**: Endoscopic retrograde biliary drainage (ERBD) demonstrated a higher rate of postprocedural pancreatitis (RR = 2.22, *p* < 0.01), intra-abdominal abscess (RR = 1.64, *p* < 0.01), and delayed gastric emptying (DGE) (RR = 2.07, *p* < 0.01) than percutaneous transhepatic biliary drainage (PTBD) or endoscopic nasobiliary drainage (ENBD). Plastic stent (PS) had higher rates of catheter occlusion (RR = 2.20, *p* < 0.01) and POPF (RR = 1.66, *p* < 0.01) compared to self-expandable metallic stent (SEMS), which could explain a longer hospital stay (MD = 2.41 days, *p* < 0.01). However, PS had lower rates of grade 1–2 complications (RR = 0.79, *p* = 0.017) and wound infection rates (RR = 0.66, *p* = 0.017) than self-expandable metallic stent (SEMS). **Conclusions**: The choice of a preoperative drainage method can influence postprocedural and postoperative complications rates. ERBD appears to be associated with higher procedure-related and postoperative complication rates and may be linked to a prolonged hospital stay compared to ENBD or PTBD. Moreover, the type of stent placed through ERBD procedure had an important impact on prognosis, as PS had a higher rate of catheter occlusion and POPF, with a prolonged hospital stay compared to SEMS, while mild complications and wound infections were less common in PS group.

## 1. Introduction

Periampullary cancers include 4 main categories of neoplasms: ductal adenocarcinoma of the pancreatic head, distal cholangiocarcinoma, ampullary carcinoma, and duodenal adenocarcinoma. The only curative treatment is surgery, represented by pancreaticoduodenectomy (Whipple procedure and its variants). The prognosis of this category of cancers is unfavorable, with ductal pancreatic adenocarcinoma being the third oncological cause of death after pulmonary and rectal cancers. The 5 years survival rate of patients with pancreatic cancer is 8%, only 15–20% of patients being diagnosed in a resectable stage [1,2]. Patients with periampullary tumors often experience obstructive jaundice, an important risk factor for increased perioperative morbidity [3]. In such cases, preoperative biliary drainage (PBD) can improve liver function, thereby supporting coagulation, immunity, and nutrition, and ultimately reducing perioperative complications and mortality [4]. Preoperative biliary drainage (PBD) has long been used to manage obstructive jaundice in patients with periampullary tumors; however, its role remains controversial due to the infectious perioperative complications associated with it [3]. Current clinical guidelines, including those from the National Comprehensive Cancer Network (NCCN) and the European Society for Medical Oncology (ESMO), recommend against routine PBD in patients with resectable disease, advising its use only in selected situations such as acute cholangitis, anticipated delay in surgery (including neoadjuvant treatment), or markedly elevated bilirubin levels (≥15 mg/dL) [5,6]. This cautious approach stems from evidence showing that PBD may be associated with higher postoperative morbidity and mortality compared with immediate surgery, and that prior meta-analyses have failed to demonstrate a clear survival benefit in unselected patients [3,7,8,9]. In this context, our meta-analysis focuses specifically on comparing outcomes among the various PBD techniques in patients for whom drainage is clinically indicated, aiming to provide evidence to guide optimal method selection. The most commonly used PBD methods include endoscopic retrograde biliary drainage (ERBD), endoscopic nasobiliary drainage (ENBD), and percutaneous transhepatic biliary drainage (PTBD) [4]. Different studies have reported in different percentages the types of complications that can be induced by PBD, such as acute pancreatitis, cholangitis, papillary hemorrhage, biliary/duodenal perforation, and catheter occlusion with relapse of obstructive jaundice [10,11,12,13,14]. Another important aspect of PBD is that it contaminates the bile and increases the risk of perioperative infectious complications. Probably the best approach in medical practice would be to send patients directly to surgery whenever possible without performing any type of biliary drainage. Some evidence supporting this approach was recently published in a network meta-analysis by Lee et al. in 2018 [15]. However, in some clinical scenarios, when a longer time from diagnosis to surgery is estimated (e.g., preoperative chemotherapy), a PBD is necessary. ERBD is the first-line modality for preoperative biliary decompression in distal malignant obstruction when drainage is indicated—typically in the presence of acute cholangitis, severe/symptomatic hyperbilirubinemia, or when surgery is expected to be delayed (e.g., during neoadjuvant therapy). In resectable disease, routine preoperative drainage is generally discouraged by contemporary guidelines; when ERCP is not feasible or fails, alternative approaches (e.g., PTBD, ENBD, EUS-guided biliary drainage) are recommended [6,16,17,18]. PTBD is generally reserved as a second-line option in the preoperative management of periampullary tumors. Its main indications include failed or technically unfeasible ERCP (e.g., due to duodenal obstruction, large or infiltrative tumors, or surgically altered anatomy), contraindications to endoscopic drainage, or urgent decompression in severe cholangitis when ERCP is not immediately available. PTBD may also be indicated for selective drainage in complex hilar obstruction or when external drainage is clinically preferred. While effective, PTBD carries specific drawbacks such as catheter dislodgement, bleeding, external bile loss, and the potential risk of tumor seeding along the catheter tract, and therefore its use is typically limited to selected cases [5,19]. ENBD is less commonly employed in the preoperative management of periampullary tumors, but it may be indicated in selected situations such as severe or complicated cholangitis, when repeated cholangiography or bile sampling is required, or when short-term drainage is needed before surgery. ENBD can also be preferred in cases with a high risk of stent occlusion, contraindications to internal stenting, or as a temporary bridge to definitive drainage. However, due to patient discomfort and the risk of accidental tube dislodgement, ENBD is generally reserved for acute or short-term use rather than for prolonged preoperative drainage [20,21]. Navigating between different PBD techniques is not straightforward. Even if there are studies comparing different PBD methods, there is a need for a systematic review and a meta-analysis to determine which PBD method is the most effective and has the lowest risk of complications. Therefore, we systematically reviewed relevant studies on PBD methods, including ERBD (plastic stents (PS), fully covered self-expanding metallic stents (FCSEMS), uncovered self-expanding metallic stents (UCSEMS), ENBD, and PTBD, with the aim of answering three key questions:

1. Which PBD method has the lowest postprocedural complications?
2. Which type of PBD has a decreased postoperative morbidity?
3. Is mortality influenced by the PBD technique used?

Moreover, we performed a meta-analysis of the relevant results to demonstrate the statistical significance of the differences found between the therapeutic methods compared.

## 2. Materials and Methods

### 2.1. Literature Search Strategy

This paper’s review protocol was registered on PROSPERO—International prospective register of systematic reviews, in 23 May 2024 with the ID: CRD42024538598.

**Figure 1:**
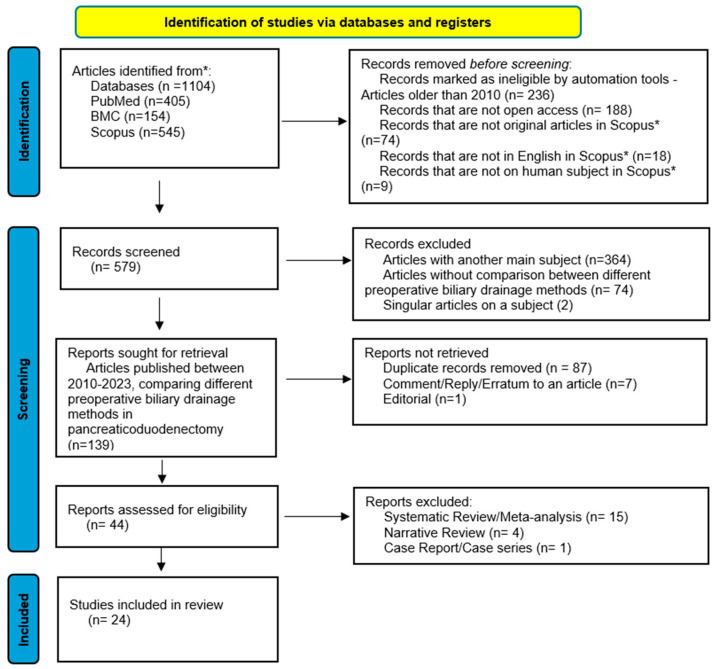
PRISMA 2020 flow diagram for new systematic reviews [22]. * Additional screening tool in Scopus database: selection of original articles.

To find answers to the three key questions raised, we performed an organized search of the literature in accordance with the Preferred Reporting Items for Systematic Reviews and Meta-Analyses (PRISMA) statement (Figure 1) [22].

Our study was conducted by two independent researchers, facilitating the proper verification of the results. Discrepancies were collectively analyzed and resolved, and the same results were obtained.

We conducted an electronic search for original articles indexed in PubMed, BMC, and Scopus databases. Eligibility criteria were restricted to studies published in English and conducted on human subjects. The literature search was conducted in two major English-language databases (MEDLINE via PubMed and Scopus) and one additional database (BMC), which together comprehensively index the vast majority of high-quality peer-reviewed studies in this field. A preliminary scoping search did not identify further eligible studies in other databases or languages. This approach was designed in accordance with the PRISMA 2020 guidelines (Supplementary Material S4). To ensure relevance to contemporary clinical practice, we limited the inclusion of studies to those published between January 2010 and December 2023. This timeframe was selected based on several considerations: (1) significant advancements in endoscopic and percutaneous drainage techniques, devices, and perioperative care have occurred since 2010; (2) a pivotal randomized controlled trial by van der Gaag et al. (2010) prompted a shift in clinical guidelines and practice patterns regarding preoperative biliary drainage; and (3) older studies risk introducing methodological and clinical heterogeneity due to outdated protocols and equipment [7].

A systematic search was performed in the PubMed database using specific word constructions separated by Boolean operators (AND, OR): “preoperative biliary drainage” OR “percutaneous transhepatic biliary drainage” OR “T-tube drainage” OR “cholecystostomy” OR “biliodigestive derivation” AND “pancreaticoduodenectomy” OR “Whipple procedure” OR “total pancreatectomy.” The BMC and Scopus databases were scanned using the following keywords: “preoperative biliary drainage” AND “pancreaticoduodenectomy” covering every method of biliary decompression in the preoperative setting.

The results were initially filtered during the title and abstract screening stage, where non-eligibility criteria were applied. These included article type (only original articles), language (English), and publication year (2010–2023). To support the exclusion of non-original articles (e.g., reviews, editorials, conference abstracts), we used Scopus article type classification as an additional screening tool. This approach complemented, but did not override, our predefined eligibility criteria, and was not used as the sole basis for exclusion. Moreover, articles without open access or unretrievable full texts despite reasonable institutional and interlibrary efforts were excluded. No study was excluded solely based on its open access status.

After excluding articles that did not meet the inclusion criteria, duplicates were removed from the remaining group by two separate researchers to avoid bias. All the sorted articles were included in a Microsoft Excel database, with their title and DOI or PMID facilitating a fast recognition of duplicates by a search in the database, and moreover, allowing Mendeley application to include studies as citations and automatically generating the bibliography (Supplementary Material S1). Tables and statistical functions were generated in Microsoft Excel with the aim of organizing the raw data to facilitate drawing the results and conclusions of the study.

### 2.2. Inclusion Criteria

Inclusion criteria consisted of: (1) subjects from the studies with confirmed periampullary tumors; (2) original articles that highlight a comparison between different types of preoperative biliary drainage with the following techniques taken into consideration: Endoscopic Retrograde Biliary Drainage—ERBD (plastic, uncovered, partially or fully covered metal biliary stents), T-tube/Kehr’s tube biliary drainage (laparoscopic/open surgery), cholecystostomy (percutaneous/endoscopic/surgically), percutaneous transhepatic biliary drainage, nasobiliary drainage (ENBD), biliodigestive derivation (laparoscopic/open surgery); (3) type of study: retrospective/prospective cohort studies and randomized control trials (RCTs); (4) patient sample size: unlimited; (5) language of published articles: English; (6) subjects type: human patients; (7) publication years: 2010–2023 (8) research variables: patients’ characteristics (soft pancreas, main biliary duct (MBD) diameter (mm)), postprocedural complications (duration of drainage (days), pancreatitis, cholangitis, perforation, hemorrhage, stent occlusion, catheter exchange), postoperative complications (overall complication rate, sepsis, intraabdominal abscess, wound infection, post pancreatectomy hemorrhage (PPH), Chyle leak, postoperative biliary fistula (POBF), postoperative pancreatic fistula (POPF), delayed gastric emptying (DGE), intraoperative parameters and postoperative characteristics (operative time (min), blood loss (ml), hospital stay (days), mortality, reoperation).

### 2.3. Data Exclusion

Studies that fall into the following categories were removed from our literature assessment: (1) irrelevant and incomplete scientific papers; (2) duplicate articles; (3) publications older than 2010; (4) literature written in a language other than English; (5) studies performed on non-human subjects (cell cultures or animals); (6) type of publication: narrative review, systematic review, meta-analysis, case report, comments/replies/erratum to an article and editorial articles; (7) studies about different PBD methods, but failing to make a comparison among them, (8) singular studies on certain PBD methods comparison

Consequently, we retrieved 24 articles that met the inclusion criteria. Two separate investigators performed a thorough check of the results, eliminating discrepancies and drawing a common line regarding the results.

### 2.4. Outcomes and Definitions

In this study, we assessed outcomes across four main categories: postprocedural complications (e.g., pancreatitis, cholangitis, perforation, and hemorrhage), intraoperative characteristics (e.g., soft pancreas, MBD diameter, operative time, and blood loss), postoperative complications (e.g., overall complication rate, POPF, PPH, and DGE), and prognostic factors (e.g., hospital stay, reoperation rate, and mortality). This classification allowed for a comprehensive evaluation of the clinical impact of different biliary drainage approaches at multiple stages of the perioperative course.

Soft pancreas, mean biliary diameter, and detailed complications were not mentioned in the initial protocol and were added during data extraction due to their frequent reporting and clinical relevance.

Post-ERCP pancreatitis was defined as new or worsened abdominal pain, accompanied by an elevation of serum amylase and/or lipase to at least three times the upper limit of normal, occurring within 24 h after the procedure, and requiring hospitalization or extension of planned admission, consistent with the revised Atlanta classification and the Cotton criteria [23,24]. Tokyo Guidelines 2018 adapted for post-procedural context defines cholangitis as the development of fever (≥38 °C) and clinical signs of systemic inflammation (e.g., chills, leukocytosis) within 24 to 72 h following ERCP, accompanied by biochemical evidence of cholestasis (elevated bilirubin and/or alkaline phosphatase) and no other identifiable source of infection [25]. Perforation was defined, according to ASGE guidelines, as an abnormal connection between the gastrointestinal tract and surrounding spaces, confirmed by clinical symptoms, imaging findings, endoscopic observation, or the need for surgical or radiological intervention [26]. Post-ERCP hemorrhage was defined, in alignment with ASGE guidelines, as bleeding during or after ERCP—typically after sphincterotomy—evidenced by gastrointestinal bleeding symptoms, a hemoglobin drop of ≥2 g/dL, or the need for transfusion or hemostatic intervention [26,27]. Catheter occlusion was defined as recurrent jaundice, cholangitis, or imaging evidence of biliary obstruction after initial improvement, requiring catheter replacement or revision, and was considered a procedure-related complication in studies comparing biliary drainage methods.

Soft pancreas was defined as the surgeon’s intraoperative assessment of a non-fibrotic, pliable gland with low firmness and a small duct (<3 mm) and is a known risk factor for postoperative pancreatic fistula due to its fragile texture [28]. The main biliary duct (MBD) diameter refers to the internal width of the common bile duct, usually measured on imaging or during surgery, with values over 6 mm generally considered dilated.

Overall complication rate was defined as the proportion of patients experiencing at least one postoperative adverse event, regardless of type or severity, during the defined follow-up period. To ensure consistency across studies, complications were also classified—where available—according to the Clavien–Dindo classification system, which categorizes surgical complications based on severity, from minor deviations in postoperative course (Grade I) to life-threatening events or death (Grades IV–V) [29] Infectious complications were defined as postoperative infections confirmed by clinical symptoms, laboratory evidence, or imaging, and requiring antimicrobial therapy or other intervention. These included: Intra-abdominal abscess (localized fluid collection with signs of infection confirmed by imaging or intraoperatively), Wound infections (erythema, purulent drainage, or positive wound cultures requiring treatment), and Sepsis (systemic inflammatory response to infection, defined by fever or hypothermia, leukocytosis or leukopenia, and signs of organ dysfunction) [30]. Post-pancreatectomy hemorrhage (PPH) was defined according to the general criteria established by the International Study Group of Pancreatic Surgery (ISGPS) as any bleeding occurring after pancreatic resection, regardless of timing, location, or severity [31]. Chyle leak is defined as the presence of lymphatic fluid in postoperative abdominal drainage, characterized by milky or opalescent appearance of the fluid, and triglyceride concentration ≥110 mg/dL (≥1.2 mmol/L) in the drainage fluid [32]. Postoperative biliary fistula was defined as bilious drain output with a bilirubin concentration at least three times higher than serum levels, as per ISGLS criteria [33]. Postoperative pancreatic fistula was defined according to ISGPS as drain fluid with an amylase level more than three times the upper limit of normal serum amylase, measured on or after postoperative day 3, and associated with a clinical impact (such as infection, need for intervention, or prolonged drainage). POPFs were graded based on clinical impact: Grade 1 (biochemical leak, no clinical relevance), Grade 2 (requiring change in management, such as prolonged drainage or antibiotics), and Grade 3 (severe, requiring reoperation, invasive intervention, or associated with organ failure or death) [28]. As grading was not consistently reported across studies, we grouped postoperative pancreatic fistulas into two categories for analysis: Grade 1 as biochemical leaks and Grades 2–3 as clinically relevant fistulas. Delayed gastric emptying (DGE) was defined according to ISGPS criteria as the inability to tolerate oral intake due to impaired gastric motility, requiring the continued use of a nasogastric tube or the inability to resume a solid diet within a specified postoperative timeframe [34].

To address heterogeneity and enhance clinical interpretability, we conducted subgroup analyses based on predefined comparisons (ERBD vs. ENBD/PTBD and PS vs. SEMS). For each subgroup, outcomes were analyzed individually due to varying availability of data across studies. Variables reported in only a few studies were still presented for completeness, though their results should be interpreted with caution due to limited statistical power.

Risk of bias was assessed using the RoB 2.0 tool for randomized controlled trials, evaluating five domains: bias arising from the randomization process, deviations from intended interventions, missing outcome data, measurement of the outcome, and selection of the reported result. For non-randomized studies, the ROBINS-I tool was applied, covering seven domains including confounding, selection of participants, classification of interventions, deviations from intended interventions, missing data, measurement of outcomes, and selection of the reported results (Supplementary Material S2).

### 2.5. Statistical Analysis

Finally, we performed a quantitative analysis of the 24 studies by comparing different parameters between multiple PBD methods to respond to the following three key questions:

1. Which PBD method has the lowest postprocedural complications?
2. Which type of PBD has a decreased postoperative morbidity?
3. Is mortality influenced by the PBD technique used?

Meta-analysis was performed using R Commander 4.3.2 software. Risk ratio (RR) was the tool of choice for dichotomous qualitative variables. Continuous variables were found among different articles expressed as mean and standard deviation, median, quartile 1 and quartile 3, or median, minimum, and maximum. Therefore, we applied the methods proposed by Wan et al. (2014) and Luo et al. (2018) for estimating the sample mean (M) and standard deviation (SD) from reported medians and dispersion measures, implemented using Microsoft Excel [35,36] (Supplementary Material S3). One study presented a variable in terms of standard error (SE), and we excluded this singular variable from our analysis. The funnel plot was the chart chosen to depict the mean and SD for the variables compared in different studies. The chi-squared test was used to establish the homogeneity of each study. The fixed effects model was used only if homogeneity was accepted (I^2^ < 35%, *p* < 0.05). If there was significant clinical heterogeneity among the studies, a random effects model was used. Statistical significance was set at *p* < 0.05. Heterogeneity could not be assessed when there was only one study included in the meta-analysis performed on a variable. Leave-one-out sensitivity analysis consisted of consecutively removing one study to assess whether the results were influenced by a single study. A leave-one-out sensitivity plot was used to graphically depict the effect size after excluding one study at a time from our statistical analysis. The publication bias test (Egger’s test) was applied for every comparison between at least three studies.

Results were expressed as mean difference (MD) with a 95% confidence (CI) for continuous variables with a level of statistical significance established at a *p* < 0.05, by applying the test of overall effect (Z-test) (Supplementary Material S6). On the other hand, dichotomous variables were analyzed by counting Mean Risk ratio (RR) with 95% confidence (CI) (Supplementary Material S5). The results were schematically expressed in a forest plot. The rhombus (diamond) shape at the end of each forest plot represents the pooled effect estimate and its 95% confidence interval. Its center corresponds to the overall effect size, and its width reflects the precision of the estimate. If the diamond does not intersect the line of no effect, the pooled result is statistically significant.

Subgroup analysis of the mean difference (MD) was performed in the same manner as when leave-one-out sensitivity analysis indicated a study influencing the results.

## 3. Results

### 3.1. Search Tools and Study Characteristics

All the relevant studies (n = 24) included in this analysis compared different biliary drainage methods used before pancreaticoduodenectomy, with the aim of establishing the most effective technique with the lowest complication rate. The publications included in the current meta-analysis consisted of 2 RCTs, 4 prospective cohort studies, and 18 retrospective cohort studies. Our meta-analysis was performed on 5885 patients. From a procedural approach point of view, we analyzed a number of 2614 patients with Endoscopic Retrograde Biliary Drainage (ERBD), 1761 with Endoscopic Nasobiliary Drainage (ENBD)/Percutaneous Transhepatic Biliary Drainage (PTBD). Subsequently, different types of stents placed during ERBD procedure were compared, with a number of 949 patients with Plastic Stents (PS), 561 with self-expandable metallic stents (SEMS). When ERBD was considered as a group, it included patients who had a PS, UCSEMS, partially covered self-expandable metallic stents (PCSEMS), or FCSEMS placed in the main biliary duct (MBD), without comparing the types of biliary stents between them.

Basic characteristics regarding the studies included in our analysis are illustrated in Table 1. Moreover, we have pointed out in Table 1 the differences between the groups by patients’ age, neoadjuvant therapy, and pancreatic features.

Most studies had similar groups according to age, sex distribution, type of operation, histological type of the tumor, and percentage of neoadjuvant treatment, with some exceptions. The age of the patients was higher in the group with internal drainage (ERBD) than in the external drainage group (ENBD/PTBD) in a study by Okano et al. in 2019 [44]. The percentage of male patients was higher in the ERBD group than in the ENBD group in the study by Satoh et al. from 2022 [38]. The ratio of pylorus-preserving pancreaticoduodenectomy (PPPD) to pancreaticoduodenectomy (PD) was higher in the ERBD group than in the PTBD group in two studies (Lee et al. from 2018 and Byun et al. from 2021) [15,48]. In a retrospective cohort study conducted in 2014, Kitahata et al. compared internal drainage (ERBD) and external drainage (PTBD/ENBD) and showed a statistically significant difference between the PD/PPPD/Pylorus resecting pancreaticoduodenectomy (PRPD) ratio, with a much higher rate of PRPD in the ERBD group [49]. The percentage of histological type of ductal adenocarcinoma of the pancreas was higher in the SEMS group than in the PS (Latenstein et al., 2021), and in the PTBD group than in the ERBD group (Lee et al., 2018) [15,40]. Neoadjuvant treatment was more prevalent in the SEMS group than in the PS group in 3 studies (Latenstein et al., 2021, Roberts et al. 2021, Cavell et al. 2013) [40,45,52]. In the studies that considered the intraoperative characteristics of the main biliary duct diameter and the percentage of patients with soft texture of the pancreas, there was homogeneity between the studied groups, except for Latenstein et al., 2021, where the group drained by PS had a much higher incidence of soft pancreas than the group that underwent preoperative biliary drainage by SEMS [40]. Adding the above exceptions marks a clinical heterogeneity among groups that requires utilizing the model for random effects.

A meta-analysis was performed on subgroups of patients sorted according to the type of preoperative biliary drainage procedures.

### 3.2. Statistical Analysis Among Subgroups

#### 3.2.1. Procedural Approach: ERBD Versus ENBD/PTBD

##### Postprocedural Complications and Delay Until Surgery

From the 15 studies which compared ERBD with ENBD and/or PTBD we extracted the following conclusions.

Pancreatitis

**Figure 2:**
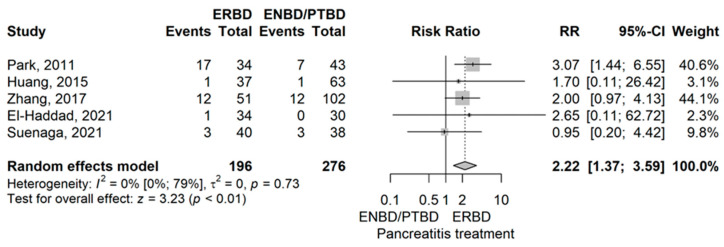
Forest plot for Pancreatitis rate, comparing ERBD with ENBD/PTBD [10,14,42,50,53].

Pancreatitis rate had demonstrated a higher rate in the ERBD group compared to ENBD/PTBD with an RR value of 2.22 (95% CI 1.37–3.59), *p* = 0.001 using the model with random effects (Figure 2).

There was no compelling evidence of publication bias (*p* = 0.533). Park et al. (2011) had shown a divergent result compared to the other articles by the leave-one-out sensitivity analysis. No heterogeneity was demonstrated (I^2^ = 0%, *p* = 0.735) [14].

Cholangitis

With an RR value of 1.51 (95% CI 0.82–2.80), *p* = 0.19 when comparing ERBD to ENBD/PTBD using the model with random effects, cholangitis was not observed to vary statistically among the compared studies. Studies were free of publication bias (*p* = 0.276). No impacting studies were found by using the leave-one-out sensitivity analysis. Significant heterogeneity was present (I^2^ = 69.4%, *p* = 0.003).

Perforation

None of the 15 articles studied have analyzed the perforation rate after ERBD compared to ENBD/PTBD.

Hemorrhage

Hemorrhage did not demonstrate a significant difference between ERBD and ENBD/PTBD (RR = 1.06 (95% CI 0.11–9.92), *p* = 0.961) by the model with random effects.

The publication bias test was not applicable. Leave-one-out sensitivity analysis had found both studies compared (Park et al., 2011 and El-Haddad et al., 2021) as influencing to the RR value [14,50]. No heterogeneity was demonstrated (I^2^ = 0%, *p* = 0.42).

Catheter occlusion

No statistically significant variation in catheter occlusion, depending on the procedure, was observed (RR = 2.26 (95% CI 0.34; 15.24), *p* = 0.401).

Egger’s test showed no publication bias (*p* = 0.124). According to leave-one-out sensitivity analysis, the results were affected by leaving out Zhang et al., 2017 or Park et al., 2011 [10,14]. Moderate to substantial heterogeneity was revealed (I^2^ = 64.9%, *p* = 0.058).

Catheter exchange

No statistically significant difference between ERBD and ENBD/PTBD was estimated (RR = 0.82 (95% CI: 0.62 to 1.08), *p* = 0.157).

No publication bias was identified (*p* = 0.296). Omitting Suenaga et al. (2021) and Satoh et al. (2022) did impact the findings [38,53]. Moderate to substantial heterogeneity was observed (I^2^ = 69%, *p* = 0.04).

Duration of biliary drainage

The meta-analysis estimated the mean difference (MD) in the duration of drainage (in days) between the ERBD and ENBD/PTBD groups as 12.31 days (95% CI: −2.76 to 27.39), but without achieving a statistically significant threshold (*p* = 0.109).

No evidence of significant publication bias resulted (*p* = 0.18). Omitting the study by Satoh et al., 2022 significantly influenced the findings [38]. Substantial heterogeneity was demonstrated (I^2^ = 90.3%, *p* < 0.001).

##### Postoperative Complications

Overall complication rate

No statistical difference was demonstrated in terms of overall complication rate, when comparing the ERBD group to the ENBD/PTBD group (RR = 0.17, *p* = 0.21).

No publication bias was revealed (0.399). Only Okano et al.’s 2019 study was identified as an influencing study [44]. Significant heterogeneity was found (I^2^ = 97.5%), which was confirmed by the Q test (*p* < 0.001).

No statistical difference was shown for Grade 1–2 complication rate (RR = 0.99, *p* = 0.972). Grade ≥3 complication rates demonstrated no significant difference (RR = 0.89, *p* = 0.69).

Infectious complications

Infectious complications rate difference between ERBD and ENBD/PTBD did not reach statistical significance (RR = 1.13, *p* = 0.733).

Potential publication bias was shown by the Egger’s test (*p* = 0.015). Leave-one-out sensitivity analysis indicated 2 influencing studies: Fujii et al., 2015 and Okano et al., 2019 [12,44]. High level of statistical heterogeneity was confirmed (I^2^ = 91.5%, *p* < 0.001).

Sepsis

No statistically significant difference was found between ERBD and ENBD/PTBD in terms of the sepsis rate (RR = 2.42, *p* = 0.081).

No publication bias was detected (*p* = 0.227). No influencing studies were indentified. No statistical heterogeneity was observed (I^2^ = 0%, *p* = 0.929).

Intraabdominal abscess

**Figure 3:**
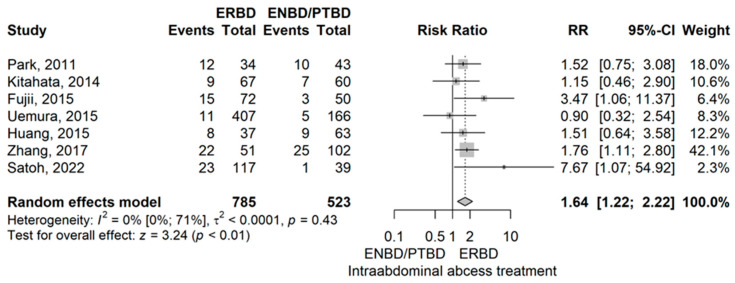
Forest plot for Intraabdominal abscess, comparing ERBD with ENBD/PTBD [10,12,14,38,42,49,54].

ERBD group compared to the ENBD/PTBD group, demonstrated a higher rate of intraabdominal abscess with an RR value of 1.64 (95% CI 1.22–2.22), *p* = 0.001 using the model with random effects (Figure 3).

Egger’s test did not detect significant publication bias (*p* = 0.506). No studies were found to have a significant influence in the leave-one-out sensitivity analysis. No heterogeneity was demonstrated by an I^2^ of 0% (95% CI 0–70.8%), nor was it confirmed by the Q test (*p* = 0.433).

Wound infections

No significant statistical difference was found between the ERBD group and the ENBD/PTBD group (RR = 1.28, *p* = 0.142).

No publication bias was detected (*p* = 0.631). Uemura et al.’ s, 2015 article was indicated as an influencing study by the leave-one-out sensitivity analysis [54]. Low to moderate heterogeneity was detected (I^2^ = 26.8%), but not confirmed (*p* = 0.215).

Postpancreatectomy hemorrhage (PPH)

No statistically significant difference between ERBD and ENBD/PTBD was indicated by the meta-analysis (RR = 1.21, *p* = 0.549).

Publication bias was detected in the dataset (*p* = 0.002). Omitting Okano et al.’s 2019 study had an influencing effect [44]. Substantial heterogeneity among the studies was revealed (I^2^ =70.3%, *p* < 0.001).

Chyle leak

Among the studies comparing ERBD with ENBD/PTBD, included in the meta-analysis, chyle leak was not a variable discussed in any of them.

Postoperative Biliary Fistula (POBF)

Meta-analysis could not demonstrate a statistically significant difference among groups compared (RR = 1.02, *p* = 0.959).

No publication bias was indicated (*p* = 0.872). No influencing studies were found. No significant heterogeneity was suggested (I^2^ = 0%, *p* = 0.727).

Postoperative Pancreatic Fistula (POPF)

Meta-analysis indicated a slightly higher risk of POPF for the ERBD group compared to the ENBD/PTBD group (RR = 1.29), but the difference was not statistically significant (*p* = 0.168).

Highly probable publication bias was evidenced (*p* < 0.001). Leave-one-out sensitivity analysis’s omission of Okano et al.’s 2019 study affected the outcomes [44]. Considerable heterogeneity among the included studies was evidenced (I^2^ = 93.1%, *p* < 0.001).

Grade 1 POPF had not demonstrated a statistically significant difference between ERBD and ENBD/PTPD (RR = 0.97, *p* = 0.907). Similarly, Grade 2–3 POPF did not have a different occurrence among the studies compared (RR = 1.20, *p* = 0.519).

Delayed gastric emptying (DGE)

**Figure 4:**
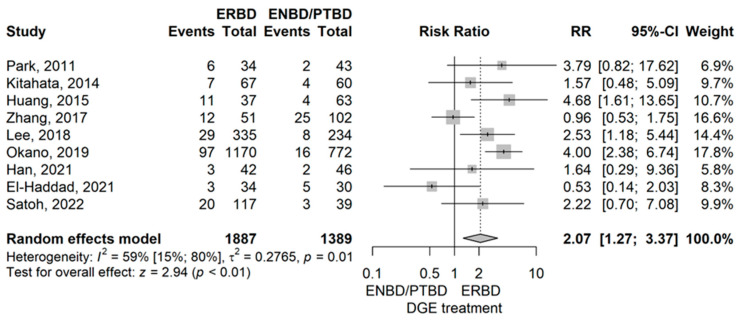
Forest plot for DGE, comparing ERBD with ENBD/PTBD [10,14,15,38,41,42,44,49,50].

The meta-analysis revealed that RR of DGE in the ERBD group was statistically higher compared to the ENBD/PTBD group (RR = 2.07 (95% CI: 1.27–3.37), *p* = 0.003) when using a random effects model (Figure 4).

No publication bias was demonstrated (*p* = 0.677). Omitting Zhang et al.’s 2017 study showed a significant influence on the results, according to leave-one-out sensitivity analysis [10]. There is evidence of moderate-to-substantial heterogeneity (I^2^ = 59.3% (95% CI 15.1–80.5%), *p* = 0.012). However, the large confidence interval suggests uncertainty about this result.

##### Intraoperative Characteristics

Soft pancreas

**Figure 5:**
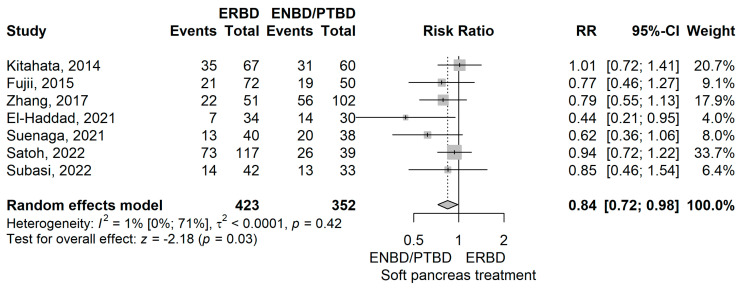
Forest plot for soft pancreas, comparing ERBD with ENBD/PTBD [10,12,38,39,49,50,53].

The relative risk (RR) for soft pancreas was 0.84 (95% CI: 0.72 to 0.98), *p* = 0.029, using a random-effects model. This indicates a statistically significant lower occurrence in the ERBD group compared to the ENBD/PTBD group (Figure 5).

The publication bias test yielded a significant *p* = 0.029. A single study was identified as influencing the outcome through a leave-one-out sensitivity analysis (Satoh et al., 2022) [38]. Low heterogeneity was demonstrated (I^2^ = 1.1% (95% CI 0–71.1%), *p* = 0.416). However, the wide CI for I^2^ indicates uncertainty in this assessment.

Main biliary duct (MBD) diameter

No statistically significant difference in MBD diameter was demonstrated (MD = 0.05 days, 95% CI: −0.82 to 0.92, *p* = 0.915).

No publication bias was indicated (*p* = 0.458). By omitting the studies by Fujii et al. (2015) and Satoh et al. (2022) findings were influenced [12,38]. Moderate heterogeneity was suggested (I^2^ = 57.3%), but it was not statistically significant (*p* = 0.096).

Operative time

Operative time showed an MD between the ERBD and ENBD/PTBD of 13.14 min (95% CI: −26.83 to 0.54), without reaching statistical significance (*p* = 0.06).

No evidence of significant publication bias had resulted (*p* = 0.531). No impacting studies were demonstrated. Moderate heterogeneity was present among studies compared (I^2^ = 49.7%, *p* = 0.026).

Blood loss

Larger blood loss was found in the ERBD group compared to ENBD/PTBD group (MD = 531.04 mL), but the result is not statistically significant (*p* = 0.23).

No strong evidence of publication bias was found (*p* = 0.115). Omitting the study by Fujii et al. (2015) had a notable impact on the results. High heterogeneity was observed (I^2^ = 98.2%, *p* < 0.001).

##### Prognostic Factors

Hospital stay

Hospital stay duration was longer in the ERBD group versus the ENBD/PTBD group (MD = 7.53 days), but the result is not statistically significant (*p* = 0.067).

No publication bias was found (*p* = 0.281). Omitting the study by Subasi et al., 2022 had a significant impact on the results [39]. High heterogeneity was revealed (I^2^ = 92.4%, *p* < 0.001).

Reoperation rate

No statistical difference in reoperation rate was demonstrated between ERBD and ENBD/PTBD (RR = 0.69, *p* = 0.514).

No evidence of publication bias was revealed (*p* = 0.871). Omitting Han et al. (2021) and Subasi et al. (2022) significantly influenced the findings [39,41]. Moderate heterogeneity across studies was suggested (I^2^ = 34.2%), but with no statistical relevance (*p* = 0.219).

Mortality

No significant difference in mortality risk between ERBD group and ENBD/PTBD group was demonstrated (RR = 1.00, *p* = 0.99).

Potential publication bias was suggested (*p* = 0.031). The algorithm did not converge during the leave-one-out sensitivity analysis. Moderate heterogeneity across studies was observed (I^2^ = 43.2%), though it lacked statistical significance (*p* = 0.07).

#### 3.2.2. Types of ERBD Stents: PS Versus SEMS

##### Postprocedural Complications and Delay Until Surgery

From the 9 studies which compared PS with SEMS we extracted the following conclusions.

Pancreatitis

The rate of pancreatitis failed to show a statistically significant difference between the ERBD group and the ENBD/PTBD group (RR = 0.72, *p* = 0.165).

No compelling evidence of publication bias was shown (*p* = 0.524). The leave-one-out sensitivity analysis revealed that Tol et al. (2016) and Latenstein et al. (2021) had shown a divergent result compared to the other articles [13,40]. Limited evidence of significant heterogeneity was found (I^2^ = 24.9%, *p* = 0.256).

Cholangitis

Cholangitis had statistical similar rates between ERBD and ENBD/PTBD (RR = 2.72, *p* = 0.054).

No publication bias was found (*p* = 0.057). Tol et al. (2016) and Latenstein et al. (2021) had an influencing effect on the outcome [13,40]. Moderate heterogeneity was observed across studies (I^2^ = 36.2%), but it did not reach statistical significance (*p* = 0.18).

Perforation

Perforation showed a higher RR-value of 2.49 in the ERBD group compared to the ENBD/PTBD group, but there was no statistically significant difference (*p* = 0.104).

The publication bias test was not applicable. Omitting Latenstein et al. (2021) significantly influenced the findings [40]. No heterogeneity was found (I^2^ = 0%, *p* = 0.374).

Hemorrhage

The meta-analysis did not reveal a statistically significant difference between PS and SEMS in terms of hemorrhage (RR = 1.57, *p* = 0.246).

Significant publication bias was indicated (*p* = 0.022). A significant impact was demonstrated by omitting the study of Latenstein et al. (2021) [40]. No evidence of heterogeneity was shown (I^2^ = 0%, *p* = 0.943).

Catheter occlusion

**Figure 6:**
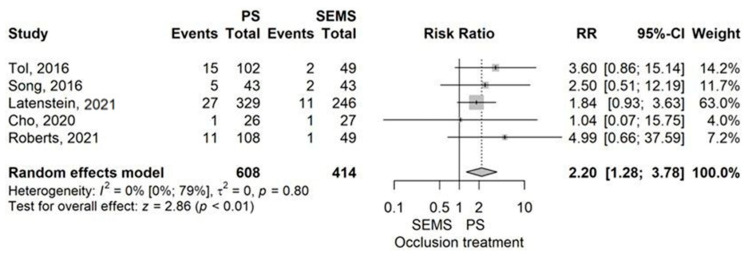
Forest plot for Catheter occlusion, comparing PS with SEMS [13,40,45,51,55].

Catheter occlusion was more frequent in the PS group than in the SEMS group, with a RR of 2.20 (95% CI 1.28–3.78), *p* = 0.004, as determined by a random-effects model (Figure 6).

No evidence of publication bias was provided (*p* = 0.482). Omitting the study by Latenstein et al. (2021) significantly influenced the outcome, as demonstrated by the leave-one-out sensitivity analysis [40]. No heterogeneity was demonstrated (I^2^ = 0% (95% CI 0–79.2%), *p* = 0.795).

Catheter exchange

No statistical difference was shown in catheter exchange rate (RR = 1.84, *p* = 0.273).

No publication bias was detected (*p* = 0.407). The studies by Tol et al. (2016) and Latenstein et al. (2021) were identified as influential studies [13,40]. Substantial heterogeneity was revealed (I^2^ = 69.5%, *p* = 0.038).

Duration of drainage

No statistically significant difference in the duration of drainage between PS and SEMS was demonstrated (RR = 1.96 days, *p* = 0.433).

No publication bias was indicated (*p* = 0.999). Omitting the study by Latenstein et al., 2021 had a significant impact on the results [40]. High heterogeneity was revealed (I^2^ = 90.9%, *p* < 0.001).

##### Postoperative Complications

Overall complication rate

No statistical difference was demonstrated in terms of the overall complication rate between PS and SEMS (RR = 0.95, *p* = 0.359).

Potential publication bias was detected (*p* = 0.038). A significant impact on the results was achieved by omitting the studies by Cavell et al. (2013) and Latenstein et al. (2021) in the leave-one-out sensitivity analysis [40,52]. There is no apparent heterogeneity among the included studies (I^2^ = 0%, *p* = 0.448).

**Figure 7:**
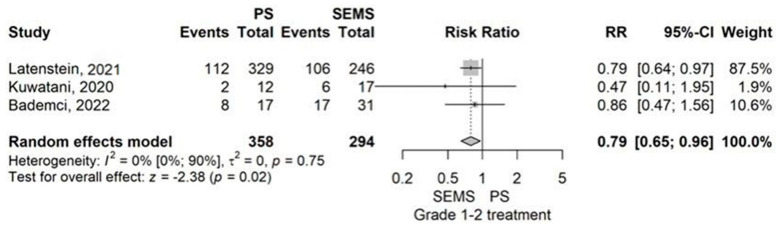
Forest plot for Grade 1–2 complication rate, comparing PS with SEMS [37,40,56].

Subgroup analysis of the Grade 1–2 complication rate revealed a significantly lower rate in the PS group compared to the SEMS group, with an RR of 0.79 (95% CI: 0.65–0.96) and *p* = 0.017 (Figure 7).

The subgroup meta-analysis of Grade ≥3 complication rates found no significant difference between the PS group and the SEMS group, with a RR value of 1.24 (95% CI 0.96–1.60), *p* = 0.096.

Infectious complications rate

No meta-analysis was performed, as only a single study (Haapamaki et al., 2015) assessed infectious complication rate in the comparison between PS and SEMS [43].

Sepsis

No meta-analysis was conducted, as only a single study (Bademci et al., 2022) assessed sepsis rate in the comparison between PS and SEMS [37].

Intraabdominal abscess

No significant difference in intraabdominal abscess risk was demonstrated (RR = 0.81, *p* = 0.559).

No publication bias was evidenced (*p* = 0.819). No influential studies were found. Low heterogeneity was suggested (I^2^ = 0%, *p* = 0.636).

Wound infection

**Figure 8:**
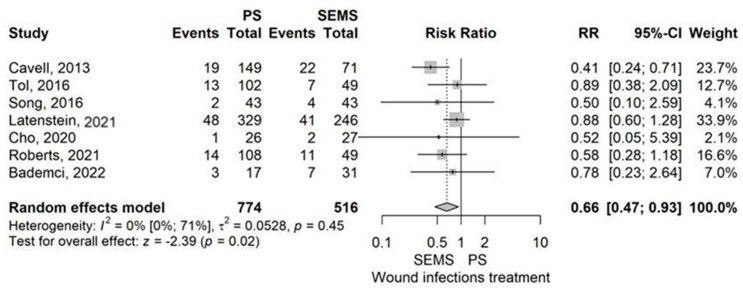
Forest plot for Wound infection rate, comparing PS with SEMS [13,37,40,45,51,52,55].

Wound infection demonstrated a lower rate in the PS group compared to the SEMS group, with an RR of 0.66 (95% CI: 0.47–0.93), *p* = 0.017, using the random-effects model (Figure 8).

No publication bias was detected by Egger’s test (*p* = 0.612). The leave-one-out sensitivity analysis revealed that omitting the studies by Cavell et al. (2013) and Latenstein et al. (2021) strongly impacted the results [40,52]. Low heterogeneity across studies was demonstrated (I^2^ = 0% (95% CI 0–70.8%), *p* = 0.45). However, the wide confidence interval (0–70.8%) indicates considerable uncertainty in this estimate.

Postpancreatectomy hemorrhage (PPH)

PPH did not demonstrate a significant difference between PS and SEMS (RR = 1.39, *p* = 0.307).

The publication bias test evidenced a statistically non-significant *p*-value of 0.671. A single study by Latenstein et al. (2021) was found to be influential [40]. Low heterogeneity was demonstrated (I^2^ = 0%, *p* = 0.664).

Chyle leak

No meta-analysis was conducted, as only a single study (Latenstein et al., 2021) assessed chyle leak in the comparison between PS and SEMS [40].

Postoperative biliary fistula (POBF)

Meta-analysis could not demonstrate a statistically significant difference in terms of POBF between PS and SEMS (RR = 0.78, *p* = 0.487).

No publication bias was indicated by Egger’s test (*p* = 0.407). Omitting Latenstein et al.’s 2021 study had a significant impact on the meta-analysis [40]. No significant heterogeneity was found (I^2^ = 0%, *p* = 0.593).

Postoperative pancreatic fistula (POPF)

**Figure 9:**
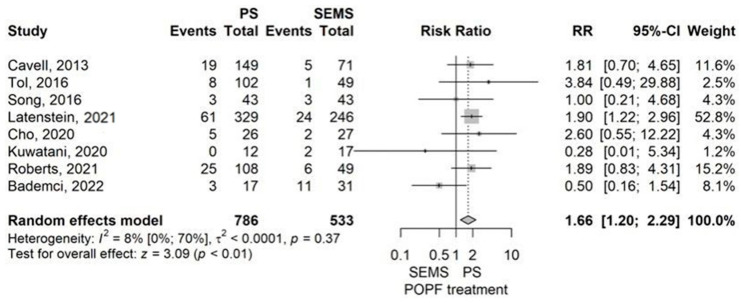
Forest plot for POPF rate, comparing PS with SEMS [13,37,40,45,51,52,55,56].

The RR value of 1.66 (95% CI 1.20–2.29) demonstrated a higher risk of POPF for the PS group compared to the SEMS, which was statistically significant (*p* = 0.002), as determined by the model with random effects (Figure 9).

The publication bias test yielded a non-significant *p*-value of 0.375. The outcomes of the meta-analysis were affected by the leave-one-out sensitivity analysis’s omission of the study of Latenstein et al. from 2021 [40]. The meta-analysis revealed low heterogeneity (I^2^ = 8.2% (95% CI 0–70.2%), *p* = 0.367). However, the wide confidence interval for I^2^ suggests some uncertainty in this assessment.

Subgroup analysis of Grade 1 POPF had not demonstrated a statistically significant difference between PS and SEMS (RR = 1.00, *p* = 1).

**Figure 10:**
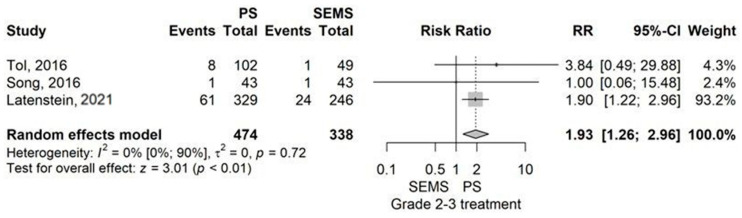
Forest plot for Grade 2–3 POPF rate, comparing PS with SEMS [13,40,55].

In contrast, PS demonstrated a higher rate of Grade 2–3 POPF than SEMS, with a RR value of 1.93 (95% CI 1.26–2.96), with statistical significance (*p* = 0.003) using the model with random effects (Figure 10).

Delayed gastric emptying (DGE)

DGE did not demonstrate a statistical difference between PS and SEMS (RR = 1.28, *p* = 0.114).

No publication bias was demonstrated (*p* = 0.231). A leave-one-out sensitivity analysis revealed that omitting the studies by Tol et al. (2016) and Latenstein et al. (2021) significantly influenced the results [13,40]. Low heterogeneity was demonstrated (I^2^ = 0%, *p* = 0.523).

##### Intraoperative Characteristics

Soft pancreas

No meta-analysis was performed for the soft pancreas rate, as only a single study (Latenstein et al., 2021) assessed it in the comparison between PS and SEMS [40].

Main biliary duct (MBD) diameter

No studies were identified that compared MBD diameter (mm) between the PS and SEMS groups.

Operative time

Mean difference (MD) between PS and SEMS was −49.03 min (95% CI: −165.14 to 67.09), but with no statistical relevance (*p* = 0.408).

The publication bias test was not applicable. The studies by Cavell et al. (2013) and Kuwatani et al. (2020) were identified as influential studies [52,56]. High heterogeneity was revealed (I^2^ = 78.6%, *p* = 0.031).

Blood loss

Blood loss (mL) had no statistical difference between PS and SEMS (MD = 259.41 (95% CI −478.46–997.29), *p* = 0.491).

The publication bias test was not applicable. The research by Cavell et al. (2013) and Kuwatani et al. (2020) had a considerable influence on the overall results [52,56]. High heterogeneity was found (I^2^ = 91%, *p* < 0.001).

##### Prognostic Factors

Hospital stay

**Figure 11:**
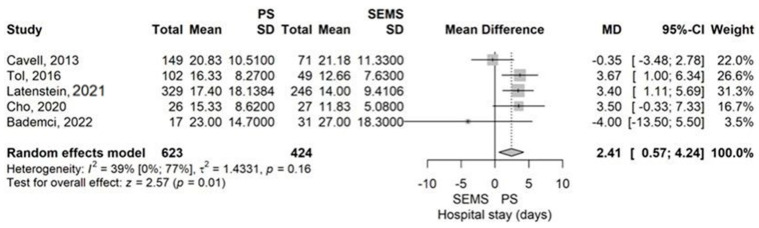
Forest plot for Hospital stay (days), comparing PS with SEMS [13,37,40,51,52].

Hospital stay was significantly longer in the PS group versus SEMS group by a MD of 2.41 days (95% CI 0.57–4.24), *p* = 0.01 using the model with random effects (Figure 11).

No publication bias was observed (*p* = 0.283). The study of Cavell et al. (2013) had demonstrated through the leave-one-out sensitivity analysis an influencing impact on the results [52]. Moderate heterogeneity was found, but not statistically significant (I^2^ = 39.1%, *p* = 0.161).

Reoperation rate

The reoperation rate was statistically similar among groups (RR = 1.03, *p* = 0.951).

No publication bias was detected (*p* = 0.727). The leave-one-out sensitivity analysis demonstrated that the studies by Haapamaki et al. (2015) and Tol et al. (2016) had a significant impact on the outcome [13,43]. There is moderate heterogeneity (I^2^ = 48.9%), but the Q test indicated that it is not statistically significant (*p* = 0.141).

Mortality

No statistically significant difference in mortality rate was found among the groups compared—PS versus SEMS (RR = 1.27, *p* = 0.654).

Absence of publication bias was tested (*p* = 0.685). The leave-one-out analysis reported Tol et al. (2016) and Latenstein et al. (2021) as influencing studies [13,40]. Low heterogeneity was demonstrated (I^2^ = 3.5%, *p* = 0.386).

### 3.3. Summarized Results

**Figure 12:**
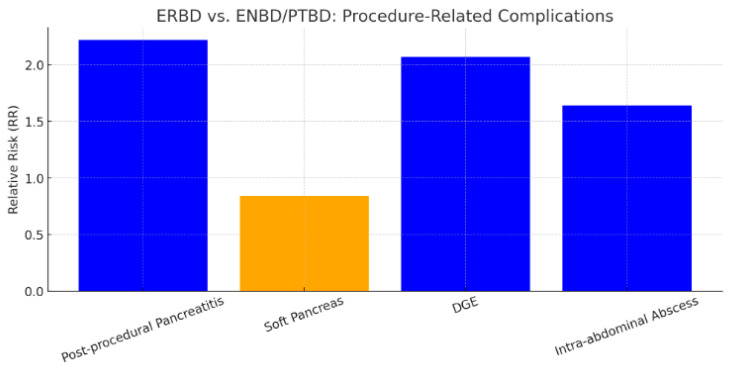
Bar graph for postprocedural and postoperative complications of ERBD versus ENBD/PTBD. Blue represents a RR > 1 and yellow represents a RR < 1.

Our meta-analysis of 15 studies suggests that ERBD may be associated with higher rates of post-procedural pancreatitis, DGE, and intra-abdominal abscess, while showing lower rates of soft pancreas (Figure 12).

**Figure 13:**
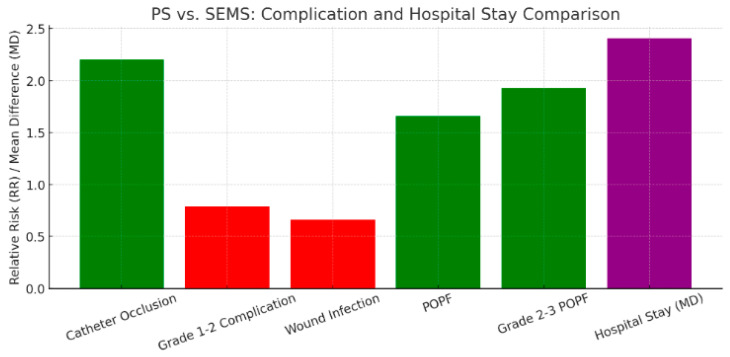
Bar graph for postprocedural and postoperative complications of PS versus SEMS. Green represents RR > 1 and red represents RR < 1. Purple represents the value of mean difference (MD) between PS and SEMS.

Our meta-analysis of 9 studies comparing plastic stents (PS) with self-expandable metal stents (SEMS) found that PS had higher rates of catheter occlusion and pancreatic fistula (POPF), particularly grade 2–3 POPF. However, PS showed lower rates of grade 1–2 complications and wound infections. Hospital stays were longer in the PS group compared to the SEMS group (Figure 13).

## 4. Discussion

Periampullary malignant tumors are only cured if surgical resection is possible and the tumoral stage permits it. Surgical treatment depends on the tumoral stage, but also on the degree of jaundice, liver function, performance status of the patient and other comorbidities and complications. Obstructive jaundice may cause altered liver function, pancreatitis, cholangitis and even sepsis and disseminated intravascular coagulation (DIC). Therefore, patients with malignant bile duct obstruction may need PBD to reduce jaundice to permit surgery in safer circumstances [4].

The role of preoperative biliary drainage (PBD) in periampullary tumors remains debated, largely due to its association with infectious perioperative complications. Current guidelines, including those from the NCCN and ESMO, discourage routine PBD in resectable disease, recommending its use only in selected circumstances such as acute cholangitis, significant hyperbilirubinemia, or anticipated surgical delay [5,6].

Multiple methods of PBD exist, including imaging-guided (cholecystostomy, PTBD), endoscopically placed (ERBD, ENBD) and surgical drainage (cholecystostomy, T-tube placement, biliodigestive bypass), for the practitioner to choose the best suited technique for the patient. Surgical drainage in significant jaundice presents the risks of general anesthesia and surgery itself, making itself limited in indications for patients with mild to moderate jaundice, when minimally invasive PBD methods are not available or feasible. Cholecystostomy may not acquire the most effective results in terms of biliary decompression, making PTBD, ERBD and ENBD the three most indicated PBD methods by professionals who treat malignant obstructions of the hepatobiliary tract [57]. In recent years, PTBD has shown a decreasing trend in practice due to a lower quality of life among patients waiting for surgery, the risk of tumoral spread and other serious complications (e.g., catheter dislodgement, bleeding, external bile loss) [58,59]. In contrast, ERBD may have a higher occurrence of postprocedural pancreatitis and cholangitis, also increasing the postoperative risk of infectious complications and POPF [12,60,61,62]. On the other hand, ENBD appears to confer perioperative advantages over ERBD: meta-analyses and cohort data report lower preoperative cholangitis, less stent dysfunction, lower overall morbidity, and reduced POPF, with no clear mortality difference; tolerability and tube dislodgement remain drawbacks, making ENBD most suitable when the interval to surgery is short [4]. However, the medical community has not yet established a consensus on the best PBD method for every situation to serve as a guideline for practitioners.

In the literature, the following question remains unanswered: What is the PBD method with the lowest associated morbidity and mortality? There are some systematic reviews and meta-analyses comparing the two PBD methods, but a consensus has not been properly established. The originality of our study is that we have made a comprehensive analysis of all the literature from three databases with the purpose of drawing a conclusion regarding which PBD technique is the best choice, with the lowest morbidity and mortality.

In our systematic review and meta-analysis, we included 24 studies: 2 RCTs, 4 prospective cohort studies and 18 retrospective cohort studies. We divided the studies into categories, considering the type of PBD: 15 compared internal drainage (ERBD) with external drainage (PTBD/ENBD). When choosing to place a biliary stent in the MBD by ERBD, it is important to choose between different types of biliary prostheses depending on the indication, procedure-related morbidity and postoperative prognosis. To answer this demand, we included 9 studies that compared PS and SEMS., which were either UCSEMS or FCSEMS.

Clinical heterogeneity among the included studies was mainly related to differences in patient age, sex distribution, type of operation performed, histological subtype, rates of neoadjuvant treatment, and pancreatic parenchymal texture, as discussed in Section 3, which should be considered when interpreting the pooled outcomes.

First, ERBD was compared with ENBD or PTBD. In terms of procedure related complications, our meta-analysis has shown an RR value of 2.22 (*p* < 0.01) of post-procedural pancreatitis for ERBD when compared to ENBD/PTBD [10,14,42,50,53]. Also, soft pancreas occurred less frequently in the ERBD group compared to ENBD/PTBD with a RR value of 0.84 (*p* = 0.03), results that can be explained by a higher rate of post-procedural pancreatitis [10,12,38,39,49,50,53]. The intraoperative consistency of the pancreas is an important indicator of postoperative prognosis in the literature, as a soft pancreas represents a risk factor for postoperative pancreatic fistula (POPF) [63]. The type of biliary drainage may also influence pancreatic parenchymal texture. Internal drainage methods, such as ERBD, are associated with higher rates of post-ERCP pancreatitis compared with external drainage [11]. It is biologically plausible that recurrent inflammation could induce localized fibrosis, potentially resulting in a firmer pancreatic texture at the time of surgery. However, direct evidence supporting this relationship is lacking, and the hypothesis should be interpreted with caution.

Our findings indicate that ERBD tended to show higher procedure-related morbidity than PTBD or ENBD, although results should be interpreted with caution due to study heterogeneity.

Compared to ENBD/PTBD, ERBD had a higher rate of delayed gastric emptying (DGE) with a RR of 2.07 (*p* < 0.01) and a higher intra-abdominal abscess rate (RR = 1.64 (*p* < 0.01) [10,12,14,15,38,41,42,44,49,50,54]

No significant differences were observed between ERBD and ENBD/PTBD in terms of blood loss, operative time, hospital stay, reoperation rate, or mortality.

In interpreting our results, we acknowledge that ENBD and PTBD differ technically—one being endoscopic, the other percutaneous. However, our decision to analyze them together as “external drainage” was data-driven and reflects the methodology of several large retrospective cohorts, including the nationwide analysis by Okano et al. (2019, n = 1942), as well as the series by Kitahata et al. (2014) and Suenaga et al. (2021), where ENBD and PTBD were explicitly reported as a combined comparator group to ERBD [44,49,53]. This approach is further supported by high-quality systematic reviews and meta-analyses that adopted the same grouping. For example, Tian et al. (2020) synthesized 10 studies (n = 1464) and demonstrated that external drainage (ENBD/PTBD) was associated with significantly lower preoperative cholangitis, stent dysfunction, and overall morbidity compared with internal stenting [64]. Similarly, meta-analyses of ENBD versus ERBD (Lin et al., 2016; Zhang et al., 2020) consistently showed advantages for ENBD, including reduced cholangitis, stent dysfunction, overall morbidity, and postoperative pancreatic fistula—outcomes more closely resembling PTBD than ERBD [4,20]. Complementary evidence from PTBD-focused syntheses (Dorcaratto et al., 2018; Duan et al., 2017) confirmed that PTBD results in fewer procedure-related complications and less cholangitis than ERBD, albeit at the cost of specific risks such as bleeding and catheter dislodgement [65,66]. Taken together, these studies provide a reproducible signal that external drainage (ENBD or PTBD) lowers the risks of cholangitis, stent dysfunction, and infectious morbidity compared with ERBD. The biological plausibility rests in the shared mechanism of continuous external bile diversion, which reduces bacterial colonization and stent-related occlusion, as supported by the correlation between contaminated bile cultures and postoperative infectious complications. Thus, grouping ENBD with PTBD preserved statistical power, aligned with precedent in the literature, and reduced small-study bias. Importantly, we emphasize that ENBD and PTBD are not identical; rather, the internal versus external comparison is intended as a pragmatic, hypothesis-generating framework supported by existing evidence. We interpret these findings cautiously, acknowledging their distinct complication spectrum, and clarify that the observed differences in delayed gastric emptying are more likely explained by time-to-surgery intervals and reduced stent dysfunction in the external group than by any direct mechanistic effect of ENBD itself.

When interpreting the comparison between PTBD and ERBD, it is important to recognize that PTBD is most often performed after failed or technically unfeasible ERCP, which introduces unavoidable selection bias. Nonetheless, several large retrospective cohorts (e.g., Okano et al., 2019; Kitahata et al., 2014; Lee et al., 2018; Byun et al., 2021) and meta-analyses (Dorcaratto et al., 2018; Duan et al., 2017) have consistently reported higher rates of cholangitis, stent dysfunction, and infectious morbidity after ERCP, with PTBD carrying specific risks such as bleeding and catheter dislodgement [44,46,48,49,65,66]. These findings are biologically plausible, as ERCP stents promote duodenobiliary reflux and bacterobilia, whereas PTBD avoids this mechanism. Therefore, while causality cannot be inferred, the PTBD versus ERCP comparison remains clinically meaningful, reflecting real-world treatment pathways and providing hypothesis-generating insights for preoperative management.

Moreover, from 9 studies comparing PS with SEMS (FCSEMS/UCSEMS), we learned that catheter occlusion (RR = 2.20, *p* < 0.01) is more common in the PS group, while grade 1–2 Clavien-Dindo complication rate (RR = 0.79, *p* = 0.017) and wound infection (RR = 0.66, *p* = 0.017) appear more rarely in the PS group [13,37,40,45,51,52,55,56]. PS had a higher rate of POPF than SEMS (RR = 1.66, *p* < 0.01) [13,37,40,45,51,52,55,56]. In addition, grade 2–3 POPF was also more frequent in the PS group than in the SEMS group (RR = 1.93, *p* < 0.01) [13,40,55]. Moreover, hospital stay was longer when a PS was placed compared to a SEMS, with a MD of 2.41 days (*p* = 0.01) [13,37,40,51,52]. As it seems from the meta-analysis, catheter occlusion as expected was higher in the PS group based on its lower caliber and strength, mild complications (grade 1–2) and wound infection rate was less common in the PS group compared to SEMS group. However, POPF rate, and also, it’s subgroup of grade 2–3 POPF rate, increased in patients where a PS was placed, prolonging their hospital stay with a mean of 2.41 days.

While our analysis suggested higher wound infection rates with plastic stents compared to SEMS, this association is unlikely to have a direct device-related effect. Plastic stents are more prone to occlusion and recurrent cholangitis, which may increase biliary contamination and indirectly predispose to postoperative infections. Thus, the observed difference should be interpreted as an infection-mediated, indirect relationship rather than a true causal effect of stent type.

There were no significant differences between PS and SEMS with respect to blood loss, operative time, reoperation rate, or mortality.

Regarding the comparison between PS and SEMS, it is important to acknowledge the potential bias introduced by small sample sizes in some of the analyzed subgroups. As a result, conclusions drawn from these subgroups should be interpreted with caution, and the absence of statistically significant differences does not necessarily imply clinical equivalence between PS and SEMS.

Although quality-of-life outcomes are clinically relevant, the included studies did not report standardized or comparable assessments, which represents an important limitation of the available evidence.

An additional limitation of this review is the lack of comparative data on economic considerations among the included studies, which prevents any conclusions regarding the cost-effectiveness of different drainage strategies.

Given the limited number of available randomized controlled trials, we included both RCTs and retrospective observational studies in the quantitative synthesis to provide a more comprehensive overview of the evidence. While we acknowledge the methodological differences between these designs, we ensured conceptual and clinical comparability across studies, performed risk of bias assessment using design-specific tools (RoB 2 and ROBINS-I), and explored study design as a potential source of heterogeneity in sensitivity analyses.

When there is an indication for preoperative biliary decompression, practitioners commonly choose between PTBD and ERBD.

It is important to acknowledge that the success and safety of ERBD are inherently dependent on the ability to achieve selective biliary cannulation during ERCP. A recent network meta-analysis by Facciorusso et al. (2021) compared various adjunctive techniques to facilitate difficult biliary cannulation and demonstrated that several advanced methods can significantly improve technical success rates while maintaining acceptable safety profiles. While our analysis does not differentiate between cannulation techniques, this factor may influence real-world outcomes of ERBD, particularly in centers with varying levels of endoscopic expertise. This represents a potential source of variability that should be considered when interpreting the pooled results [67].

In practice, starting from 1980s the minimally invasive PBD has been accomplished by ERBD more often because of its advantages over PTBD: it can obtain a biopsy (definitive histopathological diagnosis), and it can offer locoregional staging by using the ultrasonography transductor [68]. However, some studies have shown that PTBD should be recommended as the first intent to achieve PBD, with a faster decrease in cholestasis and a lower rate of catheter-related complications [14]. Three retrospective studies have demonstrated that patients who underwent PTBD had a worse overall survival compared to ERBD and no PBD, two of which linked it to peritoneal tumor seeding and carcinomatosis [54,69,70]. Another method evaluated for PBD was ENBD, which has shown a better outcome than ERBD in terms of postprocedural complications, specifically cholangitis, pancreatitis, stent occlusion, and postoperative morbidity, which could be explained by a less important ascending bacterial contamination of the bile [4,12,42,71,72].

Multiple nonrandomized studies and cohort studies have established the superiority of SEMS over plastic stents in PBD, with a lower complication rate, shorter hospital stay and fewer stent-related complications. Catheter occlusion has been reported in 0% to 15.4% when choosing SEMS, whereas plastic stent obstruction rates varied from 38% to 55% [73]. RCT by Tol et al. reported fewer complications in the SEMS group, specifically, a lower rate of catheter-related complications (migration and obstruction). Moreover, the SEMS facilitated an earlier start of neoadjuvant treatment and a longer interval of stent patency [13,74].

This study was not designed to compare preoperative biliary drainage with early surgery, nor to assess outcomes according to the duration of drainage, as these aspects have already been extensively evaluated in the literature. Rather, our focus was on comparing different PBD modalities in patients for whom drainage was already indicated. Nevertheless, future randomized controlled trials are needed to directly compare drainage methods in order to establish the optimal approach.

## 5. Conclusions

Regardless of the malignancy type causing biliary obstruction, our meta-analysis suggests that ERBD may be associated with higher rates of procedure-related and postoperative complications compared with external drainage methods (ENBD and PTBD), though these results should be interpreted cautiously in light of heterogeneity and study design limitations. Based on these findings, external drainage should be considered the preferred option in patients scheduled for early surgery, as it is associated with lower perioperative morbidity. ENBD, in particular, provides a safe drainage option when patient tolerance is not an issue, while PTBD remains a valuable alternative when endoscopic access is not feasible.

On the other hand, in scenarios requiring rapid and effective biliary decompression—such as severe cholangitis, the need for prolonged drainage, or anticipated delays to surgery including neoadjuvant therapy—ERBD should be favored, as it ensures prompt relief of obstruction and is associated with a lower risk of catheter occlusion compared to external drainage.

Moreover, our analysis showed that SEMS are associated with lower rates of catheter occlusion and moderate to severe (grade 2–3) postoperative pancreatic fistula, leading to shorter hospital stays. While PS were linked to fewer minor complications and wound infections, the higher risk of severe POPF and longer hospitalization favors the use of SEMS in the preoperative setting.

It should be noted that the majority of available evidence originates from observational studies, subject to inherent selection bias. Given that most included studies were retrospective cohorts, these findings should be viewed as hypothesis-generating rather than definitive, and further high-quality randomized trials are needed to strengthen causal inference. Nevertheless, in accordance with international guidelines, PBD should be reserved for selected indications (e.g., acute cholangitis, markedly elevated bilirubin, or planned delay in surgery), while upfront surgery without drainage remains the standard in resectable cases without absolute indications.

Ultimately, the choice of preoperative biliary drainage technique should be individualized, considering the estimated duration of drainage, the need for neoadjuvant therapy, the clinical urgency of decompression, and tumor stage at presentation, in order to maximize the benefits and minimize the drawbacks of each approach.

## 6. Limitation of the Study

There are several limitations in this article. First, most articles found in our systematic search were retrospective cohort studies. With the advantage of common clinical practice applicability, retrospective studies are exposed to selection bias, in contrast to RCTs. Second, some of the subgroup analysis from the articles compared presented a certain degree of heterogeneity (low, moderate or high), while some had no heterogeneity demonstrated by I^2^ and Q test. However, given the fact that clinical heterogeneity was present among the studies compared, the model for random effects was applied in all the variables compared in our meta-analysis. Moreover, the number of subgroups needed to compare PBD methods two by two, made it impossible to have at least 2 articles in every group. Some secondary variables (e.g., soft pancreas, main biliary duct diameter) were not in the initial protocol but were added during data extraction for their consistent reporting and clinical relevance; however, all outcomes were defined using standardized international criteria, ensuring consistency across studies. On this matter, more studies that compare PBD techniques should be conducted in the future, preferably RCTs, in order to include more subjects in a future meta-analysis. Furthermore, our meta-analysis did not include the comparison between straight surgery and surgery after a PBD placement, considering the numerous groups that would have been formed for the meta-analysis. Nonetheless, our systematic search in three different databases did not find original studies comparing several surgical preoperative biliary drainage techniques, such as cholecystostomy, T-tube placement in the MBD and biliodigestive derivation (e.g., choledocoduodenostomy, hepaticojejunostomy) with minimally invasive techniques (ERBD, ENBD, or PTBD).

## Figures and Tables

**Table 1:** Characteristics of articles.

No.	Author Name	Year of Publication	Type of Study	Population	Groups Compared	Age (Years)	Neoadjuvant Therapy n (%)	MBD Diameter (mm)	Soft Pancreas n (%)
1	Bademci et al. [37]	2022	Retrospective Cohort Study	48	PS N = 17 SEMS N = 31	69 vs. 70 (*p* = 0.773)	-	-	-
2	Satoh et al. [38]	2022	Retrospective Cohort Study	156	ERBD N = 117 ENBD N = 39	70 vs. 71 (*p* = 0.217)	-	3 vs. 4 (*p* = 0.327)	73 (62%) vs. 26 (67%) (*p* = 0.63)
3	Subasi et al. [39]	2022	Retrospective Cohort Study	75	ERBD N = 42 PTBD N = 33	62.9 vs. 65.2 (*p* = 0.523)	-	3.7 vs. 4.3 (*p* = 0.557)	14 (33.3%) vs. 13 (39.4%) (*p* = 0.607)
4	Latenstein et al. [40]	2021	Retrospective Cohort Study	575	PS N = 329 SEMS N = 246	67 vs. 68 (*p* = 0.117)	**17 (5.2%) vs. 31 (12.6%) (*p* = 0.011)**		**209 (63.5%) vs. 121 (49.2%) (*p* = 0.001)**
5	Han et al. [41]	2021	Retrospective Cohort Study	88	ERBD N = 42 ENBD N = 46	65 vs. 67 (*p* = 0.529)	-	-	-
6	Huang et al. [42]	2015	Retrospective Cohort Study	100	ERBD N = 37 ENBD N = 18 PTBD N = 45	58.1 vs. 60.6 vs. 57.5 (*p* = 0.56)	-	-	-
7	Haapamäki et al. [43]	2015	Retrospective Cohort Study	191	PS N = 163 SEMS N = 28	64 vs. 64	22 (20%) vs. 3 (14%)	-	-
8	Okano et al. [44]	2019	Retrospective Cohort Study	1942	ERBD N = 1170 ENBD/PTBD N = 772	**64 vs. 62 (*p* = 0.025)**	-	-	-
9	Roberts et al. [45]	2021	Retrospective Cohort Study	157	PS N = 108 SEMS N = 49	66.3 vs. 67.2 (*p* = 0.63)	**2 (1.9%) vs. 9 (18.4%) (<0.001)**	-	-
10	Lee et al. [46]	2018	Retrospective Cohort Study	569	ERBD N = 335 PTBD N = 234	63.1 vs. 63.5 (*p* = 0.609)	-	-	-
11	Mori et al. [47]	2019	Retrospective Cohort Study	84	ERBD N = 60 PTBD N = 24	68 vs. 73 (*p* = 0.156)	20 (33%) vs. 9 (37%) (*p* = 0.717)	-	-
12	Byun et al. [48]	2021	Retrospective Cohort Study	167	ERBD N = 106 PTBD N = 61	62 vs. 64 (*p* = 0.805)	-	-	-
13	Park et al. [14]	2011	Retrospective Cohort Study	77	ERBD N = 34 PTBD N = 43	63.7 vs. 65.9 (*p* = 0.305)	-	-	-
14	Kitahata et al. [49]	2014	Retrospective Cohort Study	127	ERBD N = 67 ENBD/PTBD N = 60	68 vs. 70 (*p* = 0.333)	3 vs. 0 (*p* = 0.144)	-	35 vs. 31 (*p* = 0.949)
15	El-Haddad et al. [50]	2021	Prospective Cohort Study	64	ERBD N = 34 PTBD N = 30	53.3 vs. 54 (*p* = 0.79)	-	-	7 (23%) vs. 14 (46.7%) (*p* = 0.16)
16	Fujii et al. [12]	2015	Prospective Cohort Study	122	ERBD N = 72 ENBD N = 50	67 vs. 66.5 (*p* = 0.682)	-	4.9 vs. 4.3 (*p* = 0.180)	21 vs. 19 (*p* = 0.307)
17	Cho et al. [51]	2020	Prospective RCT	53	PS N = 26 UCSEMS N = 27	69 vs. 67 (*p* = 0.936)	-	-	-
18	Tol et al. [13]	2016	Prospective Cohort Study	151	PS N = 102 FCSEMS N = 49	64.7 vs. 67.5	-	-	-
19	Cavell et al. [52]	2013	Retrospective Study	220	PS N = 149 SEMS N = 71	68 vs. 67 (*p* = 0.858)	**10 (7.4%) vs. 23 (34.3%) (*p* < 0.001)**	-	-
20	Suenaga et al. [53]	2021	Prospective Cohort Study	78	ERBD N = 40 ENBD/PTBD N = 38	67 vs. 70 (*p* = 0.084)	13 (33%) vs. 5 (13%) (*p* = 0.060)	-	13 (33%) vs. 20 (53%) (*p* = 0.072)
21	Uemura et al. [54]	2015	Retrospective Cohort Study	573	ERBD N = 407 PTBD N = 166	67 vs. 67	203 (50%) vs. 79 (49%) (*p* = 0.957)	-	-
22	Song et al. [55]	2016	Prospective RCT	86	PS N = 43 FCSEMS N = 43	65.72 vs. 65.67 (*p* = 0.982)	-	-	-
23	Zhang et al. [10]	2017	Retrospective Cohort Study	153	ENBD N = 102 ERBD N = 51	55.26 vs. 56.24 (*p* = 0.542)	-	1.73 vs. 1.78 (*p* = 0.540)	56 (54.9%) vs. 22 (43.1%) (*p* = 0.170)
24	Kuwatani et al. [56]	2020	Retrospective Cohort Study	29	SEMS N = 17 PS = 12	66 vs. 68 (*p* = 0.824)	-	-	-
Total				5894					

ENBD: endoscopic nasobiliary drainage; ERBD: Endoscopic retrograde biliary drainage; PS: plastic stent; PTBD: Percutaneous transhepatic biliary drainage; SEMS: self-expanding metal stents (FCSEMSs: fully covered SEMSs and UCSEMS: uncovered SEMS); RCT: randomized controlled trial. Values presented in bold indicate results with a *p* < 0.05 and therefore represent statistically significant differences.

## Data Availability

All data were obtained from the databases. The author has sorted out all the data and attached them to the attachment at the end of the article.

## Supplementary Materials

The following supporting information can be downloaded at: https://www.mdpi.com/article/10.3390/jcm14197097/s1, Supplementary Material S1: Search strategy; Supplementary Material S2: Risk of bias assessment; Supplementary Material S3: Mean and standard deviation; Supplementary Material S4: PRISMA 2020 checklist; Supplementary Material S5: Qualitative variables-RR with 95%CI; Supplementary Material S6: Quantitative variables-Mean differences.
