# Supplementary material for: A Systematic Review and Meta-Analysis of Preoperative Biliary Drainage Methods in Periampullary Tumors
Source: J Clin Med. 2025 Oct 8;14(19):7097. doi: 10.3390/jcm14197097 (PMC12524691; doi:10.3390/jcm14197097)
Supplement: Supplementary file 1 [file jcm-14-07097-s001.zip › Supplementary material 1-Search strategy.pdf]

Item	Details
**Databases searched**	PubMed (MEDLINE), BMC, Scopus
**Search timeframe**	January 2010 – December 2023
**Language restriction**	English
**Population**	Human subjects
**Search terms – PubMed**	(“preoperative biliary drainage” OR “percutaneous transhepatic biliary drainage” OR “T-tube drainage” OR “cholecystostomy” OR “biliodigestive derivation”) AND (“pancreaticoduodenectomy” OR “Whipple procedure” OR “total pancreatectomy”)
**Search terms – BMC and Scopus**	“Preoperative biliary drainage” AND “pancreaticoduodenectomy”
**Inclusion criteria**	Title/Abstract screening:<br>• Type of studies: original articles<br>• Design of studies: observational cohort studies (prospective/retrospective) or randomized controlled trials,<br>• Accesability: open access,<br>• Language of publication: english<br>• Subjects: human<br>• Timeframe of publication: January 2010 - December 2023<br>Full – text screening:<br>• Subject: comparison of two or more preoperative drainage methods in pancreatic surgery<br>• Pathology: Periampullary tumors,<br>• Outcomes: patients’ characteristics (e.g., soft pancreas, main biliary duct (MBD) diameter), postprocedural complications (e.g., drainage duration, pancreatitis, cholangitis, perforation, hemorrhage, stent occlusion, catheter exchange), postoperative complications (e.g., sepsis, abscess, wound infection, post-pancreatectomy hemorrhage, chyle leak, POBF, POPF, delayed gastric emptying), intraoperative and postoperative outcomes (e.g., operative time, blood loss, hospital stay, mortality, reoperation)
**Exclusion criteria**	Title/Abstract screening:<br>• Type of studies: non-original studies (e.g. reviews, editorials, comments, erratum)

**Item**	**Details**
	• Design of studies: systematic reviews/meta-analysis, case reports<br>• Language of publication: other than English<br>• Subjects: animals<br>• Accesability: non-open access, inaccessible full texts,<br>• Timeframe of publication: earlier than 2010<br><br>Full- text screening:<br><br>• Subject: articles not addressing preoperative biliary drainage in pancreatic surgery, articles not comparing two or more preoperative drainage methods<br>• Pathology: other than periampullary tumors<br>• Outcomes: article articles not comparing two or more preoperative drainage methods,<br>• Number of studies on a comparison: singular studies
**Screening process**	Performed by two independent reviewers; discrepancies resolved through discussion
**Duplicate removal**	Duplicates identified and removed manually by both reviewers using Microsoft Excel and verified with DOI/PMID; Mendeley used for citation management
**Data management**	Eligible studies compiled in Microsoft Excel; tables and statistical functions used to organize and analyze data
