# Supplementary material for: A Systematic Review and Meta-Analysis of Preoperative Biliary Drainage Methods in Periampullary Tumors
Source: J Clin Med. 2025 Oct 8;14(19):7097. doi: 10.3390/jcm14197097 (PMC12524691; doi:10.3390/jcm14197097)
Supplement: Supplementary file 1 [file jcm-14-07097-s001.zip › Supplementary material 2-Risk of bias assessment.pdf]

Risk of bias assessment among studies:

**RoB 2: A revised Cochrane risk-of-bias tool for randomized trials**:

1. Cho, J.H.; Yoon, Y.-S.; Kim, E.J.; Kim, Y.S.; Cho, J.Y.; Han, H.-S.; Park, Y.H.; Shin, D.W.; Lee, J.-C.; Hwang, J.-H.; et al. A Multicenter Prospective Randomized Controlled Trial for Preoperative Biliary Drainage with Uncovered Metal versus Plastic Stents for Resectable Periampullary Cancer. *J Hepatobiliary Pancreat Sci* **2020**, *27*, 690–699, doi:10.1002/jhbp.811.

    - Random sequence generation (selection bias) - Low risk - Design is labeled as a "randomized controlled trial" with no evidence of inappropriate sequence methods.
    - Allocation concealment (selection bias) - Unclear risk - The abstract does not specify methods such as sealed envelopes or central randomization. No details are provided on concealment processes.
    - Blinding of participants and personnel (performance bias) - High risk / Unclear - Impossible to blind stents (metal vs plastic) from endoscopists or surgical teams. The study doesn't report any masking of operators or outcome assessors.
    - Blinding of outcome assessment (detection bias) - Unclear risk - No information on whether outcome assessment (e.g., complication rates, reinterventions) was conducted blinded to stent type.
    - Incomplete outcome data (attrition bias) - Low risk - Of 60 randomized, 53 analyzed—likely due to pre-planned dropout or protocol violations, but no evidence of differential attrition.
    - Selective reporting (reporting bias) - Low risk - Primary (reintervention) and secondary outcomes (complications, bilirubin, surgery wait time, stay) are fully reported as per protocol.
    - Other bias - Low risk - Multicenter design reduces center-specific biases; baseline characteristics reportedly balanced. No evidence of funding influence or early stopping.

2. Song, T.J.; Lee, J.H.; Lee, S.S.; Jang, J.W.; Kim, J.W.; Ok, T.J.; Oh, D.W.; Park, D.H.; Seo, D.W.; Lee, S.K.; et al. Metal versus Plastic Stents for Drainage of Malignant Biliary Obstruction before Primary Surgical Resection. *Gastrointest Endosc* **2016**, *84*, 814–821, doi:10.1016/j.gie.2016.04.018

    - Random sequence generation (selection bias) - Low risk - Described as a "prospective randomized trial," with participants randomly assigned to either plastic or fully covered self-expandable metal stents
    - Allocation concealment (selection bias) - Unclear risk - The abstract and methods do not detail the mechanism used to conceal the allocation sequence, such as sealed envelopes or centralized randomization.
    - Blinding of participants and personnel (performance bias) - High risk - Due to the nature of the intervention (visible stent types), blinding of endoscopists, surgical teams, and patients was infeasible. No attempt at masking was.

- Blinding of outcome assessment (detection bias) - Unclear risk -There is no indication whether outcome assessors (evaluating complications, reinterventions) were blinded to stent allocation.
- Incomplete outcome data (attrition bias) - Low risk- The flow appears balanced, with no evidence of differential dropout; both arms had similar follow-up, and outcomes were reported in all randomized participants
- Selective reporting (reporting bias) - Low risk - Key outcomes—including adverse events, reintervention, surgery interval, and surgical complications—were fully reported as planned
- Other biases - Low risk - A multicenter design and balanced baseline characteristics minimize bias. No funding or early stopping concerns were noted

**Risk Of Bias In Non-Randomized Studies - of Interventions (ROBINS-I)**

1. Bademci, R.; Temidayo Talabi, M.O.; Salas, P.; Blanco, M.R.; Riart, G.C.; Bollo, J.; Raventós, V.A. Impact of Biliary Drainage Prior to Pancreatectomy. *Acta Chir Belg* **2022**, *122*, 390–395, doi:10.1080/00015458.2021.1920659.

    - Confounding bias – due to unmeasured differences between groups (e.g., disease severity).
    - Selection bias – from non-random assignment to biliary drainage or no drainage.
    - Classification bias – low risk; interventions were clearly classified.
    - Performance bias – low risk; no deviation from standard care post-intervention.
    - Attrition bias – low risk; minimal or no missing data reported.
    - Detection bias – moderate risk; outcomes likely assessed without blinding.
    - Reporting bias – low risk; all relevant outcomes appear reported.
    - Overall bias - Moderate risk

2. Satoh, D.; Matsukawa, H.; Shiozaki, S. The Optimal Type and Management of Biliary Drainage in Patients With Obstructive Jaundice Who Undergo Pancreaticoduodenectomy. In Vivo 2022, 36, 391–397, doi:10.21873/invivo.12716.

    - Confounding bias – moderate risk; no randomization, potential for baseline differences (e.g., severity, comorbidity) between ERBD and ENBD groups.
    - Selection bias – moderate risk; treatment selection was based on clinician/endoscopist choice rather than protocol.
    - Classification bias – low risk; intervention type clearly recorded (ERBD vs ENBD).
    - Performance bias (deviations from intended interventions) – low risk; standard care likely consistent post-PBD across groups.
    - Missing data bias – low risk; no evidence of differential or significant missing information.
    - Detection bias (measurement of outcomes) – moderate risk; outcome assessors likely aware of intervention, no blinding reported.
    - Selective reporting bias – low risk; outcomes consistently reported including Clavien-Dindo complications.
    - Overall bias – Moderate risk

3. Subasi, O.; Ercan, M.; Aziret, M.; Biricik, A.; Kahraman, Y.S.; Altıntoprak, F.; Celebi, F.; Karaman, K. Effects of Preoperative Biliary Drainage Methods and Time to Postoperative Complications after Biliary Drainage in Peri-ampullary Tumors. Ann Ital Chir 2022, 93, 403–409.
    - Confounding bias: Moderate – there is a significant risk of baseline differences (e.g., symptom severity, bilirubin levels, comorbidities) influencing group allocation and outcomes.
    - Selection bias: Moderate – patients were assigned to drainage types based on clinical decisions rather than protocol, introducing potential selection bias.
    - Classification bias: Low – biliary drainage methods (none, endoscopic, percutaneous) were clearly defined and consistently recorded.
    - Performance bias (deviations from intended interventions): Low – treatment after intervention was not modified by the study and followed standard care.
    - Missing data bias: Low – retrospective chart review included nearly all patients (n=122), with no evidence of systematic attrition.
    - Detection bias (measurement of outcomes): Moderate – postoperative complications likely assessed by unblinded clinicians based on medical records.
    - Selective reporting bias: Low – results for postoperative morbidity and mortality across groups and intervals were fully reported.
    - Overal bias – Moderate risk

4. Latenstein, A.E.J.; Mackay, T.M.; van Huijgevoort, N.C.M.; Bonsing, B.A.; Bosscha, K.; Hol, L.; Bruno, M.J.; van Coolsen, M.M.E.; Festen, S.; van Geenen, E.; et al. Nationwide Practice and Outcomes of Endoscopic Biliary Drainage in Resectable Pancreatic Head and Periampullary Cancer. HPB (Oxford) 2021, 23, 270–278, doi:10.1016/j.hpb.2020.06.009.

    - Confounding bias: Moderate – despite multivariable regression, unmeasured confounders (e.g., center variation, stent type preference) may still influence outcomes
    - Selection bias: Moderate – patients were selected based on clinical judgment and audit inclusion, not randomized assignment.
    - Classification bias: Low – clear documentation of stent type (SEMS vs plastic) from audit data
    - Performance bias (deviations from intended interventions): Low – post-drainage management likely followed standard clinical protocols across centers.
    - Missing data bias: Low – audit dataset is comprehensive; any missing data appeared randomly distributed
    - Detection bias (measurement of outcomes): Moderate – outcomes such as complications were extracted from clinical records without assessor blinding .
    - Selective reporting bias: Low – all key outcomes (EBD complications, postoperative fistula, hospital stay) are reported as planned
    - Overall bias: Moderate risk.

5. Han, S.H.; Kim, J.S.; Hwang, J.W.; Kim, H.S. Preoperative Endoscopic Retrograde Biliary Drainage Increases Postoperative Complications after Pancreaticoduodenectomy Compared to Endoscopic Nasobiliary Drainage. Gland Surg 2021, 10, 1669–1676, doi:10.21037/gs-20-711
    - Confounding bias – moderate risk; non-randomized comparison may reflect differences in patient health or tumor characteristics influencing both drainage type and outcomes
    - Selection bias – moderate risk; allocation to ERBD or ENBD was based on clinical preference, not protocol.
    - Classification bias – low risk; drainage method clearly defined (ERBD vs ENBD).
    - Performance bias – low risk; post-PBD care likely consistent across groups.
    - Missing data bias – low risk; clinical records appear complete for all 88 pre-drained patients.
    - Detection bias – moderate risk; postoperative complications were assessed from records without blinding
    - Selective reporting bias – low risk; reported outcomes include POPF, PPH, overall complications.
    - Overall bias: Moderate risk

6. Huang, X.; Liang, B.; Zhao, X.-Q.; Zhang, F.-B.; Wang, X.-T.; Dong, J.-H. The Effects of Different Preoperative Bili-ary Drainage Methods on Complications Following Pancreaticoduodenectomy. Medicine 2015, 94, e723, doi:10.1097/MD.0000000000000723.
    - Confounding bias – moderate risk; non-randomized comparison may reflect differences in patient health or tumor characteristics influencing both drainage type and outcomes
    - Selection bias – moderate risk; allocation to ERBD or ENBD was based on clinical preference, not protocol.
    - Classification bias – low risk; drainage method clearly defined (ERBD vs ENBD).
    - Performance bias – low risk; post-PBD care likely consistent across groups.
    - Missing data bias – low risk; clinical records appear complete for all 88 pre-drained patients.
    - Detection bias – moderate risk; postoperative complications were assessed from records without blinding
    - Selective reporting bias – low risk; reported outcomes include POPF, PPH, overall complications.
    - Overall bias:  Moderate risk

7. Haapamäki, C.; Seppänen, H.; Udd, M.; Juuti, A.; Halttunen, J.; Kiviluoto, T.; Sirén, J.; Mustonen, H.; Kylänpää, L. Preoperative Biliary Decompression Preceding Pancreaticoduodenectomy with Plastic or Self-Expandable Metal-lic Stent. Scand J Surg 2015, 104, 79–85, doi:10.1177/1457496914543975.
    - Confounding bias – Moderate risk: Retrospective design with no randomization; patient characteristics (e.g., obstruction severity, comorbidities) may differ between plastic-stent, SEMS, and unstented groups
    - Selection bias – Moderate risk: Intervention decisions made clinically rather than per protocol—susceptible to selection bias.

- Classification bias – Low risk: Biliary drainage method (plastic vs SEMS vs none) clearly and accurately recorded in medical records
- Performance bias (deviations from intended interventions) – Low risk: Management post–drainage likely followed standard care without systematic differences.
- Missing data bias – Low risk: Large sample of 366 patients with no indication of significant missing or differential follow-up
- Detection bias (measurement of outcomes) – Moderate risk: Outcomes such as stent failure, bilirubin levels, and postoperative complications were based on chart review; assessors were likely unblinded.
- Selective reporting bias – Low risk: All reported outcomes (e.g., stent failure, infection, fistula) appear to have been included in full as described in the abstract and results
- Overall bias – Moderate risk

8. Okano, K.; Suzuki, Y. Influence of Bile Contamination for Patients Who Undergo Pancreaticoduodenectomy after Biliary Drainage. *World J Gastroenterol* **2019**, *25*, 6847–6856, doi:10.3748/wjg.v25.i47.6847.
    - Confounding bias – Moderate: Retrospective cohort; although multivariable regression adjusted for factors, unmeasured variables (e.g., timing/type of drainage) could influence postoperative infection outcomes
    - Selection bias – Moderate: Patients undergoing drainage and those with contamination were selected based on clinical indications, not by protocol assignment.
    - Classification bias – Low: Bile contamination status was clearly defined and documented based on bile culture results at the time of drainage.
    - Performance bias (deviations from intended interventions) – Low: Post-drainage management and perioperative care were standard and unlikely to differ between contaminated and non-contaminated groups.
    - Missing data bias – Low: Uses a large cohort (n≈4,101) from institutional records; minimal missing data reported.
    - Detection bias (measurement of outcomes) – Moderate: Postoperative complications (e.g., POPF, wound infection) were identified from clinical records with no indication of assessor blinding
    - Selective reporting bias – Low: Key outcomes—postoperative infections, POPF Grade B/C, bloodstream contamination—were reported as per objectives.
    - Overall bias – Moderate risk

9. Roberts, A.T.; Jaya, J.; Ha, P.; Thakur, U.; Aldridge, O.; Pilgrim, C.H.C.; Tan, E.; Wong, E.; Fox, A.; Choi, J.; et al. Metal Stents Are Safe and Cost-effective for Preoperative Biliary Drainage in Resectable Pancreaticobiliary Tu-mours. ANZ J Surg 2021, 91, 1841–1846, doi:10.1111/ans.17060.
    - Confounding bias – Moderate: Treatment groups likely differed by baseline factors (e.g., bilirubin levels, comorbidities), though adjusted in multivariable analysis.
    - Selection bias – Moderate: Choice of SEMS vs PS based on clinical indication rather than randomized assignment.

- Classification bias – Low: Stent type (SEMS vs PS) was clearly and consistently documented.
- Performance bias (deviations from intended interventions) – Low: Post-drainage care followed standard protocols across centers.
- Missing data bias – Low: Comprehensive dataset (n = 157) with minimal missing follow-up.
- Detection bias (measurement of outcomes) – Moderate: Complications were determined from clinical records without blinded assessment
- Selective reporting bias – Low: All predefined outcomes (PBD complications, re-intervention, postoperative events, cost) were fully reported
- Overall bias – Moderate risk

10. Lee, H.; Han, Y.; Kim, J.R.; Kwon, W.; Kim, S.-W.; Jang, J.-Y. Preoperative Biliary Drainage Adversely Affects Sur-gical Outcomes in Periampullary Cancer: A Retrospective and Propensity Score-Matched Analysis. J Hepatobili-ary Pancreat Sci 2018, 25, 206–213, doi:10.1002/jhbp.529.
    - Confounding bias – Moderate: Propensity score matching used, but unmeasured confounding remains possible.
    - Selection bias – Moderate: Non-random assignment to PBD vs no-PBD based on clinical criteria.
    - Classification bias – Low: PBD status clearly defined and documented.
    - Performance bias (deviations from intended interventions) – Low: Postoperative care consistent across groups.
    - Missing data bias – Low: Complete dataset; no significant attrition.
    - Detection bias (measurement of outcomes) – Moderate: Outcomes assessed retrospectively with likely unblinded assessors.
    - Selective reporting bias – Low: All prespecified outcomes reported.
    - Overall bias – Moderate risk

11. Mori, S.; Aoki, T.; Park, K.-H.; Shiraki, T.; Sakuraoka, Y.; Iso, Y.; Kato, M.; Kubota, K. Impact of Preoperative Percu-taneous Transhepatic Biliary Drainage on Post-Operative Survival in Patients with Distal Cholangiocarcinoma. ANZ J Surg 2019, 89, E363–E367, doi:10.1111/ans.15329.
    - Confounding bias – Moderate: As a retrospective cohort, patient characteristics (e.g., tumor stage, bilirubin, comorbidities) may differ and influence survival outcomes.
    - Selection bias – Moderate: Assignment to PTBD or no-PTBD was based on clinical judgment and institutional practice, not randomized.
    - Classification bias – Low: Drainage status (PTBD vs none) was clearly defined and reliably extracted from patient records.
    - Performance bias (deviations from intended interventions) – Low: Post-drainage and perioperative care appeared standardized across groups.
    - Missing data bias – Low: No indication of differential or significant missing data; records complete for the selected cohort.
    - Detection bias (measurement of outcomes) – Moderate: Survival and complication data were recorded from medical records without assessor blinding.

- Selective reporting bias – Low: Main outcomes (survival, complications) were reported as intended.
- Overall bias – Moderate risk

12. Byun, Y.; Kwon, W.; Han, Y.; Choi, Y.J.; Kang, J.S.; Kim, H.; Jang, J.-Y. Adverse Oncologic Effects of Preoperative Biliary Drainage on Early Stage Ampulla of Vater Cancer. HPB (Oxford) 2021, 23, 253–261, doi:10.1016/j.hpb.2020.06.005.
    - Confounding bias – Moderate: Although multivariate analysis was used, unmeasured factors (e.g., tumor biology, surgical timing) may have influenced both PBD selection and survival.
    - Selection bias – Moderate: PBD was chosen based on clinical presentation (e.g., jaundice, cholangitis), not randomly assigned.
    - Classification bias – Low: PBD status (ERBD vs PTBD vs none) was clearly defined and reliably recorded.
    - Performance bias – Low: Post-drainage and perioperative care followed institutional protocols with no indication of differential treatment.
    - Missing data bias – Low: Large cohort (n=313) with complete outcome data; minimal loss to follow-up reported.
    - Detection bias – Moderate: Outcomes including postoperative complications and disease-free survival were assessed retrospectively without assessor blinding
    - Selective reporting bias – Low: All planned outcomes—including 5-year disease-free survival and postoperative complications—were reported per study objectives.
    - Overall bias – Moderate risk

13. Park, S.-Y.; Park, C.-H.; Cho, S.-B.; Lee, W.-S.; Kim, J.-C.; Cho, C.-K.; Joo, Y.-E.; Kim, H.-S.; Choi, S.-K.; Rew, J.-S. What Is Appropriate Procedure for Preoperative Biliary Drainage in Patients with Obstructive Jaundice Awaiting Pancreaticoduodenectomy? *Surg Laparosc Endosc Percutan Tech* **2011**, *21*, 344–348, doi:10.1097/SLE.0b013e3182318d2f.
    - Confounding bias – Moderate: Retrospective comparison of PTBD (n=34) vs EBD (n=43); patient characteristics like bilirubin, cholangitis severity, and timing may differ and impact outcomes.
    - Selection bias – Moderate: Type of drainage chosen based on clinical factors (e.g., availability, clinician preference), not randomized.
    - Classification bias – Low: Drainage type clearly recorded (PTBD vs EBD) from medical records.
    - Performance bias (deviations from intended interventions) – Low: Post-drainage care followed usual clinical protocols for both groups.
    - Missing data bias – Low: No indication of significant missing outcome data in the cohort (n=77).
    - Detection bias (measurement of outcomes) – Moderate: Outcomes (bilirubin decline, catheter complications, surgery interval) were assessed from records without blinding.
    - Selective reporting bias – Low: Key predefined outcomes—all reported as planned (e.g., bilirubin drop, complication rates, waiting time).
    - Overall bias – Moderate risk

14. Kitahata, Y.; Kawai, M.; Tani, M.; Hirono, S.; Okada, K.; Miyazawa, M.; Shimizu, A.; Yamaue, H. Preoperative Cholangitis during Biliary Drainage Increases the Incidence of Postoperative Severe Complications after Pancre-aticoduodenectomy. Am J Surg 2014, 208, 1–10, doi:10.1016/j.amjsurg.2013.10.021.
    - Confounding bias – Moderate: As a retrospective cohort of 127 patients (external vs internal drainage), there may be baseline differences (e.g., cholangitis severity, bilirubin levels, comorbidities) between groups.
    - Selection bias – Moderate: Group allocation (internal vs external drainage) was based on clinical decision-making, not randomized.
    - Classification bias – Low: Drainage type and cholangitis status were clearly defined and consistently recorded.
    - Performance bias (deviations from intended interventions) – Low: Post-drainage management and perioperative care likely followed institutional standards without group-based deviation.
    - Missing data bias – Low: No indication of significant missing data; all 127 patients were included in the analysis.
    - Detection bias (measurement of outcomes) – Moderate: Outcomes (e.g., severe complications, DGE) were retrospectively extracted without blinding of assessors.
    - Selective reporting bias – Low: All key outcomes (severe complication rates, DGE) were reported as intended.
    - Overall bias – Moderate risk

15. El-Haddad, H.M.; Sabry, A.A.; Shehata, G.M. Endoscopic versus Percutaneous Biliary Drainage for Resectable Pancreatic Head Cancer with Hyperbilirubinemia and Impact on Pancreaticoduodenectomy: A Randomized Controlled Study. Int J Surg 2021, 93, 106043, doi:10.1016/j.ijsu.2021.106043.

- Confounding bias – Moderate: The study is labeled as randomized, but insufficient detail is provided on randomization method or covariate balance; potential baseline imbalances (e.g., bilirubin level, ECOG status) could influence results.
- Selection bias – Moderate: Lack of clarity on allocation concealment raises concerns that participant selection may not have been entirely free from bias.
- Classification bias – Low: Intervention status (ERBD vs PTBD) was clearly defined and appropriately documented.
- Performance bias (deviations from intended interventions) – Low: All patients received allocated drainage and standard surgical management without reported deviations.
- Missing data bias – Low: Outcomes were reported for all or nearly all randomized patients; no indication of differential loss.
- Detection bias (measurement of outcomes) – Moderate: No mention of blinding of outcome assessors, which could affect subjective outcomes like operative difficulty and complications.
- Selective reporting bias – Low: Key clinical outcomes, including complications and operative metrics, were fully reported in line with objectives.
- Overall bias – Moderate risk

16. Fujii, T.; Yamada, S.; Suenaga, M.; Kanda, M.; Takami, H.; Sugimoto, H.; Nomoto, S.; Nakao, A.; Kodera, Y. Pre-operative Internal Biliary Drainage Increases the Risk of Bile Juice Infection and Pancreatic Fistula after Pancre-atoduodenectomy: A Prospective Observational Study. Pancreas 2015, 44, 465–470, doi:10.1097/MPA.0000000000000265.
- Confounding bias – Moderate: Non-randomized allocation between ERBD (n=72) and ENBD (n=50); although multivariate analysis was used, unmeasured baseline factors (e.g., cholangitis severity, stent duration) may have influenced outcomes.
- Selection bias – Moderate: Drainage method determined by clinical judgment rather than a protocol, leading to potential systematic differences between groups.
- Classification bias – Low: Drainage type and bile culture status were clearly defined and consistently documented at initial intervention
- Performance bias (deviations from intended interventions) – Low: Post-drainage and perioperative management protocols were consistently applied across groups.
- Missing data bias – Low: All 122 prospectively followed patients were included; no indication of loss to follow-up or missing data for reported outcomes.
- Detection bias (measurement of outcomes) – Moderate: Outcomes (bile infection, abscess formation, POPF) were measured prospectively but not blinded, and outcome assessment may have been influenced by knowledge of drainage type.
- Selective reporting bias – Low: All predefined key outcomes—including bile culture positivity, abscess risk, and POPF—were reported as specified in the study's objectives.
- Overall bias – Moderate risk

17. Tol, J.A.M.G.; van Hooft, J.E.; Timmer, R.; Kubben, F.J.G.M.; van der Harst, E.; de Hingh, I.H.J.T.; Vleggaar, F.P.; Molenaar, I.Q.; Keulemans, Y.C.A.; Boerma, D.; et al. Metal or Plastic Stents for Preoperative Biliary Drainage in Resectable Pancreatic Cancer. *Gut* **2016**, *65*, 1981–1987, doi:10.1136/gutjnl-2014-308762.
  - Confounding bias – Moderate: Despite being prospective, this is a cohort comparison (FCSEMS vs PS) not randomized; although baseline characteristics were reportedly similar, unmeasured confounders (e.g., operator preference, tumor stage) may have influenced outcomes.
  - Selection bias – Moderate: Patients received stents based on clinical and institutional protocol differences rather than randomized assignment, introducing potential bias in group selection.
  - Classification bias – Low: Stent type (FCSEMS vs PS) and patient inclusion category (plastic, metal, early surgery) were clearly defined and accurately recorded
  - Performance bias (deviations from intended interventions) – Low: Post-drainage and perioperative care appeared standardized across all cohorts, with no indications of differential management.
  - Missing data bias – Low: The study reported minimal attrition; all 53 FCSEMS and 102 PS patients were included in primary analyses, with no significant missing outcome data.
  - Detection bias (measurement of outcomes) – Moderate: Outcomes (PBD-related complications, surgical morbidity) were measured prospectively but likely without assessor blinding, which may introduce detection bias.

- Selective reporting bias – Low: All prespecified outcomes, including complication rates and stent exchanges, were fully reported
- Overall bias – Moderate risk

18. Cavell, L.K.; Allen, P.J.; Vinoya, C.; Eaton, A.A.; Gonen, M.; Gerdes, H.; Mendelsohn, R.B.; D'Angelica, M.I.; King-ham, T.P.; Fong, Y.; et al. Biliary Self-Expandable Metal Stents Do Not Adversely Affect Pancreaticoduodenecto-my. Am J Gastroenterol 2013, 108, 1168–1173, doi:10.1038/ajg.2013.93.
    - Confounding bias – Moderate: Being retrospective, baseline differences (e.g., comorbidities, bilirubin levels, tumor characteristics) between SEMS, plastic, and no-stent groups could have influenced outcomes.
    - Selection bias – Moderate: Biliary drainage type was determined by clinical practice, not random allocation, introducing selection bias.
    - Classification bias – Low: Interventions were clearly documented from surgical records (SEMS, plastic stent, or no stent).
    - Performance bias (deviations from intended interventions) – Low: Perioperative and postoperative care likely consistent across all groups.
    - Missing data bias – Low: The study utilized a prospectively maintained database with minimal missing data.
    - Detection bias (measurement of outcomes) – Moderate: Outcomes (e.g., operative time, wound infection) were collected retrospectively without blinding of assessors.
    - Selective reporting bias – Low: All key predefined outcomes including complication rates, margin status, and mortality were fully reported.
    - Overall bias – Moderate risk

19. Suenaga, M.; Yokoyama, Y.; Fujii, T.; Yamada, S.; Yamaguchi, J.; Hayashi, M.; Asahara, T.; Nagino, M.; Kodera, Y. Impact of Qualitative and Quantitative Biliary Contamination Status on the Incidence of Postoperative Infection Complications in Patients Undergoing Pancreatoduodenectomy. Ann Surg Oncol 2021, 28, 560–569, doi:10.1245/s10434-020-08645-w.
    - Confounding bias – Moderate: As a non-randomized cohort comparing external drainage (ED), internal drainage (ID), and no drainage (ND), baseline factors (e.g., microbiome status, patient health) could affect infection risk.
    - Selection bias – Moderate: Patients were assigned to drainage groups based on clinical decisions, not by protocol.
    - Classification bias – Low: Biliary drainage status and quantitative contamination were clearly defined and measured with RT-qPCR.
    - Performance bias (deviations from intended interventions) – Low: Uniform perioperative protocols were applied; no evidence of differential treatment.
    - Missing data bias – Low: Prospective collection across 152 patients with comprehensive bile cultures and follow-up data.
    - Detection bias (measurement of outcomes) – Moderate: Infection outcomes were assessed from clinical surveillance and records without blinding—it's likely assessors knew drainage status.
    - Selective reporting bias – Low: Key outcomes—postoperative infection rates and bile microbial load—were reported as prespecified.

- Overall bias – Moderate risk

20. Uemura, K.; Murakami, Y.; Satoi, S.; Sho, M.; Motoi, F.; Kawai, M.; Matsumoto, I.; Honda, G.; Kurata, M.; Yanag-imoto, H.; et al. Impact of Preoperative Biliary Drainage on Long-Term Survival in Resected Pancreatic Ductal Adenocarcinoma: A Multicenter Observational Study. Ann Surg Oncol 2015, 22 Suppl 3, S1238-46, doi:10.1245/s10434-015-4618-9.
    - Confounding bias – Moderate: Though multivariate analysis adjusted for known prognostic factors, residual confounders—such as center-specific practices or unmeasured patient comorbidities—may still influence survival outcomes.
    - Selection bias – Moderate: Preoperative biliary drainage (PBD) choice—none, ERBD, or PTBD—was based on clinical judgment and institutional practice, not randomized, potentially introducing selection bias.
    - Classification bias – Low: PBD status was clearly defined in the database and reliably recorded (ERBD, PTBD, or none).
    - Performance bias (deviations from intended interventions) – Low: Post-drainage surgical and perioperative management was standard across participating high-volume centers.
    - Missing data bias – Low: Large multicenter cohort (n=932) with minimal missing data; PBD type and survival outcomes were readily available.
    - Detection bias (measurement of outcomes) – Moderate: Survival and recurrence data were collected retrospectively without assessor blinding, which could influence reporting.
    - Selective reporting bias – Low: All prespecified outcomes—including overall survival and recurrence patterns—were reported according to study objectives.
    - Overall bias – Moderate risk

21. Zhang, G.-Q.; Li, Y.; Ren, Y.-P.; Fu, N.-T.; Chen, H.-B.; Yang, J.-W.; Xiao, W.-D. Outcomes of Preoperative Endo-scopic Nasobiliary Drainage and Endoscopic Retrograde Biliary Drainage for Malignant Distal Biliary Obstruc-tion Prior to Pancreaticoduodenectomy. World J Gastroenterol 2017, 23, 5386–5394, doi:10.3748/wjg.v23.i29.5386.

    - Confounding bias – Moderate: Non-random assignment to ENBD or ERBD may correlate with unmeasured clinical factors (e.g., bilirubin levels, comorbidities), affecting postoperative outcomes.
    - Selection bias – Moderate: Choice of drainage method was based on clinician preference or institutional practice rather than standardized criteria.
    - Classification bias – Low: Drainage type (ENBD vs ERBD) was clearly defined and recorded in medical records.
    - Performance bias (deviations from intended interventions) – Low: Post-drainage care and surgical protocols were applied consistently across groups.
    - Missing data bias – Low: The study reported complete data for 184 patients with no indication of missing outcome information.
    - Detection bias (measurement of outcomes) – Moderate: Outcomes such as complications and drainage-related events were assessed retrospectively without assessor blinding.

- Selective reporting bias – Low: All prespecified outcomes (e.g., cholangitis, POPF, length of stay, hospital costs) were reported in line with study objectives.
- Overall bias – Moderate risk

22. Kuwatani, M.; Nakamura, T.; Hayashi, T.; Kimura, Y.; Ono, M.; Motoya, M.; Imai, K.; Yamakita, K.; Goto, T.; Takahashi, K.; et al. Clinical Outcomes of Biliary Drainage during a Neoadjuvant Therapy for Pancreatic Cancer: Metal versus Plastic Stents. Gut Liver 2020, 14, 269–273, doi:10.5009/gnl18573.
    - Confounding bias – Moderate - Patient assignment to metal (MS) vs plastic (PS) stents was not randomized; baseline characteristics such as disease severity or comorbidities may have differed and influenced outcomes.
    - Selection bias – Moderate - Stent type was chosen based on clinical discretion rather than allocation protocol, introducing potential bias in group composition.
    - Classification bias – Low - Stent type and outcomes (e.g., recurrent biliary obstruction, therapy delay, operative details) were clearly defined and reliably recorded
    - Performance bias (deviations from intended interventions) – Low - Peri-neoadjuvant care and surgical protocols were likely consistent across both groups without systematic differences.
    - Missing data bias – Low - Complete data were available for all 29 patients included in the analysis, with no indication of loss to follow-up.
    - Detection bias (measurement of outcomes) – Moderate
    - Outcomes such as biliary obstruction and delays were assessed retrospectively without blinding of outcome adjudicators, potentially introducing measurement bias.
    - Selective reporting bias – Low - All predefined outcomes—including recurrent obstruction, neoadjuvant therapy delays, operative time, blood loss, and complications—were transparently reported
    - Overall bias – Moderate risk
