# Supplementary material for: A Systematic Review and Meta-Analysis of Preoperative Biliary Drainage Methods in Periampullary Tumors
Source: J Clin Med. 2025 Oct 8;14(19):7097. doi: 10.3390/jcm14197097 (PMC12524691; doi:10.3390/jcm14197097)
Supplement: Supplementary file 1 [file jcm-14-07097-s001.zip › Supplementary material 3-Mean and standard deviation.pdf]

Microsoft Excel formulas used for estimating the sample mean and standard deviation from reported medians and dispersion measures are depicted below:

- For mean estimation: Mean ≈ (a + 2m + b) / 4, where a = minimum, m = median and b = maximum
- For standard deviation estimation:
    a) When only range is available: SD ≈ (b – a) / 4, where a = minimum and b = maximum
    b) When the interquartile range (IQR) is available: SD ≈ IQR /1.35, where IQR = Q3 – Q1
