# Supplementary material for: A Systematic Review and Meta-Analysis of Preoperative Biliary Drainage Methods in Periampullary Tumors
Source: J Clin Med. 2025 Oct 8;14(19):7097. doi: 10.3390/jcm14197097 (PMC12524691; doi:10.3390/jcm14197097)
Supplement: Supplementary file 1 [file jcm-14-07097-s001.zip › Supplementary material 5-Qualitative variables - RR with 95%CI.pdf]

Contents

Selectie: ERBD - ENBD/PTBD 2

Meta-analysis for Pancreatitis treatment, comparing ERBD with ENBD/PTBD 2

Meta-analysis for Cholangitis treatment, comparing ERBD with ENBD/PTBD 5

Meta-analysis for Perforation treatment, comparing ERBD with ENBD/PTBD 8

Meta-analysis for Hemorrhage treatment, comparing ERBD with ENBD/PTBD 8

Meta-analysis for Occlusion treatment, comparing ERBD with ENBD/PTBD 11

Meta-analysis for Catheter exchange treatment, comparing ERBD with ENBD/PTBD 14

Meta-analysis for Mortality treatment, comparing ERBD with ENBD/PTBD 17

Meta-analysis for Reoperation treatment, comparing ERBD with ENBD/PTBD 19

Meta-analysis for Overall complication rate (Clavien - Dindo) treatment, comparing ERBD with ENBD/PTBD 22

Meta-analysis for Grade 1-2 treatment, comparing ERBD with ENBD/PTBD 25

Meta-analysis for Grade >=3 treatment, comparing ERBD with ENBD/PTBD 28

Meta-analysis for Infectious complications treatment, comparing ERBD with ENBD/PTBD 31

Meta-analysis for Sepsis treatment, comparing ERBD with ENBD/PTBD 34

Meta-analysis for Intraabdominal abcess treatment, comparing ERBD with ENBD/PTBD . 37

Meta-analysis for Wound infections treatment, comparing ERBD with ENBD/PTBD 40

Meta-analysis for PPH treatment, comparing ERBD with ENBD/PTBD 43

Meta-analysis for Chyle leak treatment, comparing ERBD with ENBD/PTBD 46

Meta-analysis for POBF treatment, comparing ERBD with ENBD/PTBD 46

Meta-analysis for POPF treatment, comparing ERBD with ENBD/PTBD 49

Meta-analysis for Grade 1 treatment, comparing ERBD with ENBD/PTBD 52

Meta-analysis for Grade 2-3 treatment, comparing ERBD with ENBD/PTBD 55

Meta-analysis for DGE treatment, comparing ERBD with ENBD/PTBD 58

Meta-analysis for Soft pancreas treatment, comparing ERBD with ENBD/PTBD 61

Sinteza metaanalizelor 64

Selectie: PS - SEMS 84

Meta-analysis for Pancreatitis treatment, comparing PS with SEMS 84

Meta-analysis for Cholangitis treatment, comparing PS with SEMS 87

Meta-analysis for Perforation treatment, comparing PS with SEMS 90

Meta-analysis for Hemorrhage treatment, comparing PS with SEMS 93

Meta-analysis for Occlusion treatment, comparing PS with SEMS 96

Meta-analysis for Catheter exchange treatment, comparing PS with SEMS 99

Meta-analysis for Mortality treatment, comparing PS with SEMS 102

Meta-analysis for Reoperation treatment, comparing PS with SEMS 105

Meta-analysis for Overall complication rate (Clavien - Dindo) treatment, comparing PS with SEMS 108

Meta-analysis for Grade 1-2 treatment, comparing PS with SEMS 111

Meta-analysis for Grade >=3 treatment, comparing PS with SEMS 114

Meta-analysis for Infectious complications treatment, comparing PS with SEMS 117

Meta-analysis for Sepsis treatment, comparing PS with SEMS 119

Meta-analysis for Intraabdominal abcess treatment, comparing PS with SEMS 121

Meta-analysis for Wound infections treatment, comparing PS with SEMS 124

Meta-analysis for PPH treatment, comparing PS with SEMS 127

Meta-analysis for Chyle leak treatment, comparing PS with SEMS 130

Meta-analysis for POBF treatment, comparing PS with SEMS 132

Meta-analysis for POPF treatment, comparing PS with SEMS 135

Meta-analysis for Grade 1 treatment, comparing PS with SEMS 138

Meta-analysis for Grade 2-3 treatment, comparing PS with SEMS 140

Meta-analysis for DGE treatment, comparing PS with SEMS 143

Meta-analysis for Soft pancreas treatment, comparing PS with SEMS 146

Sinteza metaanalizelor 148

Selectie: ERBD - ENBD/PTBD

Meta-analysis for Pancreatitis treatment, comparing ERBD with ENBD/PTBD

	datele.Year	datele.Study.name	numberCasesPerTreatment	numberCasesTreatment	numberCasesPerControl	numberCasesControl
1	2011	Park, 2011	17	34	7	43
5	2015	Huang, 2015	1	37	1	63
6	2017	Zhang, 2017	12	51	12	102
12	2021	El-Haddad, 2021	1	34	0	30
13	2021	Suenaga, 2021	3	40	3	38

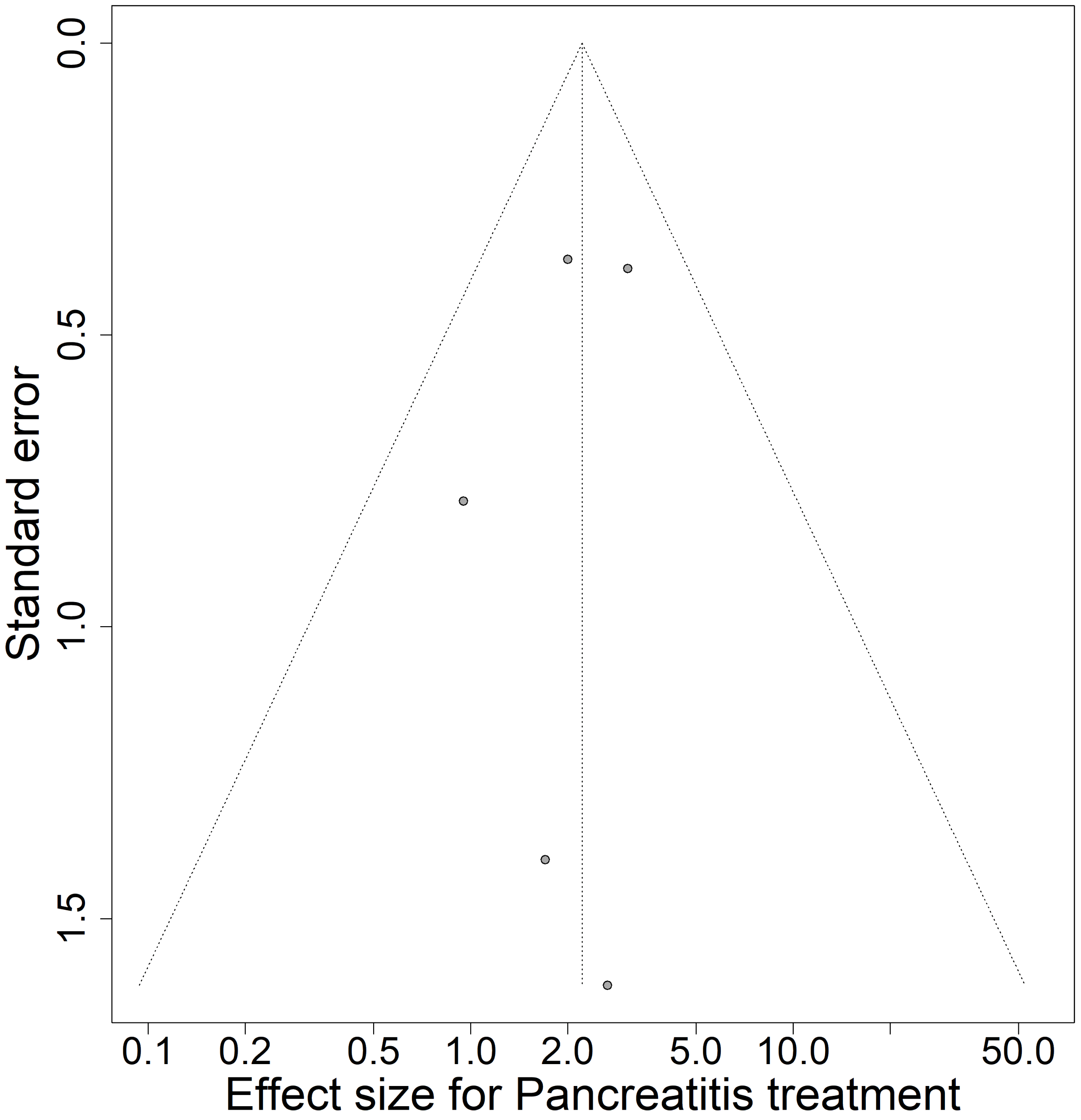

Fig. Funnel plot for Pancreatitis treatment, comparing ERBD with ENBD/PTBD

The funnel plot for Pancreatitis treatment, comparing ERBD with ENBD/PTBD is shown in figure _.

The publication bias test gave a p=0.533.

Influence studies: Omitting Park, 2011; Omitting Huang, 2015; Omitting Zhang, 2017; Omitting El-Haddad, 2021; Omitting Suenaga, 2021 - yes; no; no; no; no

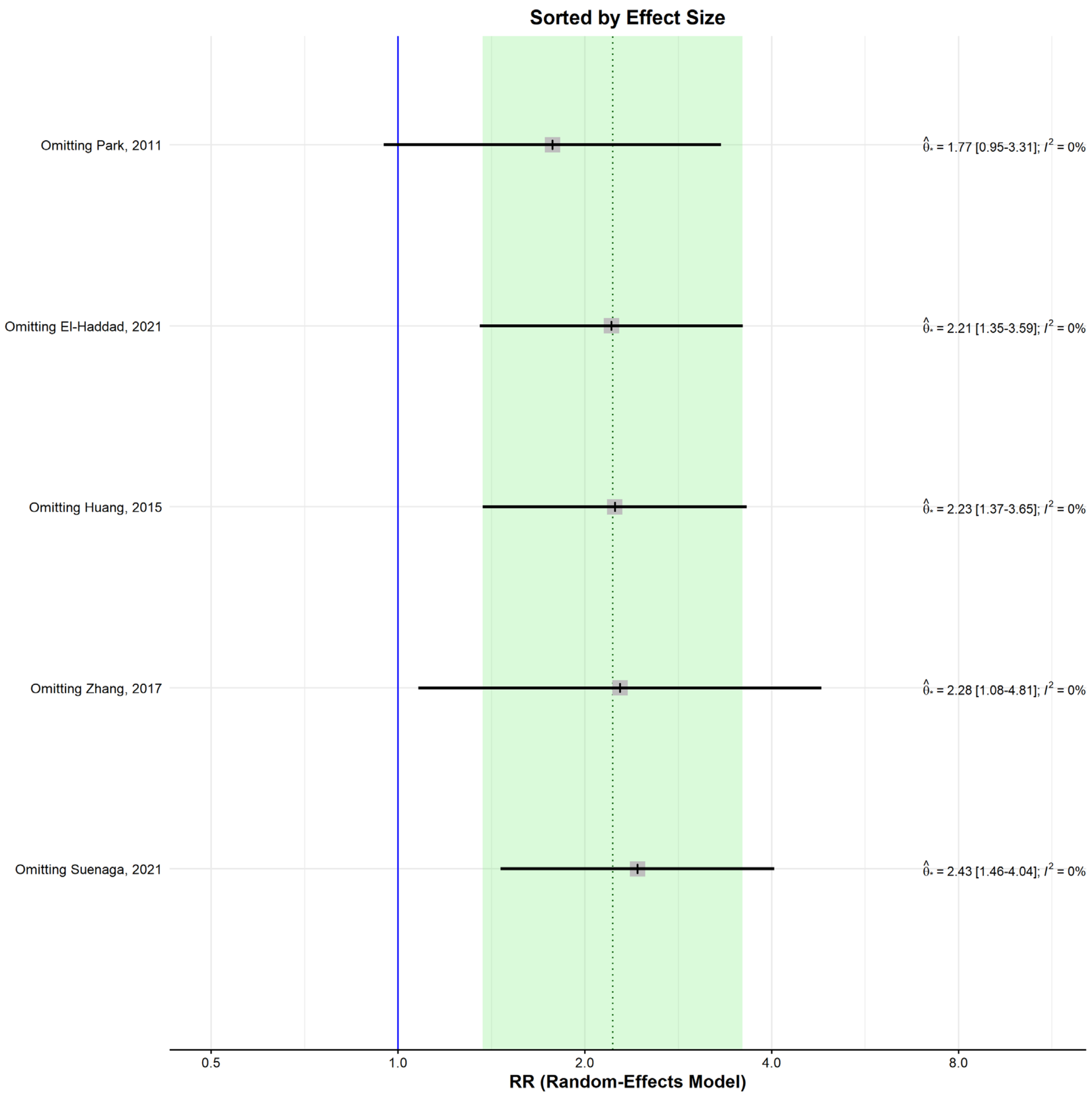

Fig. Leave-one-out sensitivity analysis plot for selected studies for Pancreatitis treatment

The heterogeneity was assessed, and we found an I2 of 0% (95% CI 0% - 79.2%) and the Q test for heterogeneity gave p=0.735.

The RR value (the RR of Pancreatitis treatment in the ERBD group compared to the ENBD/PTBD group) obtained with the meta-analysis was of 0.8 (95% CI 0.31 - 1.28), p=0.001 using the model with random effects.

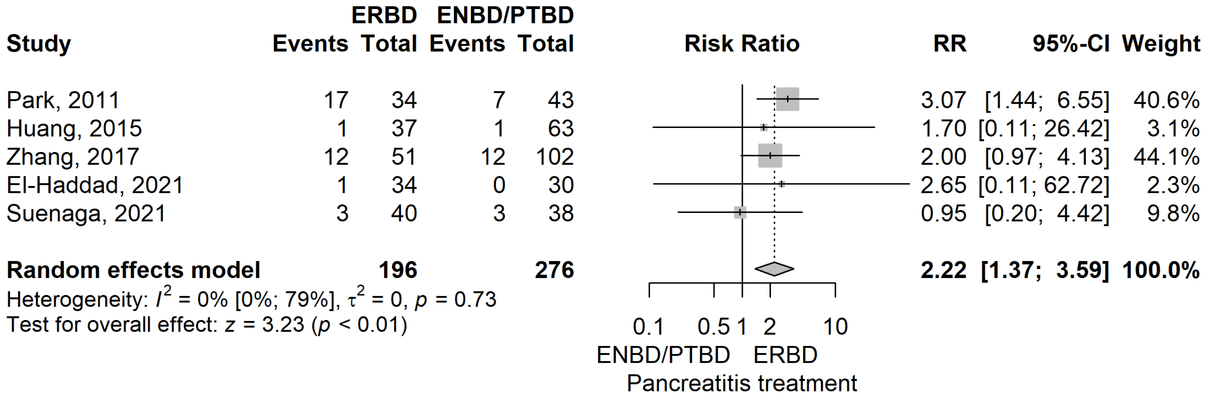

Fig. Forest plot for Pancreatitis treatment, comparing ERBD with ENBD/PTBD

Meta-analysis for Cholangitis treatment, comparing ERBD with ENBD/PTBD

	datele.Year	datele.Study.name	numberCasesPerTreatment	numberCasesTreatment	numberCasesPerControl	numberCasesControl
2	2014	Kitahata, 2014	15	67	1	60
3	2015	Fujii, 2015	22	72	6	50
5	2015	Huang, 2015	8	37	9	63
6	2017	Zhang, 2017	12	51	8	102
12	2021	El-Haddad, 2021	2	34	3	30
13	2021	Suenaga, 2021	7	40	12	38
14	2022	Satoh, 2022	53	117	19	39

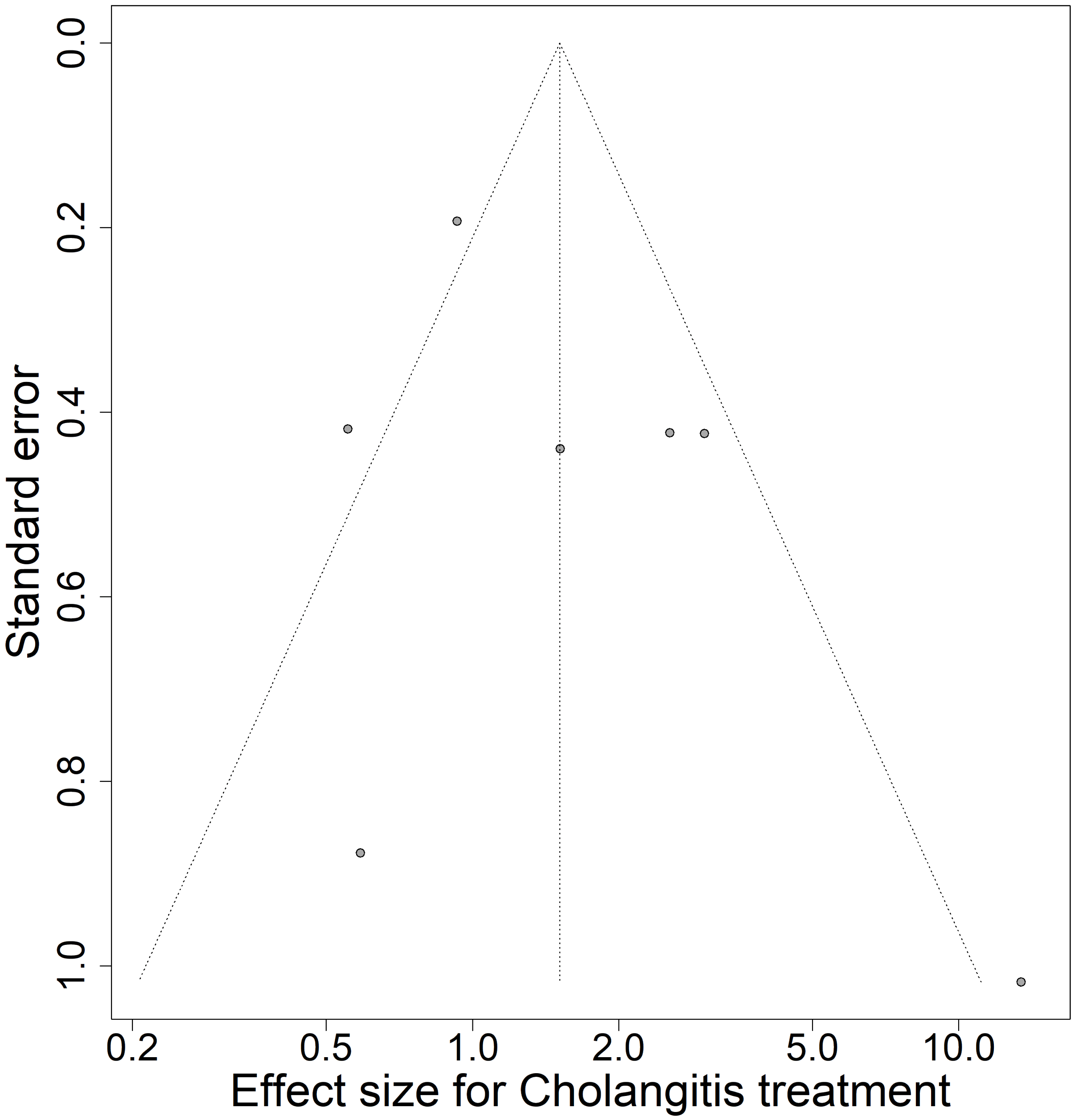

Fig. Funnel plot for Cholangitis treatment, comparing ERBD with ENBD/PTBD

The funnel plot for Cholangitis treatment, comparing ERBD with ENBD/PTBD is shown in figure _.

The publication bias test gave a p=0.276.

Influence studies: Omitting Kitahata, 2014; Omitting Fujii, 2015; Omitting Huang, 2015; Omitting Zhang, 2017; Omitting El-Haddad, 2021; Omitting Suenaga, 2021; Omitting Satoh, 2022 - no; no; no; no; no; no; no

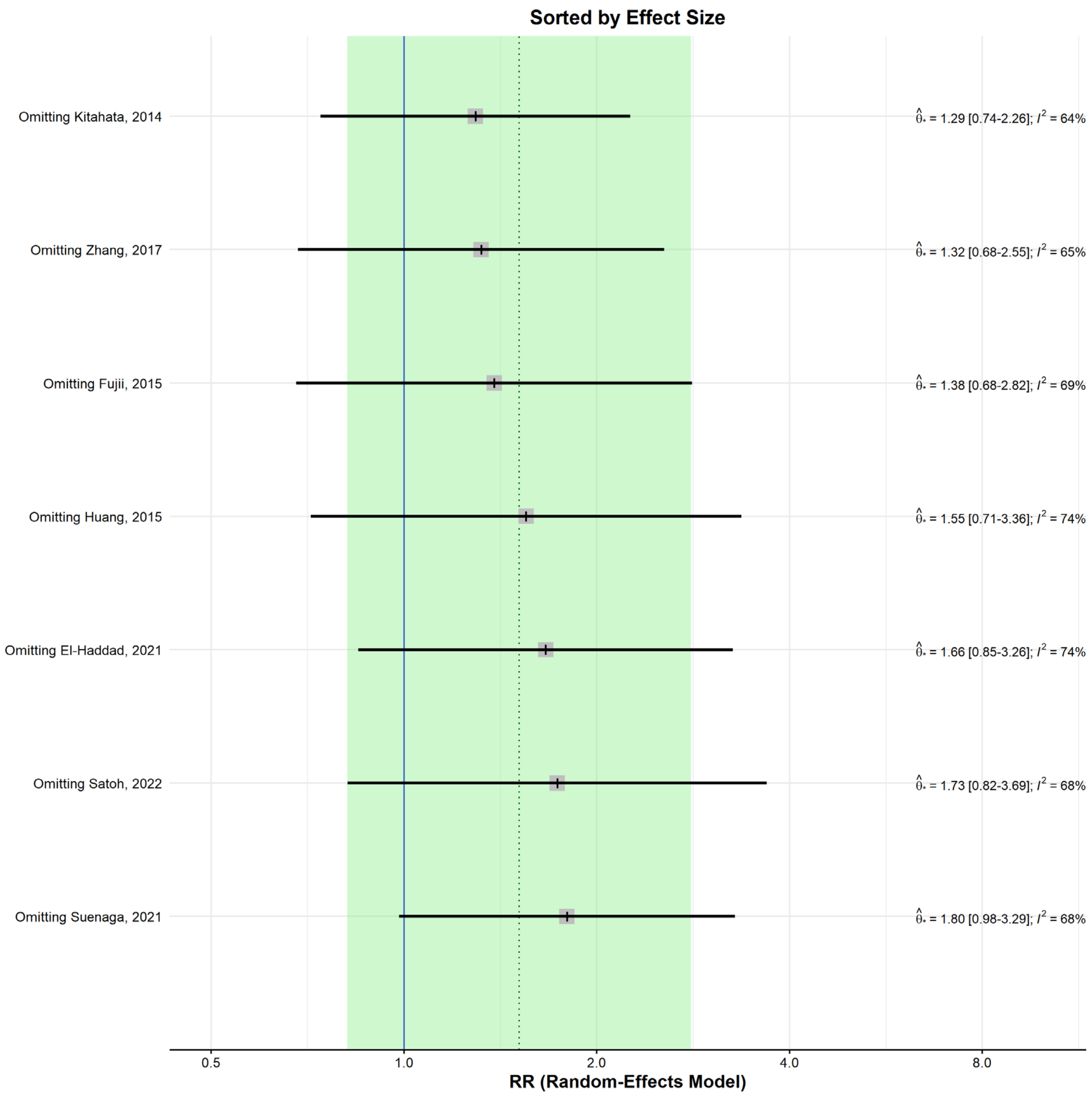

Fig. Leave-one-out sensitivity analysis plot for selected studies for Cholangitis treatment

The heterogeneity was assessed, and we found an I2 of 69.4% (95% CI 32.6% - 86.1%) and the Q test for heterogeneity gave p=0.003.

The RR value (the RR of Cholangitis treatment in the ERBD group compared to the ENBD/PTBD group) obtained with the meta-analysis was of 0.41 (95% CI -0.2 - 1.03), p=0.19 using the model with random effects.

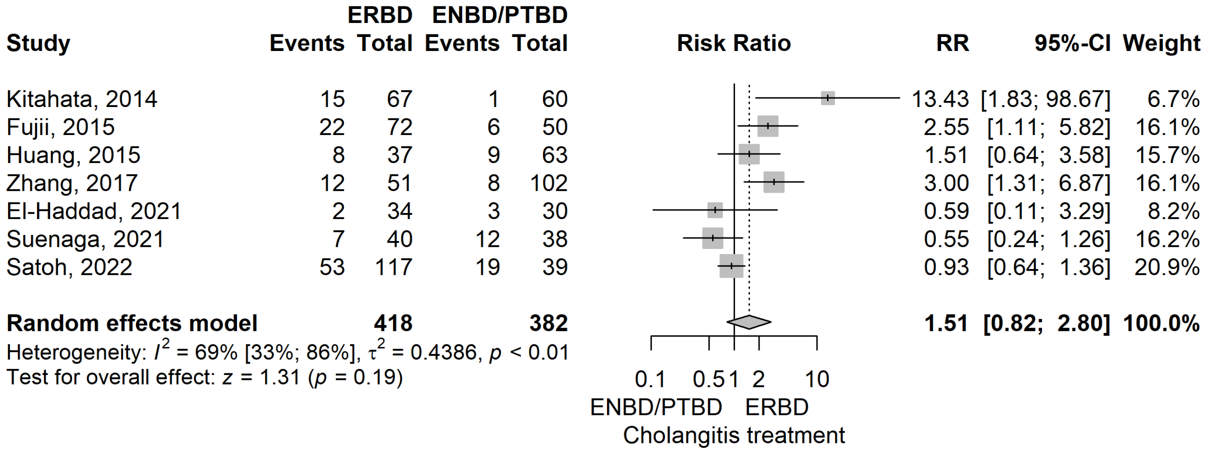

Fig. Forest plot for Cholangitis treatment, comparing ERBD with ENBD/PTBD

Meta-analysis for Perforation treatment, comparing ERBD with ENBD/PTBD

No studies to combine for this characteristic

Meta-analysis for Hemorrhage treatment, comparing ERBD with ENBD/PTBD

	datele.Year	datele.Study.name	numberCasesPerTreatment	numberCasesTreatment	numberCasesPerControl	numberCasesControl
1	2011	Park, 2011	0	34	1	43
12	2021	El-Haddad, 2021	1	34	0	30
2						

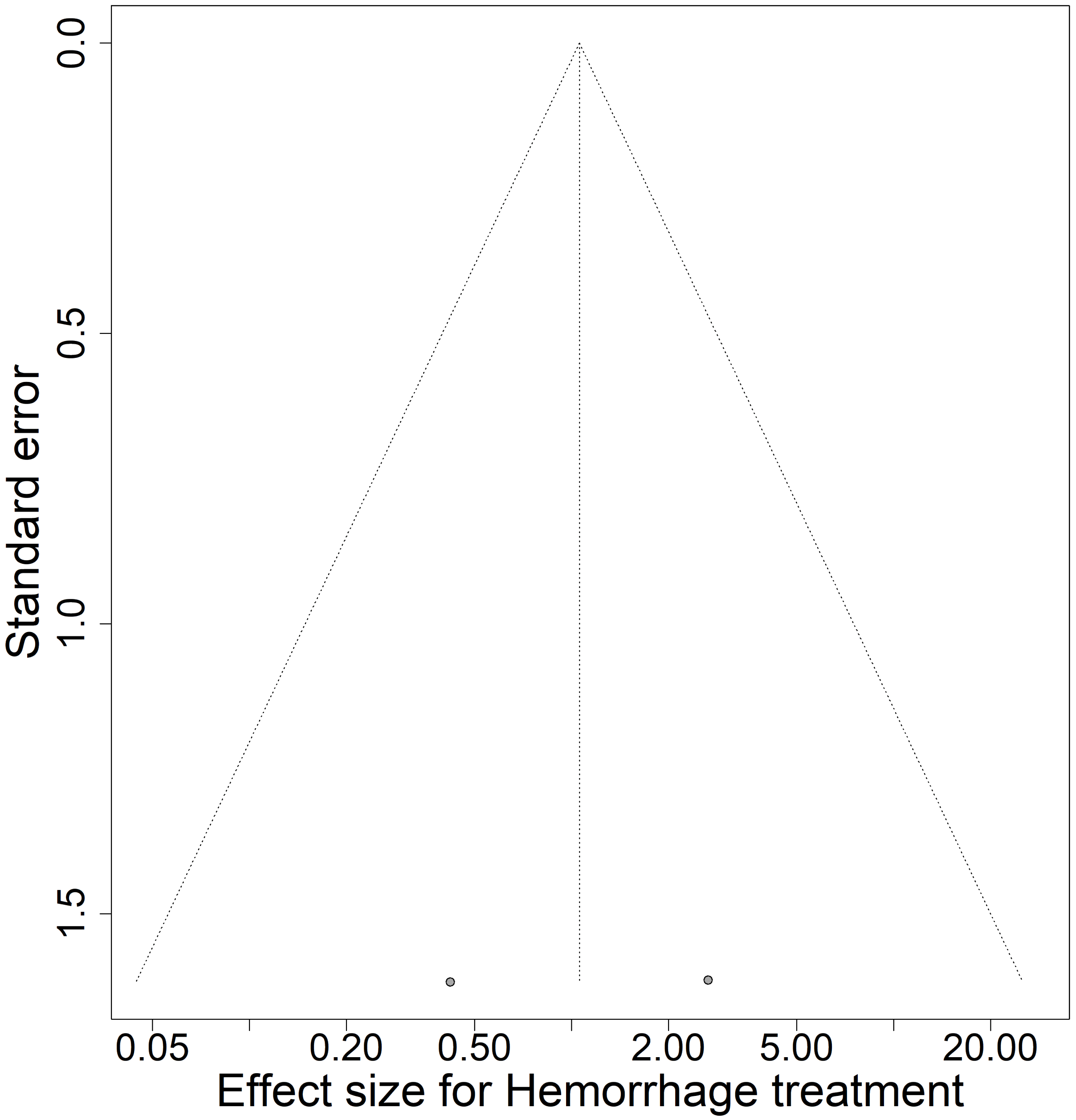

Fig. Funnel plot for Hemorrhage treatment, comparing ERBD with ENBD/PTBD

The funnel plot for Hemorrhage treatment, comparing ERBD with ENBD/PTBD is shown in figure _.

The publication bias test cannot be computed since there are not at least three studies.

Influence studies: Omitting Park, 2011; Omitting El-Haddad, 2021 - yes; yes

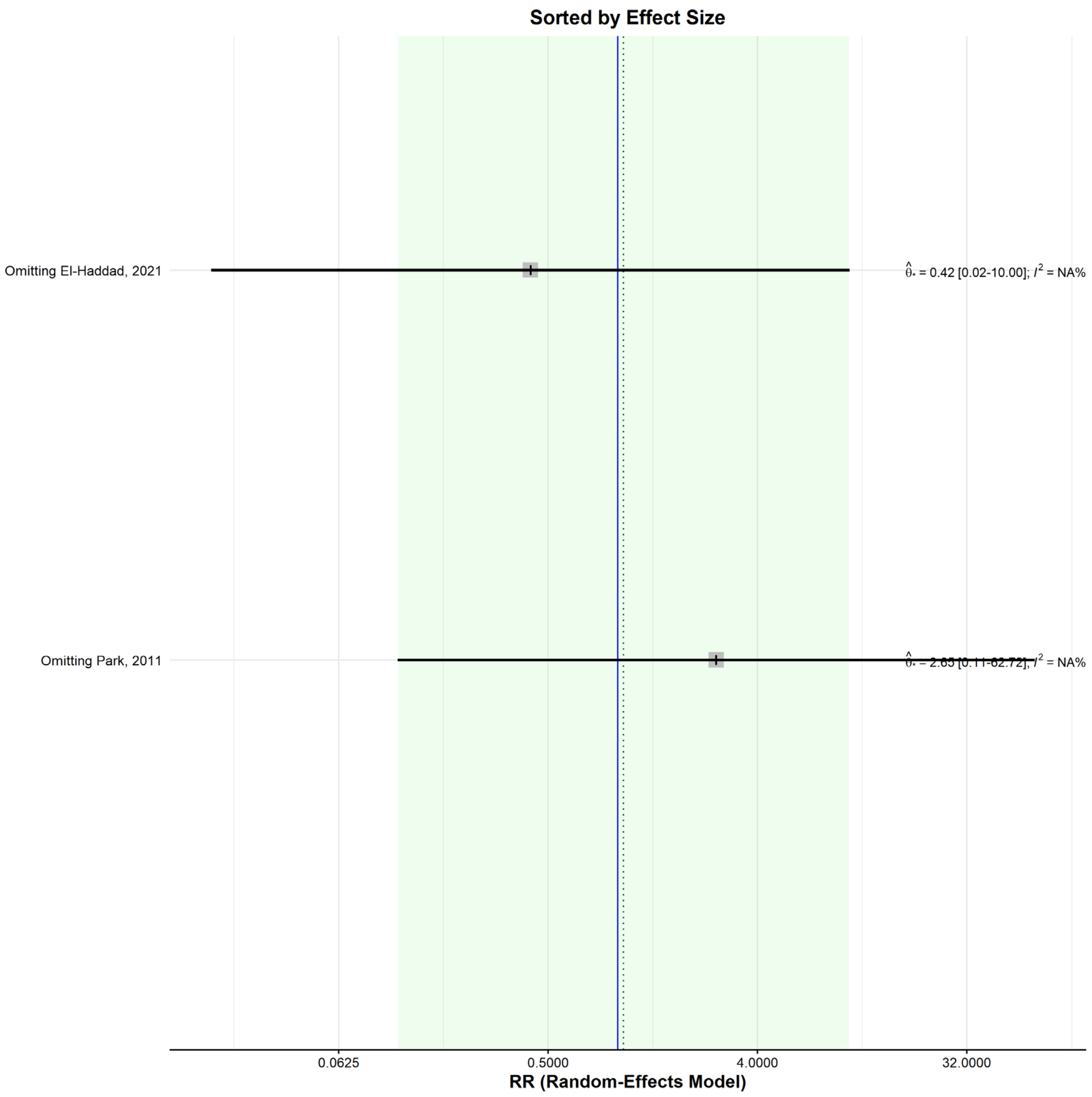

Fig. Leave-one-out sensitivity analysis plot for selected studies for Hemorrhage treatment

The heterogeneity was assessed, and we found an I2 of 0% (95% CI NA% - NA%) and the Q test for heterogeneity gave p=0.42.

The RR value (the RR of Hemorrhage treatment in the ERBD group compared to the ENBD/PTBD group) obtained with the meta-analysis was of 0.06 (95% CI -2.18 - 2.29), p=0.961 using the model with random effects.

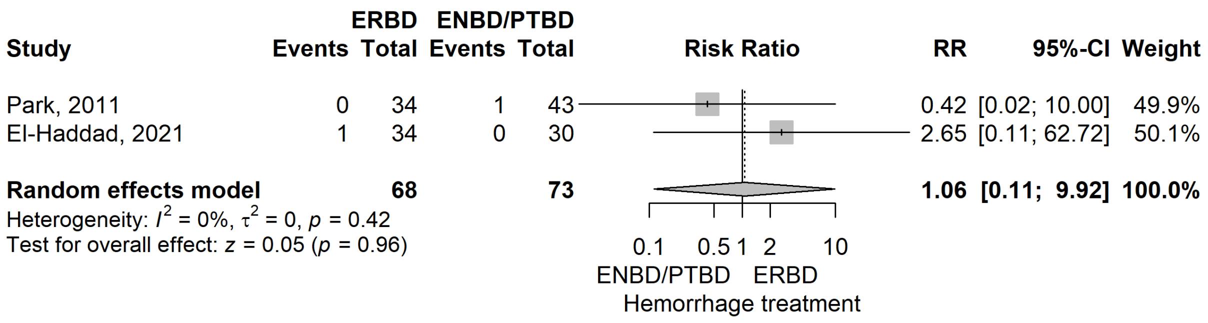

Fig. Forest plot for Hemorrhage treatment, comparing ERBD with ENBD/PTBD

Meta-analysis for Occlusion treatment, comparing ERBD with ENBD/PTBD

	datele.Year	datele.Study.name	numberCasesPerTreatment	numberCasesTreatment	numberCasesPerControl	numberCasesControl
1	2011	Park, 2011	6	34	0	43
5	2015	Huang, 2015	2	37	1	63
6	2017	Zhang, 2017	5	51	16	102

2

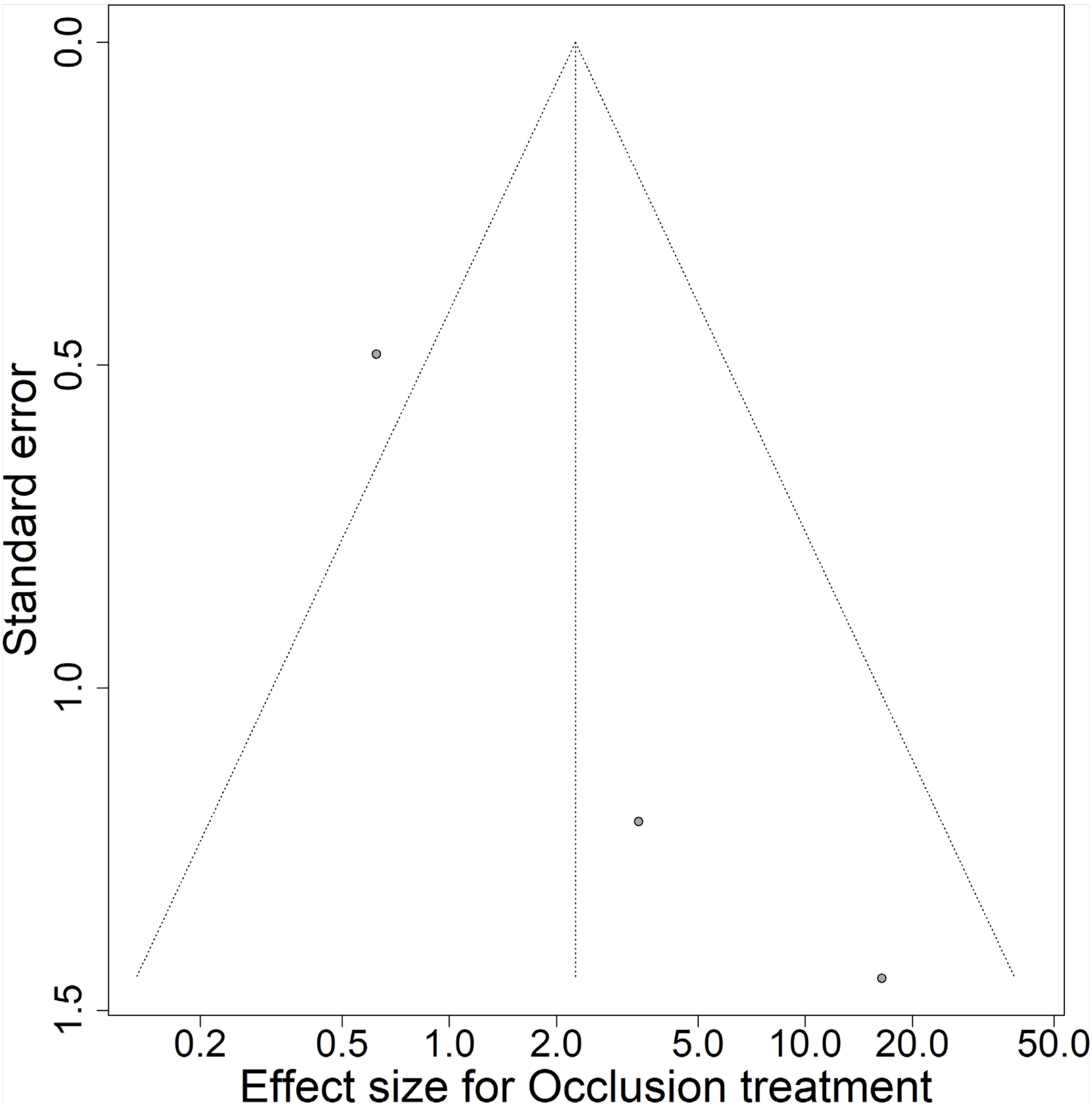

Fig. Funnel plot for Occlusion treatment, comparing ERBD with ENBD/PTBD

The funnel plot for Occlusion treatment, comparing ERBD with ENBD/PTBD is shown in figure _.

The publication bias test gave a p=0.124.

Influence studies: Omitting Park, 2011; Omitting Huang, 2015; Omitting Zhang, 2017 - yes; no; yes

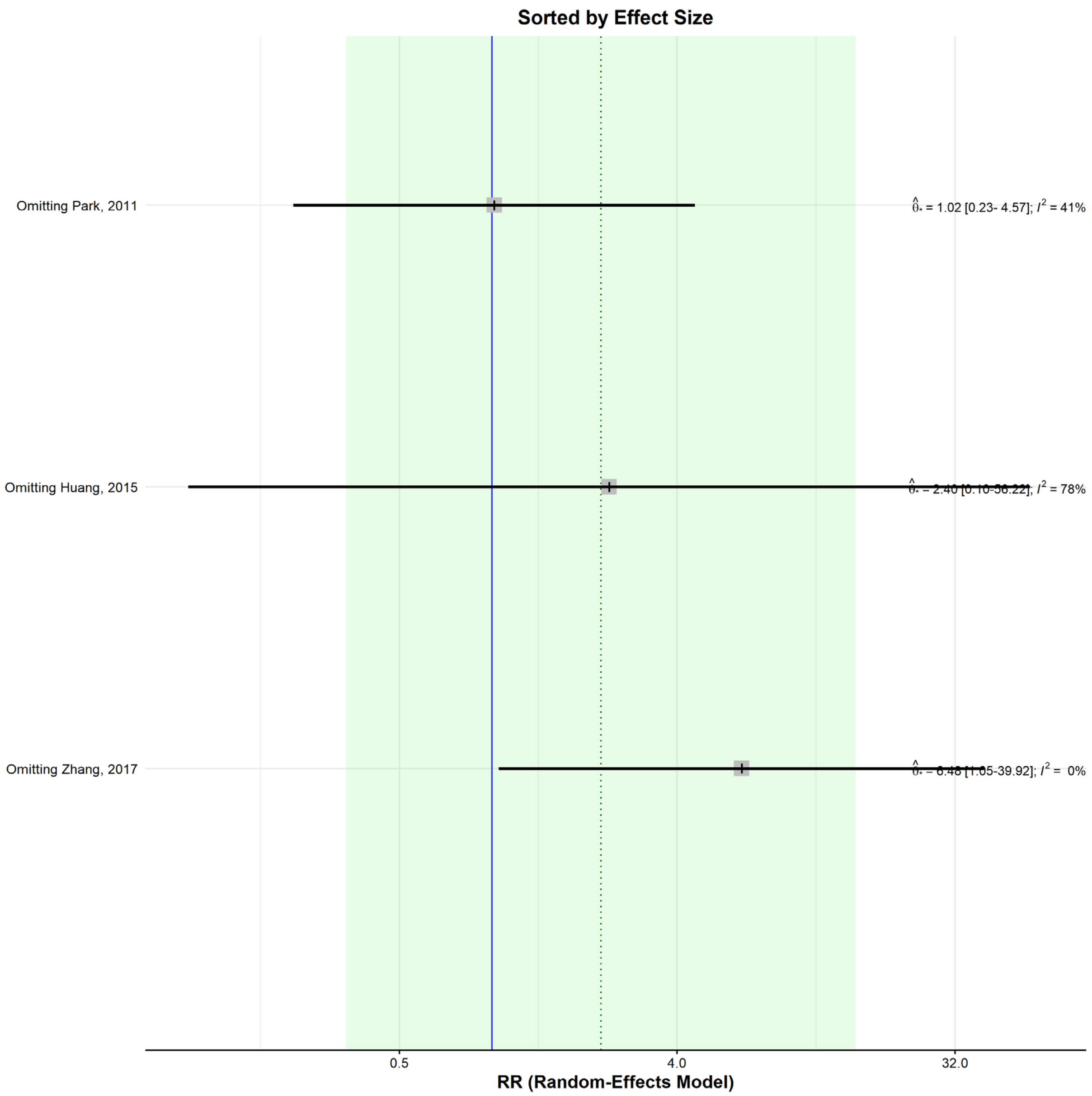

Fig. Leave-one-out sensitivity analysis plot for selected studies for Occlusion treatment

The heterogeneity was assessed, and we found an I2 of 64.9% (95% CI 0% - 89.9%) and the Q test for heterogeneity gave p=0.058.

The RR value (the RR of Occlusion treatment in the ERBD group compared to the ENBD/PTBD group) obtained with the meta-analysis was of 0.82 (95% CI -1.09 - 2.72), p=0.401 using the model with random effects.

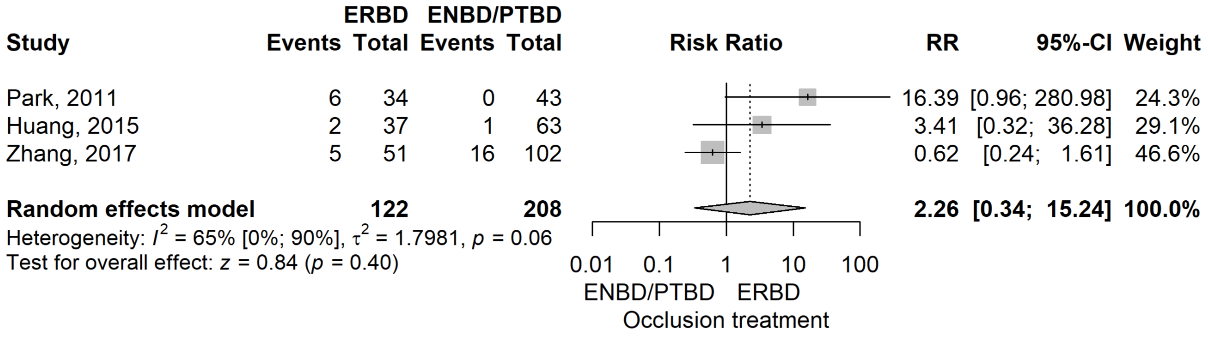

Fig. Forest plot for Occlusion treatment, comparing ERBD with ENBD/PTBD

Meta-analysis for Catheter exchange treatment, comparing ERBD with ENBD/PTBD

	datele.Year	datele.Study.name	numberCasesPerTreatment	numberCasesTreatment	numberCasesPerControl	numberCasesControl
1	2011	Park, 2011	10	34	0	43
13	2021	Suenaga, 2021	17	40	23	38
14	2022	Satoh, 2022	54	117	21	39
			2			

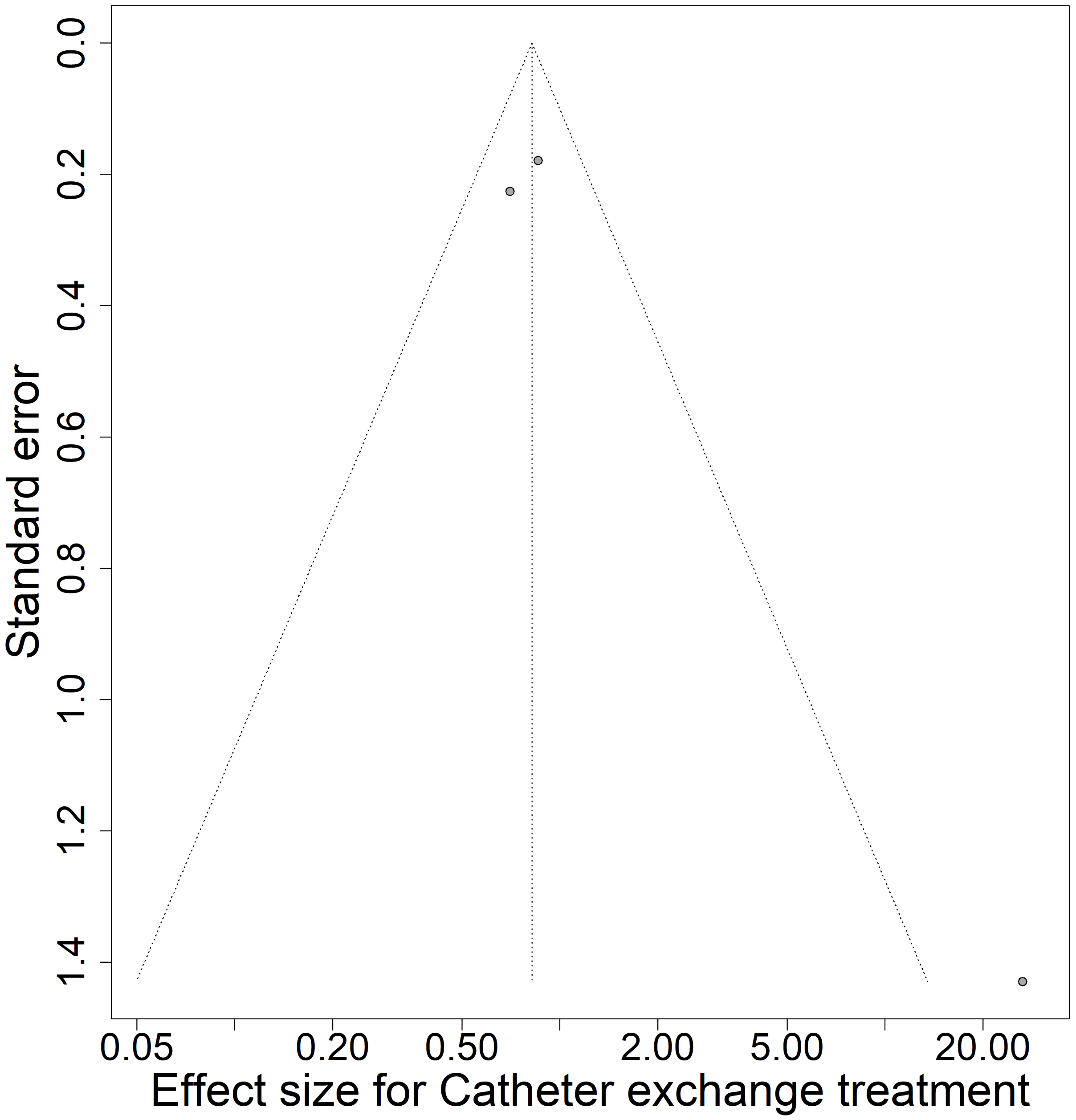

Fig. Funnel plot for Catheter exchange treatment, comparing ERBD with ENBD/PTBD

The funnel plot for Catheter exchange treatment, comparing ERBD with ENBD/PTBD is shown in figure _.

The publication bias test gave a p=0.296.

Influence studies: Omitting Park, 2011; Omitting Suenaga, 2021; Omitting Satoh, 2022 - no; yes; yes

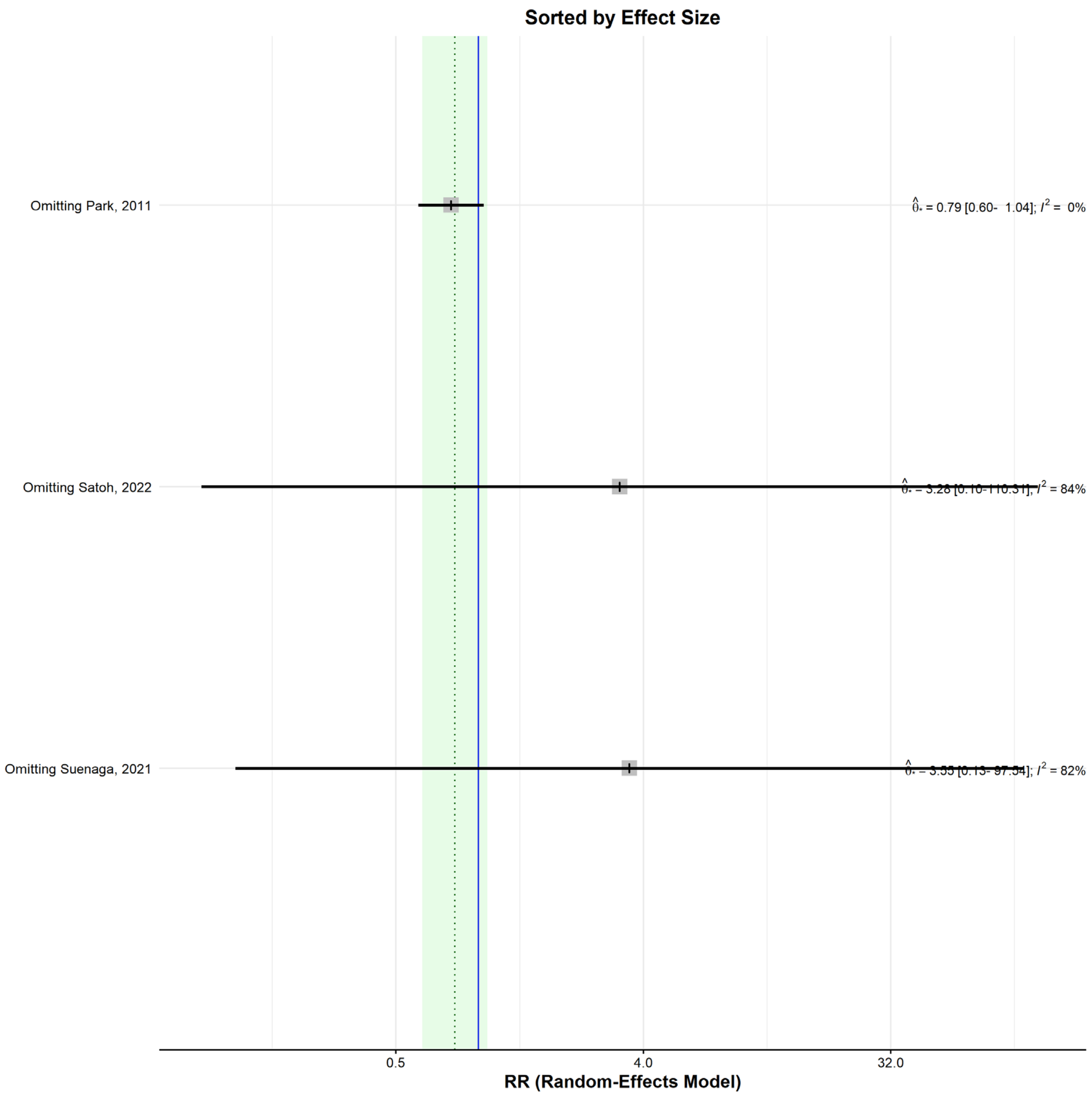

Fig. Leave-one-out sensitivity analysis plot for selected studies for Catheter exchange treatment

The heterogeneity was assessed, and we found an I2 of 69% (95% CI 0% - 91%) and the Q test for heterogeneity gave p=0.04.

The RR value (the RR of Catheter exchange treatment in the ERBD group compared to the ENBD/PTBD group) obtained with the meta-analysis was of -0.2 (95% CI -0.47 - 0.08), p=0.157 using the model with random effects.

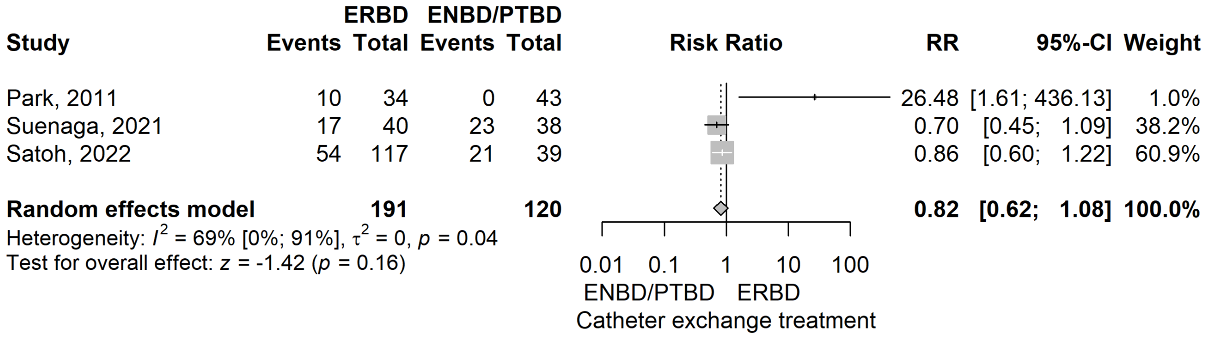

Fig. Forest plot for Catheter exchange treatment, comparing ERBD with ENBD/PTBD

Meta-analysis for Mortality treatment, comparing ERBD with ENBD/PTBD

	datele.Year	datele.Study.name	numberCasesPerTreatment	numberCasesTreatment	numberCasesPerControl	numberCasesControl
1	2011	Park, 2011	1	34	4	43
2	2014	Kitahata, 2014	3	67	1	60
3	2015	Fujii, 2015	0	72	0	50
4	2015	Uemura, 2015	12	407	2	166
5	2015	Huang, 2015	2	37	0	63
6	2017	Zhang, 2017	3	51	5	102
9	2019	Okano, 2019	15	1170	27	772
10	2021	Han, 2021	3	42	1	46
11	2021	Byun, 2021	1	106	2	61
12	2021	El-Haddad, 2021	3	34	1	30
15	2022	Subasi, 2022	2	42	3	33

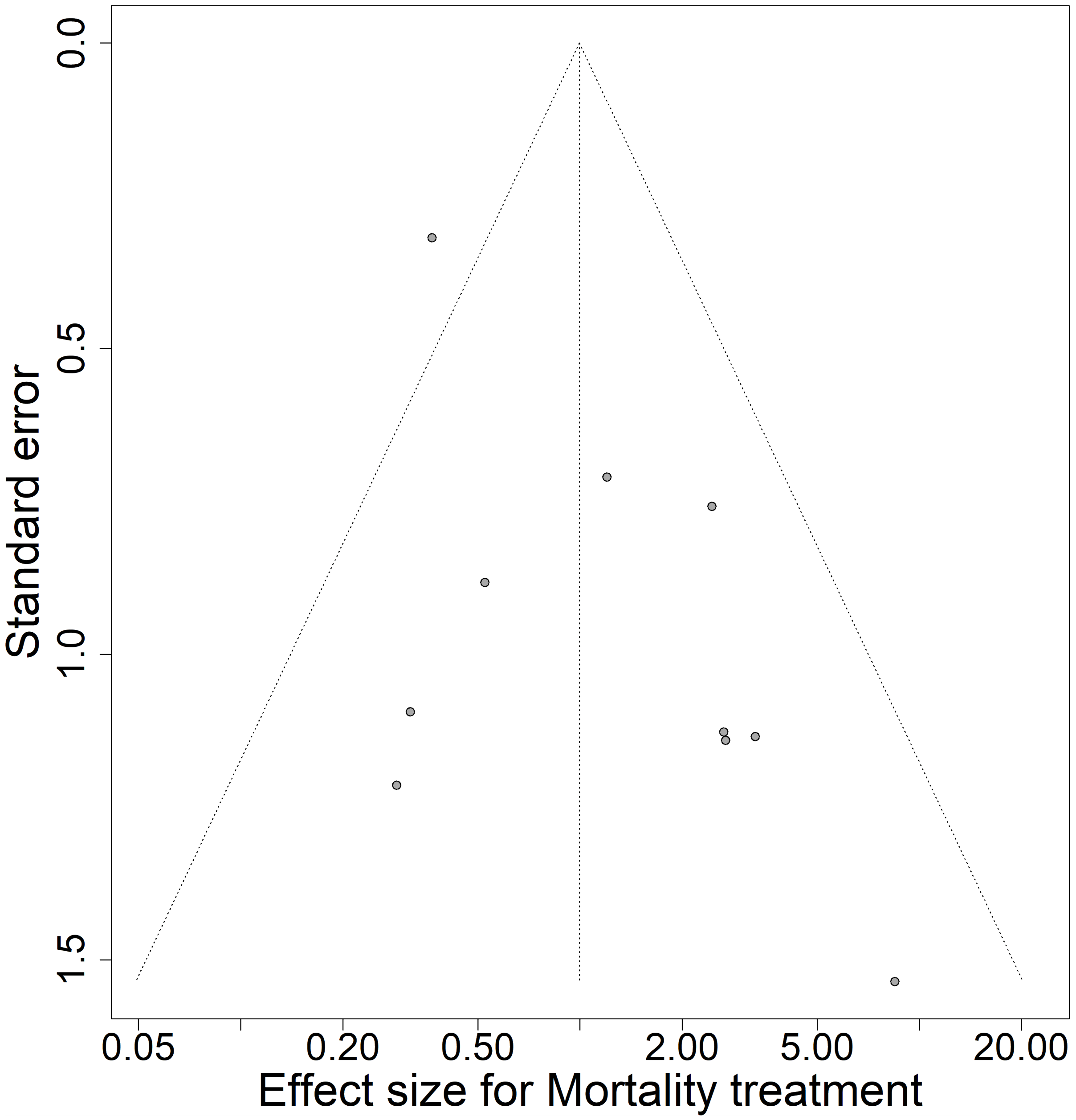

Fig. Funnel plot for Mortality treatment, comparing ERBD with ENBD/PTBD

The funnel plot for Mortality treatment, comparing ERBD with ENBD/PTBD is shown in figure _.

The publication bias test gave a p=0.031.

Influence analysis plus chart - Algorithm did not converge

The heterogeneity was assessed, and we found an I2 of 43.2% (95% CI 0% - 72.8%) and the Q test for heterogeneity gave p=0.07.

The RR value (the RR of Mortality treatment in the ERBD group compared to the ENBD/PTBD group) obtained with the meta-analysis was of 0 (95% CI -0.71 - 0.7), p=0.99 using the model with random effects.

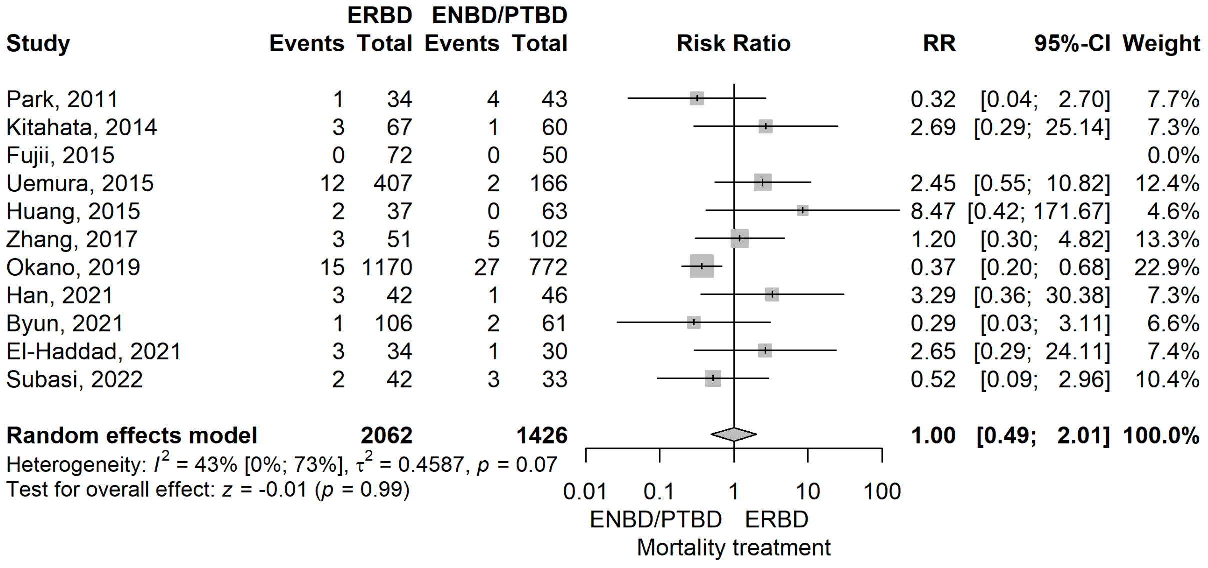

Fig. Forest plot for Mortality treatment, comparing ERBD with ENBD/PTBD

Subgroup analyses

Algorithm did not converge for subgroup analisis of

Algorithm did not converge for subgroup analisis of

Meta-analysis for Reoperation treatment, comparing ERBD with ENBD/PTBD

	datele.Year	datele.Study.name	numberCasesPerTreatment	numberCasesTreatment	numberCasesPerControl	numberCasesControl
2	2014	Kitahata, 2014	0	67	0	60
6	2017	Zhang, 2017	1	51	2	102
10	2021	Han, 2021	5	42	4	46
15	2022	Subasi, 2022	3	42	8	33

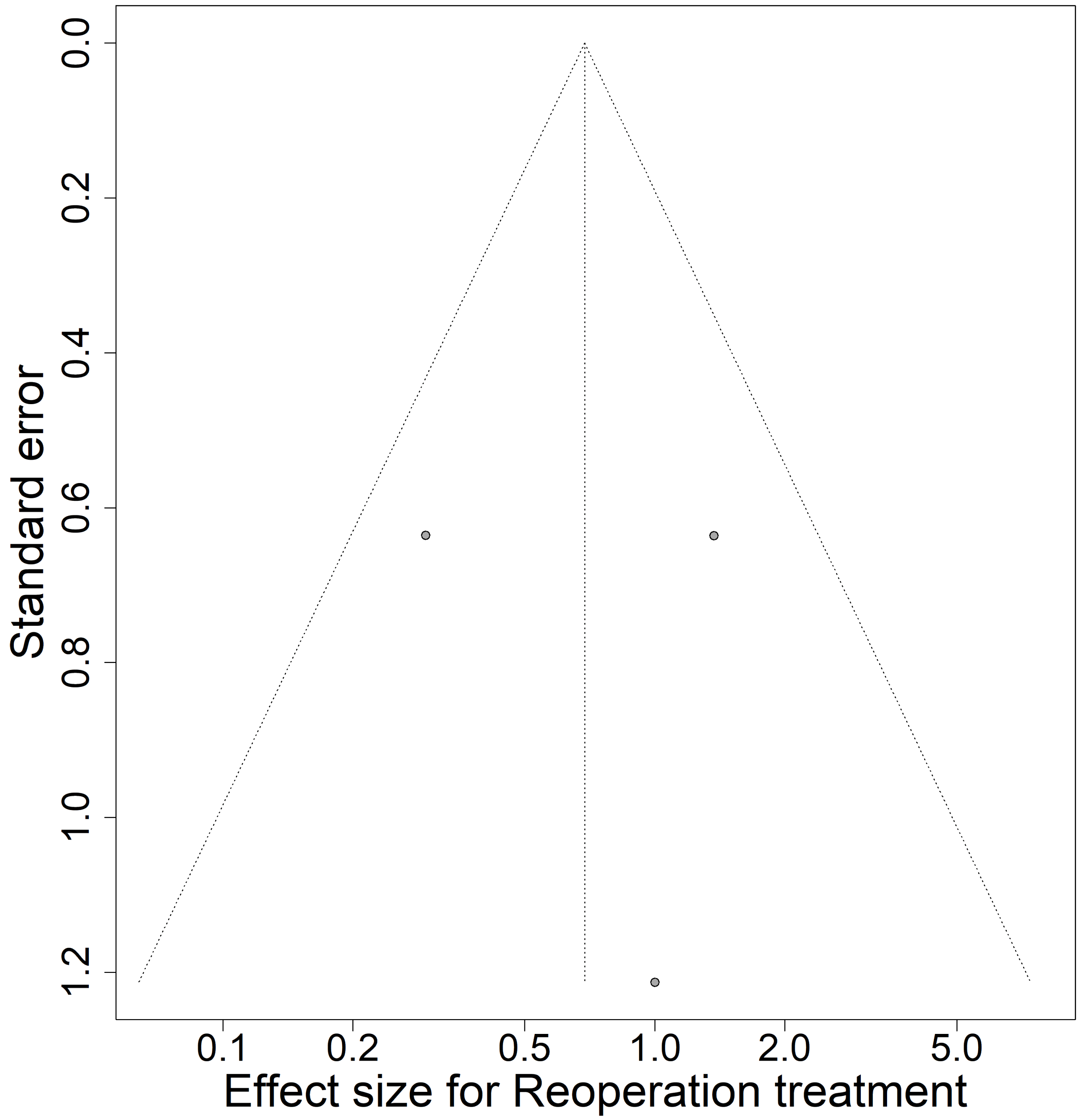

Fig. Funnel plot for Reoperation treatment, comparing ERBD with ENBD/PTBD

The funnel plot for Reoperation treatment, comparing ERBD with ENBD/PTBD is shown in figure _.

The publication bias test gave a p=0.871.

Influence studies: Omitting Zhang, 2017; Omitting Han, 2021; Omitting Subasi, 2022 - no; yes; yes

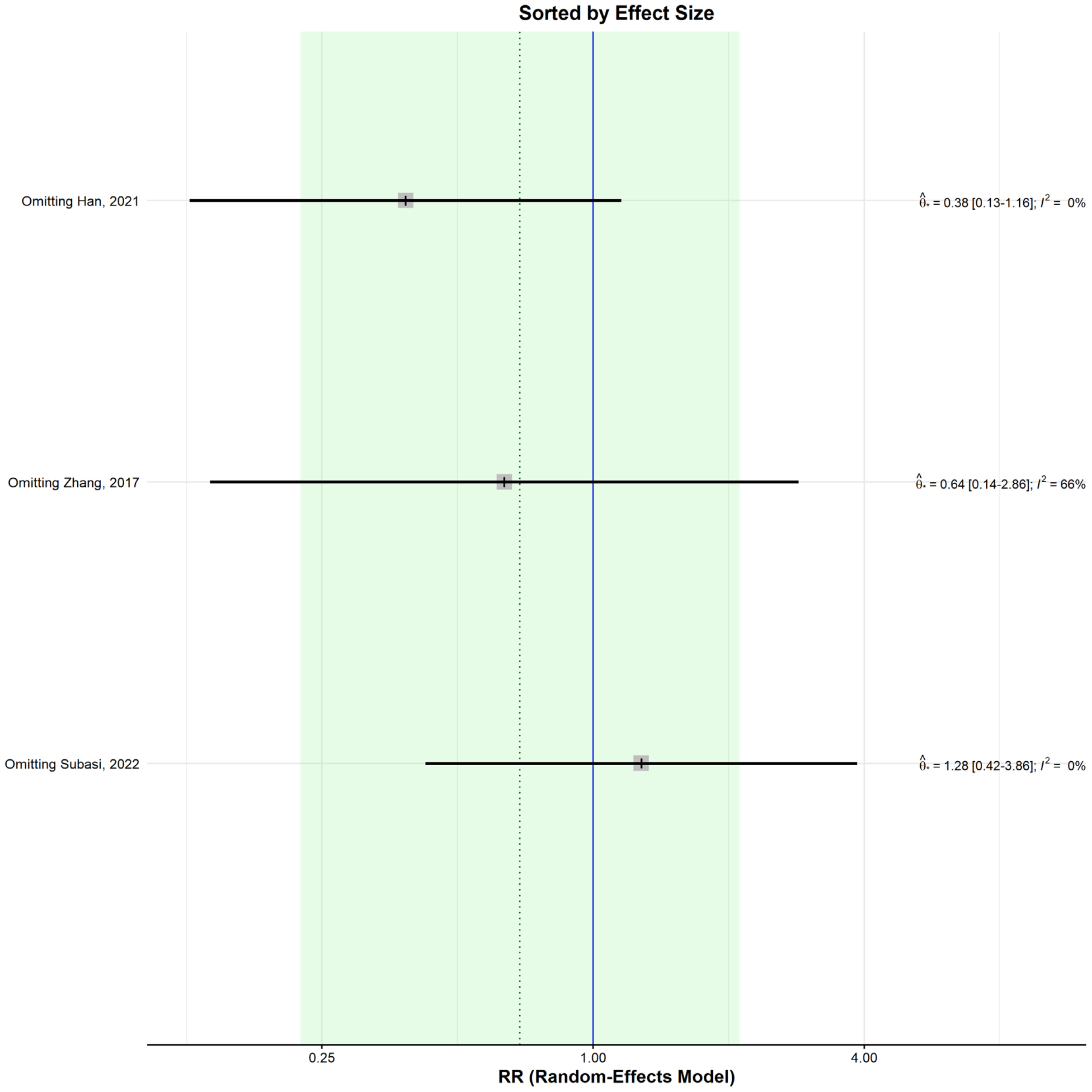

Fig. Leave-one-out sensitivity analysis plot for selected studies for Reoperation treatment

The heterogeneity was assessed, and we found an I2 of 34.2% (95% CI 0% - 78.5%) and the Q test for heterogeneity gave p=0.219.

The RR value (the RR of Reoperation treatment in the ERBD group compared to the ENBD/PTBD group) obtained with the meta-analysis was of -0.37 (95% CI -1.5 - 0.75), p=0.514 using the model with random effects.

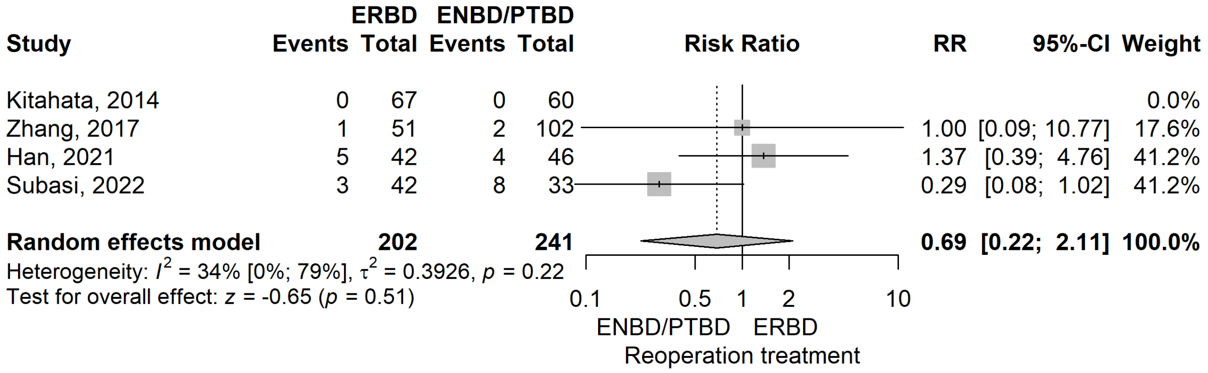

Fig. Forest plot for Reoperation treatment, comparing ERBD with ENBD/PTBD

Meta-analysis for Overall complication rate (Clavien - Dindo) treatment, comparing ERBD with ENBD/PTBD

	datele.Year	datele.Study.name	numberCasesPerTreatment	numberCasesTreatment	numberCasesPerControl	numberCasesControl
1	2011	Park, 2011	21	34	16	43
2	2014	Kitahata, 2014	28	67	14	60
4	2015	Uemura, 2015	87	407	41	166
5	2015	Huang, 2015	27	37	30	63
6	2017	Zhang, 2017	34	51	60	102
7	2018	Lee, 2018	181	335	83	234
8	2019	Mori, 2019	60	60	24	24
9	2019	Okano, 2019	433	1170	646	772
10	2021	Han, 2021	24	42	12	46
11	2021	Byun, 2021	59	106	22	61
15	2022	Subasi, 2022	42	42	33	33

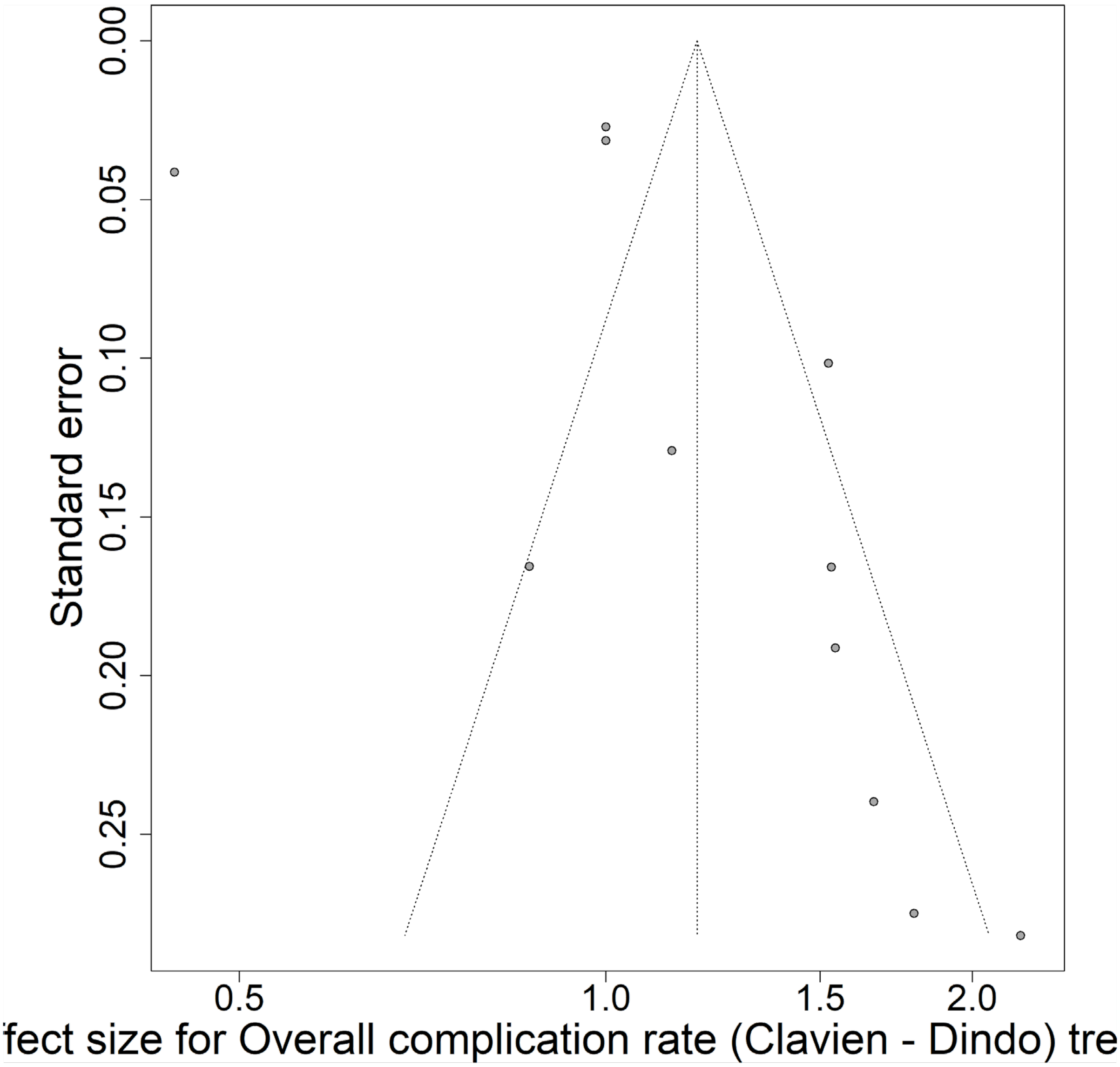

Fig. Funnel plot for Overall complication rate (Clavien - Dindo) treatment, comparing ERBD with ENBD/PTBD

The funnel plot for Overall complication rate (Clavien - Dindo) treatment, comparing ERBD with ENBD/PTBD is shown in figure _.

The publication bias test gave a p=0.399.

Influence studies: Omitting Park, 2011; Omitting Kitahata, 2014; Omitting Uemura, 2015; Omitting Huang, 2015; Omitting Zhang, 2017; Omitting Lee, 2018; Omitting Mori, 2019; Omitting Okano, 2019; Omitting Han, 2021; Omitting Byun, 2021; Omitting Subasi, 2022 - no; no; no; no; no; no; no; yes; no; no; no

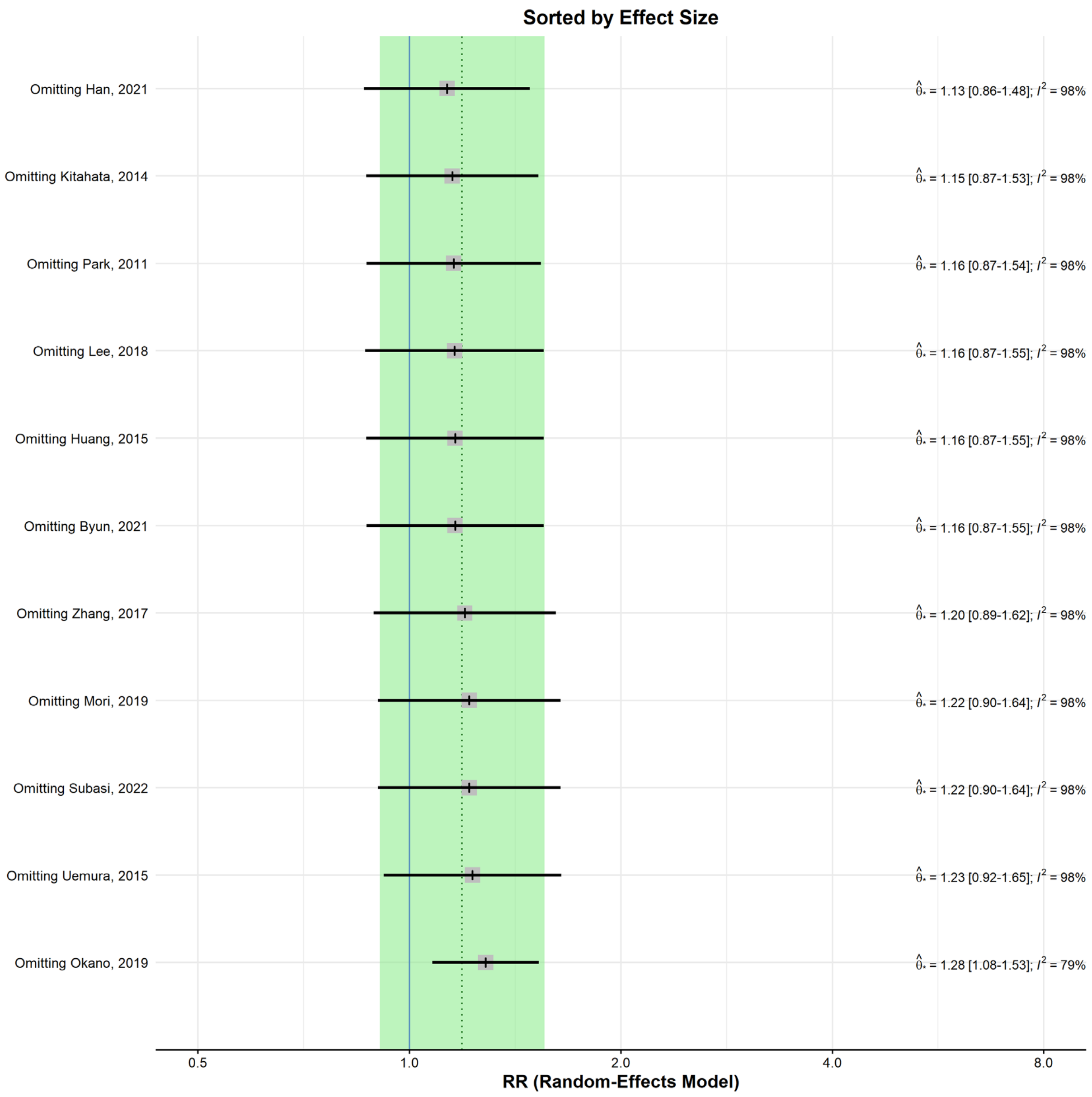

Fig. Leave-one-out sensitivity analysis plot for selected studies for Overall complication rate (Clavien - Dindo) treatment

The heterogeneity was assessed, and we found an I2 of 97.5% (95% CI 96.5% - 98.1%) and the Q test for heterogeneity gave p=< 0.001.

The RR value (the RR of Overall complication rate (Clavien - Dindo) treatment in the ERBD group compared to the ENBD/PTBD group) obtained with the meta-analysis was of 0.17 (95% CI -0.1 - 0.44), p=0.21 using the model with random effects.

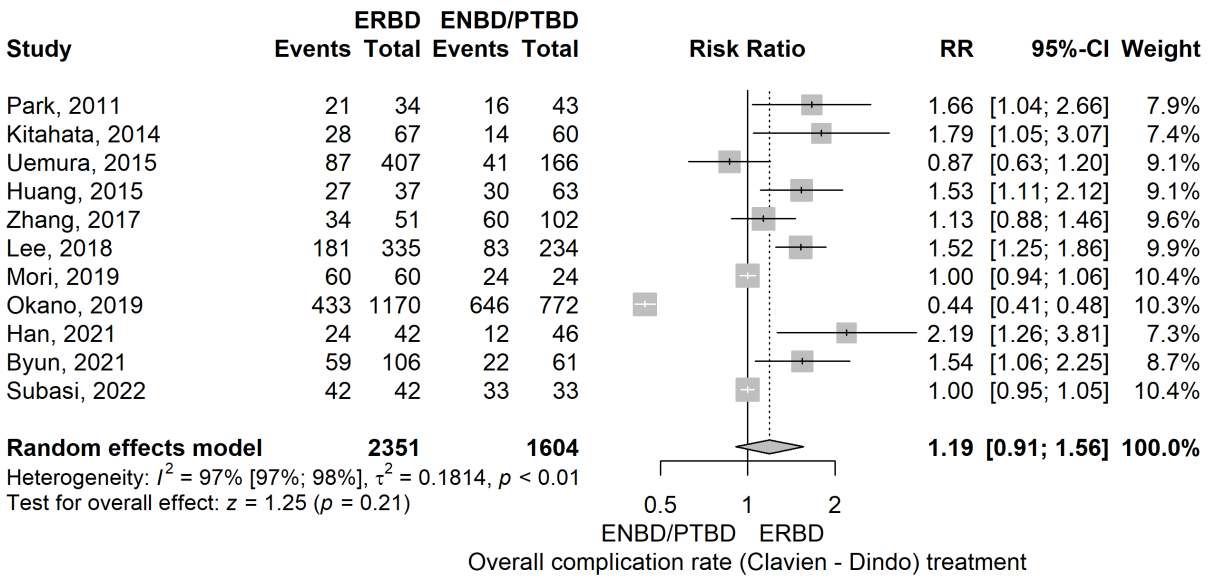

Fig. Forest plot for Overall complication rate (Clavien - Dindo) treatment, comparing ERBD with ENBD/PTBD

Subgroup analyses

Algorithm did not converge for subgroup analisis of

Algorithm did not converge for subgroup analisis of

Meta-analysis for Grade 1-2 treatment, comparing ERBD with ENBD/PTBD

	datele.Year	datele.Study.name	numberCasesPerTreatment	numberCasesTreatment	numberCasesPerControl	numberCasesControl
2	2014	Kitahata, 2014	2	67	2	60
5	2015	Huang, 2015	18	37	25	63
6	2017	Zhang, 2017	21	51	39	102
8	2019	Mori, 2019	47	60	15	24
9	2019	Okano, 2019	306	1170	369	772
15	2022	Subasi, 2022	32	42	20	33

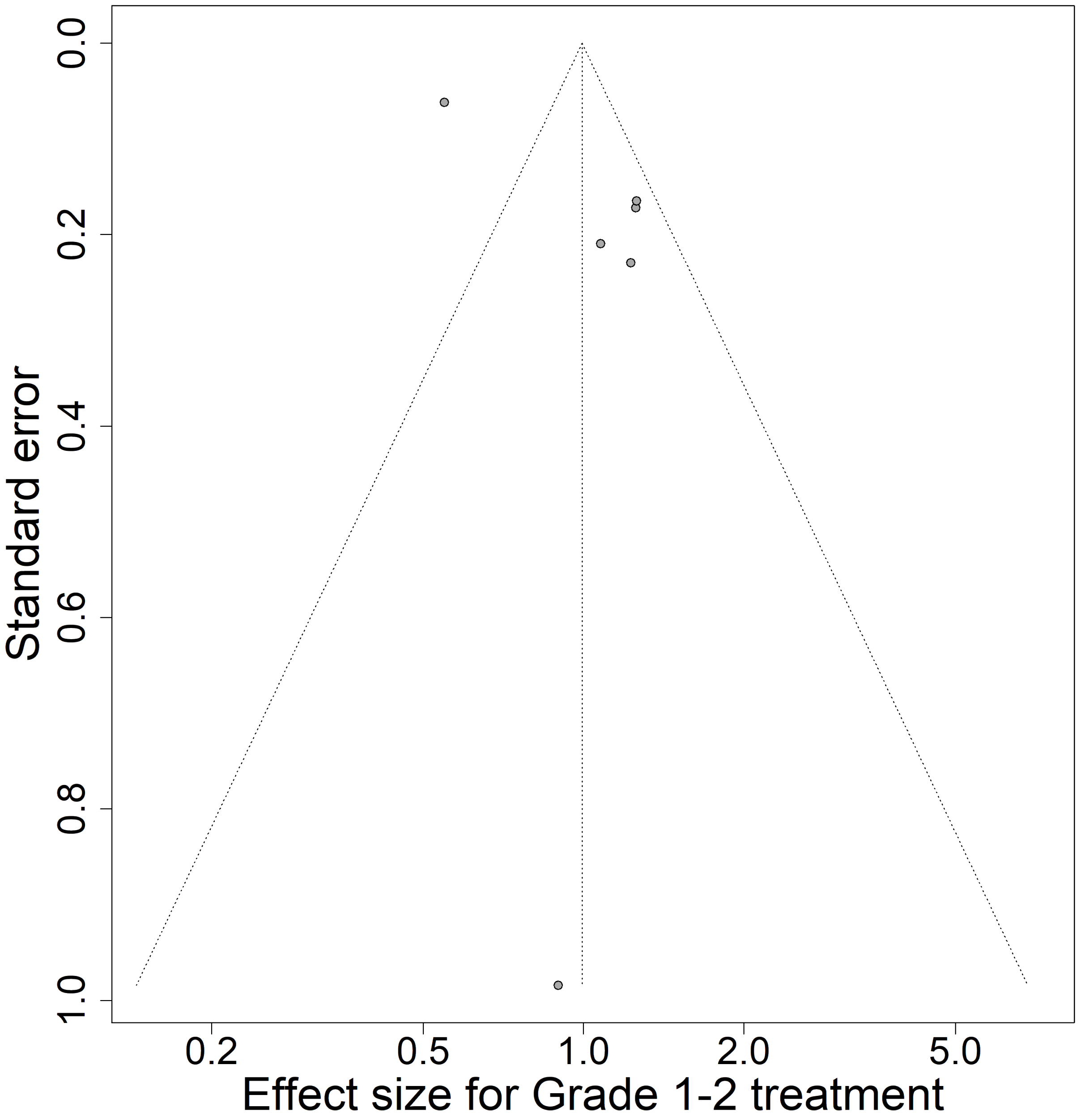

Fig. Funnel plot for Grade 1-2 treatment, comparing ERBD with ENBD/PTBD

The funnel plot for Grade 1-2 treatment, comparing ERBD with ENBD/PTBD is shown in figure _.

The publication bias test gave a p=0.071.

Influence studies: Omitting Kitahata, 2014; Omitting Huang, 2015; Omitting Zhang, 2017; Omitting Mori, 2019; Omitting Okano, 2019; Omitting Subasi, 2022 - no; no; no; no; yes; no

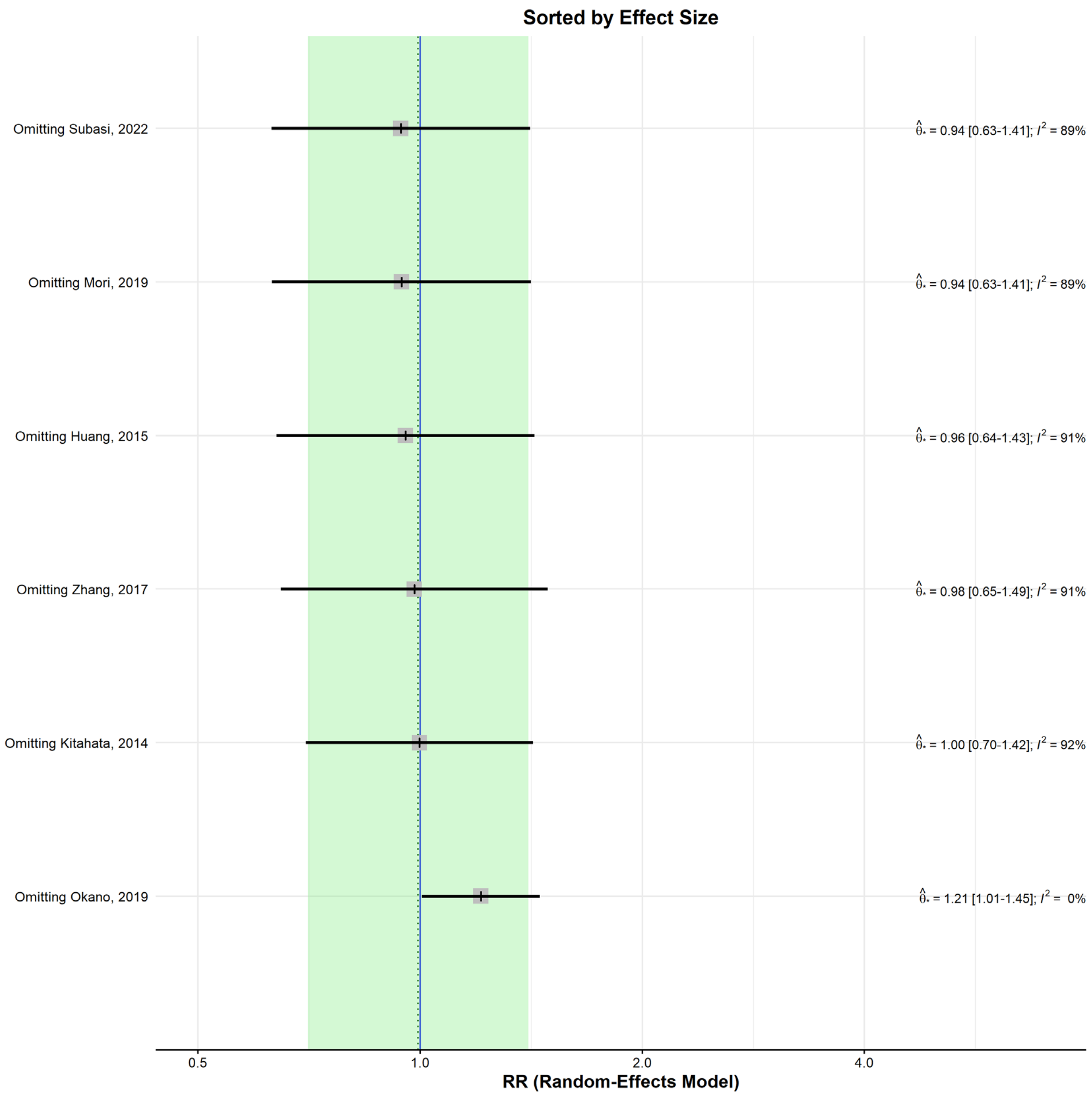

Fig. Leave-one-out sensitivity analysis plot for selected studies for Grade 1-2 treatment

The heterogeneity was assessed, and we found an I2 of 90% (95% CI 81% - 94.8%) and the Q test for heterogeneity gave p=< 0.001.

The RR value (the RR of Grade 1-2 treatment in the ERBD group compared to the ENBD/PTBD group) obtained with the meta-analysis was of -0.01 (95% CI -0.35 - 0.34), p=0.972 using the model with random effects.

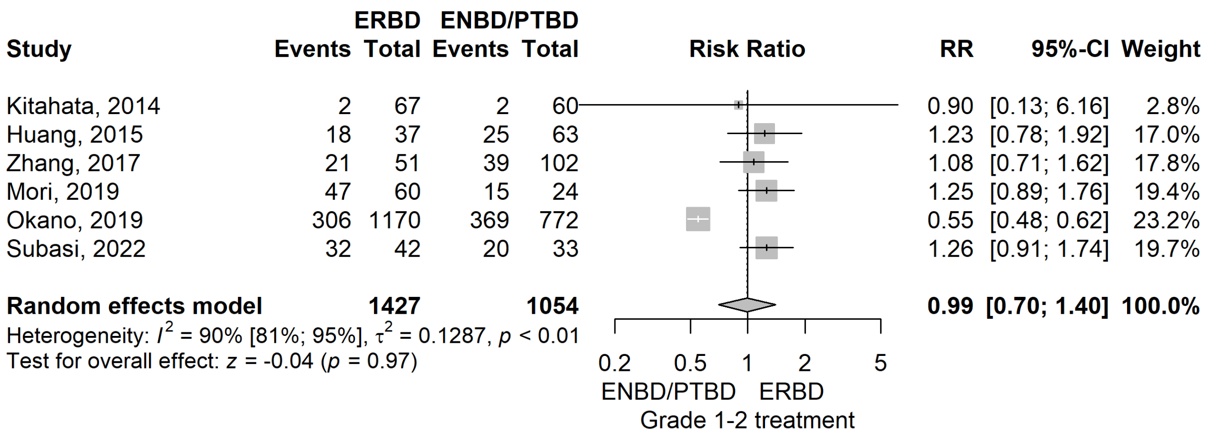

Fig. Forest plot for Grade 1-2 treatment, comparing ERBD with ENBD/PTBD

Meta-analysis for Grade >=3 treatment, comparing ERBD with ENBD/PTBD

	datele.Year	datele.Study.name	numberCasesPerTreatment	numberCasesTreatment	numberCasesPerControl	numberCasesControl
2	2014	Kitahata, 2014	26	67	12	60
4	2015	Uemura, 2015	87	407	41	166
5	2015	Huang, 2015	9	37	5	63
6	2017	Zhang, 2017	13	51	21	102
8	2019	Mori, 2019	13	60	9	24
9	2019	Okano, 2019	127	1170	277	772
15	2022	Subasi, 2022	10	42	13	33

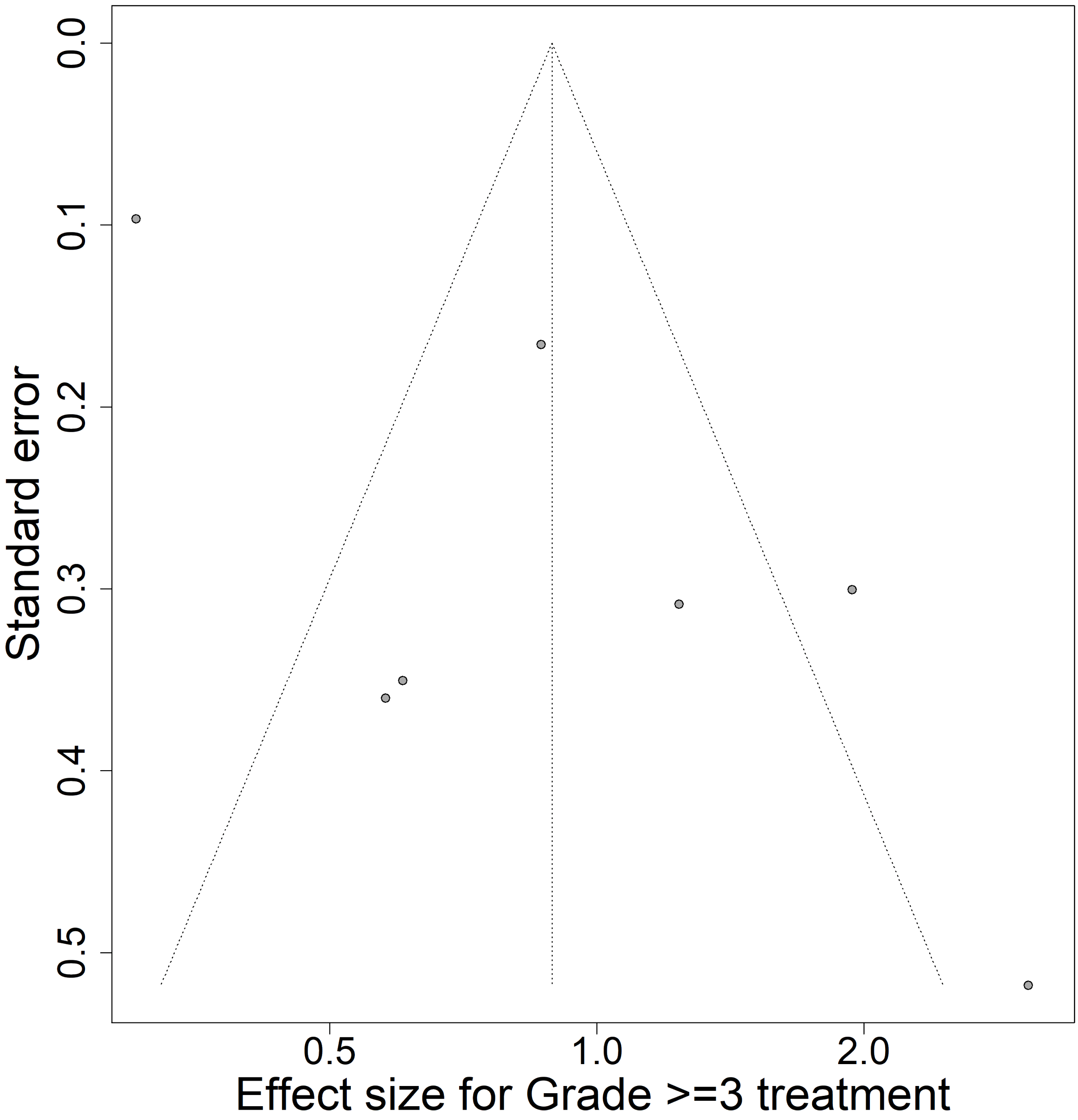

Fig. Funnel plot for Grade >=3 treatment, comparing ERBD with ENBD/PTBD

The funnel plot for Grade >=3 treatment, comparing ERBD with ENBD/PTBD is shown in figure _.

The publication bias test gave a p=0.031.

Influence studies: Omitting Kitahata, 2014; Omitting Uemura, 2015; Omitting Huang, 2015; Omitting Zhang, 2017; Omitting Mori, 2019; Omitting Okano, 2019; Omitting Subasi, 2022 - no; no; no; no; no; yes; no

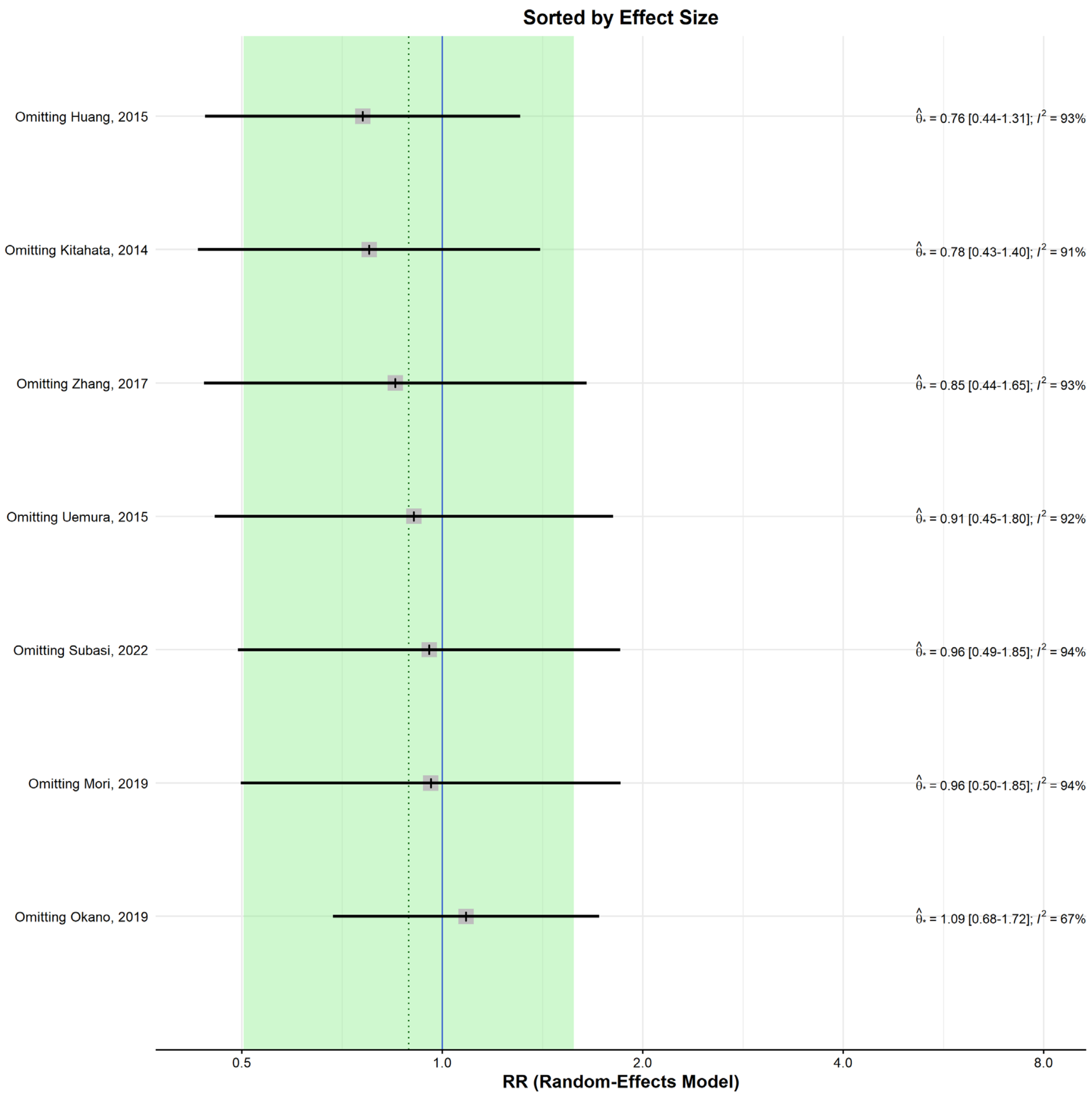

Fig. Leave-one-out sensitivity analysis plot for selected studies for Grade >=3 treatment

The heterogeneity was assessed, and we found an I2 of 92.5% (95% CI 87% - 95.6%) and the Q test for heterogeneity gave p=< 0.001.

The RR value (the RR of Grade >=3 treatment in the ERBD group compared to the ENBD/PTBD group) obtained with the meta-analysis was of -0.12 (95% CI -0.69 - 0.45), p=0.69 using the model with random effects.

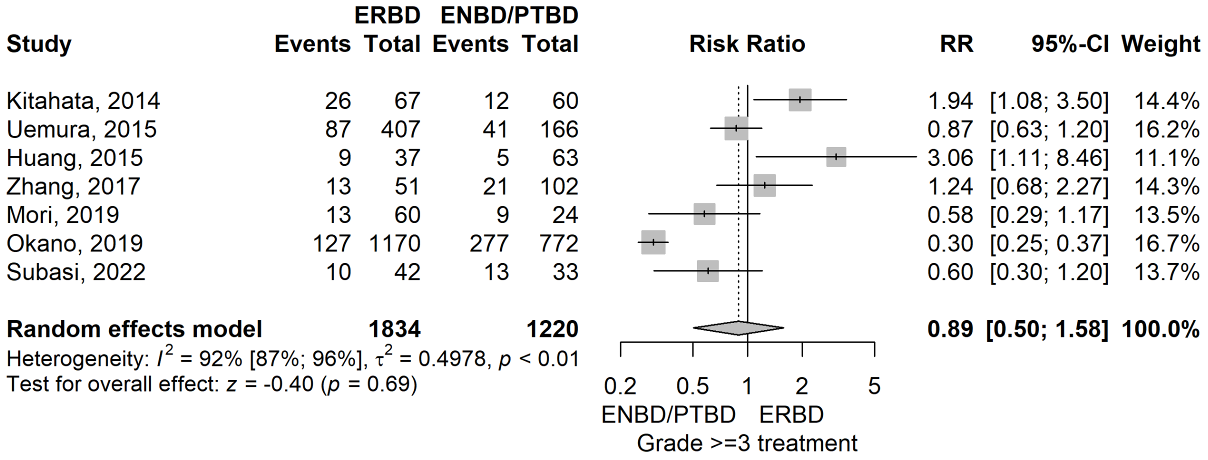

Fig. Forest plot for Grade >=3 treatment, comparing ERBD with ENBD/PTBD

Meta-analysis for Infectious complications treatment, comparing ERBD with ENBD/PTBD

	datele.Year	datele.Study.name	numberCasesPerTreatment	numberCasesTreatment	numberCasesPerControl	numberCasesControl
2	2014	Kitahata, 2014	16	67	9	60
3	2015	Fujii, 2015	31	72	7	50
9	2019	Okano, 2019	293	1170	445	772
12	2021	El-Haddad, 2021	11	34	7	30
13	2021	Suenaga, 2021	13	40	15	38

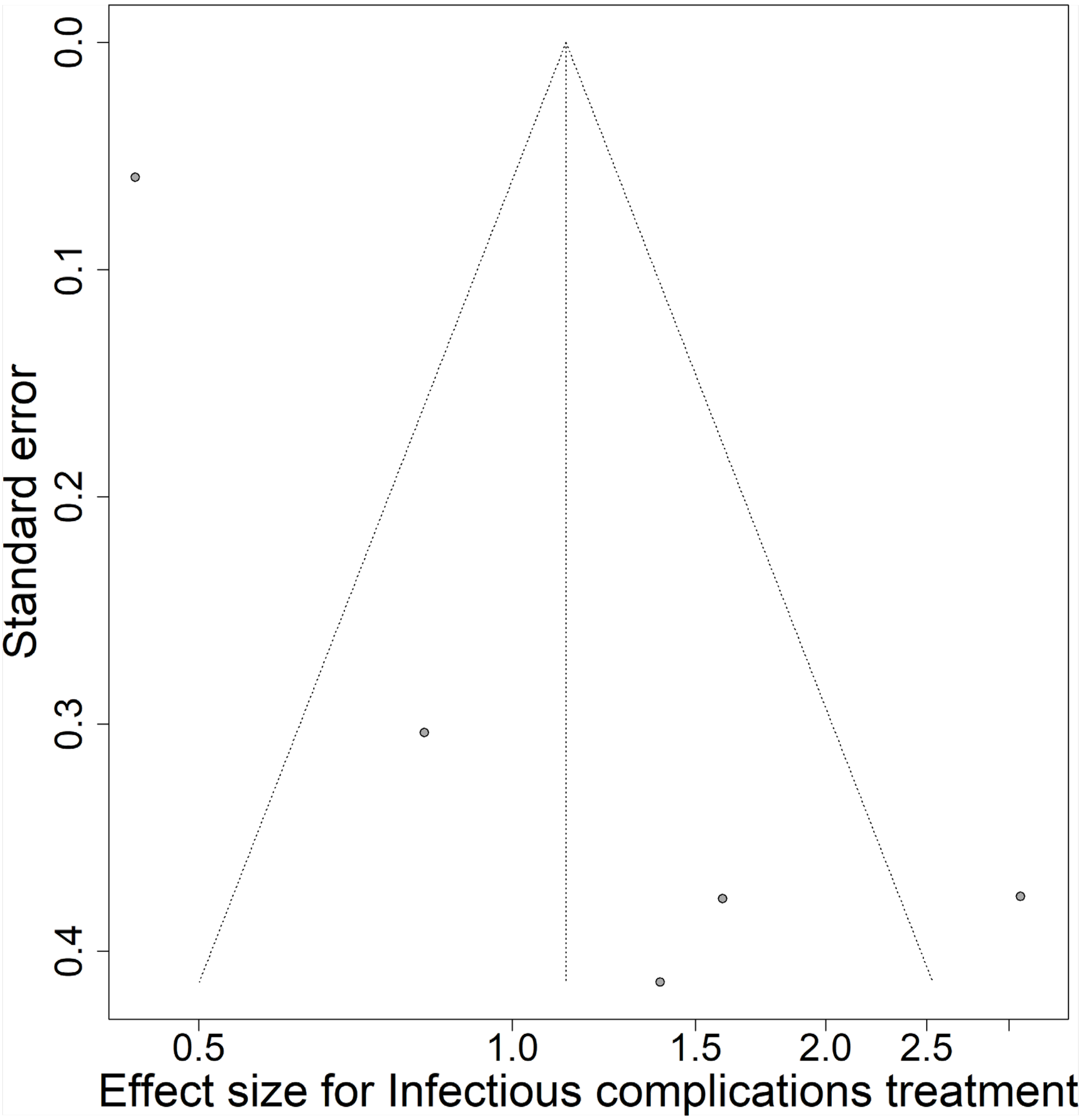

Fig. Funnel plot for Infectious complications treatment, comparing ERBD with ENBD/PTBD

The funnel plot for Infectious complications treatment, comparing ERBD with ENBD/PTBD is shown in figure _.

The publication bias test gave a p=0.015.

Influence studies: Omitting Kitahata, 2014; Omitting Fujii, 2015; Omitting Okano, 2019; Omitting El-Haddad, 2021; Omitting Suenaga, 2021 - no; yes; yes; no; no

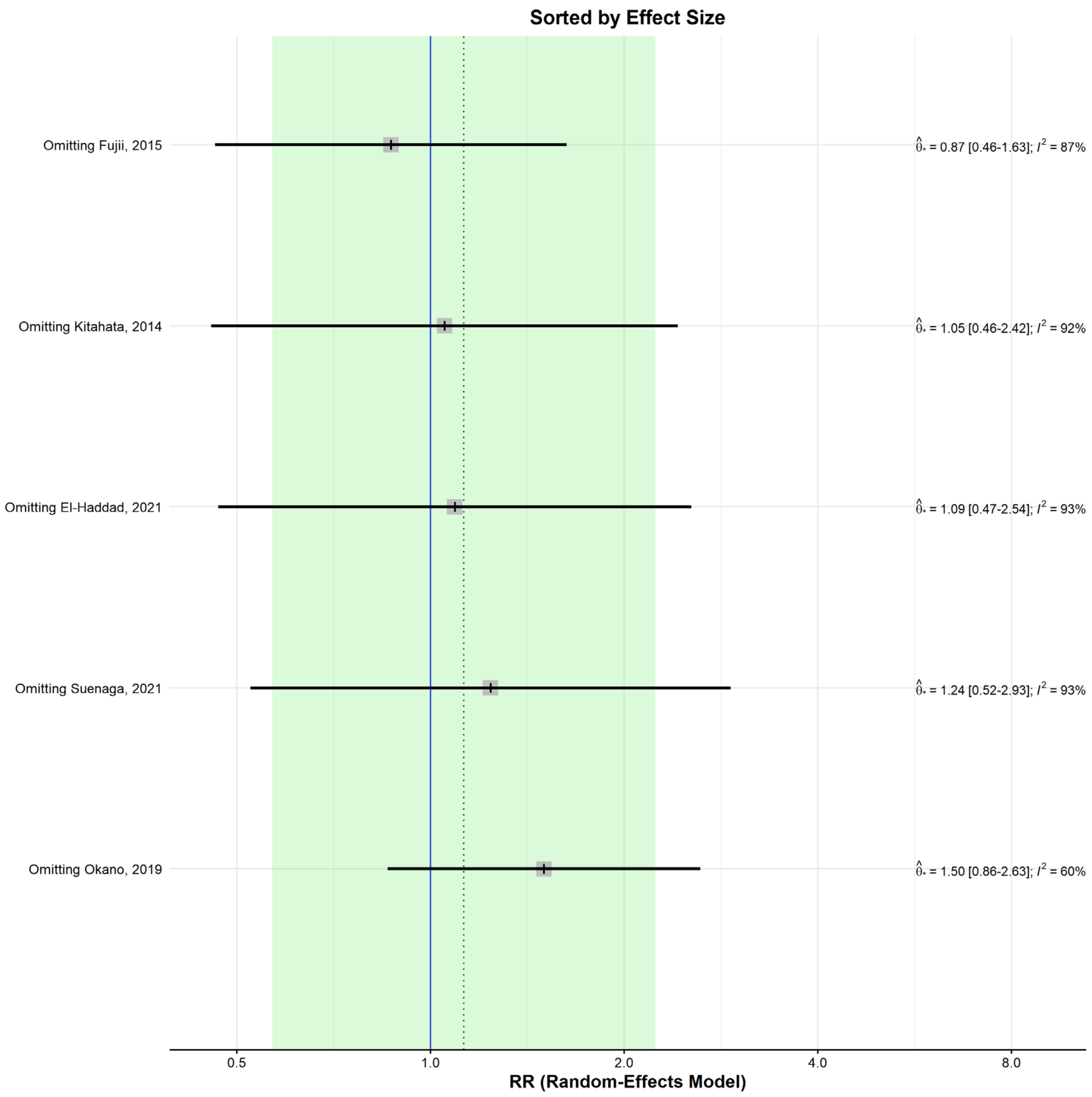

Fig. Leave-one-out sensitivity analysis plot for selected studies for Infectious complications treatment

The heterogeneity was assessed, and we found an I2 of 91.5% (95% CI 83.1% - 95.7%) and the Q test for heterogeneity gave p=< 0.001.

The RR value (the RR of Infectious complications treatment in the ERBD group compared to the ENBD/PTBD group) obtained with the meta-analysis was of 0.12 (95% CI -0.57 - 0.8), p=0.733 using the model with random effects.

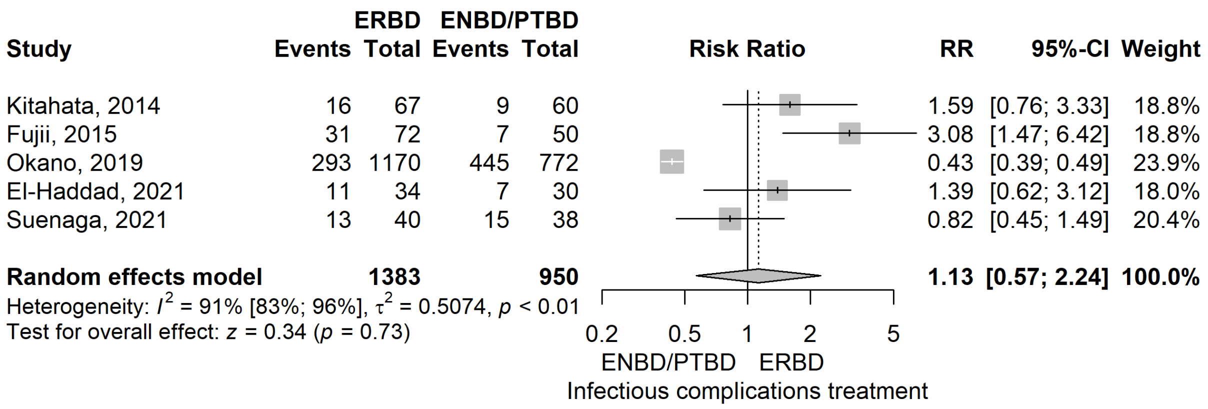

Fig. Forest plot for Infectious complications treatment, comparing ERBD with ENBD/PTBD

Meta-analysis for Sepsis treatment, comparing ERBD with ENBD/PTBD

	datele.Year	datele.Study.name	numberCasesPerTreatment	numberCasesTreatment	numberCasesPerControl	numberCasesControl
2	2014	Kitahata, 2014	4	67	1	60
3	2015	Fujii, 2015	6	72	2	50
5	2015	Huang, 2015	2	37	2	63
14	2022	Satoh, 2022	6	117	0	39
2						

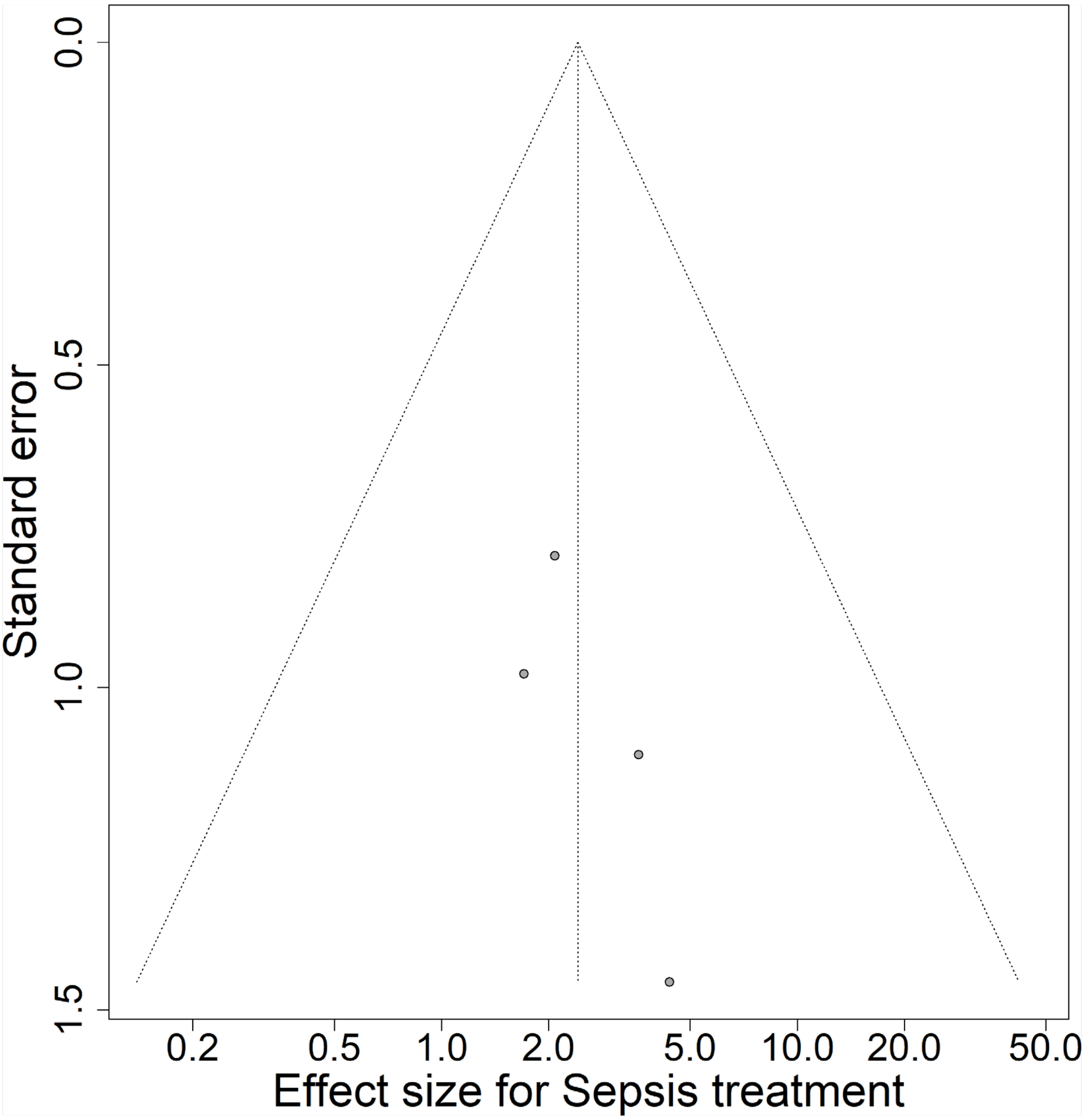

Fig. Funnel plot for Sepsis treatment, comparing ERBD with ENBD/PTBD

The funnel plot for Sepsis treatment, comparing ERBD with ENBD/PTBD is shown in figure _.

The publication bias test gave a p=0.227.

Influence studies: Omitting Kitahata, 2014; Omitting Fujii, 2015; Omitting Huang, 2015; Omitting Satoh, 2022 - no; no; no; no

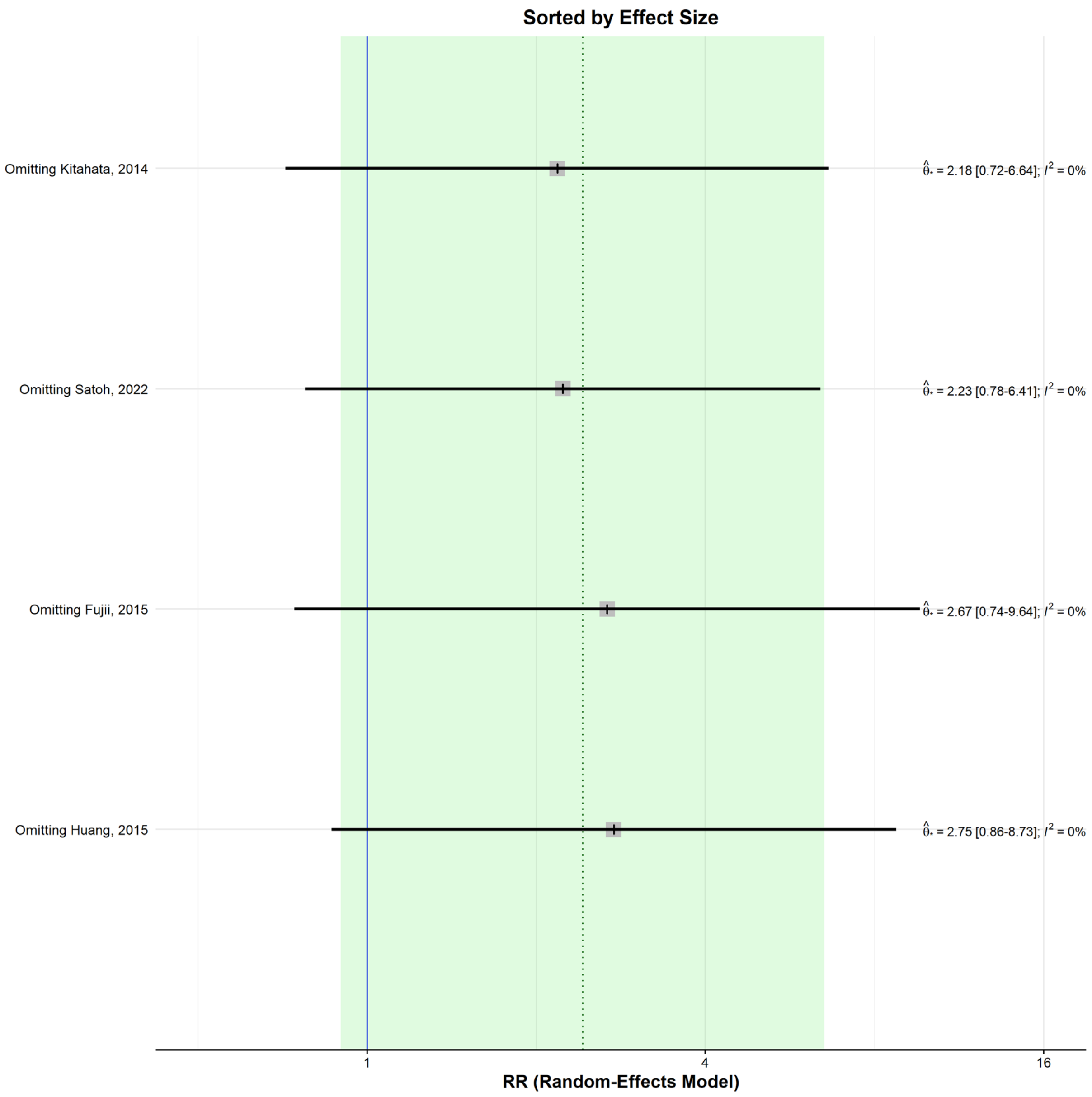

Fig. Leave-one-out sensitivity analysis plot for selected studies for Sepsis treatment

The heterogeneity was assessed, and we found an I2 of 0% (95% CI 0% - 84.7%) and the Q test for heterogeneity gave p=0.929.

The RR value (the RR of Sepsis treatment in the ERBD group compared to the ENBD/PTBD group) obtained with the meta-analysis was of 0.88 (95% CI -0.11 - 1.87), p=0.081 using the model with random effects.

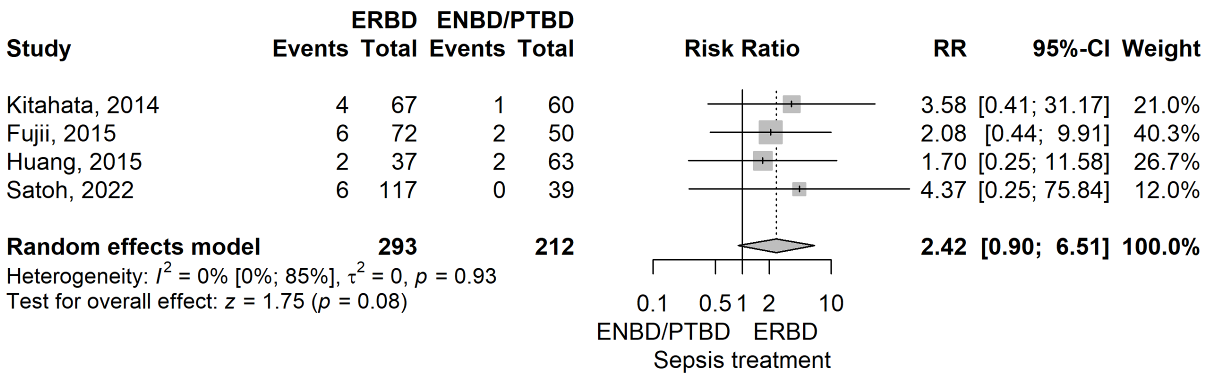

Fig. Forest plot for Sepsis treatment, comparing ERBD with ENBD/PTBD

Meta-analysis for Intraabdominal abcess treatment, comparing ERBD with ENBD/PTBD

	datele.Year	datele.Study.name	numberCasesPerTreatment	numberCasesTreatment	numberCasesPerControl	numberCasesControl
1	2011	Park, 2011	12	34	10	43
2	2014	Kitahata, 2014	9	67	7	60
3	2015	Fujii, 2015	15	72	3	50
4	2015	Uemura, 2015	11	407	5	166
5	2015	Huang, 2015	8	37	9	63
6	2017	Zhang, 2017	22	51	25	102
14	2022	Satoh, 2022	23	117	1	39

2

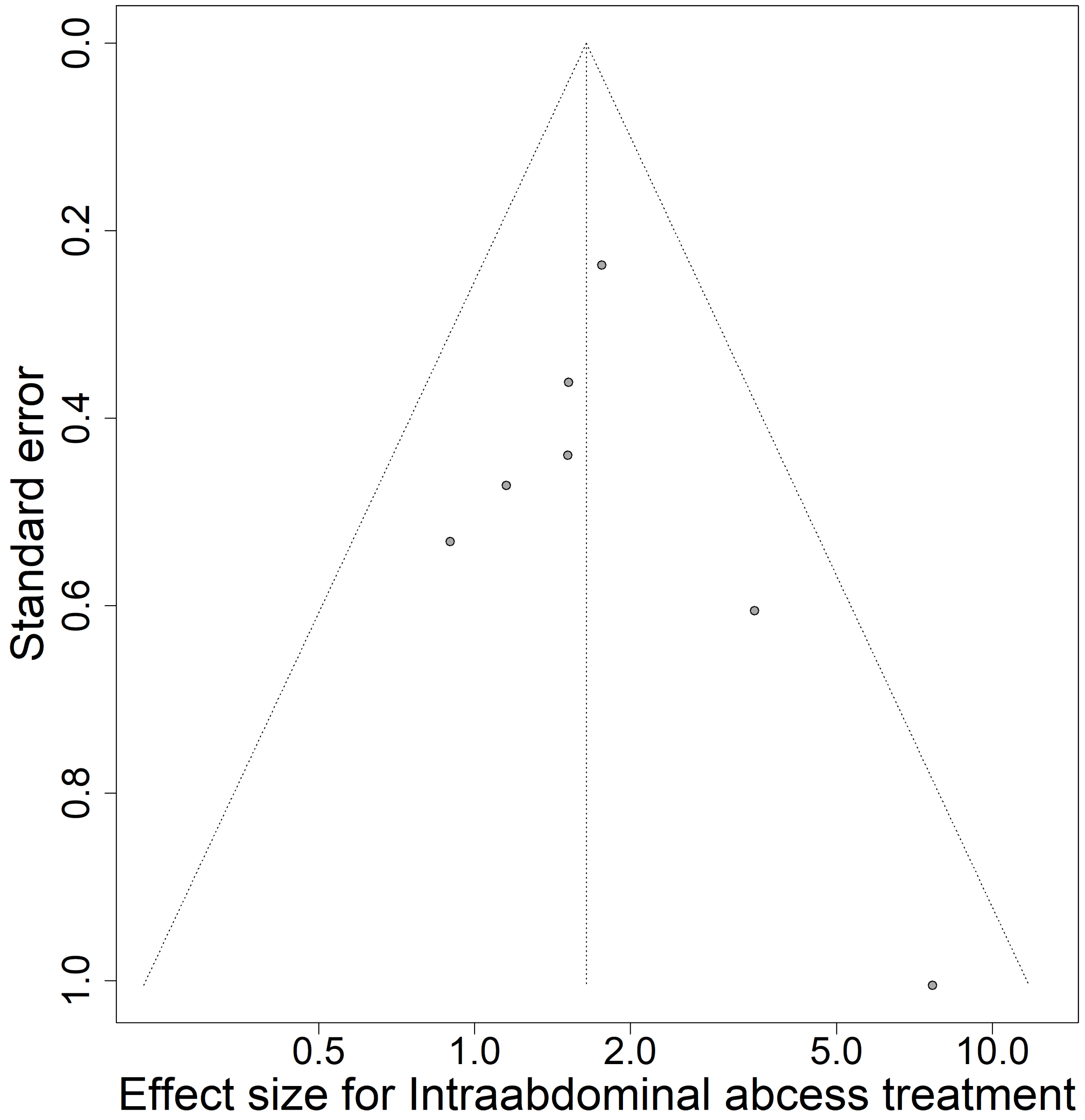

Fig. Funnel plot for Intraabdominal abcess treatment, comparing ERBD with ENBD/PTBD

The funnel plot for Intraabdominal abcess treatment, comparing ERBD with ENBD/PTBD is shown in figure _.

The publication bias test gave a p=0.506.

Influence studies: Omitting Park, 2011; Omitting Kitahata, 2014; Omitting Fujii, 2015; Omitting Uemura, 2015; Omitting Huang, 2015; Omitting Zhang, 2017; Omitting Satoh, 2022 - no; no; no; no; no; no; no

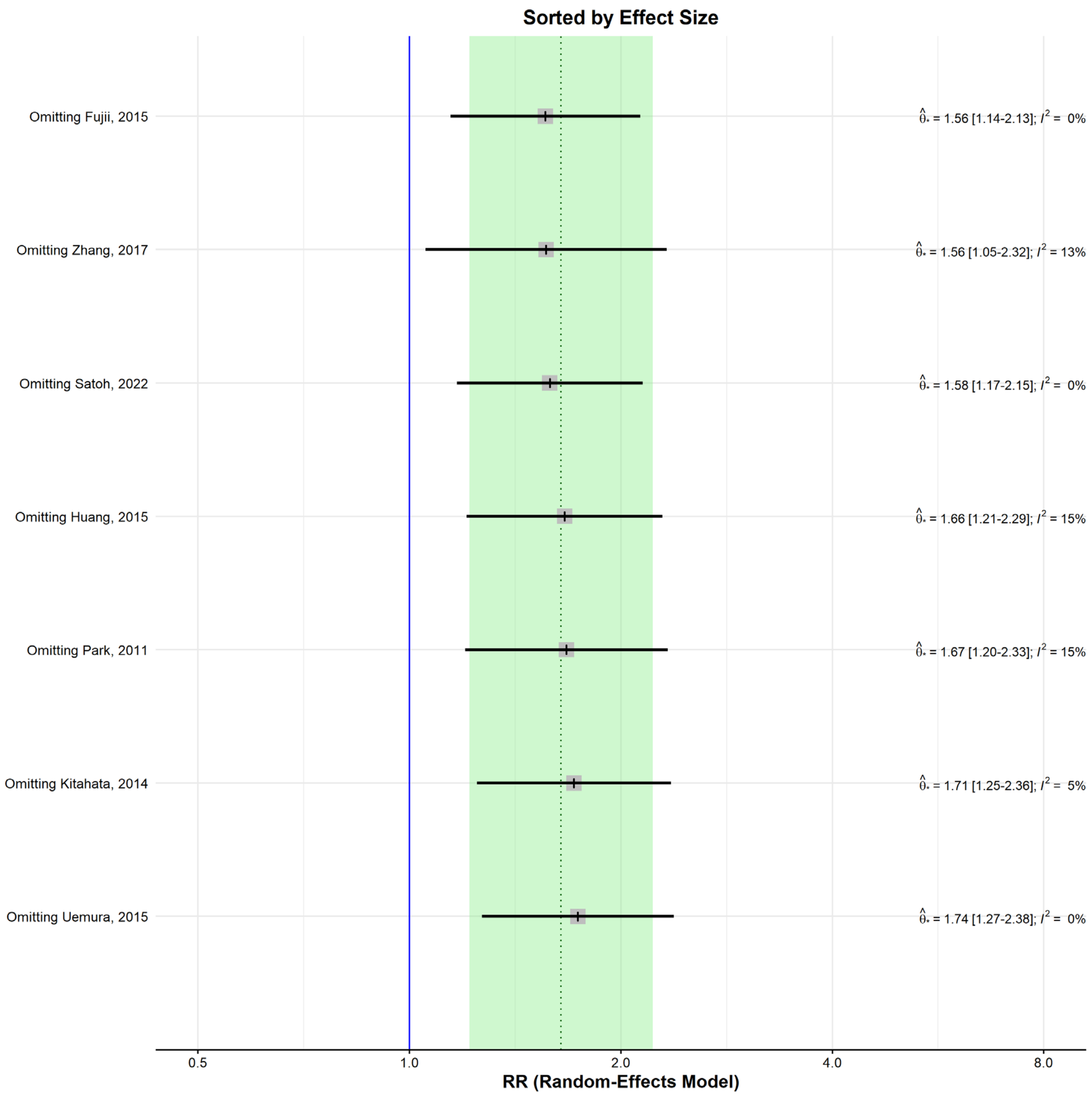

Fig. Leave-one-out sensitivity analysis plot for selected studies for Intraabdominal abcess treatment

The heterogeneity was assessed, and we found an I2 of 0% (95% CI 0% - 70.8%) and the Q test for heterogeneity gave p=0.433.

The RR value (the RR of Intraabdominal abcess treatment in the ERBD group compared to the ENBD/PTBD group) obtained with the meta-analysis was of 0.5 (95% CI 0.2 - 0.8), p=0.001 using the model with random effects.

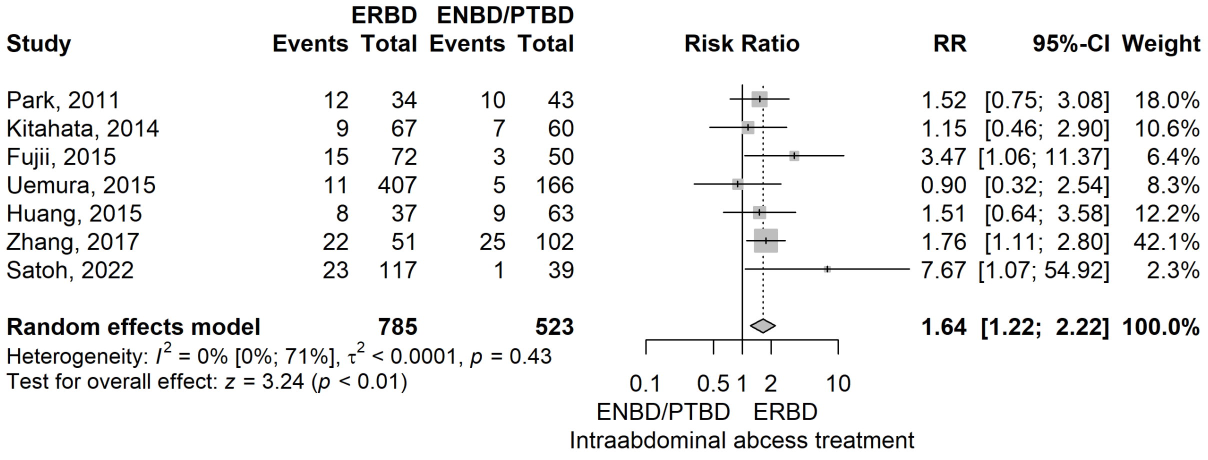

Fig. Forest plot for Intraabdominal abcess treatment, comparing ERBD with ENBD/PTBD

Meta-analysis for Wound infections treatment, comparing ERBD with ENBD/PTBD

	datele.Year	datele.Study.name	numberCasesPerTreatment	numberCasesTreatment	numberCasesPerControl	numberCasesControl
1	2011	Park, 2011	1	34	4	43
2	2014	Kitahata, 2014	7	67	2	60
4	2015	Uemura, 2015	20	407	12	166
5	2015	Huang, 2015	9	37	12	63
6	2017	Zhang, 2017	10	51	17	102
7	2018	Lee, 2018	59	335	25	234
14	2022	Satoh, 2022	30	117	4	39
15	2022	Subasi, 2022	6	42	5	33

2

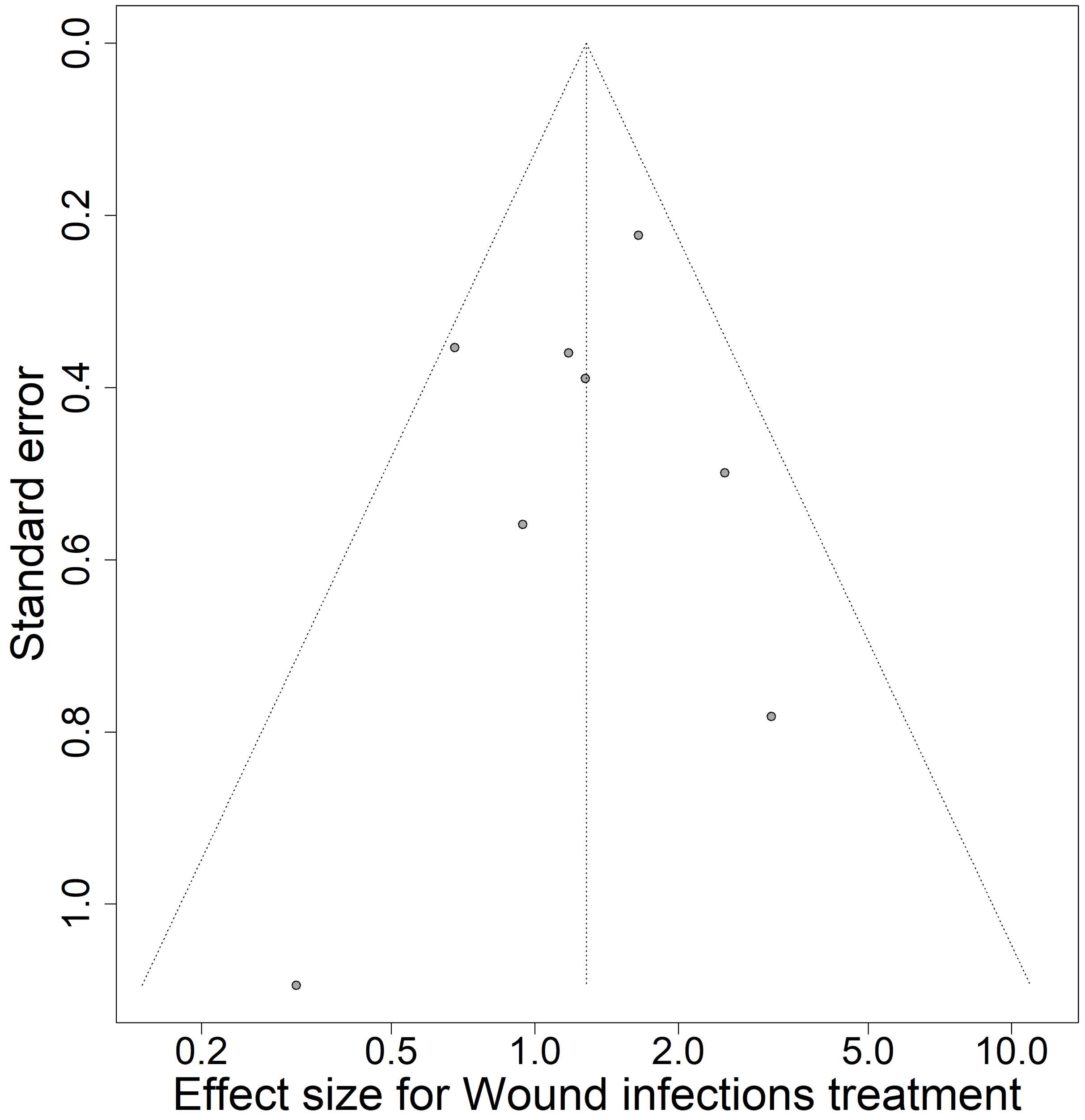

Fig. Funnel plot for Wound infections treatment, comparing ERBD with ENBD/PTBD

The funnel plot for Wound infections treatment, comparing ERBD with ENBD/PTBD is shown in figure _.

The publication bias test gave a p=0.631.

Influence studies: Omitting Park, 2011; Omitting Kitahata, 2014; Omitting Uemura, 2015; Omitting Huang, 2015; Omitting Zhang, 2017; Omitting Lee, 2018; Omitting Satoh, 2022; Omitting Subasi, 2022 - no; no; yes; no; no; no; no; no

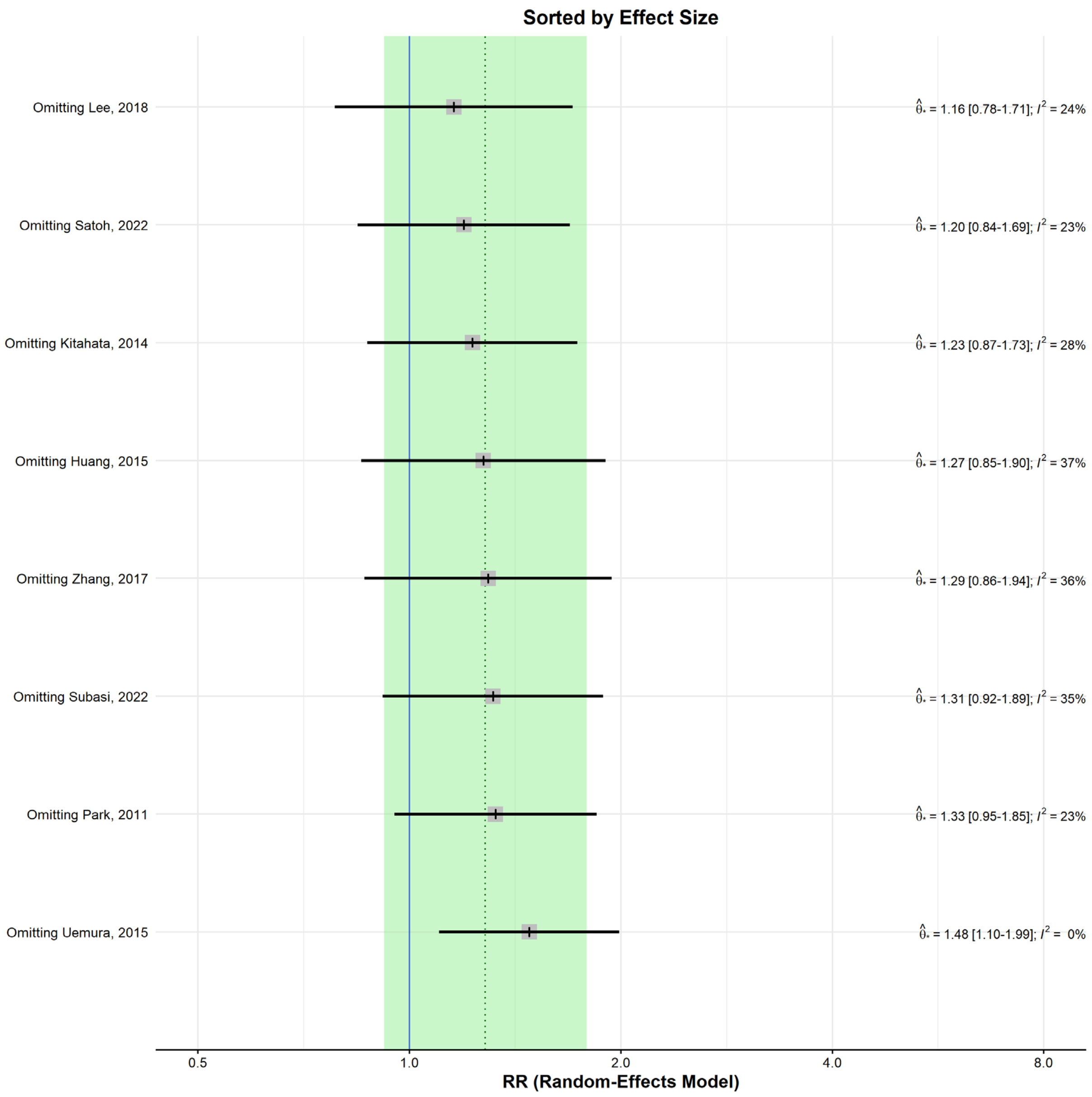

Fig. Leave-one-out sensitivity analysis plot for selected studies for Wound infections treatment

The heterogeneity was assessed, and we found an I2 of 26.8% (95% CI 0% - 67%) and the Q test for heterogeneity gave p=0.215.

The RR value (the RR of Wound infections treatment in the ERBD group compared to the ENBD/PTBD group) obtained with the meta-analysis was of 0.25 (95% CI -0.08 - 0.58), p=0.142 using the model with random effects.

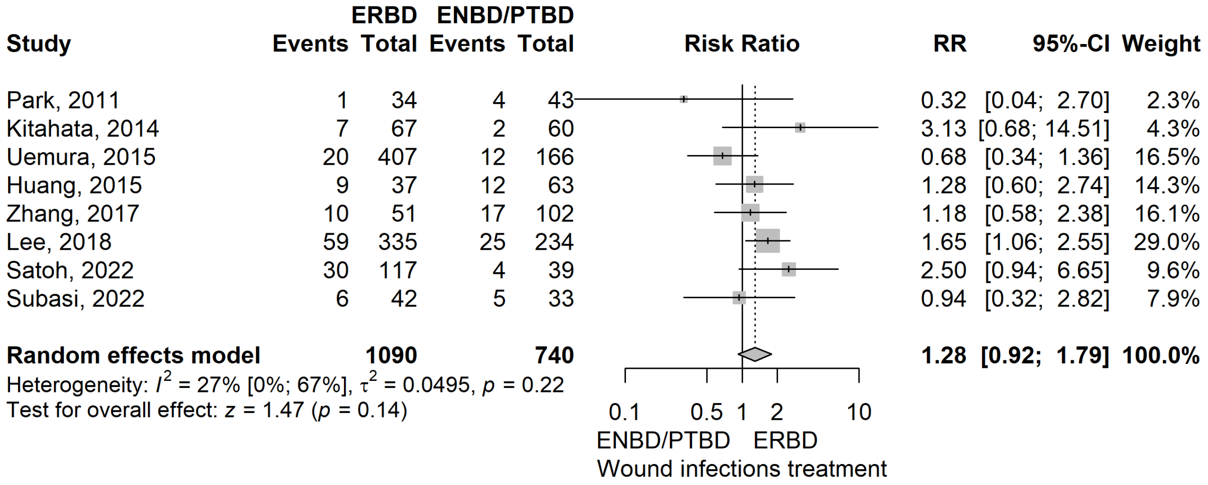

Fig. Forest plot for Wound infections treatment, comparing ERBD with ENBD/PTBD

Meta-analysis for PPH treatment, comparing ERBD with ENBD/PTBD

	datele.Year	datele.Study.name	numberCasesPerTreatment	numberCasesTreatment	numberCasesPerControl	numberCasesControl
1	2011	Park, 2011	5	34	6	43
2	2014	Kitahata, 2014	5	67	2	60
5	2015	Huang, 2015	5	37	4	63
6	2017	Zhang, 2017	5	51	7	102
7	2018	Lee, 2018	16	335	9	234
9	2019	Okano, 2019	36	1170	68	772
10	2021	Han, 2021	6	42	1	46
12	2021	El-Haddad, 2021	2	34	0	30
15	2022	Subasi, 2022	2	42	2	33

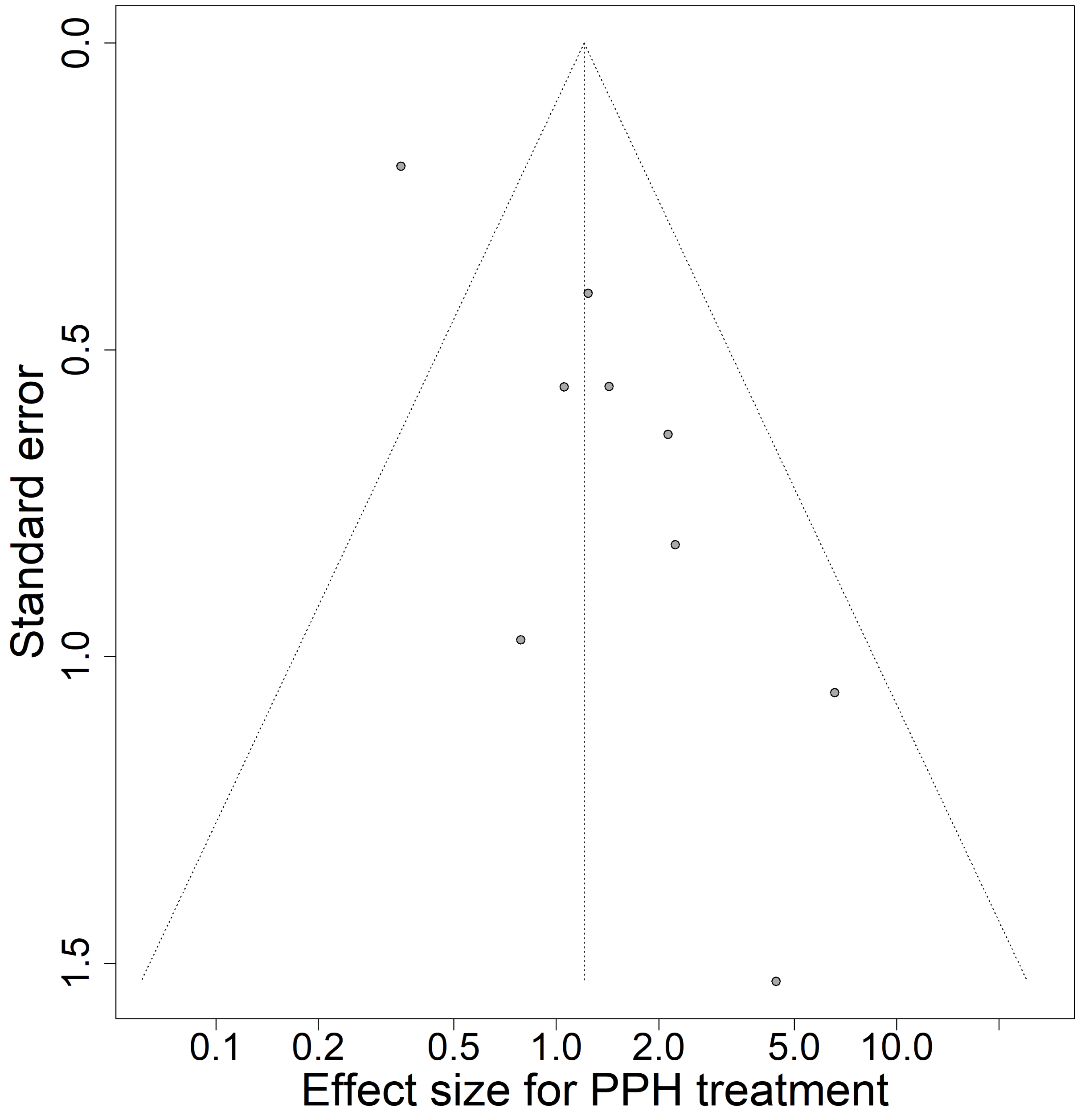

Fig. Funnel plot for PPH treatment, comparing ERBD with ENBD/PTBD

The funnel plot for PPH treatment, comparing ERBD with ENBD/PTBD is shown in figure _.

The publication bias test gave a p=0.002.

Influence studies: Omitting Park, 2011; Omitting Kitahata, 2014; Omitting Huang, 2015; Omitting Zhang, 2017; Omitting Lee, 2018; Omitting Okano, 2019; Omitting Han, 2021; Omitting El-Haddad, 2021; Omitting Subasi, 2022 - no; no; no; no; no; yes; no; no; no

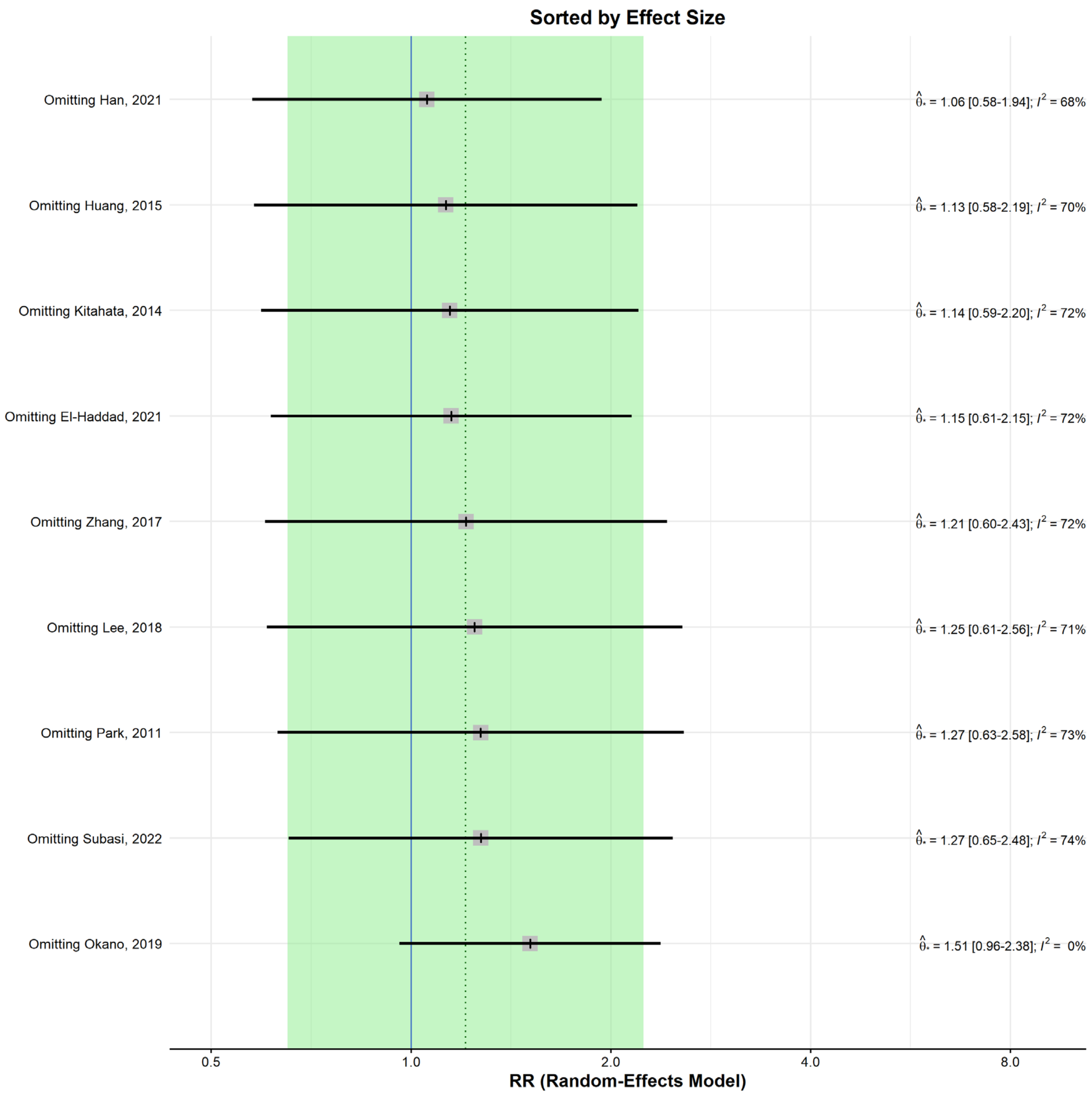

Fig. Leave-one-out sensitivity analysis plot for selected studies for PPH treatment

The heterogeneity was assessed, and we found an I2 of 70.3% (95% CI 41% - 85%) and the Q test for heterogeneity gave p=< 0.001.

The RR value (the RR of PPH treatment in the ERBD group compared to the ENBD/PTBD group) obtained with the meta-analysis was of 0.19 (95% CI -0.43 - 0.81), p=0.549 using the model with random effects.

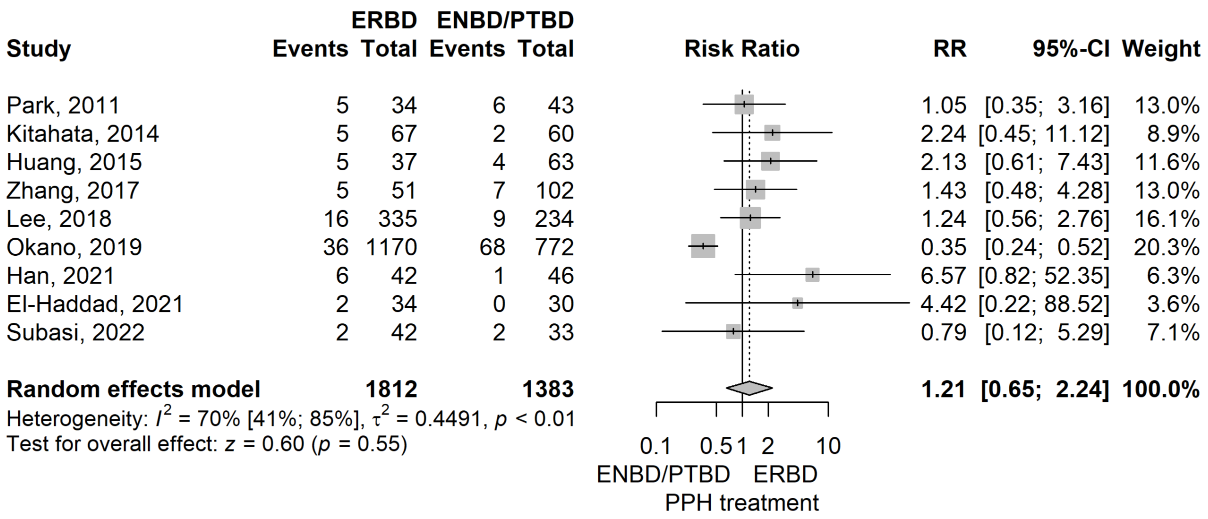

Fig. Forest plot for PPH treatment, comparing ERBD with ENBD/PTBD

Meta-analysis for Chyle leak treatment, comparing ERBD with ENBD/PTBD

No studies to combine for this characteristic

Meta-analysis for POBF treatment, comparing ERBD with ENBD/PTBD

	datele.Year	datele.Study.name	numberCasesPerTreatment	numberCasesTreatment	numberCasesPerControl	numberCasesControl
1	2011	Park, 2011	0	34	2	43
2	2014	Kitahata, 2014	1	67	1	60
5	2015	Huang, 2015	2	37	4	63
6	2017	Zhang, 2017	1	51	2	102
7	2018	Lee, 2018	5	335	4	234
12	2021	El-Haddad, 2021	5	34	1	30

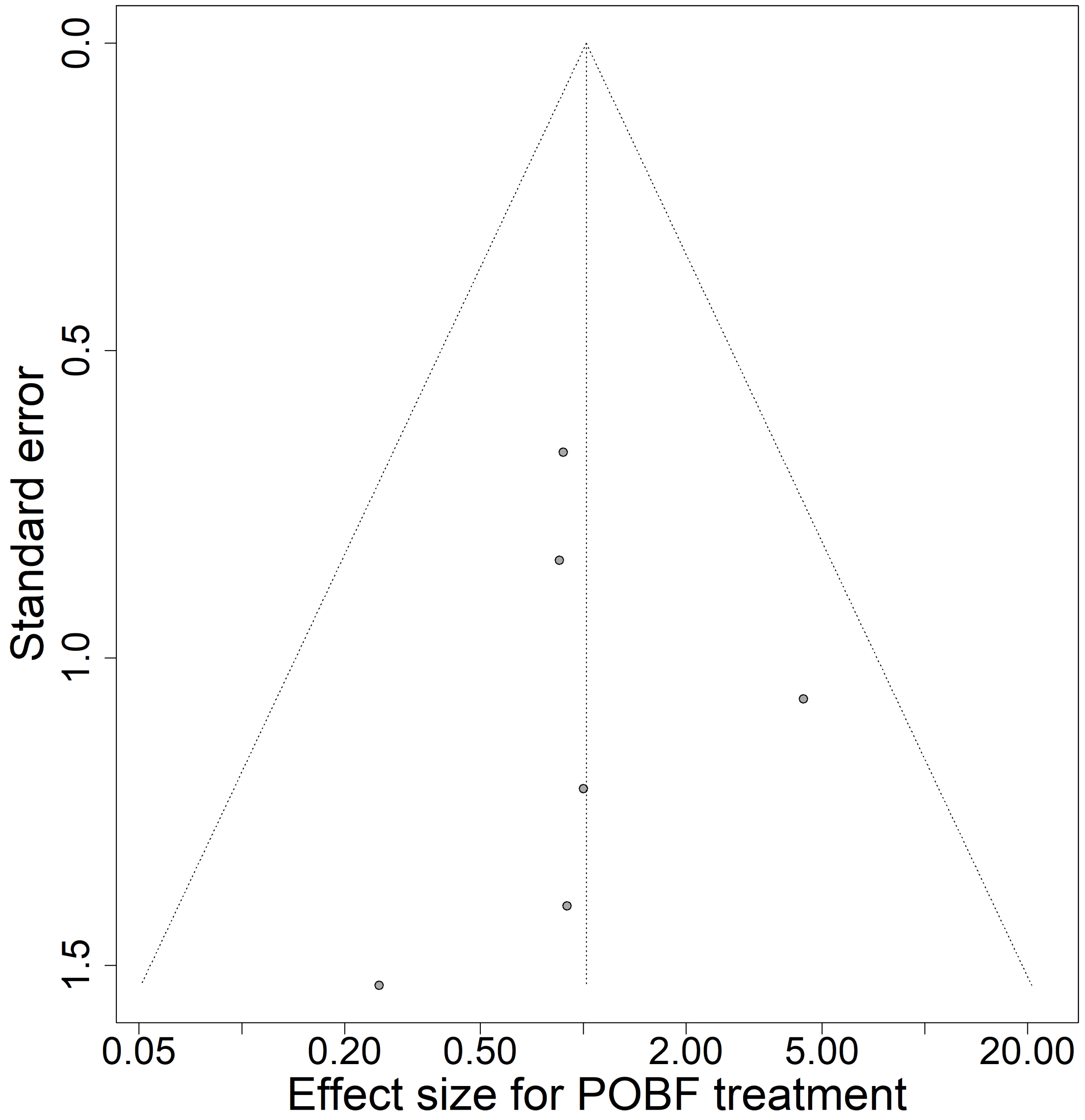

Fig. Funnel plot for POBF treatment, comparing ERBD with ENBD/PTBD

The funnel plot for POBF treatment, comparing ERBD with ENBD/PTBD is shown in figure _.

The publication bias test gave a p=0.872.

Influence studies: Omitting Park, 2011; Omitting Kitahata, 2014; Omitting Huang, 2015; Omitting Zhang, 2017; Omitting Lee, 2018; Omitting El-Haddad, 2021 - no; no; no; no; no; no

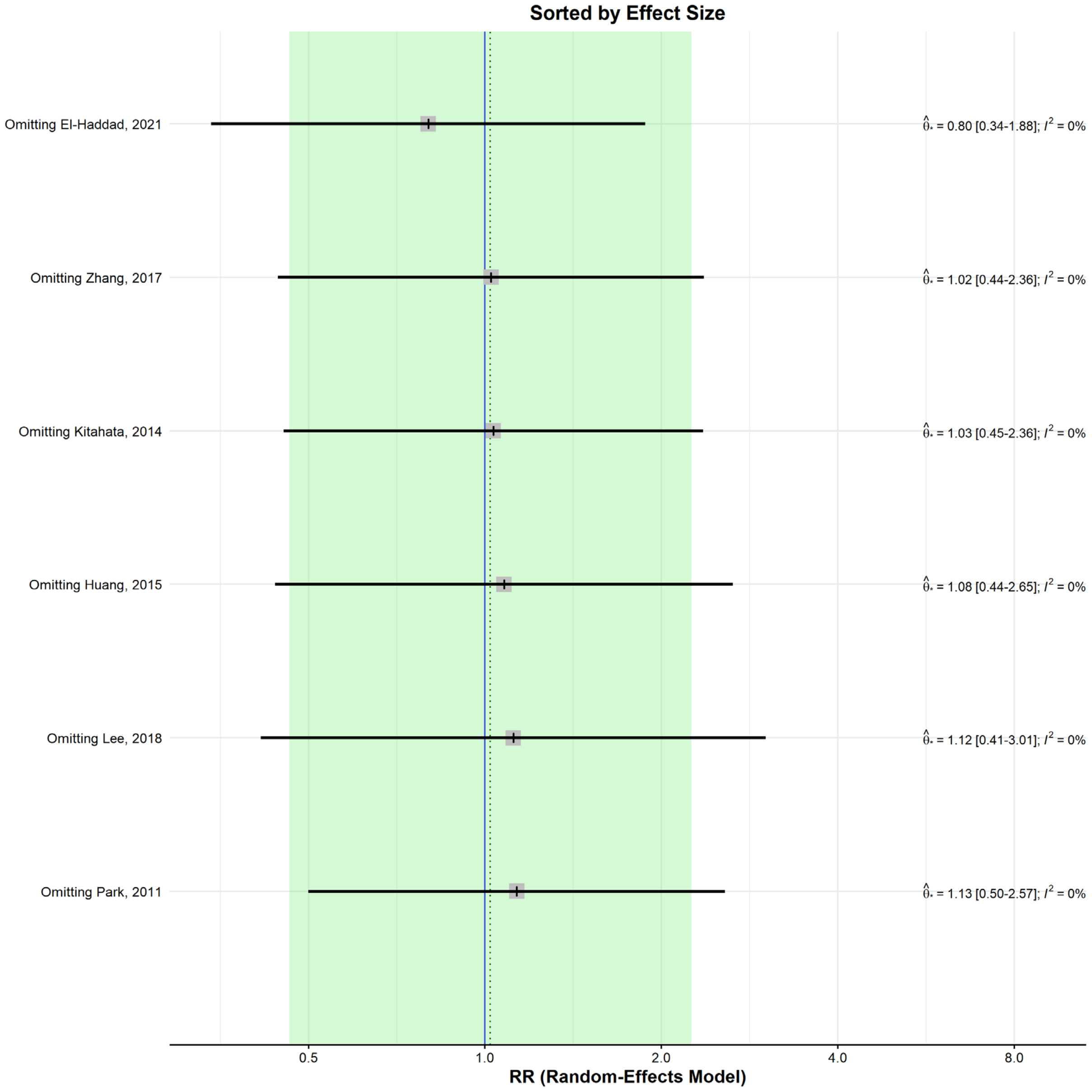

Fig. Leave-one-out sensitivity analysis plot for selected studies for POBF treatment

The heterogeneity was assessed, and we found an I2 of 0% (95% CI 0% - 74.6%) and the Q test for heterogeneity gave p=0.727.

The RR value (the RR of POBF treatment in the ERBD group compared to the ENBD/PTBD group) obtained with the meta-analysis was of 0.02 (95% CI -0.77 - 0.81), p=0.959 using the model with random effects.

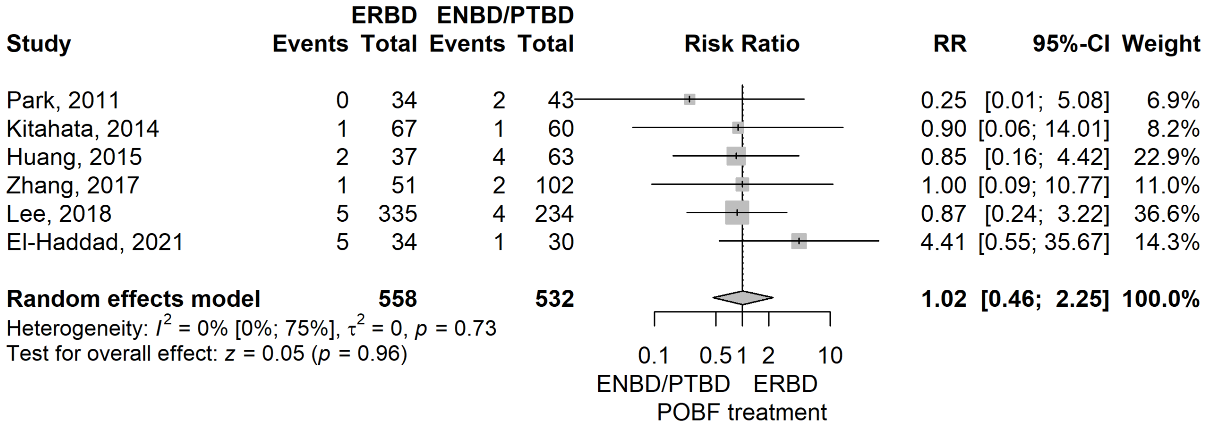

Fig. Forest plot for POBF treatment, comparing ERBD with ENBD/PTBD

Meta-analysis for POPF treatment, comparing ERBD with ENBD/PTBD

	datele.Year	datele.Study.name	numberCasesPerTreatment	numberCasesTreatment	numberCasesPerControl	numberCasesControl
1	2011	Park, 2011	2	34	1	43
2	2014	Kitahata, 2014	26	67	13	60
3	2015	Fujii, 2015	26	72	8	50
4	2015	Uemura, 2015	36	407	20	166
5	2015	Huang, 2015	20	37	19	63
6	2017	Zhang, 2017	21	51	34	102
7	2018	Lee, 2018	72	335	27	234
9	2019	Okano, 2019	284	1170	450	772
10	2021	Han, 2021	16	42	5	46
12	2021	El-Haddad, 2021	9	34	6	30
14	2022	Satoh, 2022	62	117	14	39
15	2022	Subasi, 2022	17	42	18	33

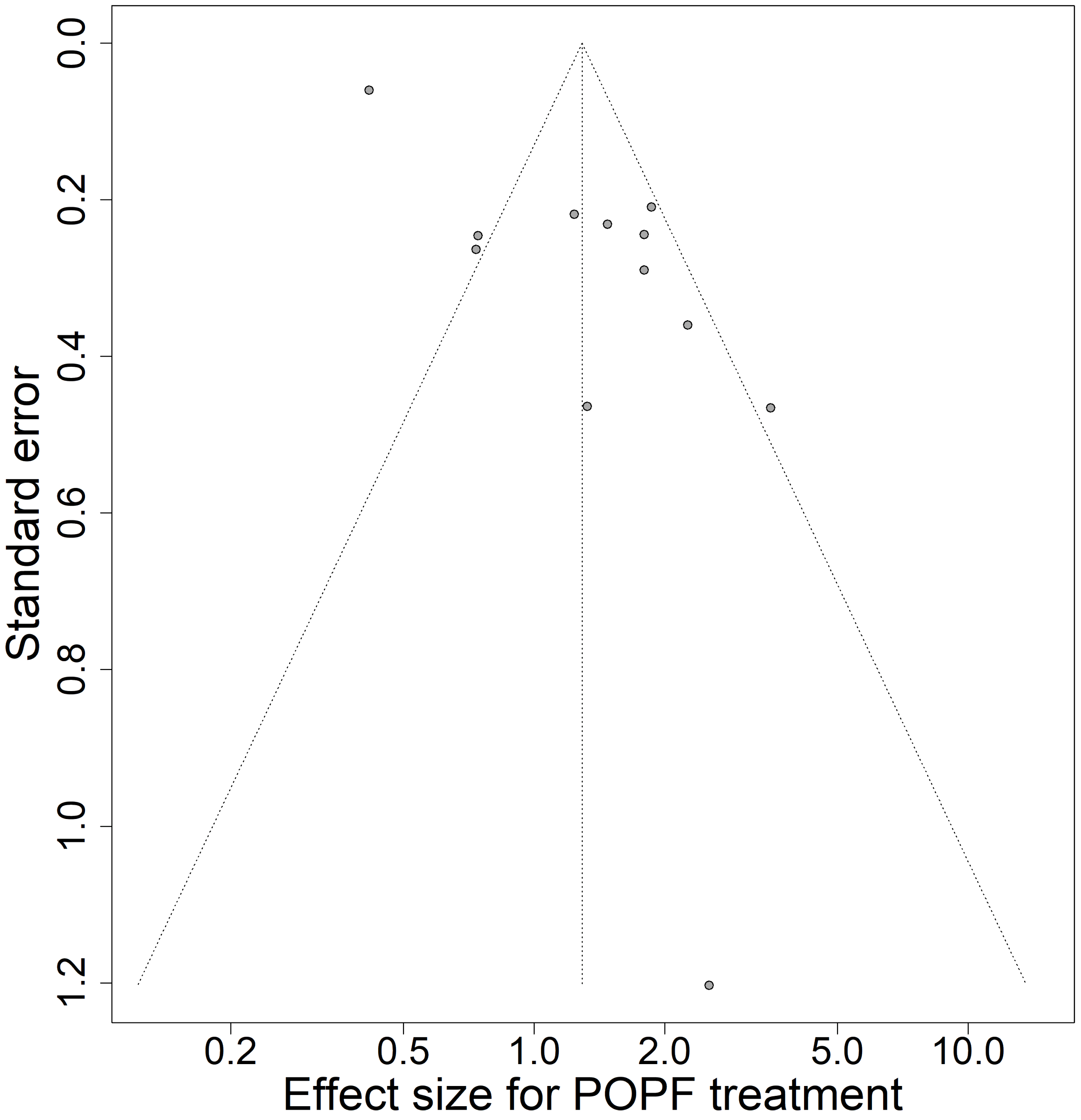

Fig. Funnel plot for POPF treatment, comparing ERBD with ENBD/PTBD

The funnel plot for POPF treatment, comparing ERBD with ENBD/PTBD is shown in figure _.

The publication bias test gave a p=< 0.001.

Influence studies: Omitting Park, 2011; Omitting Kitahata, 2014; Omitting Fujii, 2015; Omitting Uemura, 2015; Omitting Huang, 2015; Omitting Zhang, 2017; Omitting Lee, 2018; Omitting Okano, 2019; Omitting Han, 2021; Omitting El-Haddad, 2021; Omitting Satoh, 2022; Omitting Subasi, 2022 - no; no; no; no; no; no; no; yes; no; no; no; no

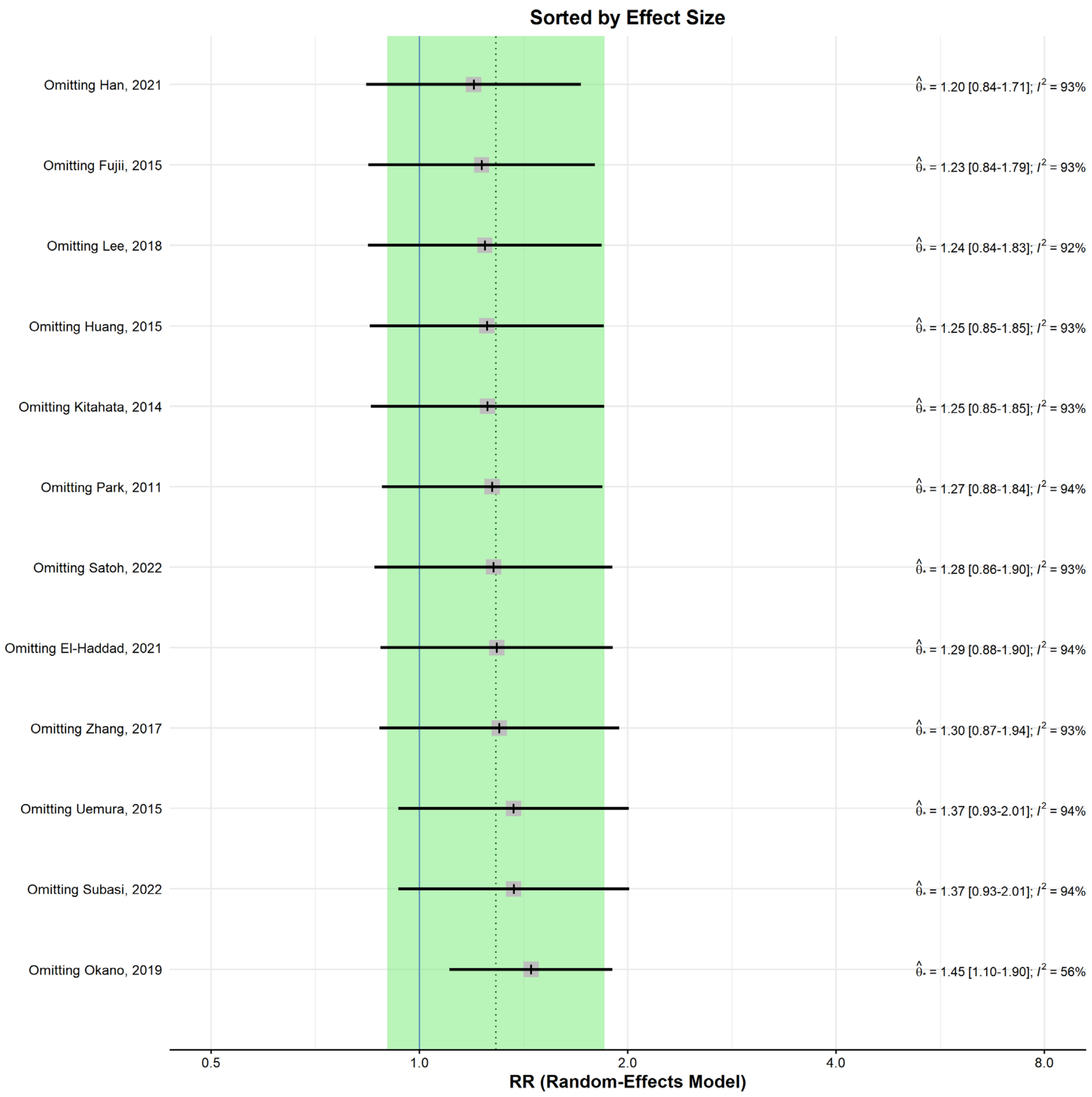

Fig. Leave-one-out sensitivity analysis plot for selected studies for POPF treatment

The heterogeneity was assessed, and we found an I2 of 93.1% (95% CI 89.8% - 95.4%) and the Q test for heterogeneity gave p=< 0.001.

The RR value (the RR of POPF treatment in the ERBD group compared to the ENBD/PTBD group) obtained with the meta-analysis was of 0.25 (95% CI -0.11 - 0.62), p=0.168 using the model with random effects.

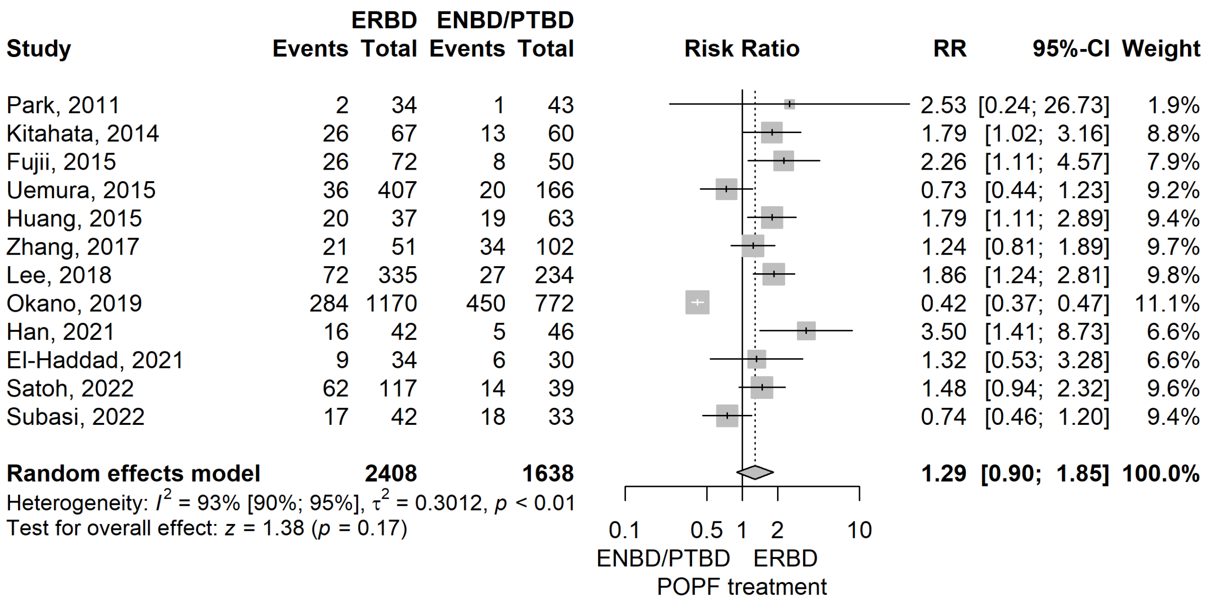

Fig. Forest plot for POPF treatment, comparing ERBD with ENBD/PTBD

Subgroup analyses

Algorithm did not converge for subgroup analisis of

Algorithm did not converge for subgroup analisis of

Meta-analysis for Grade 1 treatment, comparing ERBD with ENBD/PTBD

	datele.Year	datele.Study.name	numberCasesPerTreatment	numberCasesTreatment	numberCasesPerControl	numberCasesControl
2	2014	Kitahata, 2014	12	67	6	60
5	2015	Huang, 2015	10	37	14	63
6	2017	Zhang, 2017	13	51	14	102
9	2019	Okano, 2019	120	1170	173	772
12	2021	El-Haddad, 2021	5	34	4	30
14	2022	Satoh, 2022	16	117	10	39
15	2022	Subasi, 2022	8	42	5	33

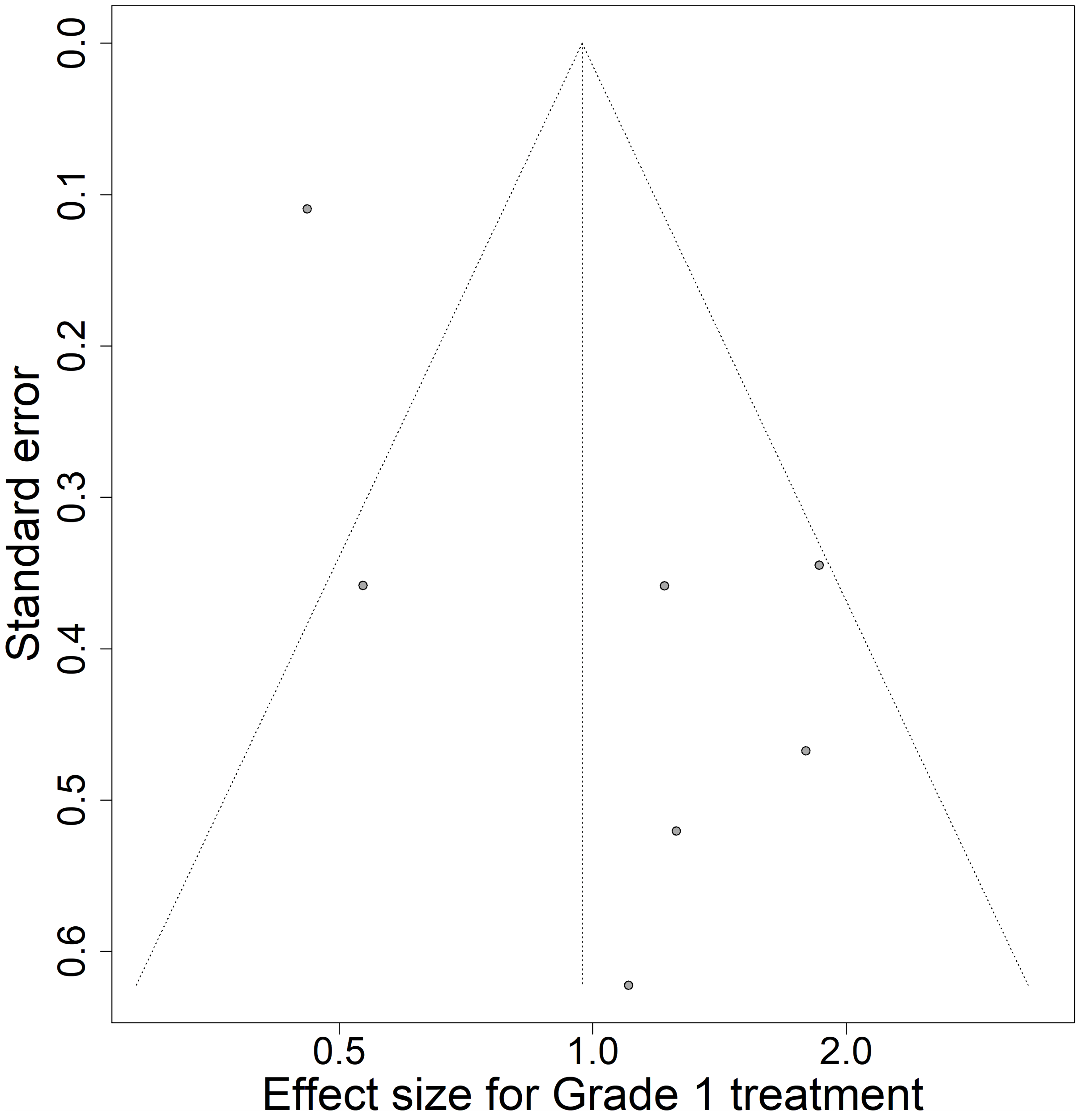

Fig. Funnel plot for Grade 1 treatment, comparing ERBD with ENBD/PTBD

The funnel plot for Grade 1 treatment, comparing ERBD with ENBD/PTBD is shown in figure _.

The publication bias test gave a p=0.019.

Influence studies: Omitting Kitahata, 2014; Omitting Huang, 2015; Omitting Zhang, 2017; Omitting Okano, 2019; Omitting El-Haddad, 2021; Omitting Satoh, 2022; Omitting Subasi, 2022 - no; no; no; yes; no; no; no

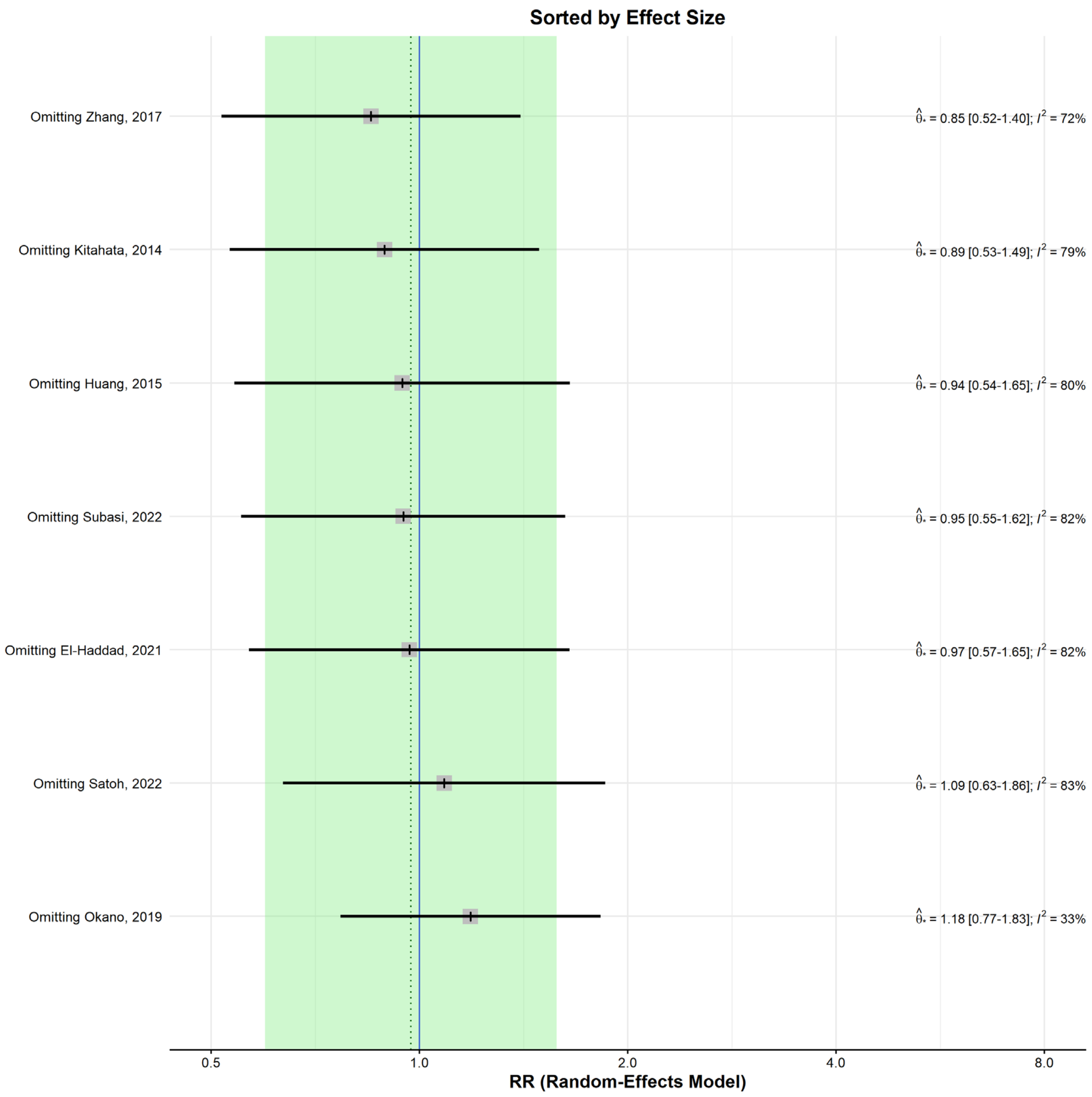

Fig. Leave-one-out sensitivity analysis plot for selected studies for Grade 1 treatment

The heterogeneity was assessed, and we found an I2 of 79.5% (95% CI 58.1% - 90%) and the Q test for heterogeneity gave p=< 0.001.

The RR value (the RR of Grade 1 treatment in the ERBD group compared to the ENBD/PTBD group) obtained with the meta-analysis was of -0.03 (95% CI -0.51 - 0.46), p=0.907 using the model with random effects.

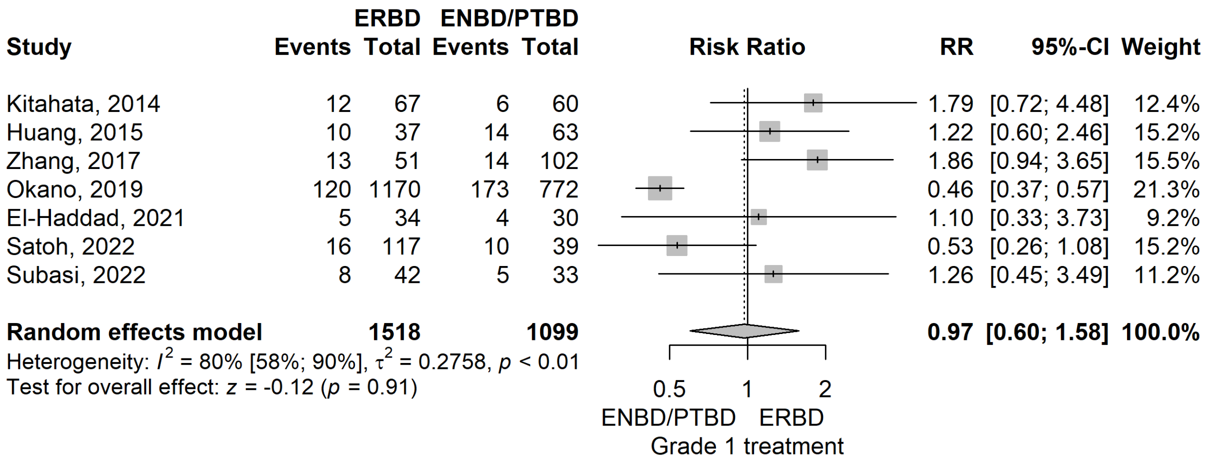

Fig. Forest plot for Grade 1 treatment, comparing ERBD with ENBD/PTBD

Meta-analysis for Grade 2-3 treatment, comparing ERBD with ENBD/PTBD

	datele.Year	datele.Study.name	numberCasesPerTreatment	numberCasesTreatment	numberCasesPerControl	numberCasesControl
2	2014	Kitahata, 2014	14	67	7	60
3	2015	Fujii, 2015	26	72	8	50
4	2015	Uemura, 2015	36	407	20	166
5	2015	Huang, 2015	10	37	5	63
6	2017	Zhang, 2017	8	51	20	102
9	2019	Okano, 2019	164	1170	277	772
12	2021	El-Haddad, 2021	4	34	2	30
14	2022	Satoh, 2022	46	117	4	39
15	2022	Subasi, 2022	9	42	13	33

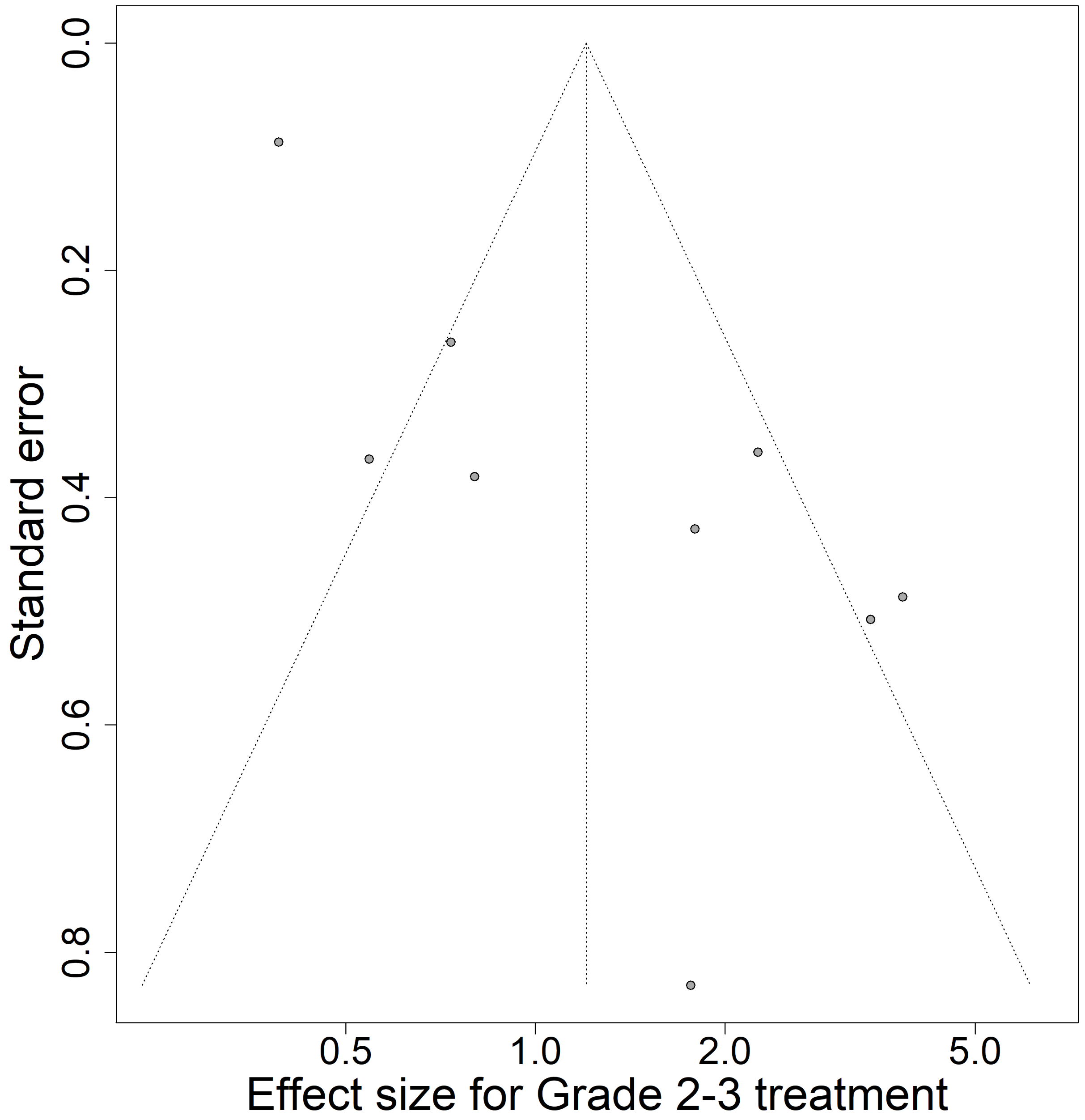

Fig. Funnel plot for Grade 2-3 treatment, comparing ERBD with ENBD/PTBD

The funnel plot for Grade 2-3 treatment, comparing ERBD with ENBD/PTBD is shown in figure _.

The publication bias test gave a p=0.002.

Influence studies: Omitting Kitahata, 2014; Omitting Fujii, 2015; Omitting Uemura, 2015; Omitting Huang, 2015; Omitting Zhang, 2017; Omitting Okano, 2019; Omitting El-Haddad, 2021; Omitting Satoh, 2022; Omitting Subasi, 2022 - no; no; no; no; no; no; no; no; no

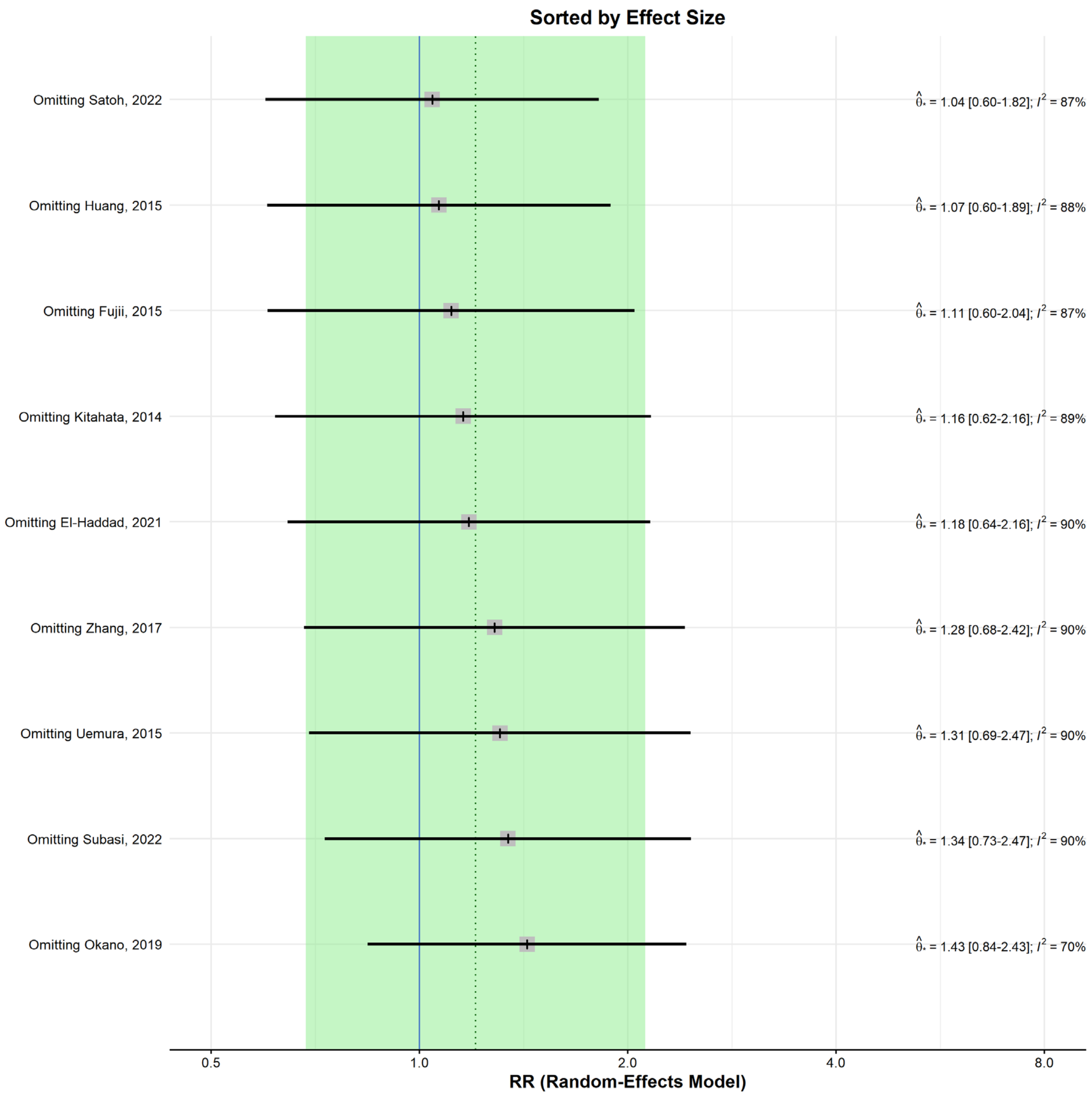

Fig. Leave-one-out sensitivity analysis plot for selected studies for Grade 2-3 treatment

The heterogeneity was assessed, and we found an I2 of 88.8% (95% CI 80.9% - 93.4%) and the Q test for heterogeneity gave p=< 0.001.

The RR value (the RR of Grade 2-3 treatment in the ERBD group compared to the ENBD/PTBD group) obtained with the meta-analysis was of 0.19 (95% CI -0.38 - 0.75), p=0.519 using the model with random effects.

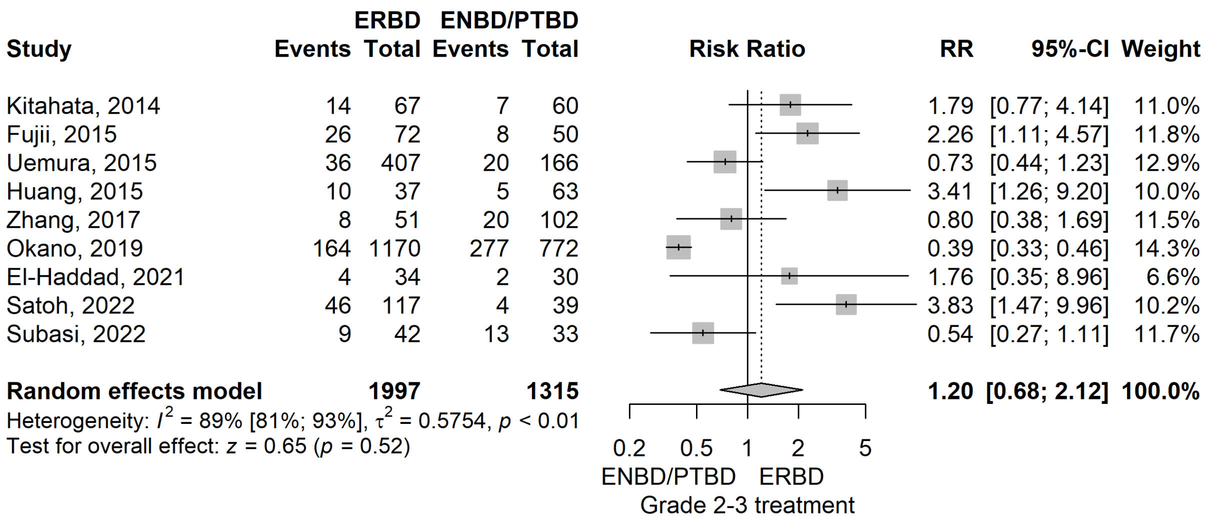

Fig. Forest plot for Grade 2-3 treatment, comparing ERBD with ENBD/PTBD

Meta-analysis for DGE treatment, comparing ERBD with ENBD/PTBD

	datele.Year	datele.Study.name	numberCasesPerTreatment	numberCasesTreatment	numberCasesPerControl	numberCasesControl
1	2011	Park, 2011	6	34	2	43
2	2014	Kitahata, 2014	7	67	4	60
5	2015	Huang, 2015	11	37	4	63
6	2017	Zhang, 2017	12	51	25	102
7	2018	Lee, 2018	29	335	8	234
9	2019	Okano, 2019	97	1170	16	772
10	2021	Han, 2021	3	42	2	46
12	2021	El-Haddad, 2021	3	34	5	30
14	2022	Satoh, 2022	20	117	3	39

2

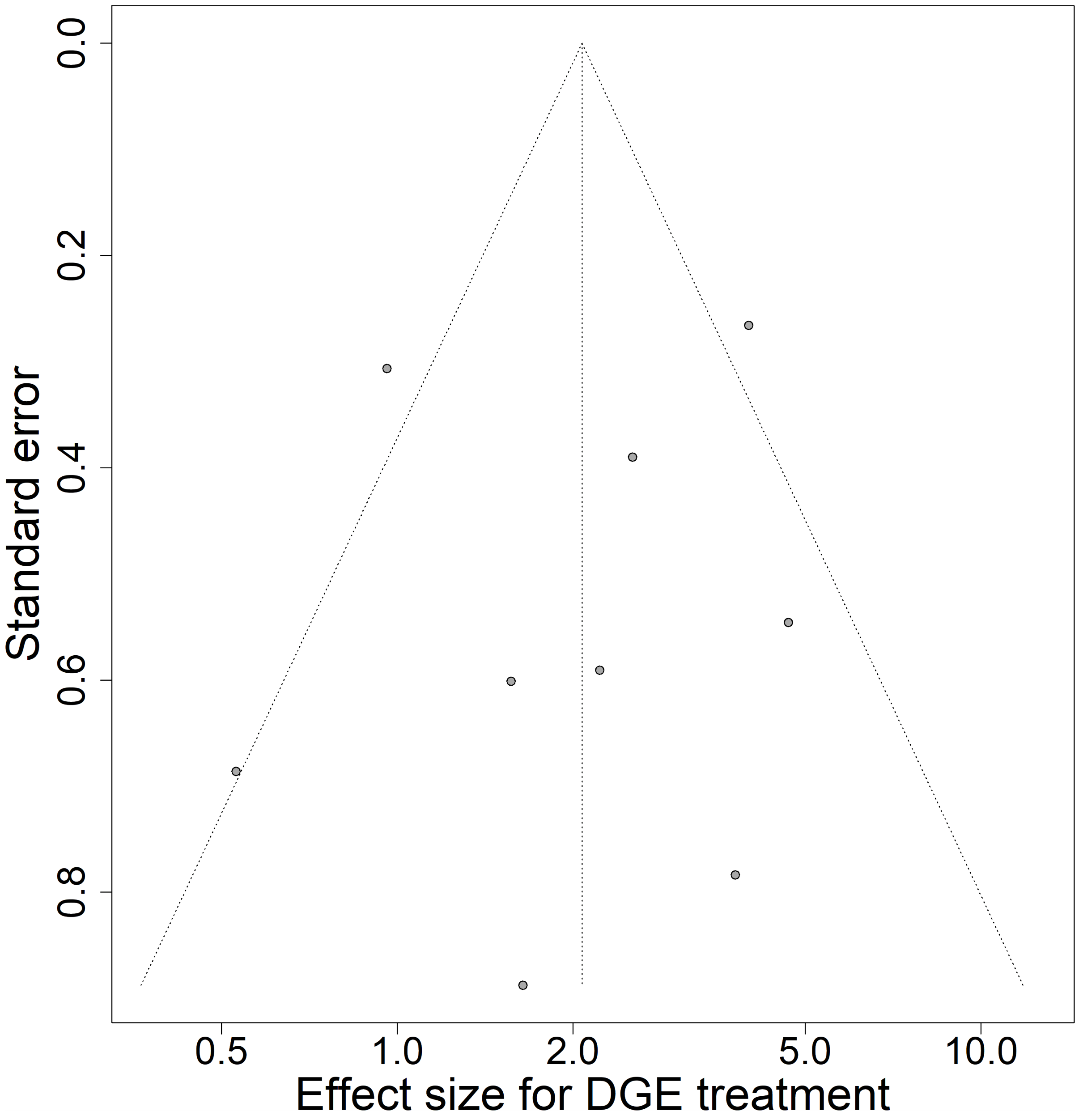

Fig. Funnel plot for DGE treatment, comparing ERBD with ENBD/PTBD

The funnel plot for DGE treatment, comparing ERBD with ENBD/PTBD is shown in figure _.

The publication bias test gave a p=0.677.

Influence studies: Omitting Park, 2011; Omitting Kitahata, 2014; Omitting Huang, 2015; Omitting Zhang, 2017; Omitting Lee, 2018; Omitting Okano, 2019; Omitting Han, 2021; Omitting El-Haddad, 2021; Omitting Satoh, 2022 - no; no; no; yes; no; no; no; no; no

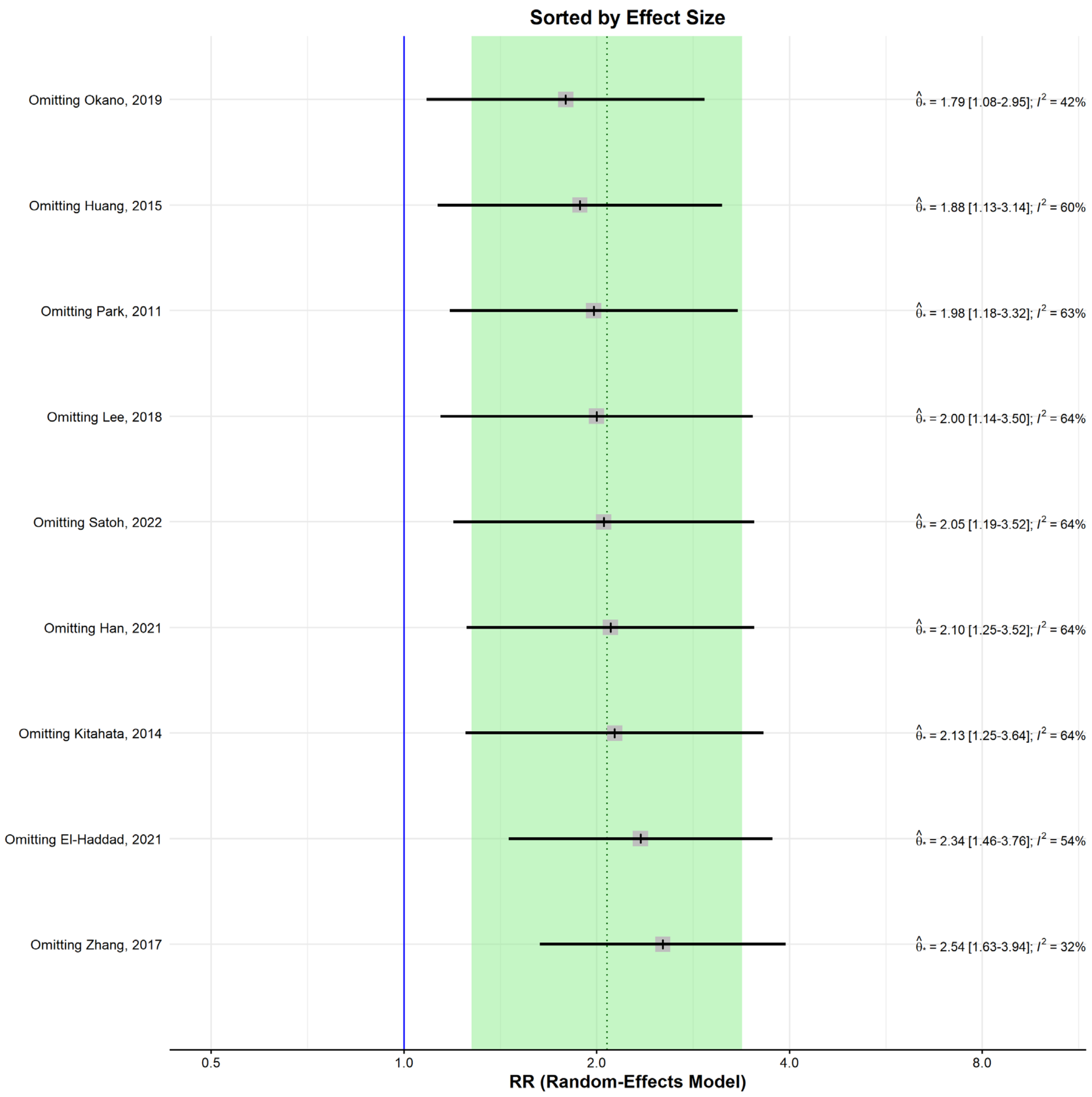

Fig. Leave-one-out sensitivity analysis plot for selected studies for DGE treatment

The heterogeneity was assessed, and we found an I2 of 59.3% (95% CI 15.1% - 80.5%) and the Q test for heterogeneity gave p=0.012.

The RR value (the RR of DGE treatment in the ERBD group compared to the ENBD/PTBD group) obtained with the meta-analysis was of 0.73 (95% CI 0.24 - 1.22), p=0.003 using the model with random effects.

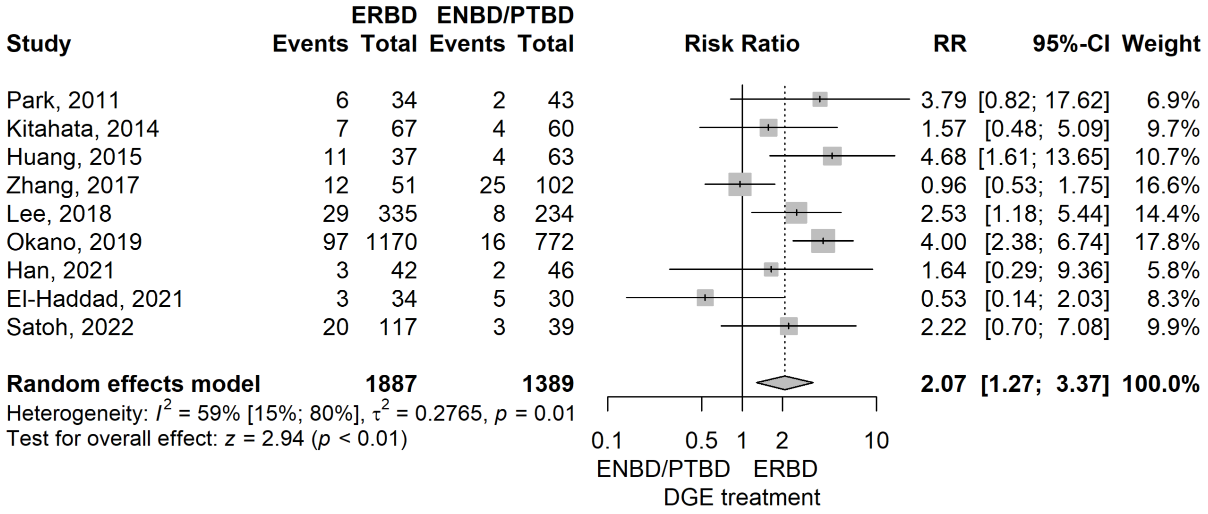

Fig. Forest plot for DGE treatment, comparing ERBD with ENBD/PTBD

Meta-analysis for Soft pancreas treatment, comparing ERBD with ENBD/PTBD

	datele.Year	datele.Study.name	numberCasesPerTreatment	numberCasesTreatment	numberCasesPerControl	numberCasesControl
2	2014	Kitahata, 2014	35	67	31	60
3	2015	Fujii, 2015	21	72	19	50
6	2017	Zhang, 2017	22	51	56	102
12	2021	El-Haddad, 2021	7	34	14	30
13	2021	Suenaga, 2021	13	40	20	38
14	2022	Satoh, 2022	73	117	26	39
15	2022	Subasi, 2022	14	42	13	33

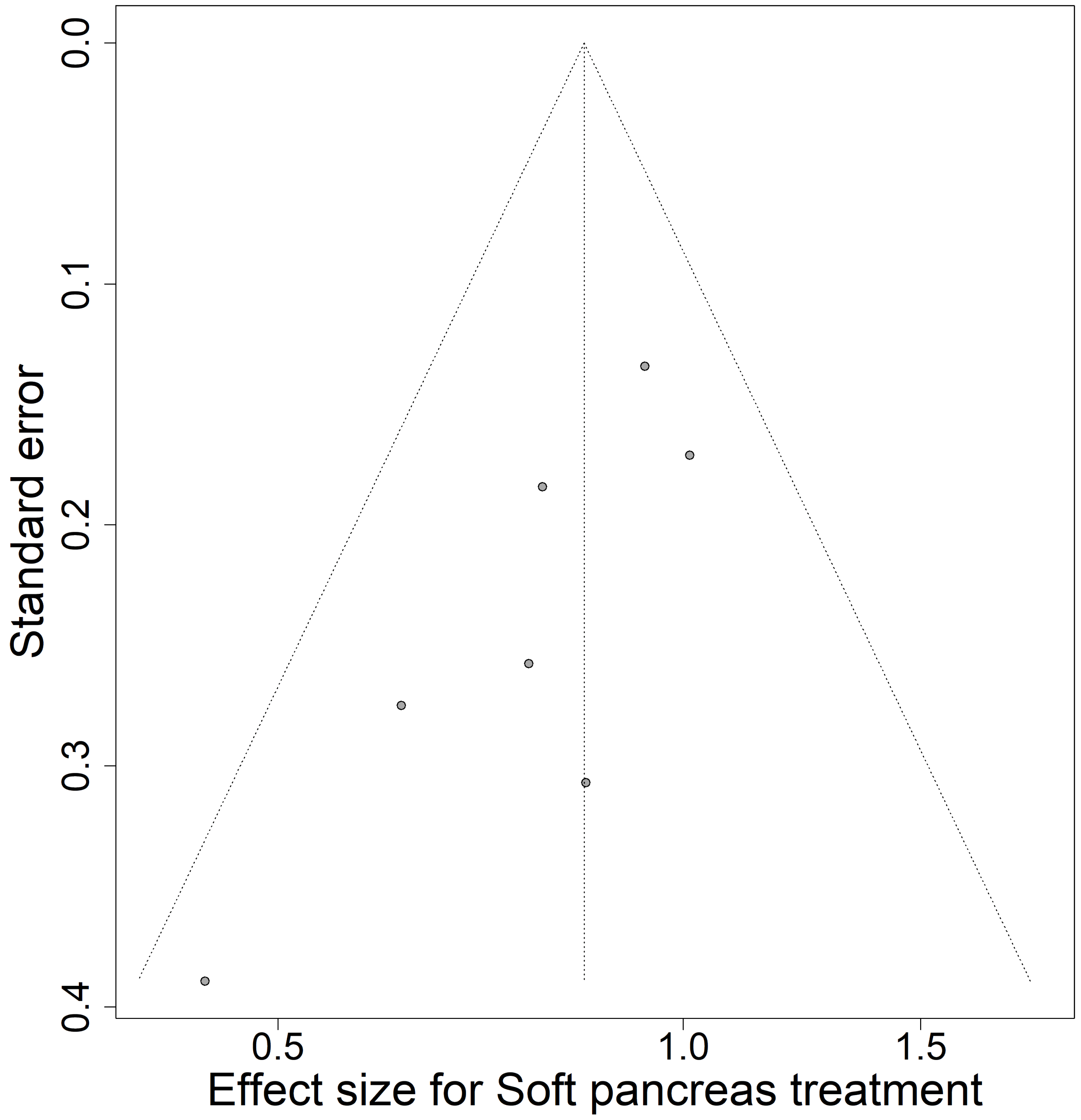

Fig. Funnel plot for Soft pancreas treatment, comparing ERBD with ENBD/PTBD

The funnel plot for Soft pancreas treatment, comparing ERBD with ENBD/PTBD is shown in figure _.

The publication bias test gave a p=0.029.

Influence studies: Omitting Kitahata, 2014; Omitting Fujii, 2015; Omitting Zhang, 2017; Omitting El-Haddad, 2021; Omitting Suenaga, 2021; Omitting Satoh, 2022; Omitting Subasi, 2022 - no; no; no; no; no; yes; no

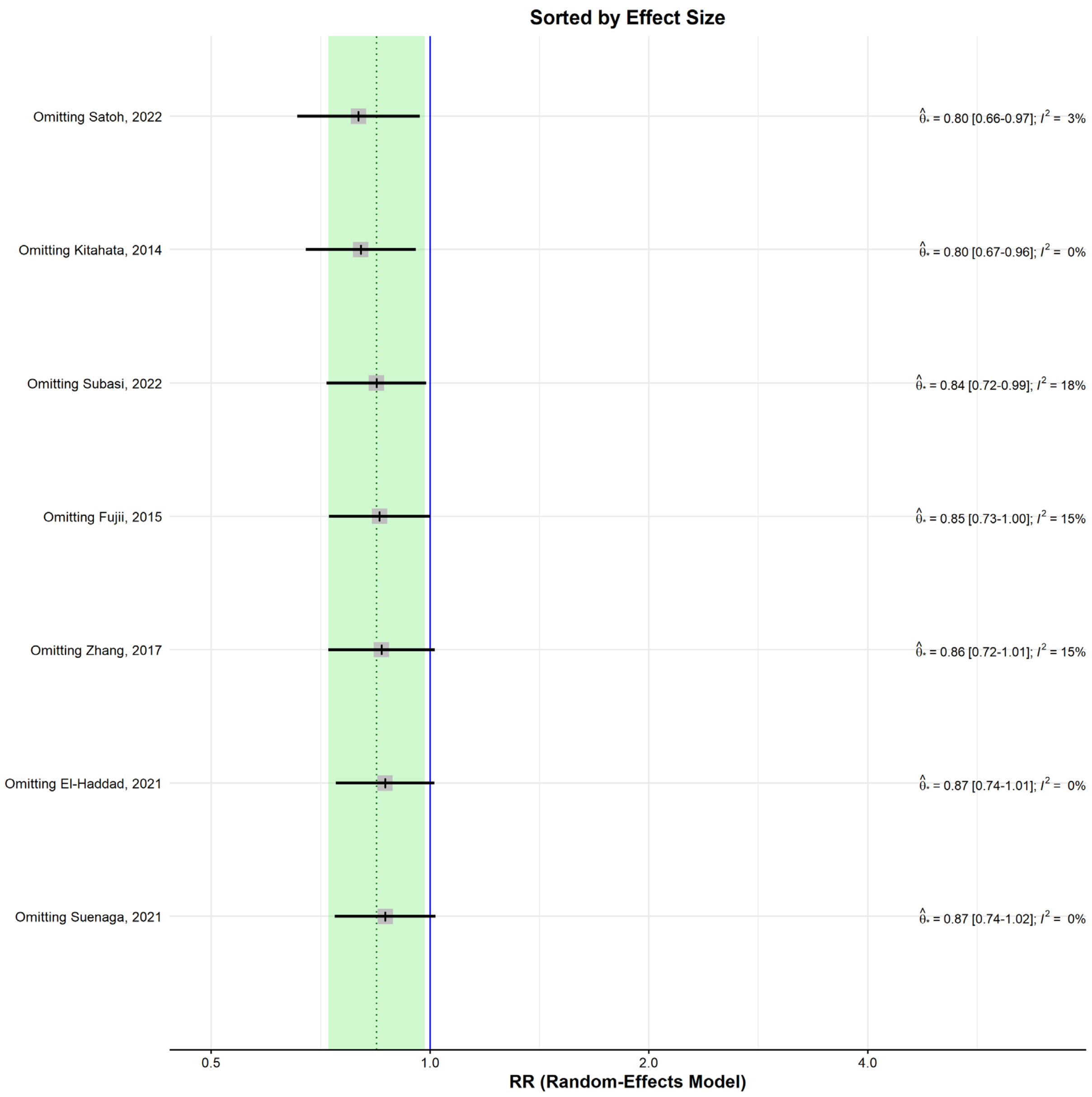

Fig. Leave-one-out sensitivity analysis plot for selected studies for Soft pancreas treatment

The heterogeneity was assessed, and we found an I2 of 1.1% (95% CI 0% - 71.1%) and the Q test for heterogeneity gave p=0.416.

The RR value (the RR of Soft pancreas treatment in the ERBD group compared to the ENBD/PTBD group) obtained with the meta-analysis was of -0.17 (95% CI -0.32 - -0.02), p=0.029 using the model with random effects.

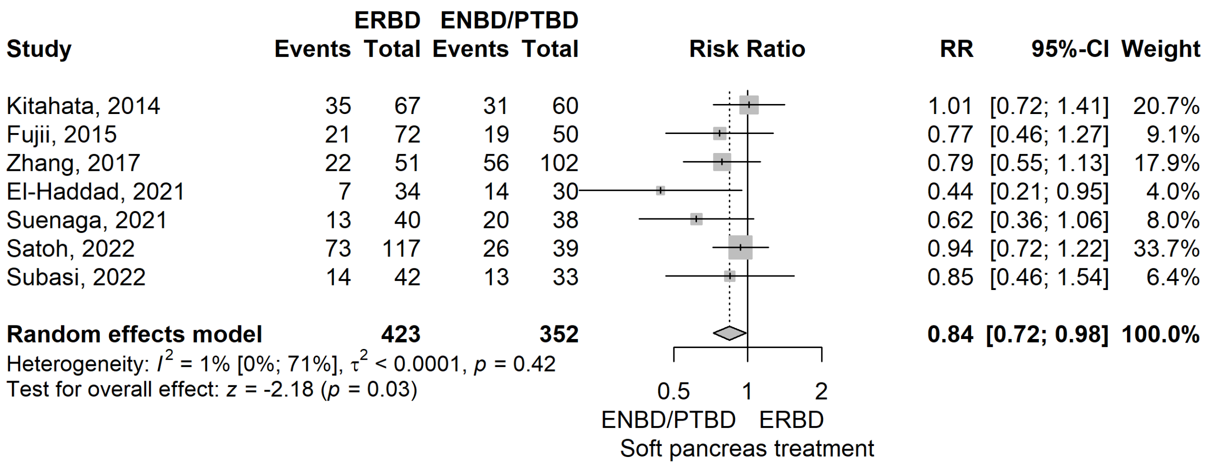

Fig. Forest plot for Soft pancreas treatment, comparing ERBD with ENBD/PTBD

Sinteza metaanalizelor

Characteristic, effect size type	N studies	e/nt intervention	e/nt control	Effect size (95% CI)	p-value	I2 (95% CI)	p-value	Egger test	Studies	Leave one out
Pancreatitis treatment, RR	5	34/196	23/276	0.8 (0.31 - 1.28)	0.001	0 (0 - 79.2)	0.735	0.533	Park, 2011; Huang, 2015; Zhang, 2017; El-Haddad, 2021; Suenaga, 2021	- Park, 2011: 1.77 (0.95-3.31), p=0.073, I2=0%; - Huang, 201: 2.23 (1.37-3.65), p=0.001, I2=0%; - Zhang, 201: 2.28 (1.08-4.81), p=0.031, I2=0%; - El-Haddad,: 2.21 (1.35-3.59), p=0.001, I2=0%;

									- Suenaga, 2: 2.43 (1.46-4.04), p=< 0.001, I2=0%
Cholangitis treatment, RR	7	119/418	58/382	0.41 (-0.2 - 1.03)	0.19	69.4 (32.6 - 86.1)	0.003	0.276	Kitahata, 2014; Fujii, 2015; Huang, 2015; Zhang, 2017; El-Haddad, 2021; Suenaga, 2021; Satoh, 2022	- Kitahata, 2014: 1.29 (0.74-2.26), p=0.367, I2=64%; - Fujii, 2015: 1.38 (0.68-2.82), p=0.373, I2=69%; - Huang, 2015: 1.55 (0.71-3.36), p=0.267, I2=74%; - Zhang, 2017: 1.32 (0.68-2.55), p=0.409, I2=65%; - El-Haddad, 202: 1.66 (0.85-3.26), p=0.139, I2=74%; - Suenaga, 2021: 1.8

										(0.98-3.29), p=0.057, I2=68%;<br>- Satoh, 2022: 1.73 (0.82-3.69), p=0.152, I2=68%
Perforation treatment, RR	0	-	-	-	-	-	-	-	-	-
Hemorrhage treatment, RR	2	1/68	1/73	0.06 (-2.18 - 2.29)	0.961	0 (NA - NA)	0.42	NC	Park, 2011; El-Haddad, 2021	- Park, 2011: 2.65 (0.11-62.72), p=0.546, I2=NA%;<br>- El-Haddad,: 0.42 (0.02-10), p=0.592, I2=NA%
Occlusion treatment, RR	3	13/122	17/208	0.82 (-1.09 - 2.72)	0.401	64.9 (0 - 89.9)	0.058	0.124	Park, 2011; Huang, 2015; Zhang, 2017	- Park, 2011: 1.02 (0.23-4.57), p=0.983, I2=41%;<br>- Huang, 201: 2.4 (0.1-56.22), p=0.585, I2=78%;<br>- Zhang, 201: 6.48 (1.05-39.92), p=0.044, I2=0%

Catheter exchange treatment, RR	3	81/191	44/120	-0.2 (-0.47 - 0.08)	0.157	69 (0 - 91)	0.04	0.296	Park, 2011; Suenaga, 2021; Satoh, 2022	- Park, 2011: 0.79 (0.6-1.04), p=0.099, I2=0%; - Suenaga, 2: 3.55 (0.13-97.54), p=0.453, I2=82%; - Satoh, 202: 3.28 (0.1-110.31), p=0.508, I2=84%
Mortality treatment, RR	11	45/2062	46/1426	0 (-0.71 - 0.7)	0.99	43.2 (0 - 72.8)	0.07	0.031	Park, 2011; Kitahata, 2014; Fujii, 2015; Uemura, 2015; Huang, 2015; Zhang, 2017; Okano, 2019; Han, 2021; Byun, 2021; El-Haddad, 2021; Subasi, 2022	- Park, 2011: 1.12 (0.53-2.37), p=0.775, I2=48%; - Kitahata, : 0.92 (0.44-1.9), p=0.822, I2=44%; - Fujii, 201: 1 (0.49-2.01), p=0.99, I2=43%; - Uemura, 20: 0.86 (0.42-1.78), p=0.684, I2=37%; - Huang,

										201: 0.89 (0.44-1.77), p=0.731, I2=39%; - Zhang, 201: 1 (0.45-2.22), p=0.998, I2=47%; - Okano, 201: 1.33 (0.7-2.53), p=0.387, I2=0%; - Han, 2021: 0.9 (0.44-1.84), p=0.77, I2=42%; - Byun, 2021: 1.11 (0.52-2.33), p=0.792, I2=48%; - El-Haddad,: 0.92 (0.44-1.9), p=0.822, I2=44%
Reoperation treatment, RR	4	9/202	14/241	-0.37 (-1.5 - 0.75)	0.514	34.2 (0 - 78.5)	0.219	0.871	Kitahata, 2014; Zhang, 2017; Han, 2021; Subasi, 2022	- Kitahata, 2014: 0.69 (0.22-2.11), p=0.514, I2=34%; - Zhang,

									2017: 0.64 (0.14-2.86), p=0.554, I2=66%; - Han, 2021: 0.38 (0.13-1.16), p=0.089, I2=0%
Overall complication rate (Clavien - Dindo) treatment, RR	11	996/2351	981/1604	0.17 (-0.1 - 0.44)	0.21	97.5 (96.5 - 98.1)	< 0.001	0.399	Park, 2011; Kitahata, 2014; Uemura, 2015; Huang, 2015; Zhang, 2017; Lee, 2018; Mori, 2019; Okano, 2019; Han, 2021; Byun, 2021; Subasi, 2022	- Park, 2011: 1.16 (0.87-1.54), p=0.321, I2=98%; - Kitahata, : 1.15 (0.87-1.53), p=0.33, I2=98%; - Uemura, 20: 1.23 (0.92-1.65), p=0.165, I2=98%; - Huang, 201: 1.16 (0.87-1.55), p=0.315, I2=98%; - Zhang, 201: 1.2 (0.89-1.62), p=0.234, I2=98%; - Lee, 2018:

										1.16 (0.87-1.55), p=0.322, I2=98%; - Mori, 2019: 1.22 (0.9-1.64), p=0.199, I2=98%; - Okano, 201: 1.28 (1.08-1.53), p=0.005, I2=79%; - Han, 2021: 1.13 (0.86-1.48), p=0.375, I2=98%; - Byun, 2021: 1.16 (0.87-1.55), p=0.312, I2=98%; - Subasi, 20: 1.22 (0.9-1.64), p=0.199, I2=98%
Grade 1-2 treatment, RR	6	426/1427	470/1054	-0.01 (-0.35 - 0.34)	0.972	90 (81 - 94.8)	< 0.001	0.071	Kitahata, 2014; Huang, 2015; Zhang, 2017; Mori, 2019; Okano, 2019;	- Kitahata, 2014: 1 (0.7-1.42), p=0.991, I2=92%; - Huang, 2015: 0.96

									Subasi, 2022	(0.64-1.43), p=0.824, I2=91%; - Zhang, 2017: 0.98 (0.65-1.49), p=0.932, I2=91%; - Mori, 2019: 0.94 (0.63-1.41), p=0.777, I2=89%; - Okano, 2019: 1.21 (1.01-1.45), p=0.044, I2=0%; - Subasi, 2022: 0.94 (0.63-1.41), p=0.77, I2=89%
Grade >=3 treatment, RR	7	285/1834	378/1220	-0.12 (-0.69 - 0.45)	0.69	92.5 (87 - 95.6)	< 0.001	0.031	Kitahata, 2014; Uemura, 2015; Huang, 2015; Zhang, 2017; Mori, 2019; Okano, 2019; Subasi, 2022	- Kitahata, 2014: 0.78 (0.43-1.4), p=0.401, I2=91%; - Uemura, 2015: 0.91 (0.45-1.8), p=0.779, I2=92%; - Huang,

										2015: 0.76 (0.44-1.31), p=0.322, I2=93%; - Zhang, 2017: 0.85 (0.44-1.65), p=0.629, I2=93%; - Mori, 2019: 0.96 (0.5-1.85), p=0.905, I2=94%; - Okano, 2019: 1.09 (0.68-1.72), p=0.728, I2=67%; - Subasi, 2022: 0.96 (0.49-1.85), p=0.892, I2=94%
Infectious complications treatment, RR	5	364/1383	483/950	0.12 (-0.57 - 0.8)	0.733	91.5 (83.1 - 95.7)	< 0.001	0.015	Kitahata, 2014; Fujii, 2015; Okano, 2019; El-Haddad, 2021; Suenaga, 2021	- Kitahata, 2014: 1.05 (0.46-2.42), p=0.905, I2=92%; - Fujii, 2015: 0.87 (0.46-1.63), p=0.659, I2=87%;

										- Okano, 2019: 1.5 (0.86-2.63), p=0.155, I2=60%;<br>- El-Haddad, 202: 1.09 (0.47-2.54), p=0.84, I2=93%;<br>- Suenaga, 2021: 1.24 (0.52-2.93), p=0.624, I2=93%
Sepsis treatment, RR	4	18/293	5/212	0.88 (-0.11 - 1.87)	0.081	0 (0 - 84.7)	0.929	0.227	Kitahata, 2014; Fujii, 2015; Huang, 2015; Satoh, 2022	- Kitahata, 2014: 2.18 (0.72-6.64), p=0.171, I2=0%;<br>- Fujii, 2015: 2.67 (0.74-9.64), p=0.133, I2=0%;<br>- Huang, 2015: 2.75 (0.86-8.73), p=0.087, I2=0%;<br>- Satoh, 2022: 2.23 (0.78-

Intraabdominal abcess treatment, RR	7	100/785	60/523	0.5 (0.2 - 0.8)	0.001	0 (0 - 70.8)	0.433	0.506	Park, 2011; Kitahata, 2014; Fujii, 2015; Uemura, 2015; Huang, 2015; Zhang, 2017; Satoh, 2022	6.41), p=0.137, I2=0% - Park, 2011: 1.67 (1.2-2.33), p=0.002, I2=15%; - Kitahata, : 1.71 (1.25-2.36), p=< 0.001, I2=5%; - Fujii, 201: 1.56 (1.14-2.13), p=0.005, I2=0%; - Uemura, 20: 1.74 (1.27-2.38), p=< 0.001, I2=0%; - Huang, 201: 1.66 (1.21-2.29), p=0.002, I2=15%; - Zhang, 201: 1.56 (1.05-2.32), p=0.026, I2=13%; - Satoh, 202:

										1.58 (1.17-2.15), p=0.003, I2=0%
Wound infections treatment, RR	8	142/1090	81/740	0.25 (-0.08 - 0.58)	0.142	26.8 (0 - 67)	0.215	0.631	Park, 2011; Kitahata, 2014; Uemura, 2015; Huang, 2015; Zhang, 2017; Lee, 2018; Satoh, 2022; Subasi, 2022	- Park, 2011: 1.33 (0.95-1.85), p=0.095, I2=23%; - Kitahata, : 1.23 (0.87-1.73), p=0.24, I2=28%; - Uemura, 20: 1.48 (1.1-1.99), p=0.009, I2=0%; - Huang, 201: 1.27 (0.85-1.9), p=0.236, I2=37%; - Zhang, 201: 1.29 (0.86-1.94), p=0.212, I2=36%; - Lee, 2018: 1.16 (0.78-1.71), p=0.465, I2=24%; - Satoh, 202: 1.2

										(0.84-1.69), p=0.315, I2=23%; - Subasi, 20: 1.31 (0.92-1.89), p=0.138, I2=35%
PPH treatment, RR	9	82/1812	99/1383	0.19 (-0.43 - 0.81)	0.549	70.3 (41 - 85)	< 0.001	0.002	Park, 2011; Kitahata, 2014; Huang, 2015; Zhang, 2017; Lee, 2018; Okano, 2019; Han, 2021; El-Haddad, 2021; Subasi, 2022	- Park, 2011: 1.27 (0.63-2.58), p=0.503, I2=73%; - Kitahata, : 1.14 (0.59-2.2), p=0.688, I2=72%; - Huang, 201: 1.13 (0.58-2.19), p=0.723, I2=70%; - Zhang, 201: 1.21 (0.6-2.43), p=0.593, I2=72%; - Lee, 2018: 1.25 (0.61-2.56), p=0.549, I2=71%; - Okano, 201: 1.51 (0.96-

										2.38), p=0.074, I2=0%; - Han, 2021: 1.06 (0.58-1.94), p=0.859, I2=68%; - El-Haddad,: 1.15 (0.61-2.15), p=0.663, I2=72%; - Subasi, 20: 1.27 (0.65-2.48), p=0.477, I2=74%
Chyle leak treatment, RR	0	-	-	-	-	-	-	-	-	-
POBF treatment, RR	6	14/558	14/532	0.02 (-0.77 - 0.81)	0.959	0 (0 - 74.6)	0.727	0.872	Park, 2011; Kitahata, 2014; Huang, 2015; Zhang, 2017; Lee, 2018; El-Haddad, 2021	- Park, 2011: 1.13 (0.5-2.57), p=0.765, I2=0%; - Kitahata, : 1.03 (0.45-2.36), p=0.938, I2=0%; - Huang, 201: 1.08 (0.44-2.65), p=0.87, I2=0%; - Zhang, 201: 1.02

									(0.44-2.36), p=0.956, I2=0%; - Lee, 2018: 1.12 (0.41-3.01), p=0.826, I2=0%; - El-Haddad,: 0.8 (0.34-1.88), p=0.609, I2=0%
POPF treatment, RR	12	591/2408	615/1638	0.25 (-0.11 - 0.62)	0.168	93.1 (89.8 - 95.4)	< 0.001	< 0.001	Park, 2011; Kitahata, 2014; Fujii, 2015; Uemura, 2015; Huang, 2015; Zhang, 2017; Lee, 2018; Okano, 2019; Han, 2021; El-Haddad, 2021; Satoh, 2022; Subasi, 2022	- Park, 2011: 1.27 (0.88-1.84), p=0.197, I2=94%; - Kitahata,: 1.25 (0.85-1.85), p=0.254, I2=93%; - Fujii, 201: 1.23 (0.84-1.79), p=0.283, I2=93%; - Uemura, 20: 1.37 (0.93-2.01), p=0.11, I2=94%; - Huang, 201: 1.25

(0.85-1.85), p=0.26, I2=93%; - Zhang, 201: 1.3 (0.87-1.94), p=0.192, I2=93%; - Lee, 2018: 1.24 (0.84-1.83), p=0.273, I2=92%; - Okano, 201: 1.45 (1.1-1.9), p=0.007, I2=56%; - Han, 2021: 1.2 (0.84-1.71), p=0.324, I2=93%; - El-Haddad,: 1.29 (0.88-1.9), p=0.193, I2=94%; - Satoh, 202: 1.28 (0.86-1.9), p=0.224, I2=93%; - Subasi, 20: 1.37 (0.93-2.01),

Grade 1 treatment, RR	7	184/1518	226/1099	-0.03 (-0.51 - 0.46)	0.907	79.5 (58.1 - 90)	< 0.001	0.019	Kitahata, 2014; Huang, 2015; Zhang, 2017; Okano, 2019; El-Haddad, 2021; Satoh, 2022; Subasi, 2022	p=0.11, I2=94% - Kitahata, 2014: 0.89 (0.53-1.49), p=0.656, I2=79%; - Huang, 2015: 0.94 (0.54-1.65), p=0.839, I2=80%; - Zhang, 2017: 0.85 (0.52-1.4), p=0.525, I2=72%; - Okano, 2019: 1.18 (0.77-1.83), p=0.443, I2=33%; - El-Haddad, 202: 0.97 (0.57-1.65), p=0.9, I2=82%; - Satoh, 2022: 1.09 (0.63-1.86), p=0.765, I2=83%; - Subasi, 2022: 0.95

Grade 2-3 treatment, RR	9	317/1997	356/1315	0.19 (-0.38 - 0.75)	0.519	88.8 (80.9 - 93.4)	< 0.001	0.002	Kitahata, 2014; Fujii, 2015; Uemura, 2015; Huang, 2015; Zhang, 2017; Okano, 2019; El-Haddad, 2021; Satoh, 2022; Subasi, 2022	(0.55-1.62), p=0.844, I2=82% - Kitahata, 2014: 1.16 (0.62-2.16), p=0.649, I2=89%; - Fujii, 2015: 1.11 (0.6-2.04), p=0.736, I2=87%; - Uemura, 2015: 1.31 (0.69-2.47), p=0.409, I2=90%; - Huang, 2015: 1.07 (0.6-1.89), p=0.826, I2=88%; - Zhang, 2017: 1.28 (0.68-2.42), p=0.441, I2=90%; - Okano, 2019: 1.43 (0.84-2.43), p=0.186, I2=70%; - El-

										Haddad, 202: 1.18 (0.64-2.16), p=0.594, I2=90%; - Satoh, 2022: 1.04 (0.6-1.82), p=0.882, I2=87%; - Subasi, 2022: 1.34 (0.73-2.47), p=0.344, I2=90%
DGE treatment, RR	9	188/1887	69/1389	0.73 (0.24 - 1.22)	0.003	59.3 (15.1 - 80.5)	0.012	0.677	Park, 2011; Kitahata, 2014; Huang, 2015; Zhang, 2017; Lee, 2018; Okano, 2019; Han, 2021; El-Haddad, 2021; Satoh, 2022	- Park, 2011: 1.98 (1.18-3.32), p=0.01, I2=63%; - Kitahata, : 2.13 (1.25-3.64), p=0.006, I2=64%; - Huang, 201: 1.88 (1.13-3.14), p=0.015, I2=60%; - Zhang, 201: 2.54 (1.63-3.94), p=< 0.001,

										I2=32%;<br>- Lee, 2018: 2 (1.14-3.5), p=0.016, I2=64%;<br>- Okano, 201: 1.79 (1.08-2.95), p=0.023, I2=42%;<br>- Han, 2021: 2.1 (1.25-3.52), p=0.005, I2=64%;<br>- El-Haddad,: 2.34 (1.46-3.76), p=< 0.001, I2=54%;<br>- Satoh, 202: 2.05 (1.19-3.52), p=0.009, I2=64%
Soft pancreas treatment, RR	7	185/423	179/352	-0.17 (-0.32 - -0.02)	0.029	1.1 (0 - 71.1)	0.416	0.029	Kitahata, 2014; Fujii, 2015; Zhang, 2017; El-Haddad, 2021; Suenaga, 2021; Satoh, 2022;	- Kitahata, 2014: 0.8 (0.67-0.96), p=0.014, I2=0%;<br>- Fujii, 2015: 0.85 (0.73-1), p=0.05,

Subasi, 2022	I2=15%; - Zhang, 2017: 0.86 (0.72-1.01), p=0.073, I2=15%; - El-Haddad, 202: 0.87 (0.74-1.01), p=0.073, I2=0%; - Suenaga, 2021: 0.87 (0.74-1.02), p=0.08, I2=0%; - Satoh, 2022: 0.8 (0.66-0.97), p=0.022, I2=3%; - Subasi, 2022: 0.84 (0.72-0.99), p=0.035, I2=18%

Selectie: PS - SEMS

Meta-analysis for Pancreatitis treatment, comparing PS with SEMS

	datele. Year	datele.Study.name	numberCasesPerTreatment	numberCasesTreatment	numberCasesPerControl	numberCasesControl
3	2016	Tol, 2016	7	102	9	49
4	2016	Song, 2016	0	43	5	43
5	2020	Latenstein, 2020	25	329	22	246
6	2020	Cho, 2020	5	26	5	27
8	2021	Roberts, 2021	6	108	2	49
2						

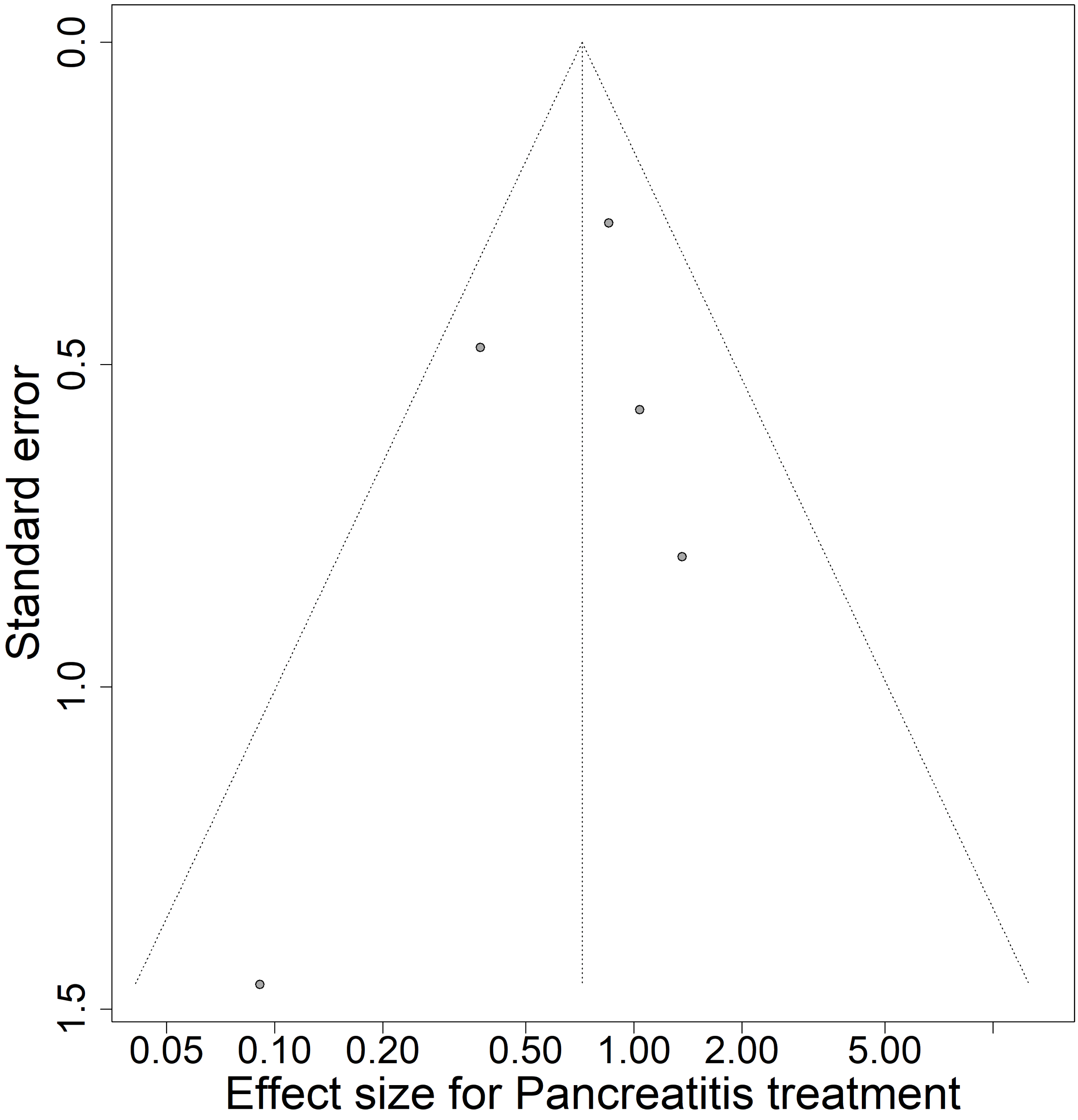

Fig. Funnel plot for Pancreatitis treatment, comparing PS with SEMS

The funnel plot for Pancreatitis treatment, comparing PS with SEMS is shown in figure _.

The publication bias test gave a p=0.524.

Influence studies: Omitting Tol, 2016; Omitting Song, 2016; Omitting Latenstein, 2020; Omitting Cho, 2020; Omitting Roberts, 2021 - yes; no; yes; no; no

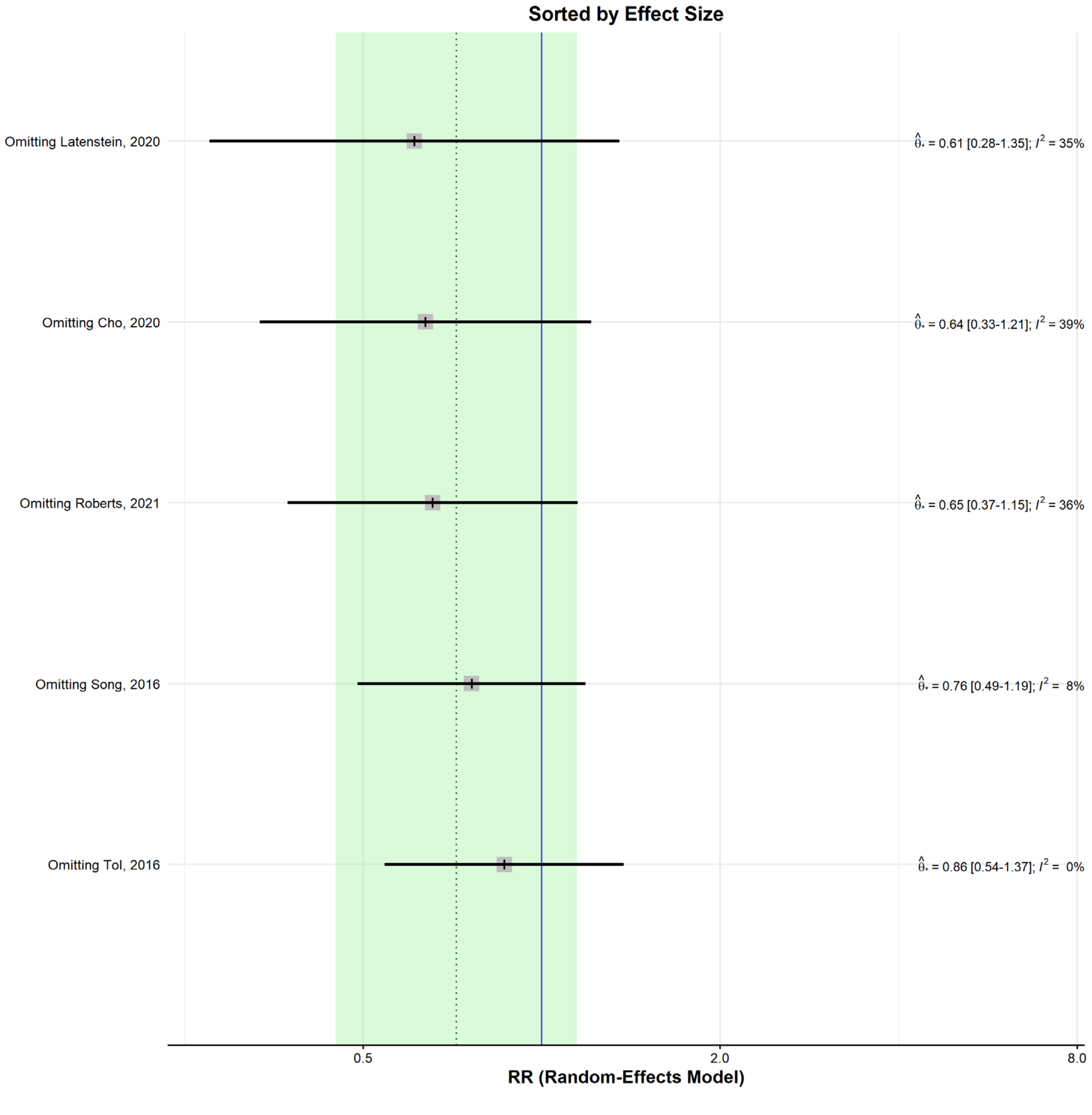

Fig. Leave-one-out sensitivity analysis plot for selected studies for Pancreatitis treatment

The heterogeneity was assessed, and we found an I2 of 24.9% (95% CI 0% - 69.7%) and the Q test for heterogeneity gave p=0.256.

The RR value (the RR of Pancreatitis treatment in the PS group compared to the SEMS group) obtained with the meta-analysis was of -0.33 (95% CI -0.8 - 0.14), p=0.165 using the model with random effects.

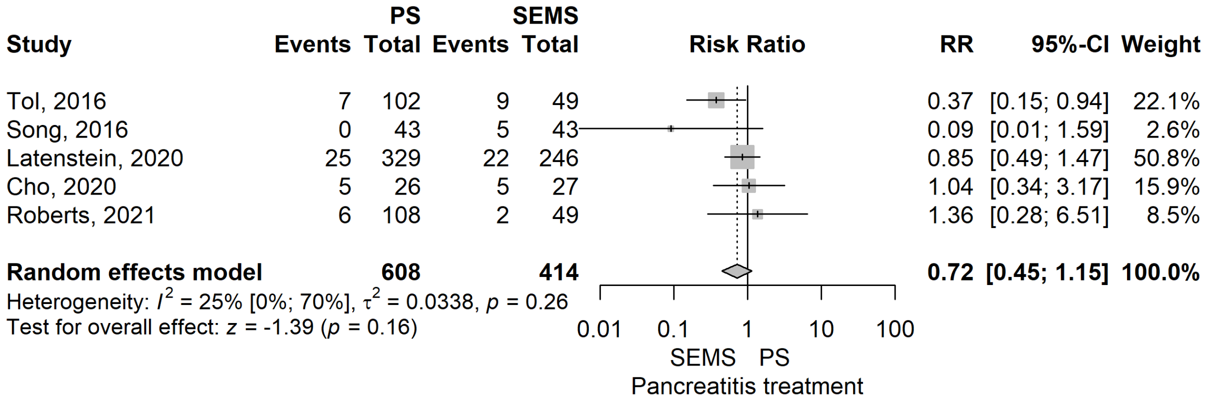

Fig. Forest plot for Pancreatitis treatment, comparing PS with SEMS

Meta-analysis for Cholangitis treatment, comparing PS with SEMS

	datele.Year	datele.Study.name	numberCasesPerTreatment	numberCasesTreatment	numberCasesPerControl	numberCasesControl
3	2016	Tol, 2016	27	102	0	49
4	2016	Song, 2016	2	43	0	43
5	2020	Latenstein, 2020	32	329	19	246
6	2020	Cho, 2020	1	26	0	27
8	2021	Roberts, 2021	14	108	2	49

2

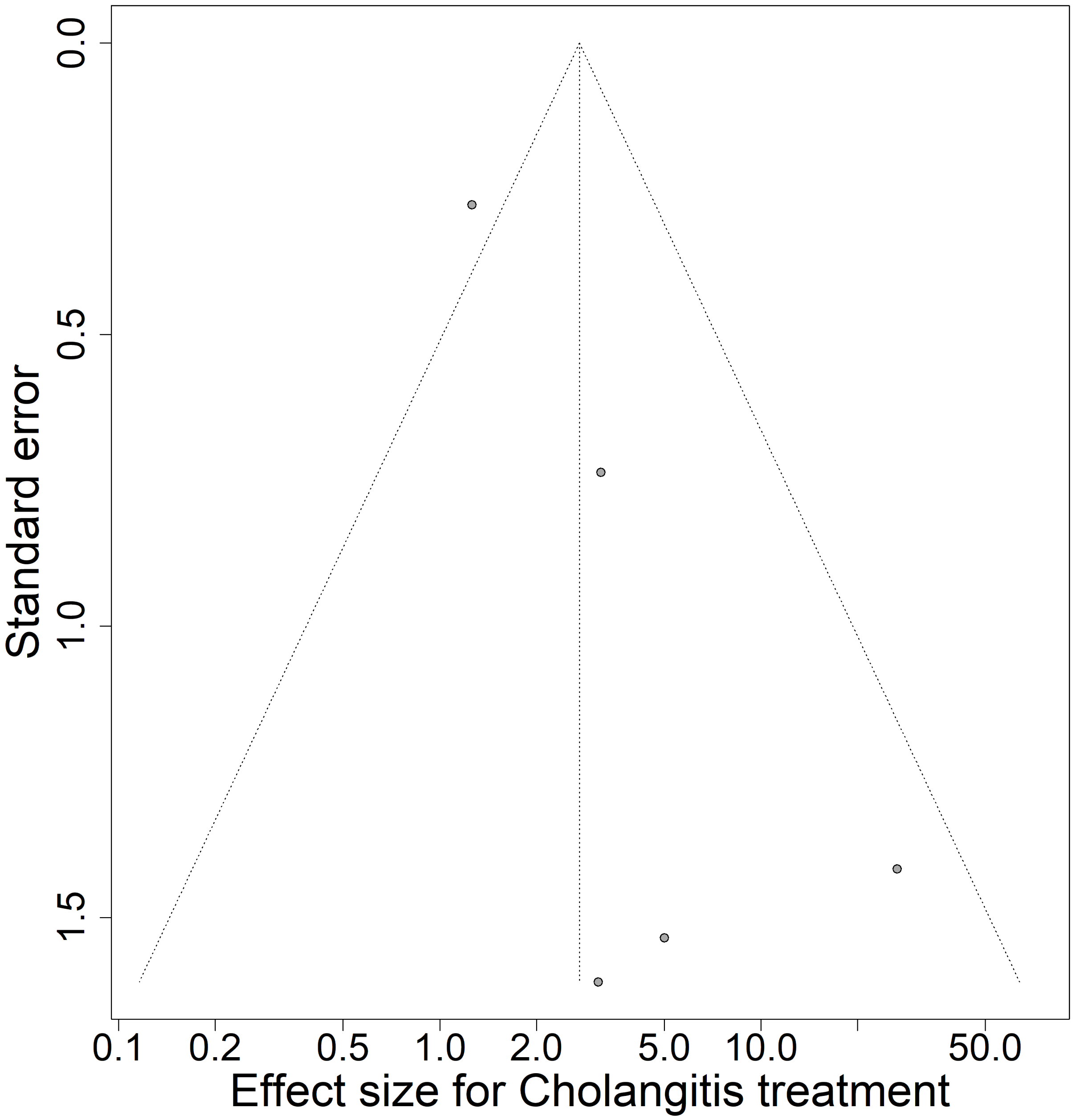

Fig. Funnel plot for Cholangitis treatment, comparing PS with SEMS

The funnel plot for Cholangitis treatment, comparing PS with SEMS is shown in figure _.

The publication bias test gave a p=0.057.

Influence studies: Omitting Tol, 2016; Omitting Song, 2016; Omitting Latenstein, 2020; Omitting Cho, 2020; Omitting Roberts, 2021 - yes; no; yes; no; no

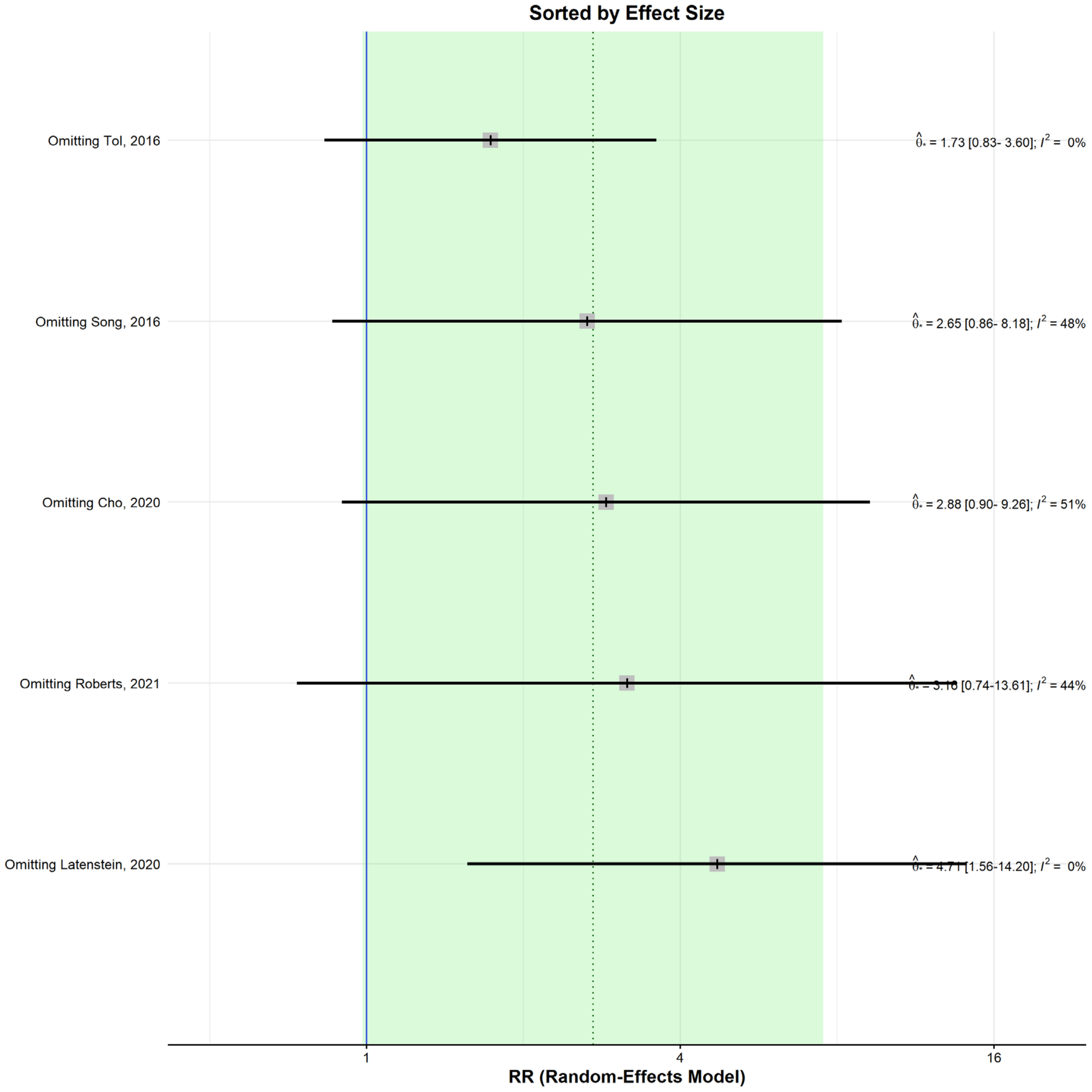

Fig. Leave-one-out sensitivity analysis plot for selected studies for Cholangitis treatment

The heterogeneity was assessed, and we found an I2 of 36.2% (95% CI 0% - 76.1%) and the Q test for heterogeneity gave p=0.18.

The RR value (the RR of Cholangitis treatment in the PS group compared to the SEMS group) obtained with the meta-analysis was of 1 (95% CI -0.02 - 2.02), p=0.054 using the model with random effects.

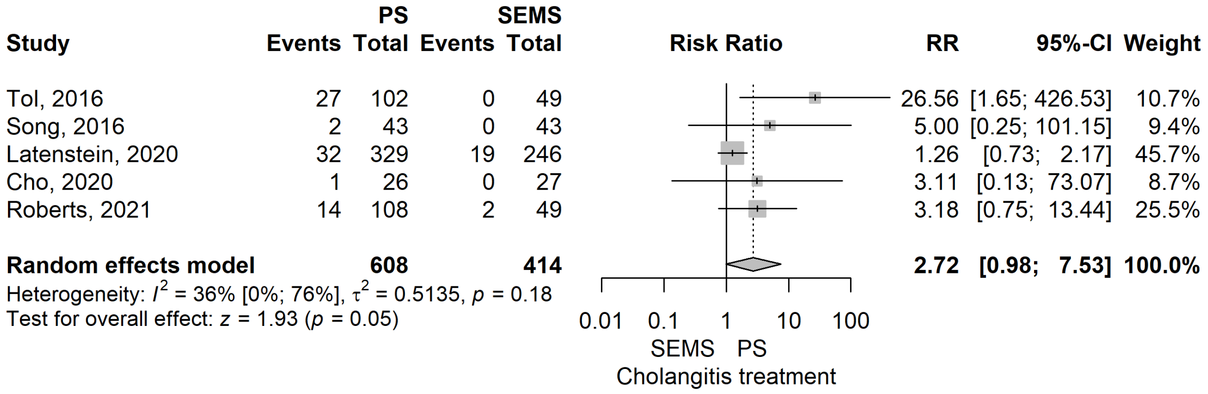

Fig. Forest plot for Cholangitis treatment, comparing PS with SEMS

Meta-analysis for Perforation treatment, comparing PS with SEMS

	datele.Year	datele.Study.name	numberCasesPerTreatment	numberCasesTreatment	numberCasesPerControl	numberCasesControl
3	2016	Tol, 2016	2	102	1	49
5	2020	Latenstein, 2020	13	329	3	246
8	2021	Roberts, 2021	0	108	0	49
			2			

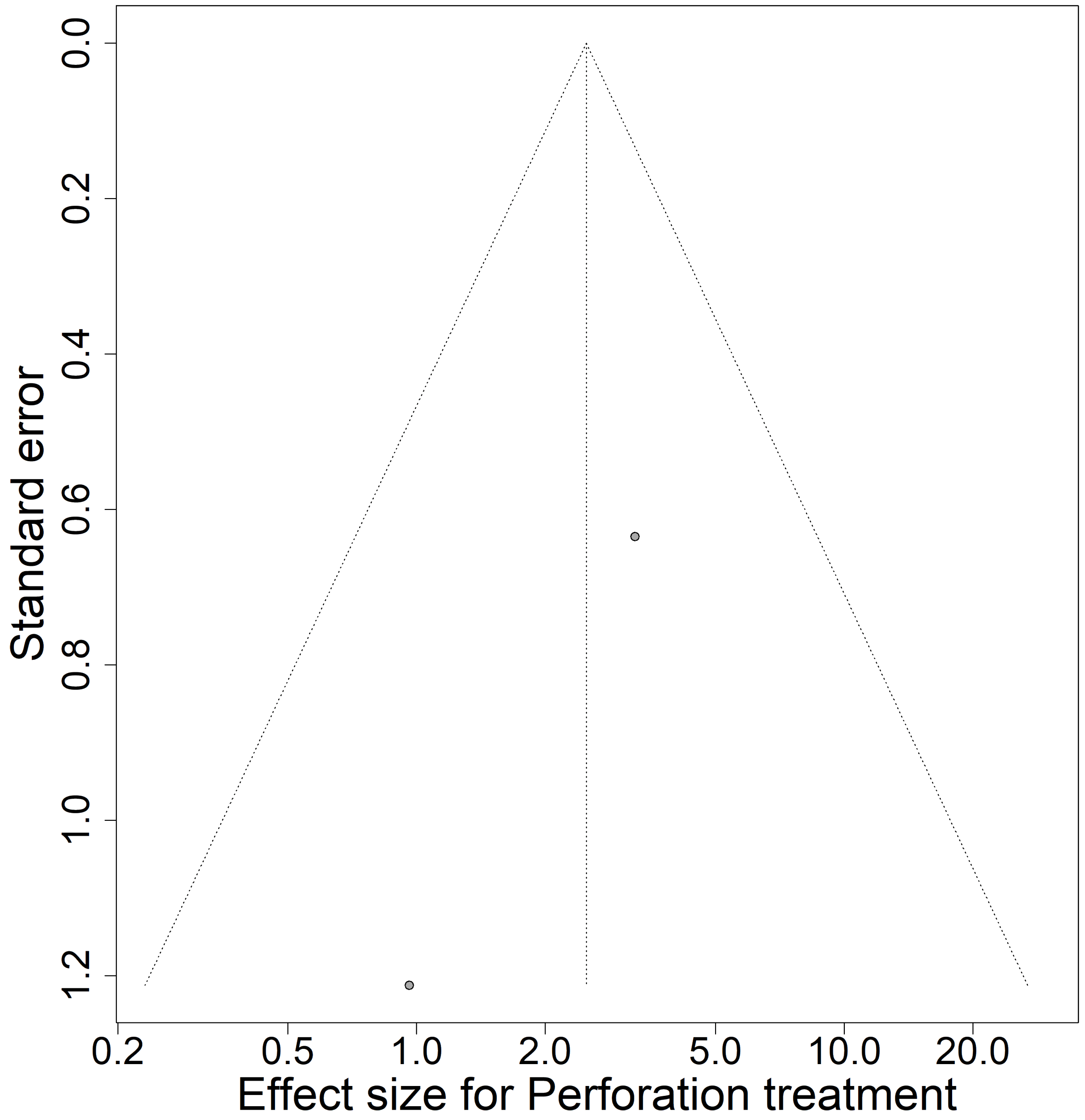

Fig. Funnel plot for Perforation treatment, comparing PS with SEMS

The funnel plot for Perforation treatment, comparing PS with SEMS is shown in figure _.

The publication bias test cannot be computed since there is a problem with the studies.

Influence studies: Omitting Tol, 2016; Omitting Latenstein, 2020 - no; yes

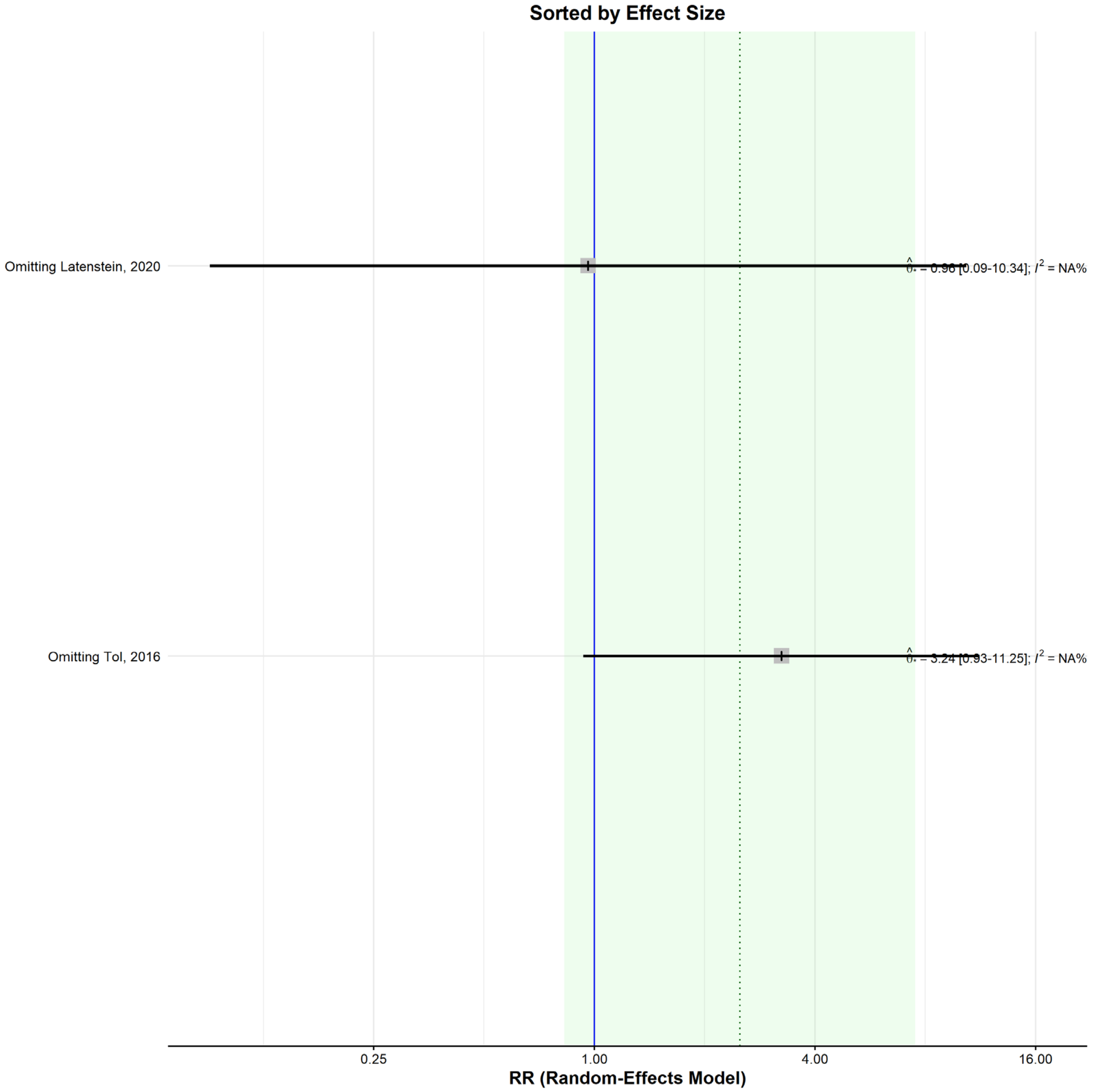

Fig. Leave-one-out sensitivity analysis plot for selected studies for Perforation treatment

The heterogeneity was assessed, and we found an I2 of 0% (95% CI NA% - NA%) and the Q test for heterogeneity gave p=0.374.

The RR value (the RR of Perforation treatment in the PS group compared to the SEMS group) obtained with the meta-analysis was of 0.91 (95% CI -0.19 - 2.02), p=0.104 using the model with random effects.

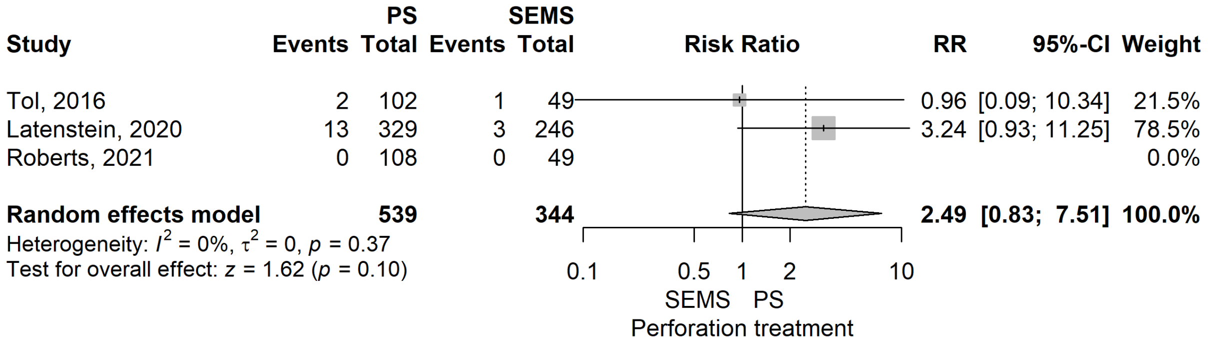

Fig. Forest plot for Perforation treatment, comparing PS with SEMS

Meta-analysis for Hemorrhage treatment, comparing PS with SEMS

	datele.Year	datele.Study.name	numberCasesPerTreatment	numberCasesTreatment	numberCasesPerControl	numberCasesControl
3	2016	Tol, 2016	2	102	0	49
5	2020	Latenstein, 2020	15	329	8	246
6	2020	Cho, 2020	1	26	0	27
8	2021	Roberts, 2021	2	108	0	49

2

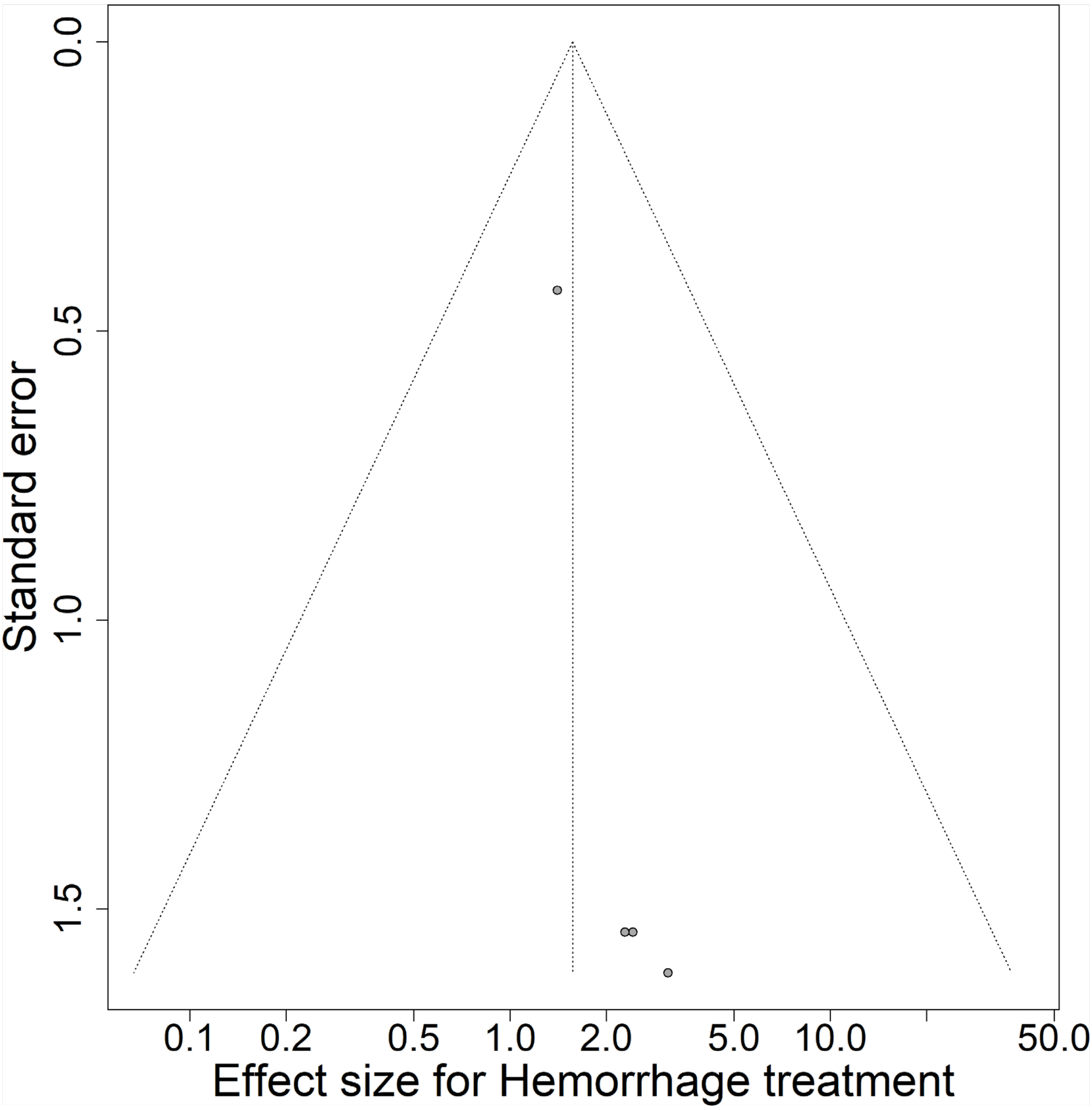

Fig. Funnel plot for Hemorrhage treatment, comparing PS with SEMS

The funnel plot for Hemorrhage treatment, comparing PS with SEMS is shown in figure _.

The publication bias test gave a p=0.022.

Influence studies: Omitting Tol, 2016; Omitting Latenstein, 2020; Omitting Cho, 2020; Omitting Roberts, 2021 - no; yes; no; no

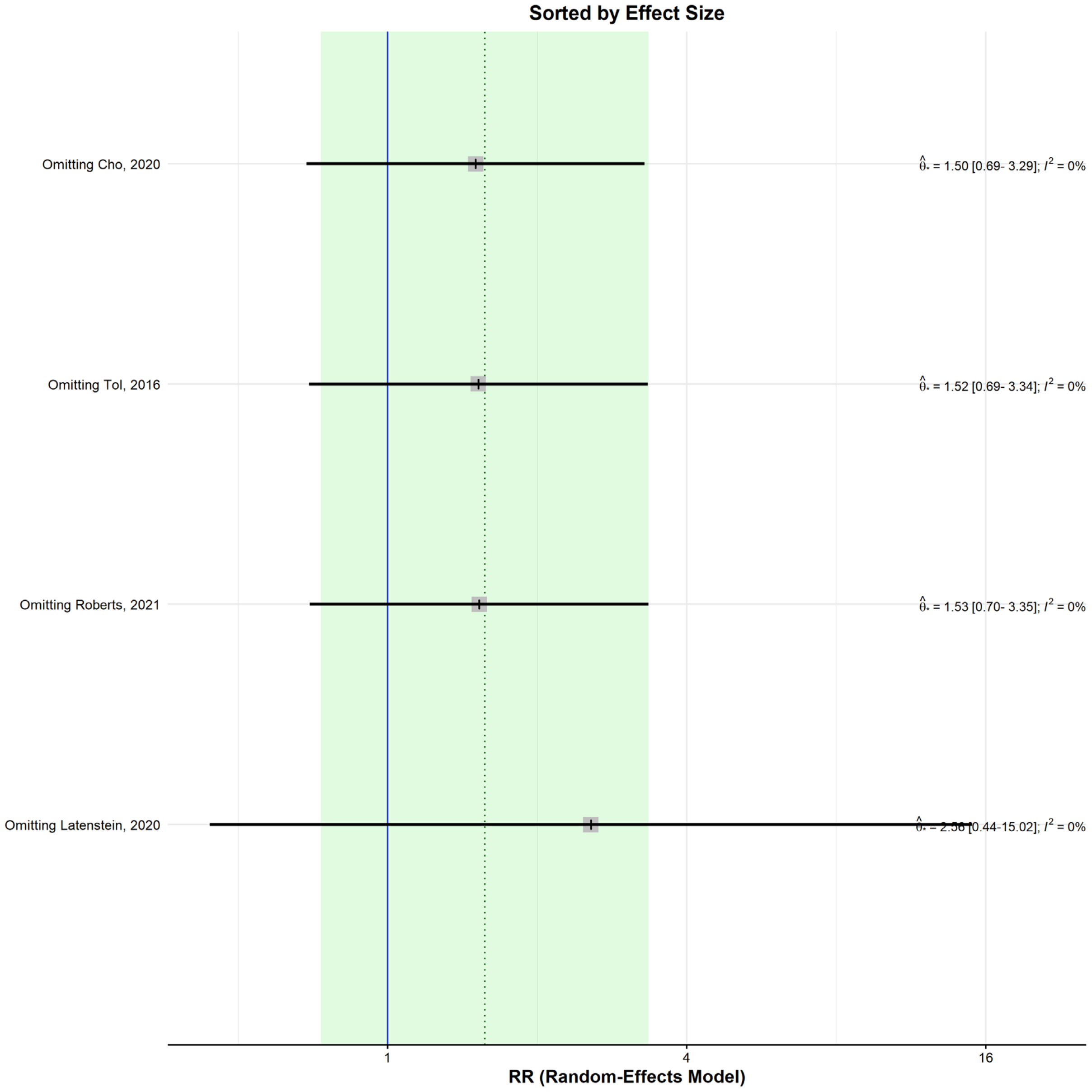

Fig. Leave-one-out sensitivity analysis plot for selected studies for Hemorrhage treatment

The heterogeneity was assessed, and we found an I2 of 0% (95% CI 0% - 84.7%) and the Q test for heterogeneity gave p=0.943.

The RR value (the RR of Hemorrhage treatment in the PS group compared to the SEMS group) obtained with the meta-analysis was of 0.45 (95% CI -0.31 - 1.21), p=0.246 using the model with random effects.

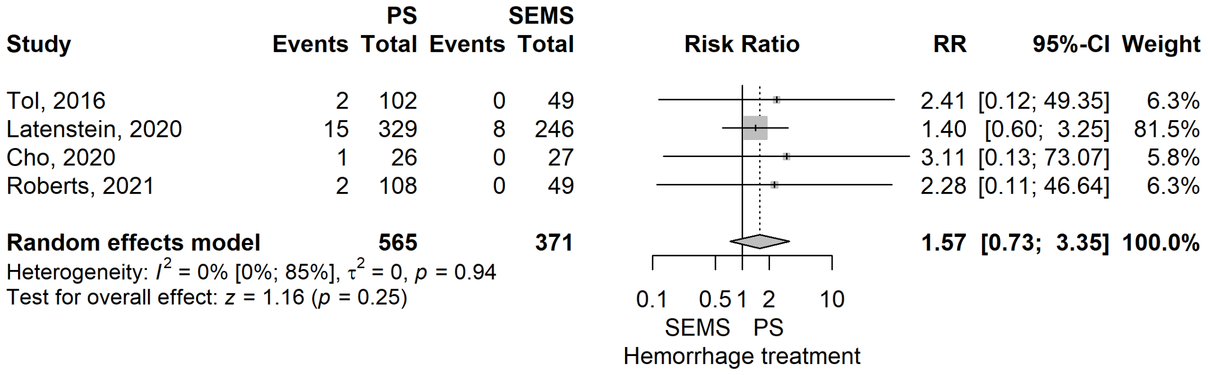

Fig. Forest plot for Hemorrhage treatment, comparing PS with SEMS

Meta-analysis for Occlusion treatment, comparing PS with SEMS

	datele.Year	datele.Study.name	numberCasesPerTreatment	numberCasesTreatment	numberCasesPerControl	numberCasesControl
3	2016	Tol, 2016	15	102	2	49
4	2016	Song, 2016	5	43	2	43
5	2020	Latenstein, 2020	27	329	11	246
6	2020	Cho, 2020	1	26	1	27
8	2021	Roberts, 2021	11	108	1	49

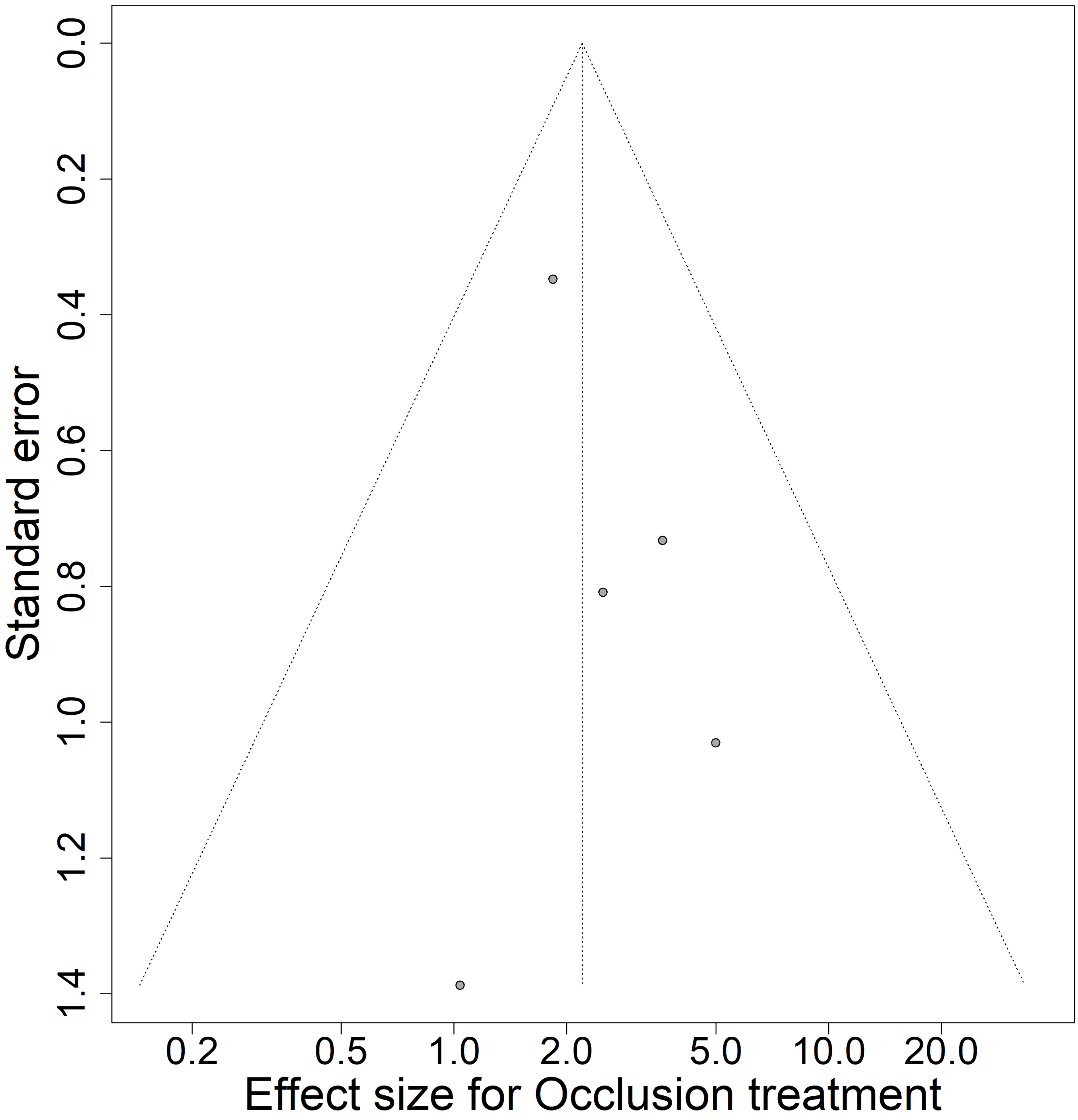

Fig. Funnel plot for Occlusion treatment, comparing PS with SEMS

The funnel plot for Occlusion treatment, comparing PS with SEMS is shown in figure _.

The publication bias test gave a p=0.482.

Influence studies: Omitting Tol, 2016; Omitting Song, 2016; Omitting Latenstein, 2020; Omitting Cho, 2020; Omitting Roberts, 2021 - no; no; yes; no; no

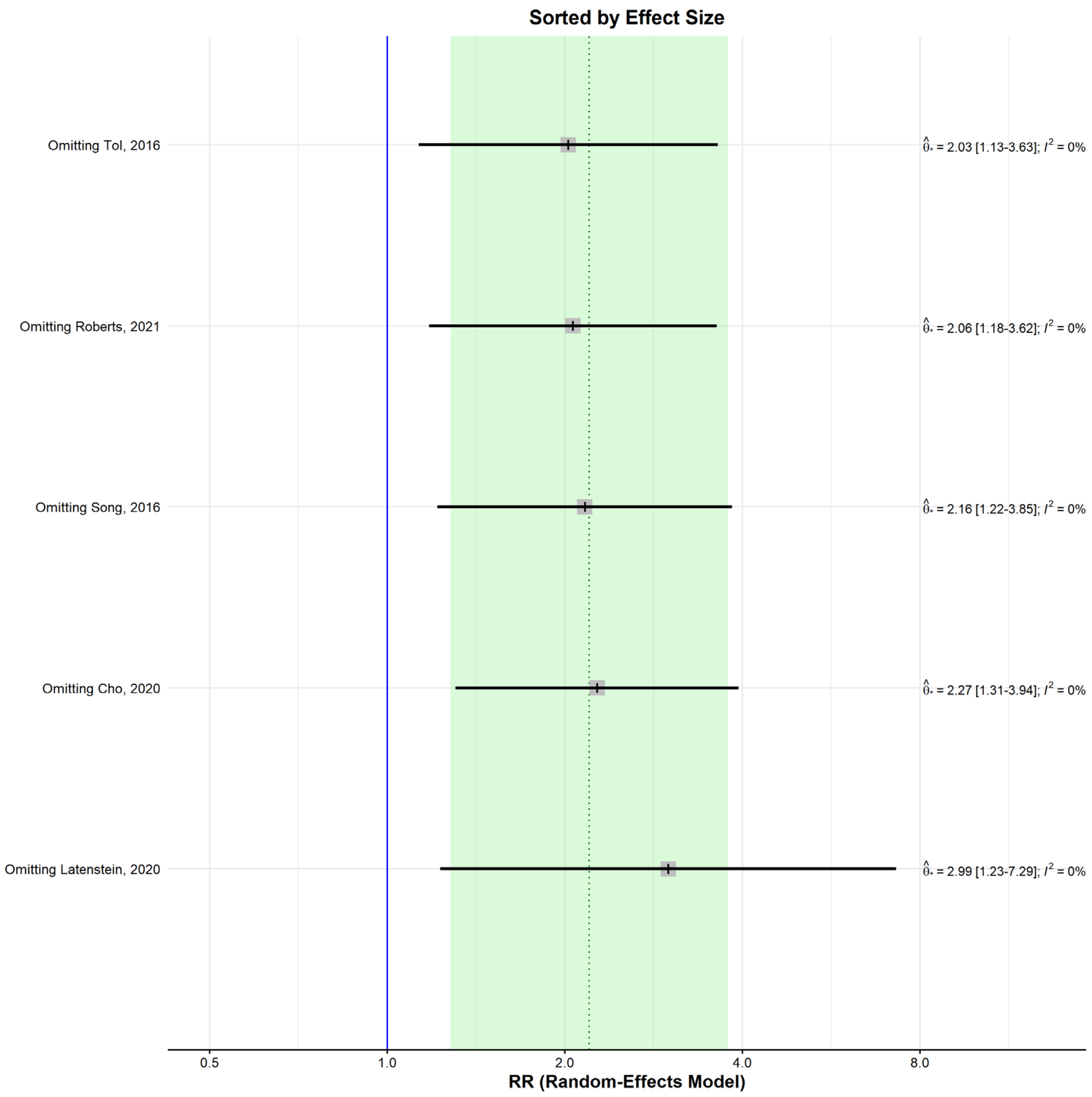

Fig. Leave-one-out sensitivity analysis plot for selected studies for Occlusion treatment

The heterogeneity was assessed, and we found an I2 of 0% (95% CI 0% - 79.2%) and the Q test for heterogeneity gave p=0.795.

The RR value (the RR of Occlusion treatment in the PS group compared to the SEMS group) obtained with the meta-analysis was of 0.79 (95% CI 0.25 - 1.33), p=0.004 using the model with random effects.

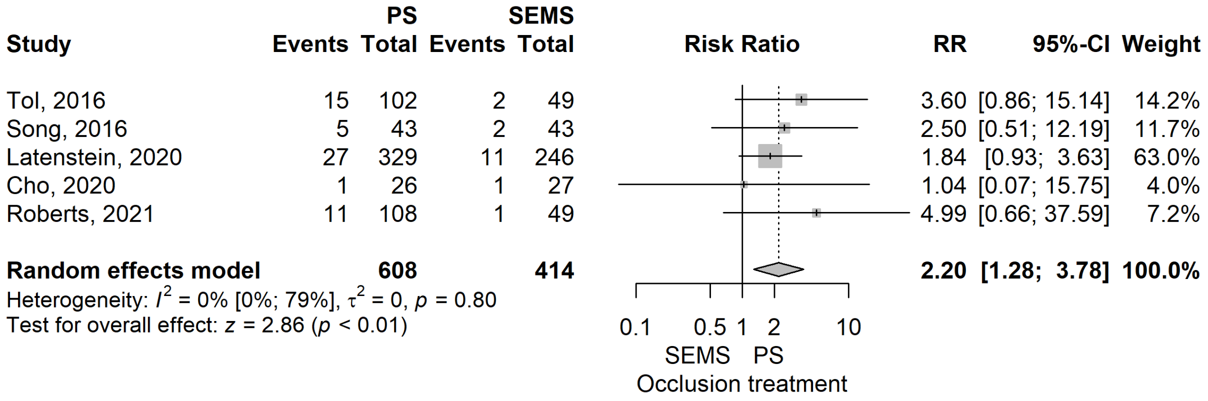

Fig. Forest plot for Occlusion treatment, comparing PS with SEMS

Meta-analysis for Catheter exchange treatment, comparing PS with SEMS

	datele.Year	datele.Study.name	numberCasesPerTreatment	numberCasesTreatment	numberCasesPerControl	numberCasesControl
3	2016	Tol, 2016	31	102	2	49
4	2016	Song, 2016	7	43	6	43
5	2020	Latenstein, 2020	32	329	22	246
6	2020	Cho, 2020	0	26	0	27

2

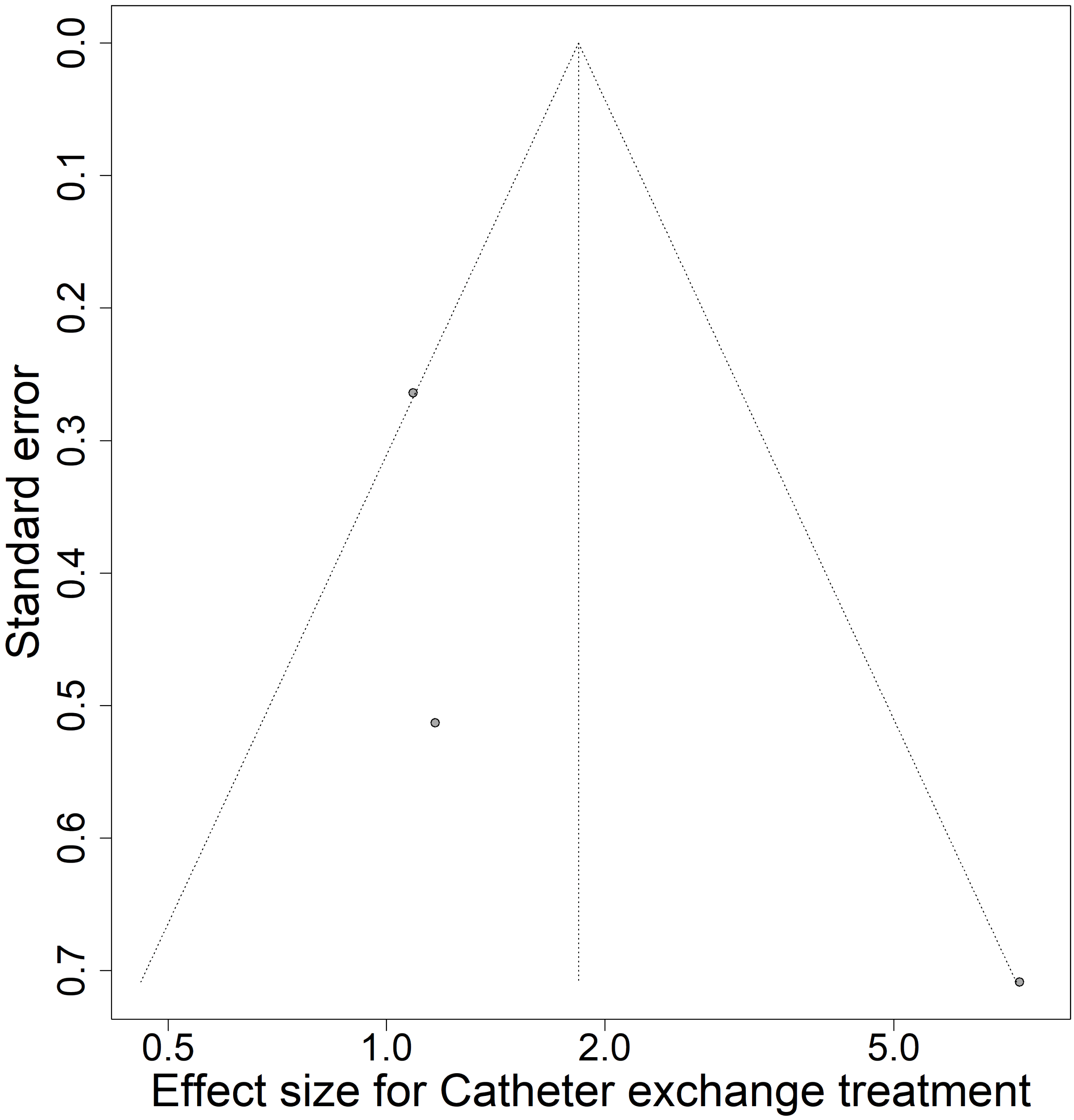

Fig. Funnel plot for Catheter exchange treatment, comparing PS with SEMS

The funnel plot for Catheter exchange treatment, comparing PS with SEMS is shown in figure _.

The publication bias test gave a p=0.407.

Influence studies: Omitting Tol, 2016; Omitting Song, 2016; Omitting Latenstein, 2020 - yes; no; yes

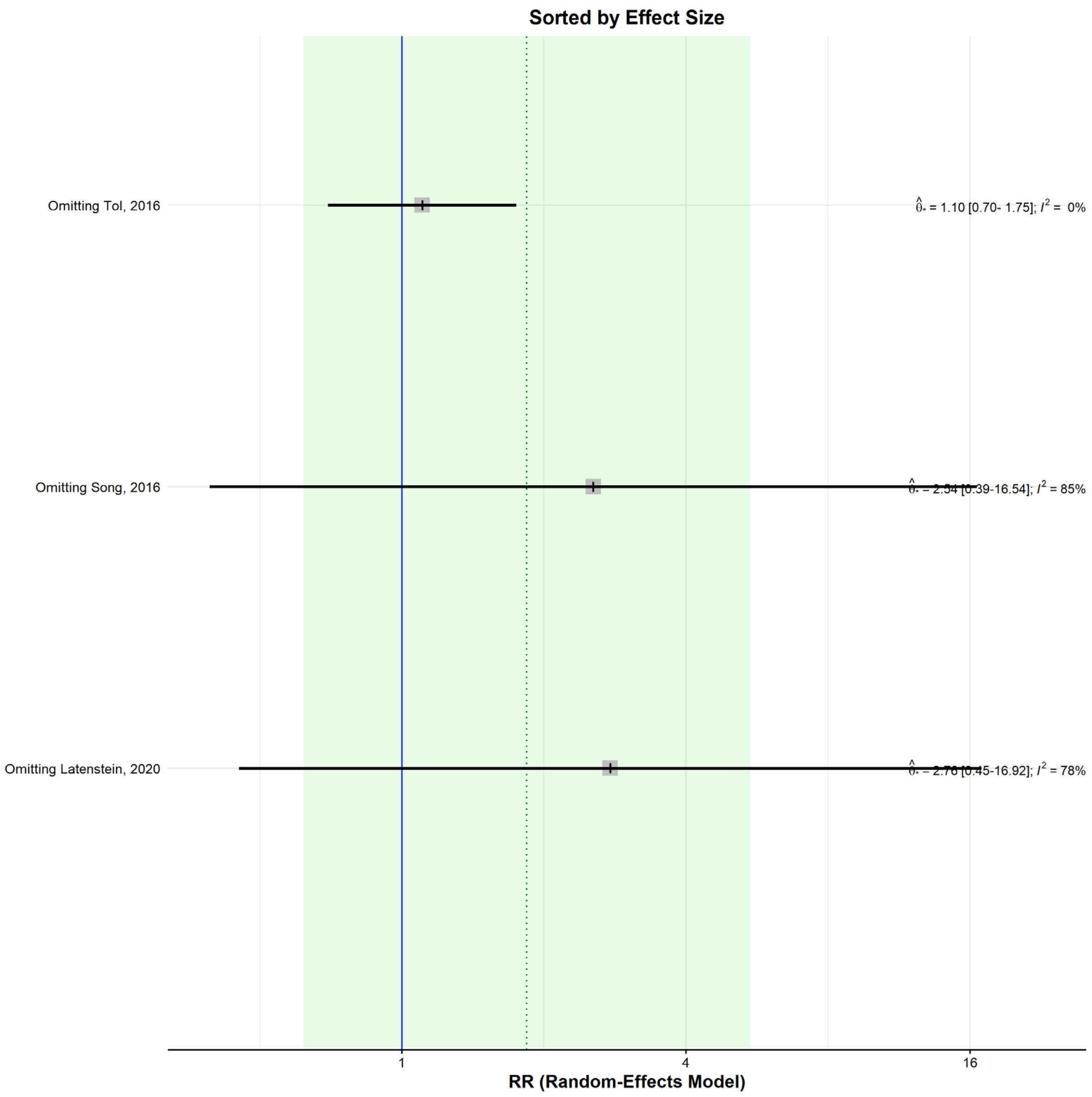

Fig. Leave-one-out sensitivity analysis plot for selected studies for Catheter exchange treatment

The heterogeneity was assessed, and we found an I2 of 69.5% (95% CI 0% - 91.1%) and the Q test for heterogeneity gave p=0.038.

The RR value (the RR of Catheter exchange treatment in the PS group compared to the SEMS group) obtained with the meta-analysis was of 0.61 (95% CI -0.48 - 1.7), p=0.273 using the model with random effects.

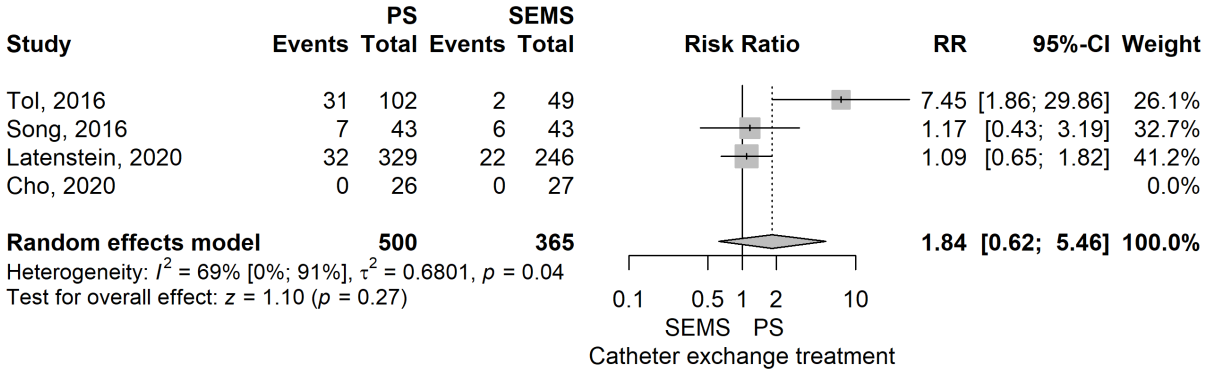

Fig. Forest plot for Catheter exchange treatment, comparing PS with SEMS

Meta-analysis for Mortality treatment, comparing PS with SEMS

	datele.Year	datele.Study.name	numberCasesPerTreatment	numberCasesTreatment	numberCasesPerControl	numberCasesControl
1	2013	Cavell, 2013	0	149	0	71
3	2016	Tol, 2016	9	102	1	49
4	2016	Song, 2016	1	43	0	43
5	2020	Latenstein, 2020	4	329	4	246
6	2020	Cho, 2020	1	26	0	27
9	2022	Bademci, 2022	0	17	4	31

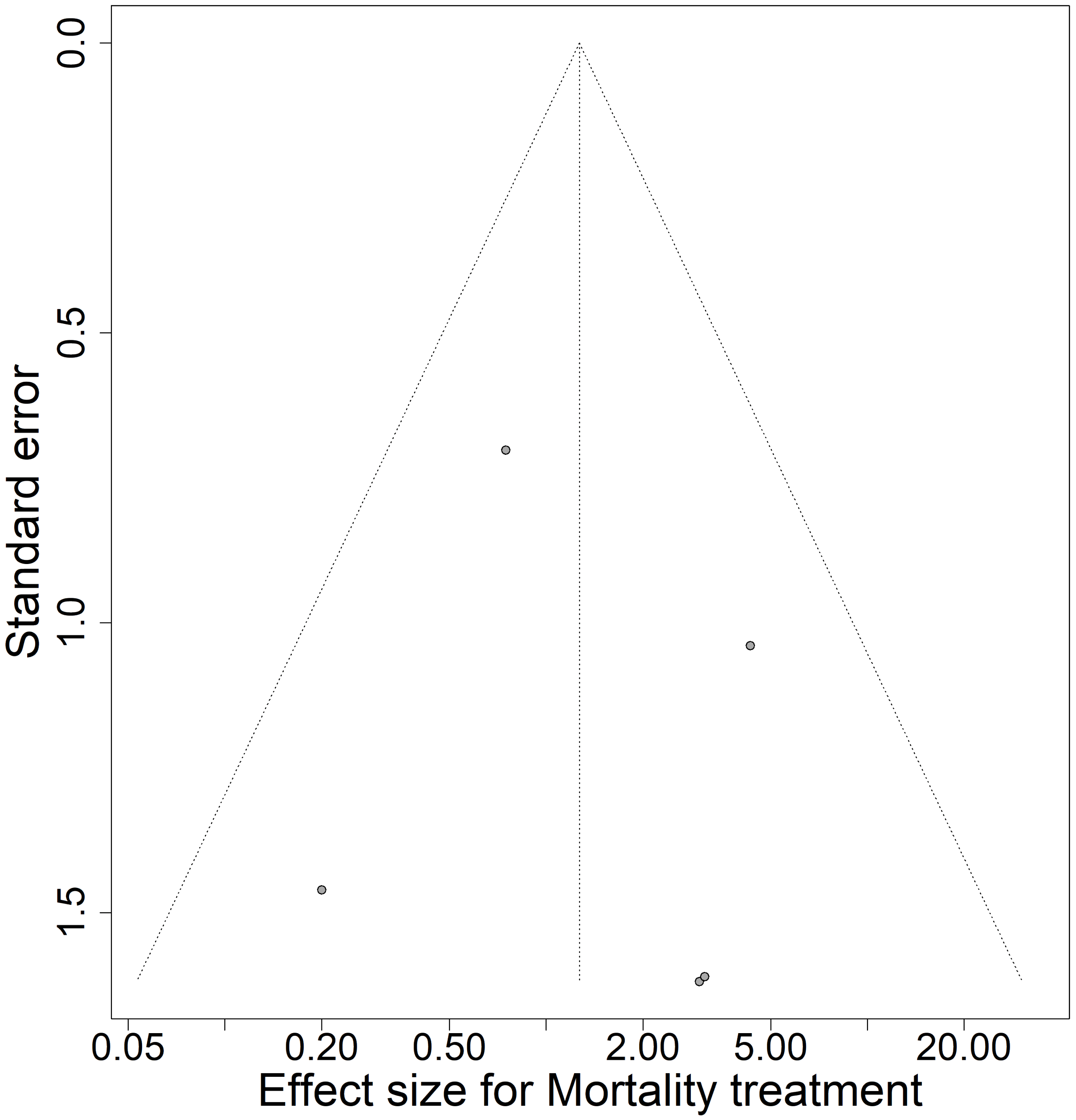

Fig. Funnel plot for Mortality treatment, comparing PS with SEMS

The funnel plot for Mortality treatment, comparing PS with SEMS is shown in figure _.

The publication bias test gave a p=0.685.

Influence studies: Omitting Tol, 2016; Omitting Song, 2016; Omitting Latenstein, 2020; Omitting Cho, 2020; Omitting Bademci, 2022 - yes; no; yes; no; no

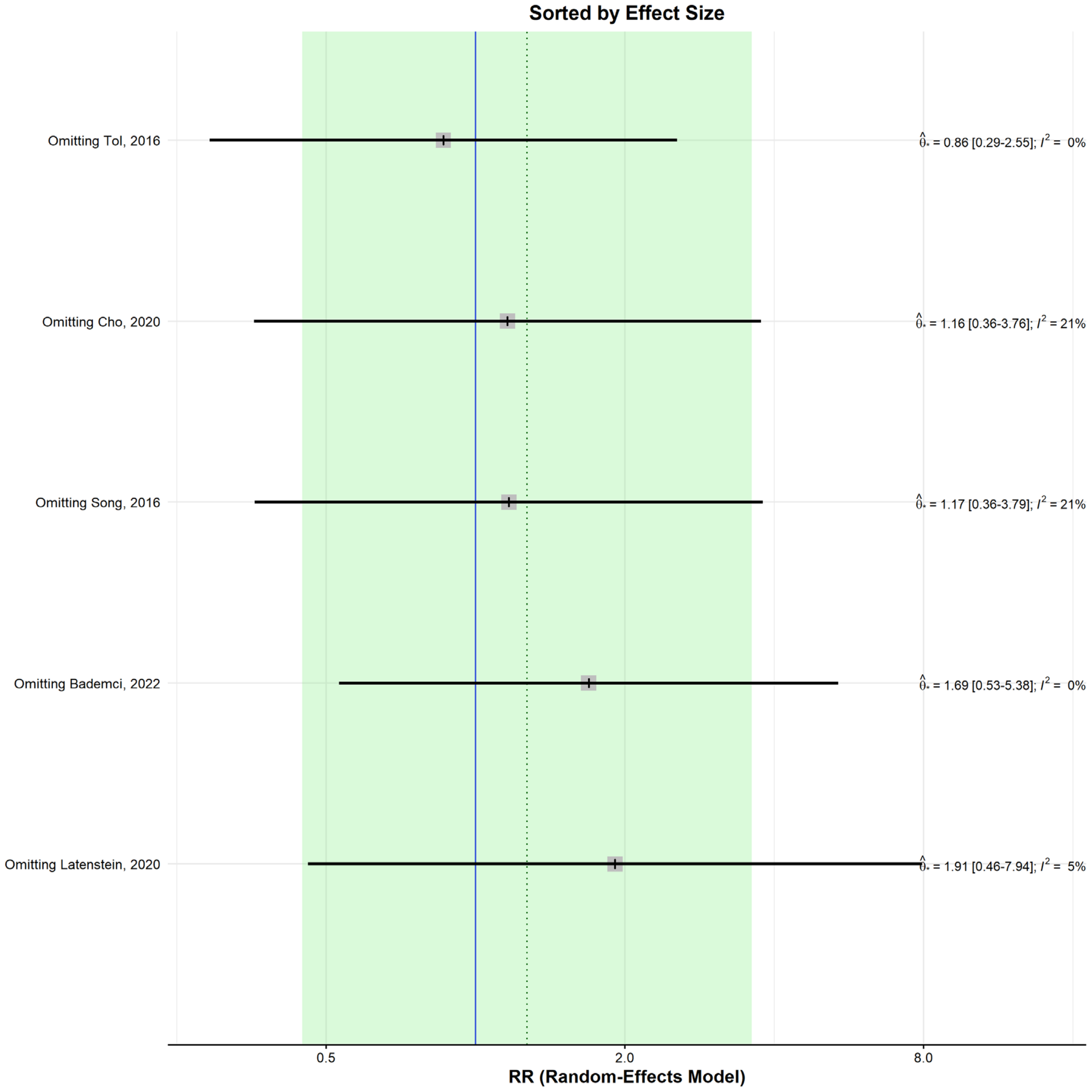

Fig. Leave-one-out sensitivity analysis plot for selected studies for Mortality treatment

The heterogeneity was assessed, and we found an I2 of 3.5% (95% CI 0% - 79.9%) and the Q test for heterogeneity gave p=0.386.

The RR value (the RR of Mortality treatment in the PS group compared to the SEMS group) obtained with the meta-analysis was of 0.24 (95% CI -0.8 - 1.28), p=0.654 using the model with random effects.

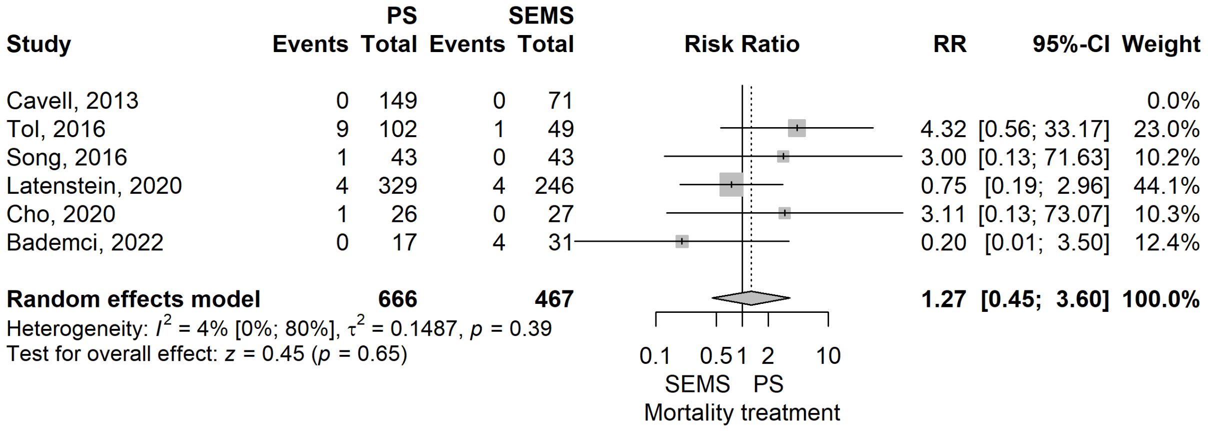

Fig. Forest plot for Mortality treatment, comparing PS with SEMS

Meta-analysis for Reoperation treatment, comparing PS with SEMS

	datele.Year	datele.Study.name	numberCasesPerTreatment	numberCasesTreatment	numberCasesPerControl	numberCasesControl
2	2015	Haapamaki, 2015	6	163	3	28
3	2016	Tol, 2016	12	102	3	49
5	2020	Latenstein, 2020	25	329	15	246

2

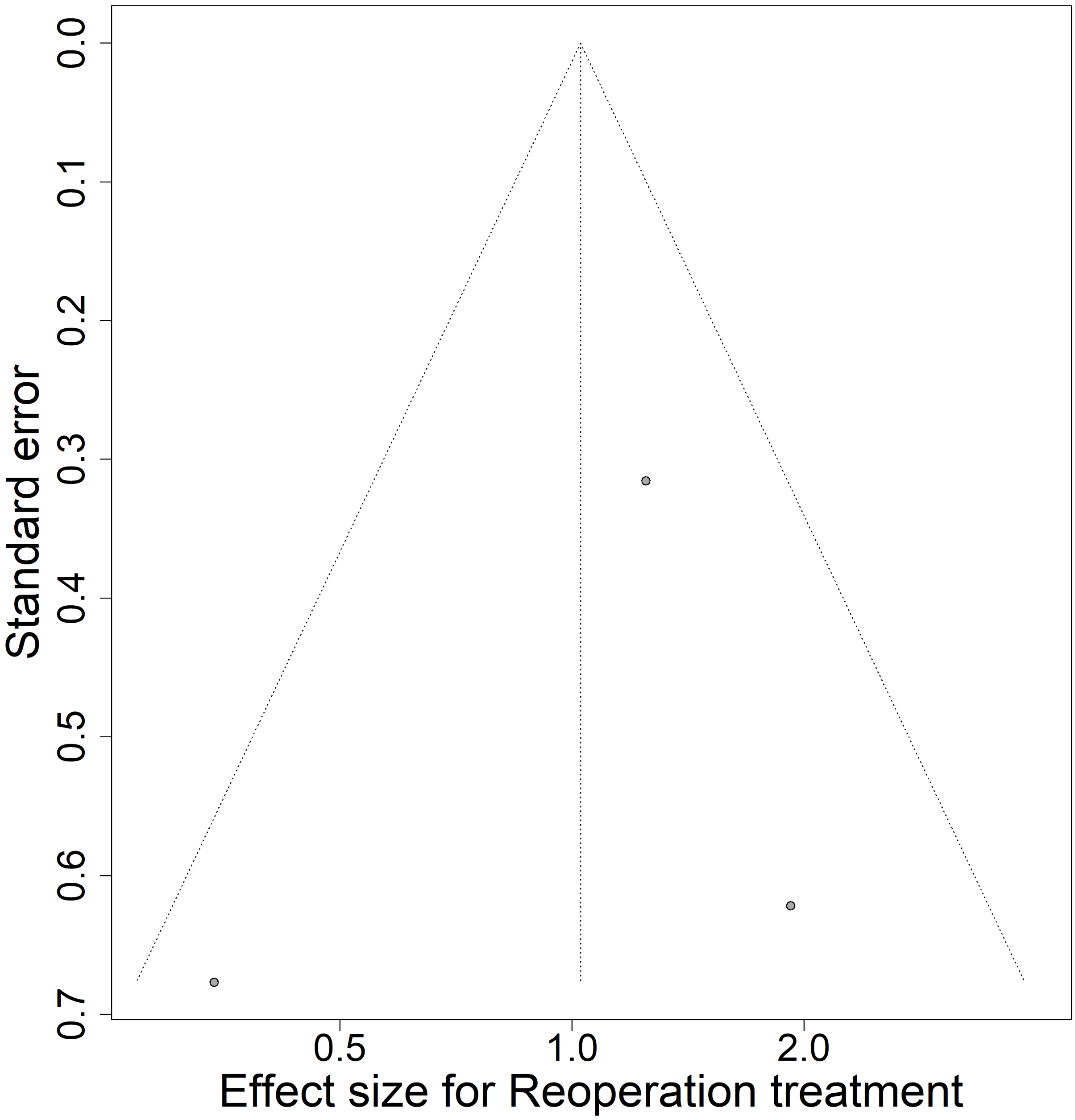

Fig. Funnel plot for Reoperation treatment, comparing PS with SEMS

The funnel plot for Reoperation treatment, comparing PS with SEMS is shown in figure _.

The publication bias test gave a p=0.727.

Influence studies: Omitting Haapamaki, 2015; Omitting Tol, 2016; Omitting Latenstein, 2020 - yes; yes; no

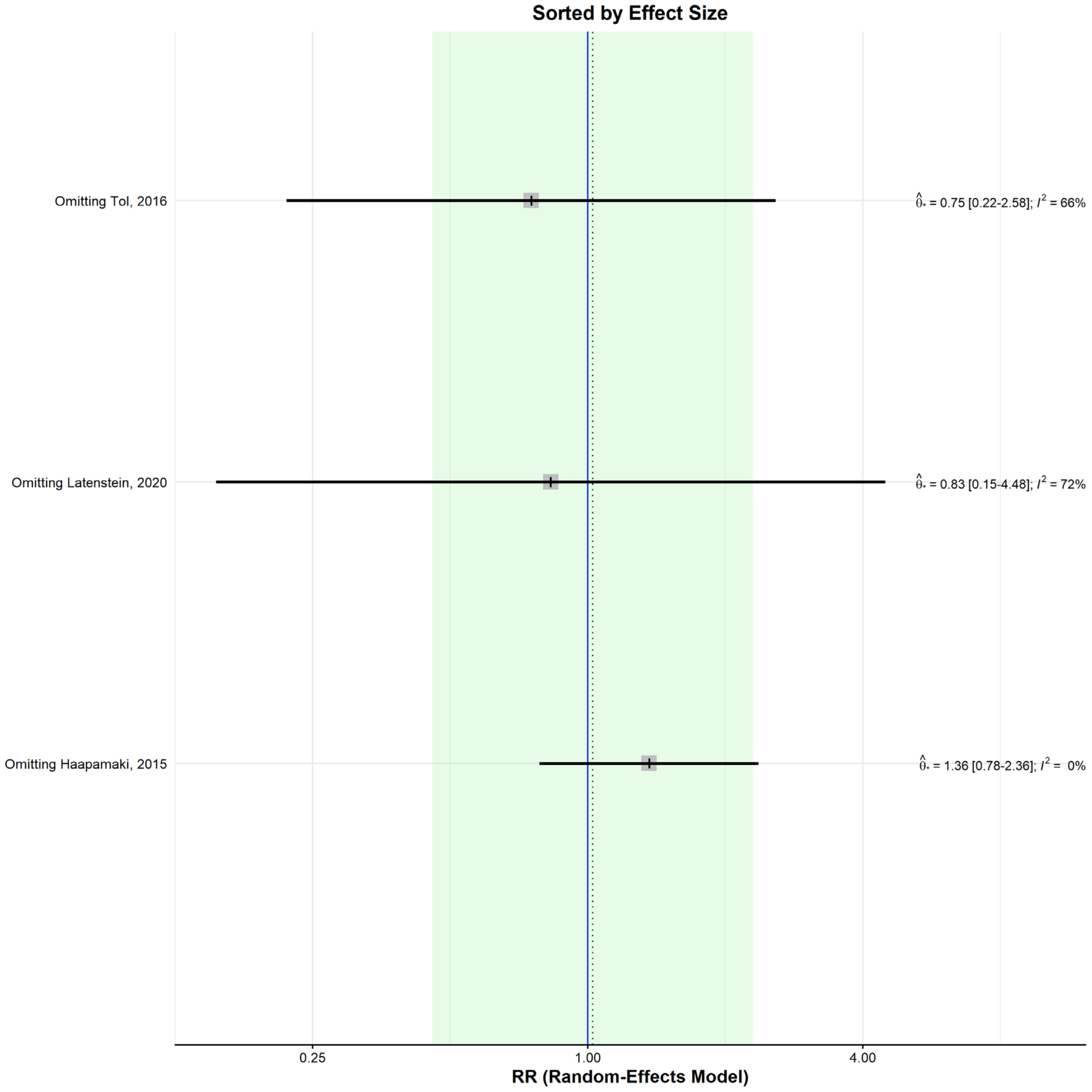

Fig. Leave-one-out sensitivity analysis plot for selected studies for Reoperation treatment

The heterogeneity was assessed, and we found an I2 of 48.9% (95% CI 0% - 85.1%) and the Q test for heterogeneity gave p=0.141.

The RR value (the RR of Reoperation treatment in the PS group compared to the SEMS group) obtained with the meta-analysis was of 0.03 (95% CI -0.78 - 0.83), p=0.951 using the model with random effects.

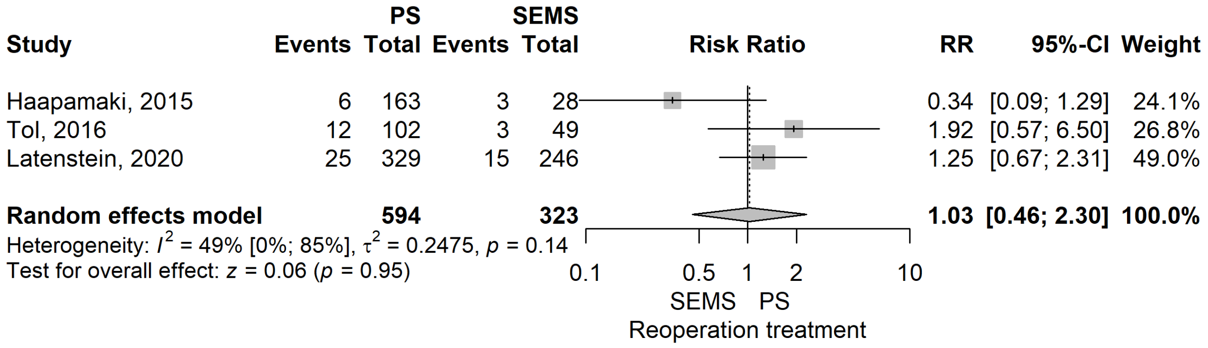

Fig. Forest plot for Reoperation treatment, comparing PS with SEMS

Meta-analysis for Overall complication rate (Clavien - Dindo) treatment, comparing PS with SEMS

	datele.Year	datele.Study.name	numberCasesPerTreatment	numberCasesTreatment	numberCasesPerControl	numberCasesControl
1	2013	Cavell, 2013	82	149	45	71
2	2015	Haapamaki, 2015	18	163	0	28
3	2016	Tol, 2016	48	102	19	49
4	2016	Song, 2016	17	43	14	43
5	2020	Latenstein, 2020	202	329	161	246
6	2020	Cho, 2020	11	26	8	27
8	2021	Roberts, 2021	40	108	20	49

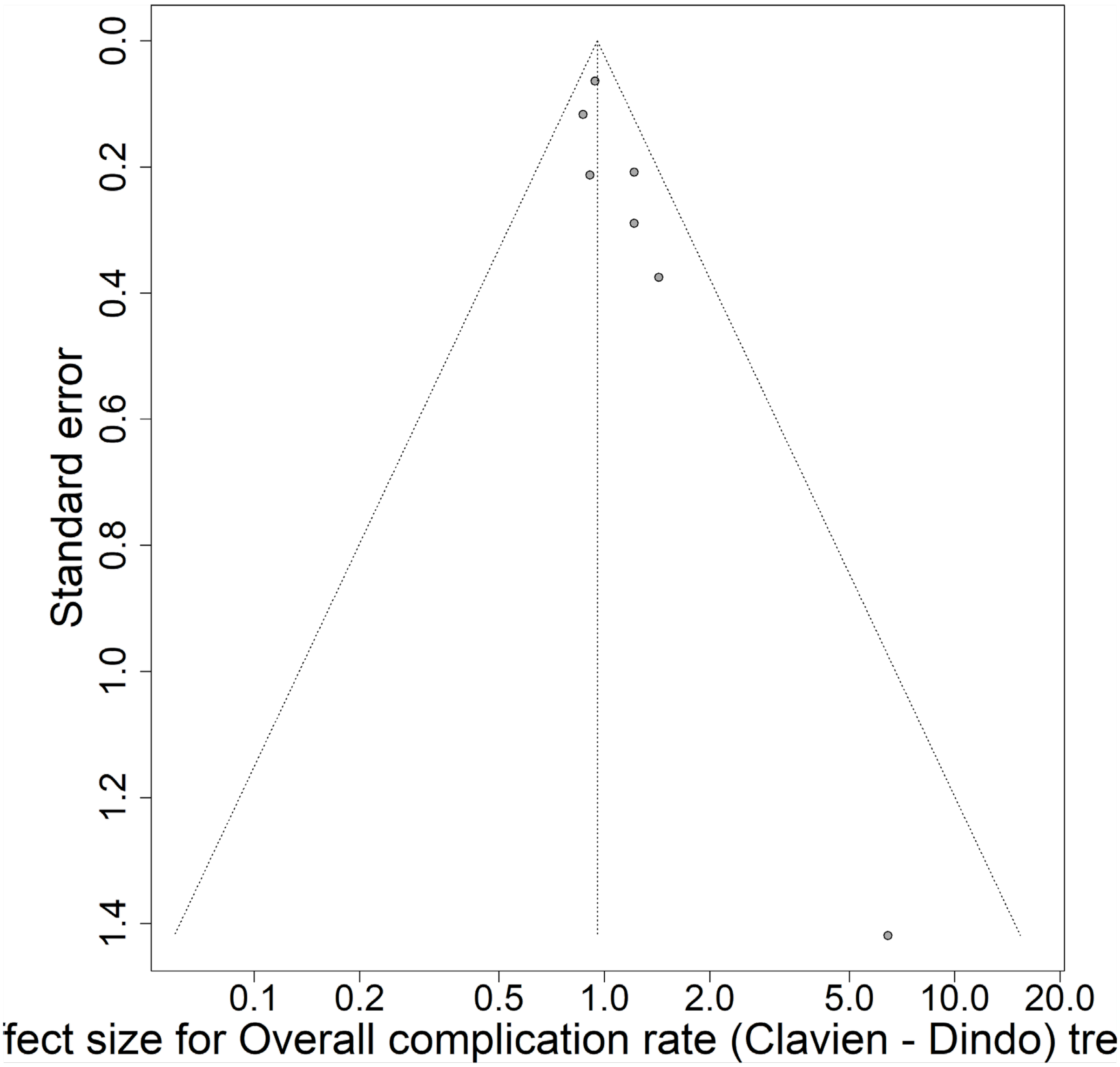

Fig. Funnel plot for Overall complication rate (Clavien - Dindo) treatment, comparing PS with SEMS

The funnel plot for Overall complication rate (Clavien - Dindo) treatment, comparing PS with SEMS is shown in figure _.

The publication bias test gave a p=0.038.

Influence studies: Omitting Cavell, 2013; Omitting Haapamaki, 2015; Omitting Tol, 2016; Omitting Song, 2016; Omitting Latenstein, 2020; Omitting Cho, 2020; Omitting Roberts, 2021 - yes; no; no; no; yes; no; no

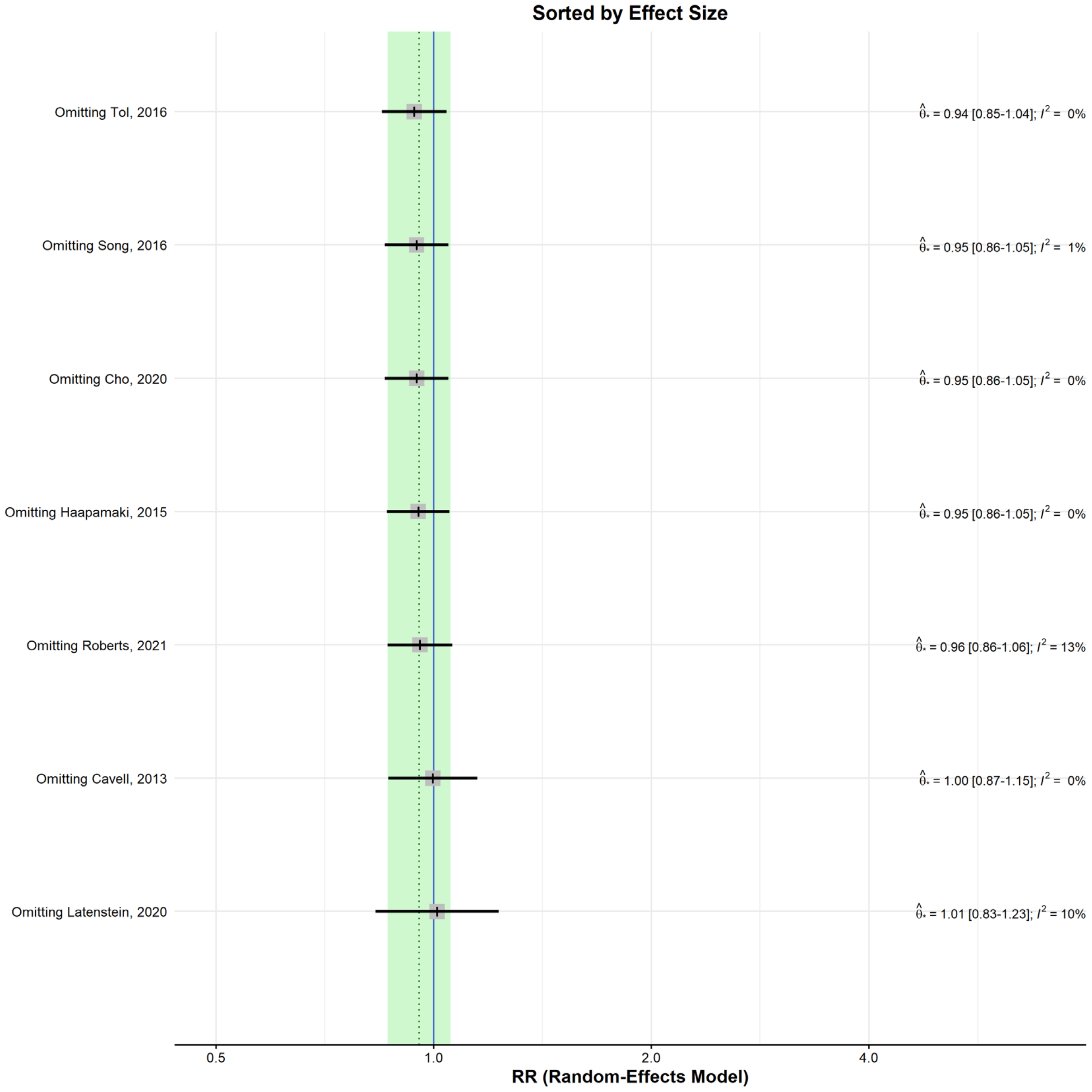

Fig. Leave-one-out sensitivity analysis plot for selected studies for Overall complication rate (Clavien - Dindo) treatment

The heterogeneity was assessed, and we found an I2 of 0% (95% CI 0% - 70.8%) and the Q test for heterogeneity gave p=0.448.

The RR value (the RR of Overall complication rate (Clavien - Dindo) treatment in the PS group compared to the SEMS group) obtained with the meta-analysis was of -0.05 (95% CI -0.15 - 0.05), p=0.359 using the model with random effects.

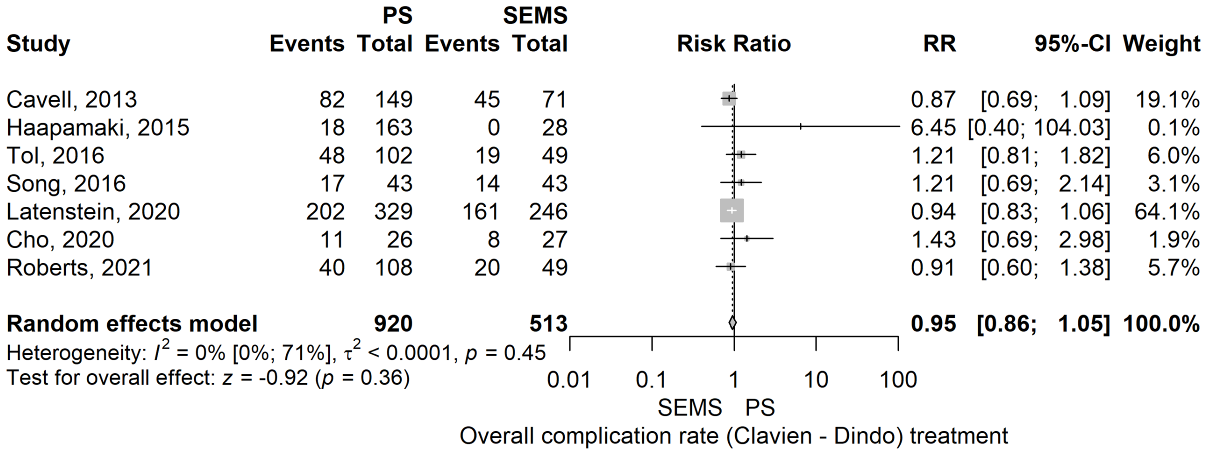

Fig. Forest plot for Overall complication rate (Clavien - Dindo) treatment, comparing PS with SEMS

Meta-analysis for Grade 1-2 treatment, comparing PS with SEMS

	datele.Year	datele.Study.name	numberCasesPerTreatment	numberCasesTreatment	numberCasesPerControl	numberCasesControl
5	2020	Latenstein, 2020	112	329	106	246
7	2020	Kuwatani, 2020	2	12	6	17
9	2022	Bademci, 2022	8	17	17	31

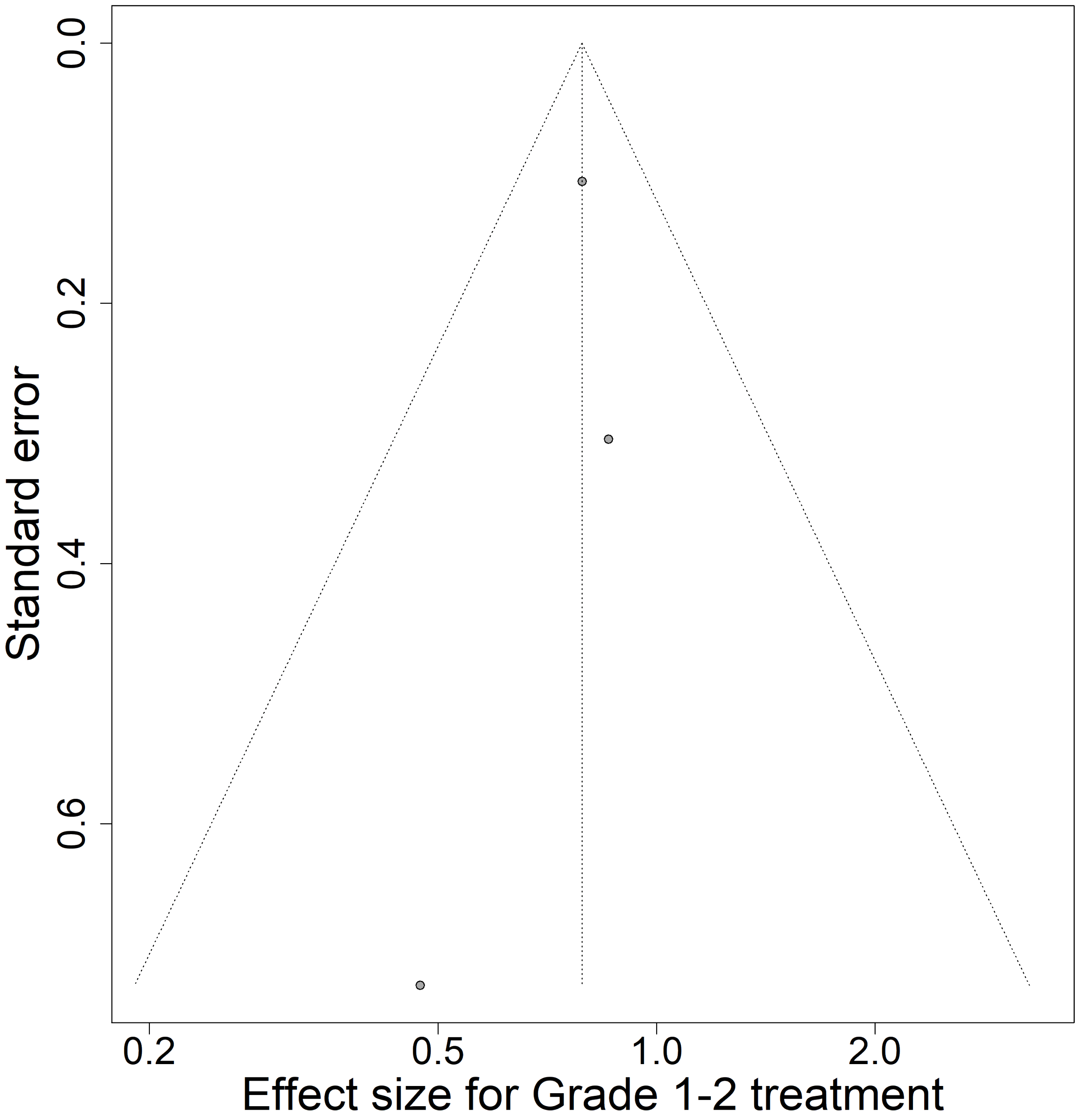

Fig. Funnel plot for Grade 1-2 treatment, comparing PS with SEMS

The funnel plot for Grade 1-2 treatment, comparing PS with SEMS is shown in figure _.

The publication bias test gave a p=0.63.

Influence studies: Omitting Latenstein, 2020; Omitting Kuwatani, 2020; Omitting Bademci, 2022 - no; no; no

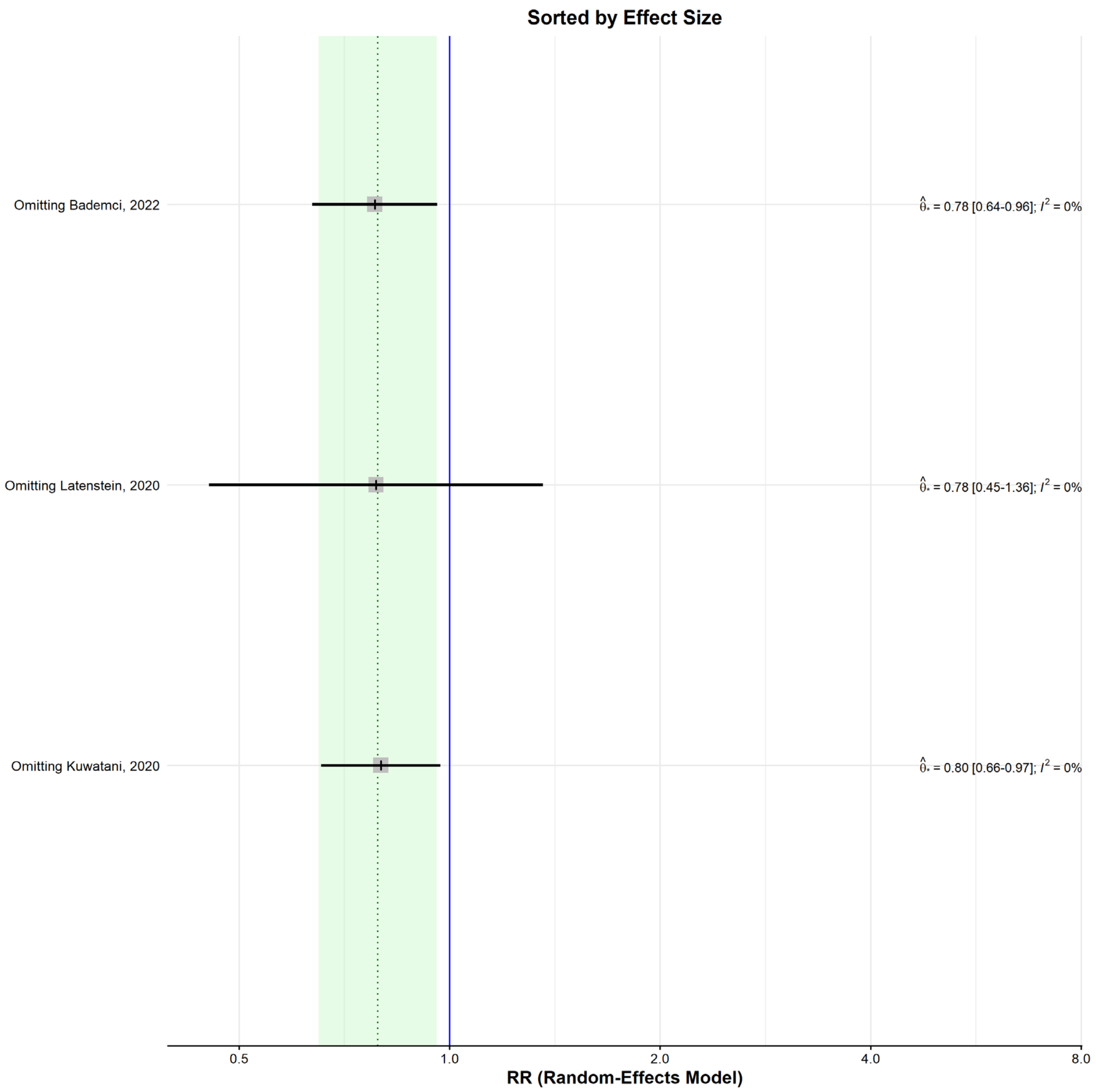

Fig. Leave-one-out sensitivity analysis plot for selected studies for Grade 1-2 treatment

The heterogeneity was assessed, and we found an I2 of 0% (95% CI 0% - 89.6%) and the Q test for heterogeneity gave p=0.749.

The RR value (the RR of Grade 1-2 treatment in the PS group compared to the SEMS group) obtained with the meta-analysis was of -0.24 (95% CI -0.43 - -0.04), p=0.017 using the model with random effects.

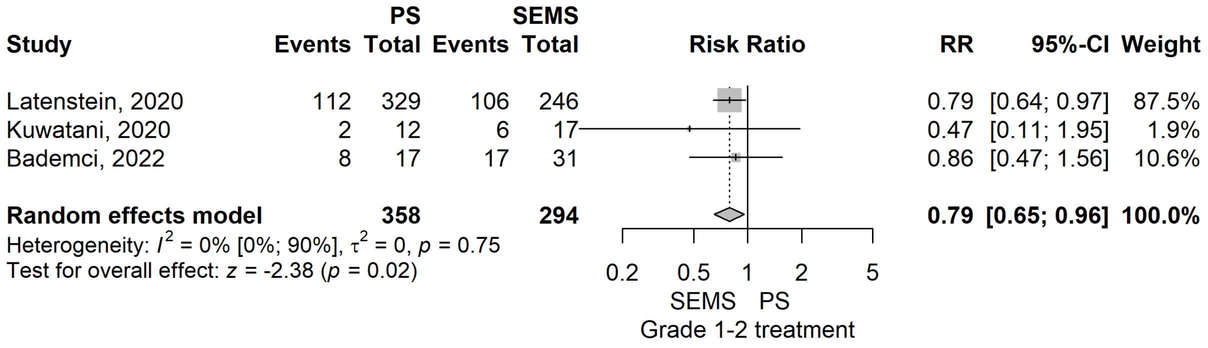

Fig. Forest plot for Grade 1-2 treatment, comparing PS with SEMS

Meta-analysis for Grade >=3 treatment, comparing PS with SEMS

	datele.Year	datele.Study.name	numberCasesPerTreatment	numberCasesTreatment	numberCasesPerControl	numberCasesControl
1	2013	Cavell, 2013	30	149	12	71
5	2020	Latenstein, 2020	90	329	55	246
7	2020	Kuwatani, 2020	4	12	2	17
9	2022	Bademci, 2022	3	17	5	31

2

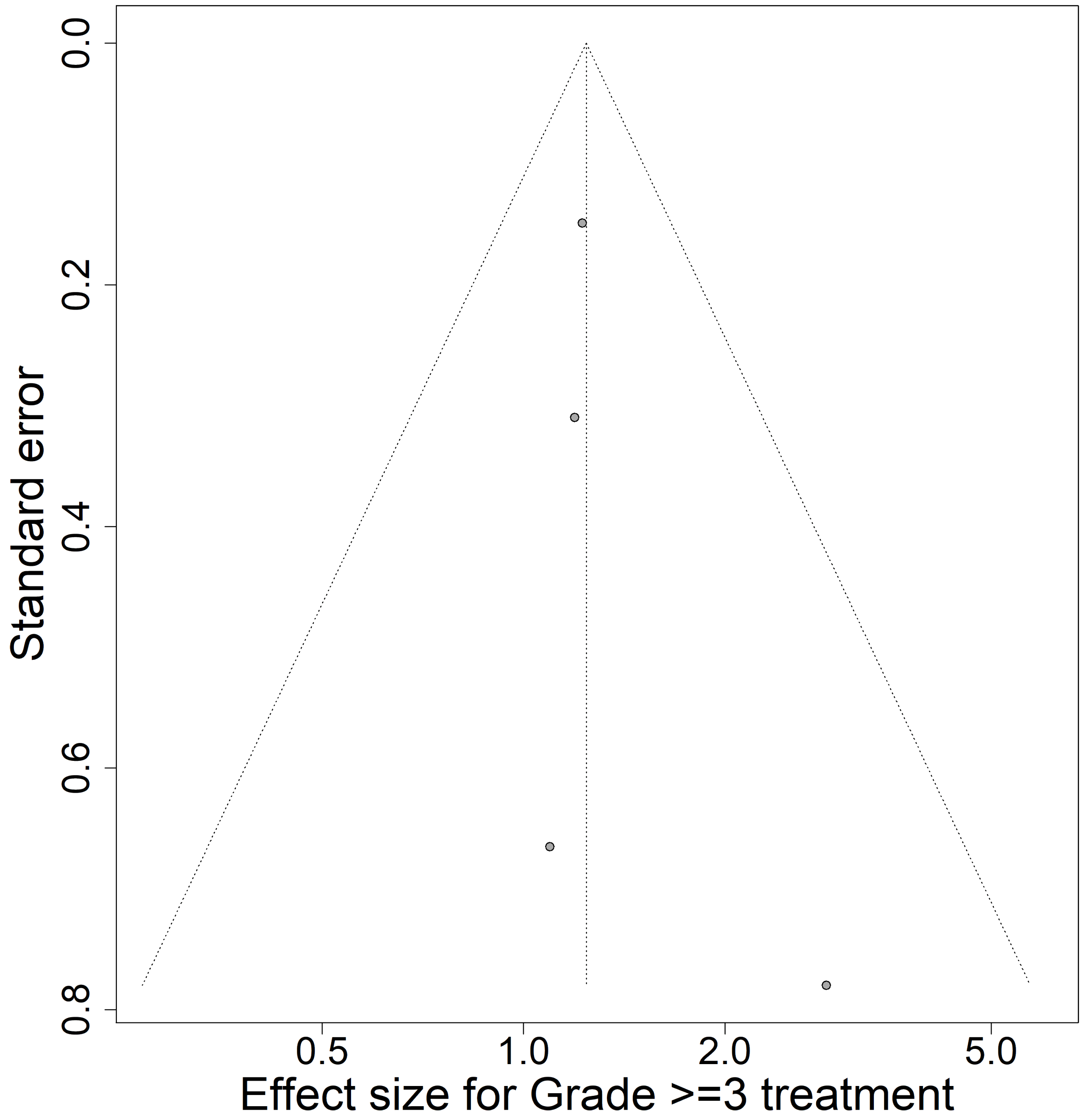

Fig. Funnel plot for Grade >=3 treatment, comparing PS with SEMS

The funnel plot for Grade >=3 treatment, comparing PS with SEMS is shown in figure _.

The publication bias test gave a p=0.478.

Influence studies: Omitting Cavell, 2013; Omitting Latenstein, 2020; Omitting Kuwatani, 2020; Omitting Bademci, 2022 - no; yes; no; no

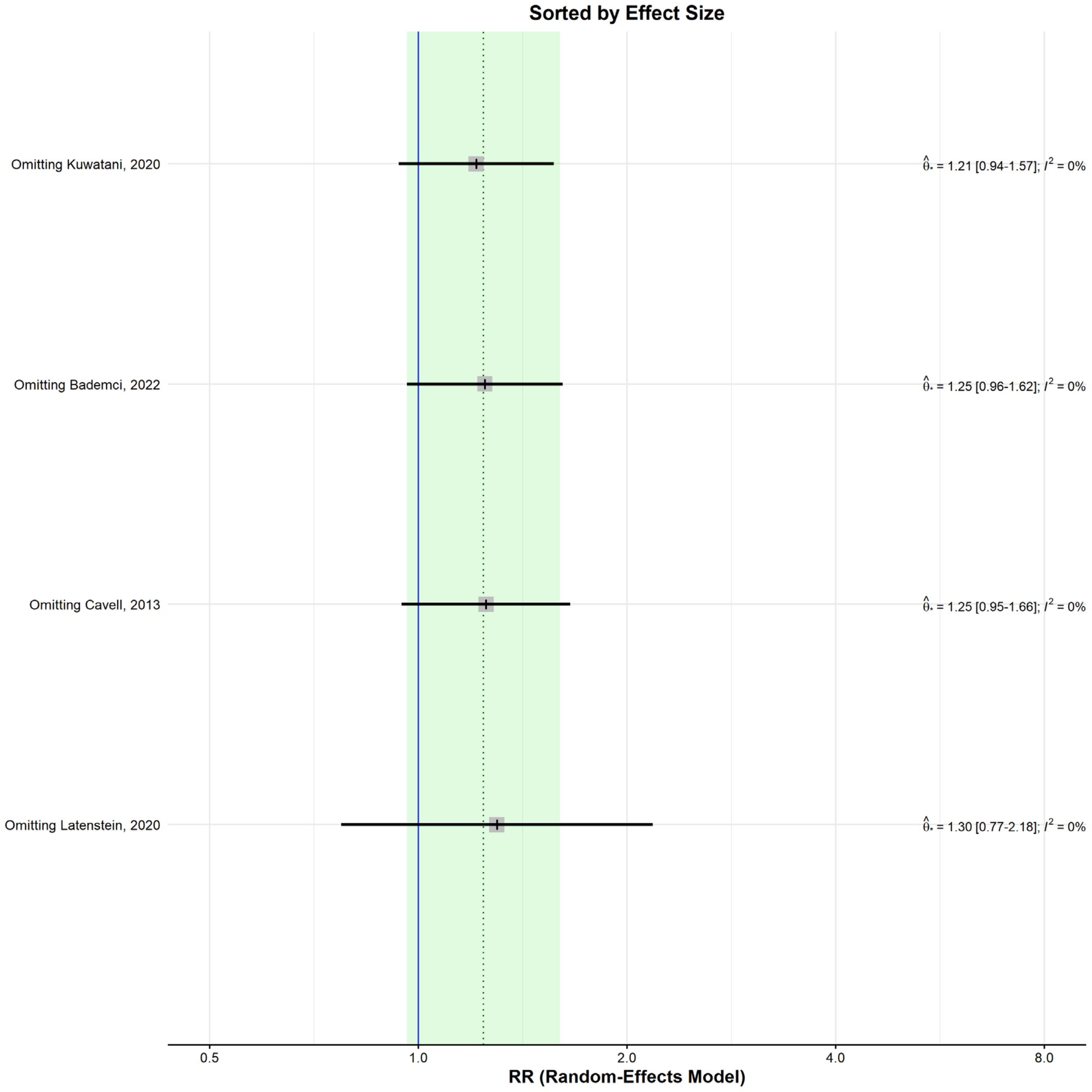

Fig. Leave-one-out sensitivity analysis plot for selected studies for Grade >=3 treatment

The heterogeneity was assessed, and we found an I2 of 0% (95% CI 0% - 84.7%) and the Q test for heterogeneity gave p=0.757.

The RR value (the RR of Grade >=3 treatment in the PS group compared to the SEMS group) obtained with the meta-analysis was of 0.22 (95% CI -0.04 - 0.47), p=0.096 using the model with random effects.

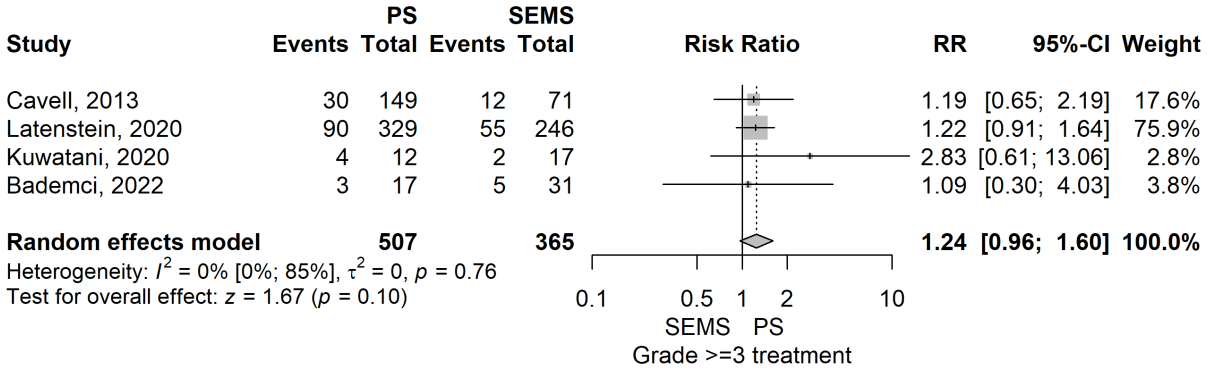

Fig. Forest plot for Grade >=3 treatment, comparing PS with SEMS

Meta-analysis for Infectious complications treatment, comparing PS with SEMS

	datele.Year	datele.Study.name	numberCasesPerTreatment	numberCasesTreatment	numberCasesPerControl	numberCasesControl
2	2015	Haapamaki, 2015	23	163	5	28

2

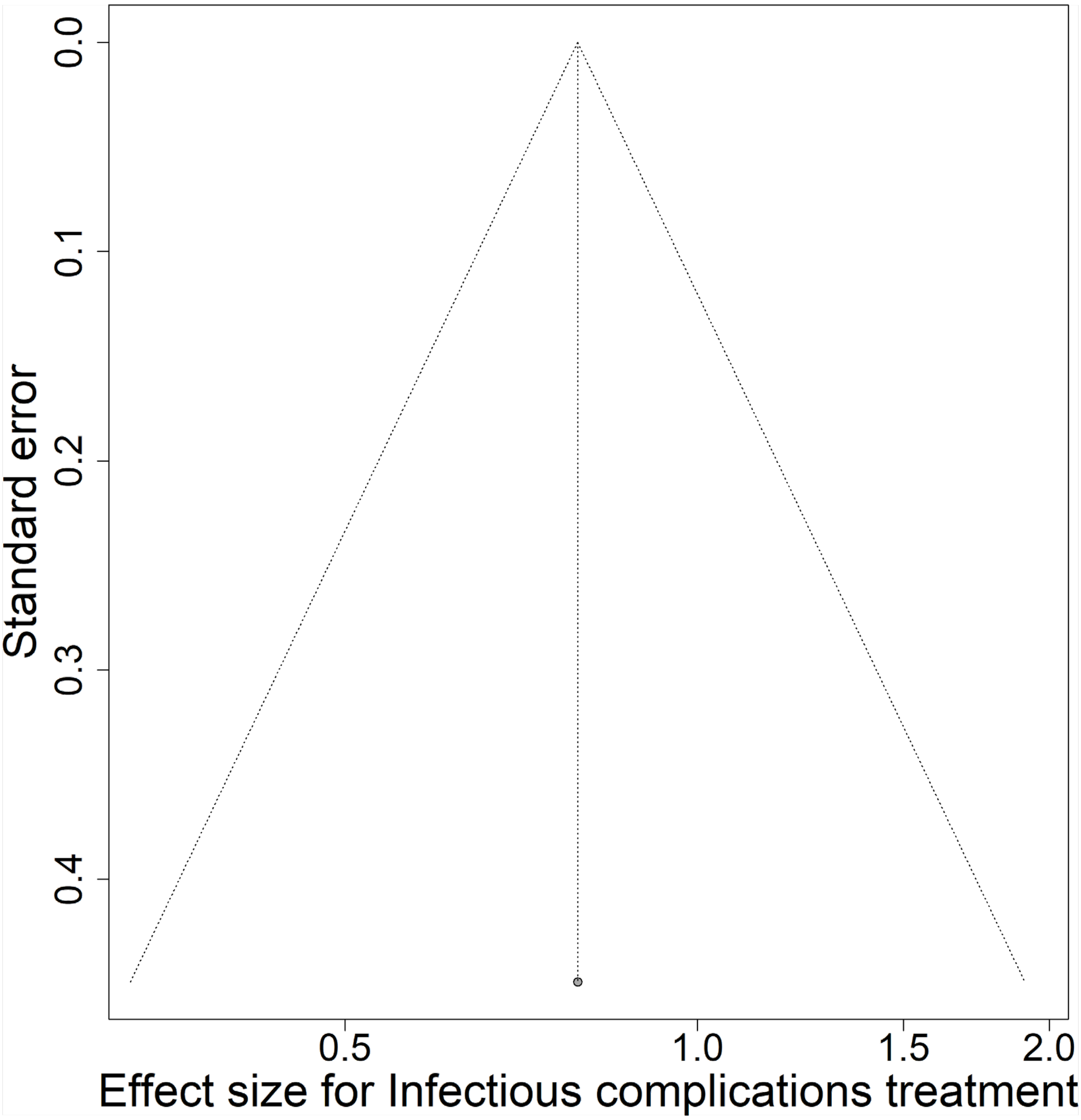

Fig. Funnel plot for Infectious complications treatment, comparing PS with SEMS

The funnel plot for Infectious complications treatment, comparing PS with SEMS is shown in figure _.

The publication bias test cannot be computed since there are not at least three studies.

The heterogeneity cannot be assessed since there are not at least two studies.

The RR value (the RR of Infectious complications treatment in the PS group compared to the SEMS group) obtained with the meta-analysis was of -0.24 (95% CI -1.12 - 0.64), p=0.6 using the model with random effects.

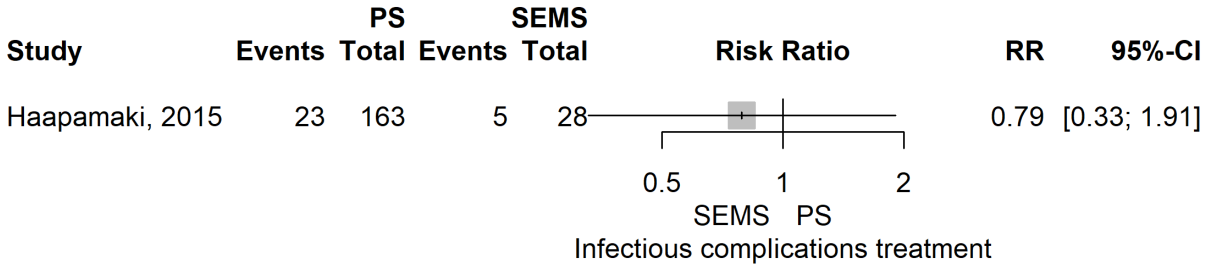

Fig. Forest plot for Infectious complications treatment, comparing PS with SEMS

Meta-analysis for Sepsis treatment, comparing PS with SEMS

	datele.Year	datele.Study.name	numberCasesPerTreatment	numberCasesTreatment	numberCasesPerControl	numberCasesControl
9	2022	Bademci, 2022	3	17	8	31
2						

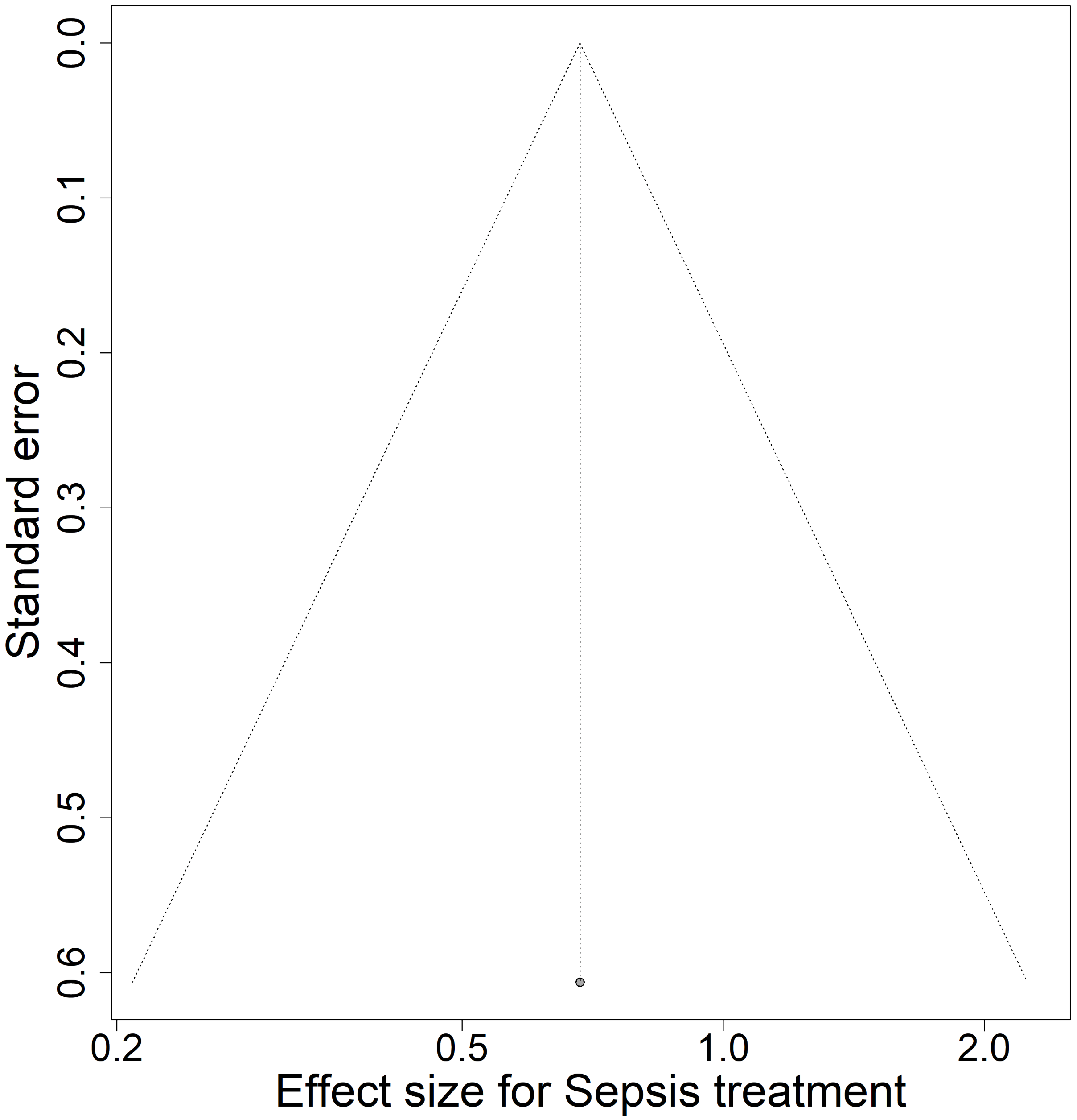

Fig. Funnel plot for Sepsis treatment, comparing PS with SEMS

The funnel plot for Sepsis treatment, comparing PS with SEMS is shown in figure _.

The publication bias test cannot be computed since there are not at least three studies.

The heterogeneity cannot be assessed since there are not at least two studies.

The RR value (the RR of Sepsis treatment in the PS group compared to the SEMS group) obtained with the meta-analysis was of -0.38 (95% CI -1.57 - 0.81), p=0.531 using the model with random effects.

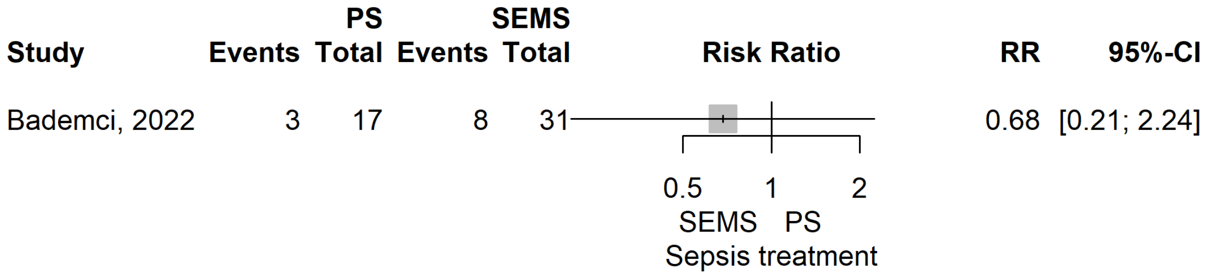

Fig. Forest plot for Sepsis treatment, comparing PS with SEMS

Meta-analysis for Intraabdominal abcess treatment, comparing PS with SEMS

	datele.Year	datele.Study.name	numberCasesPerTreatment	numberCasesTreatment	numberCasesPerControl	numberCasesControl
3	2016	Tol, 2016	2	102	2	49
6	2020	Cho, 2020	2	26	1	27
8	2021	Roberts, 2021	14	108	8	49

2

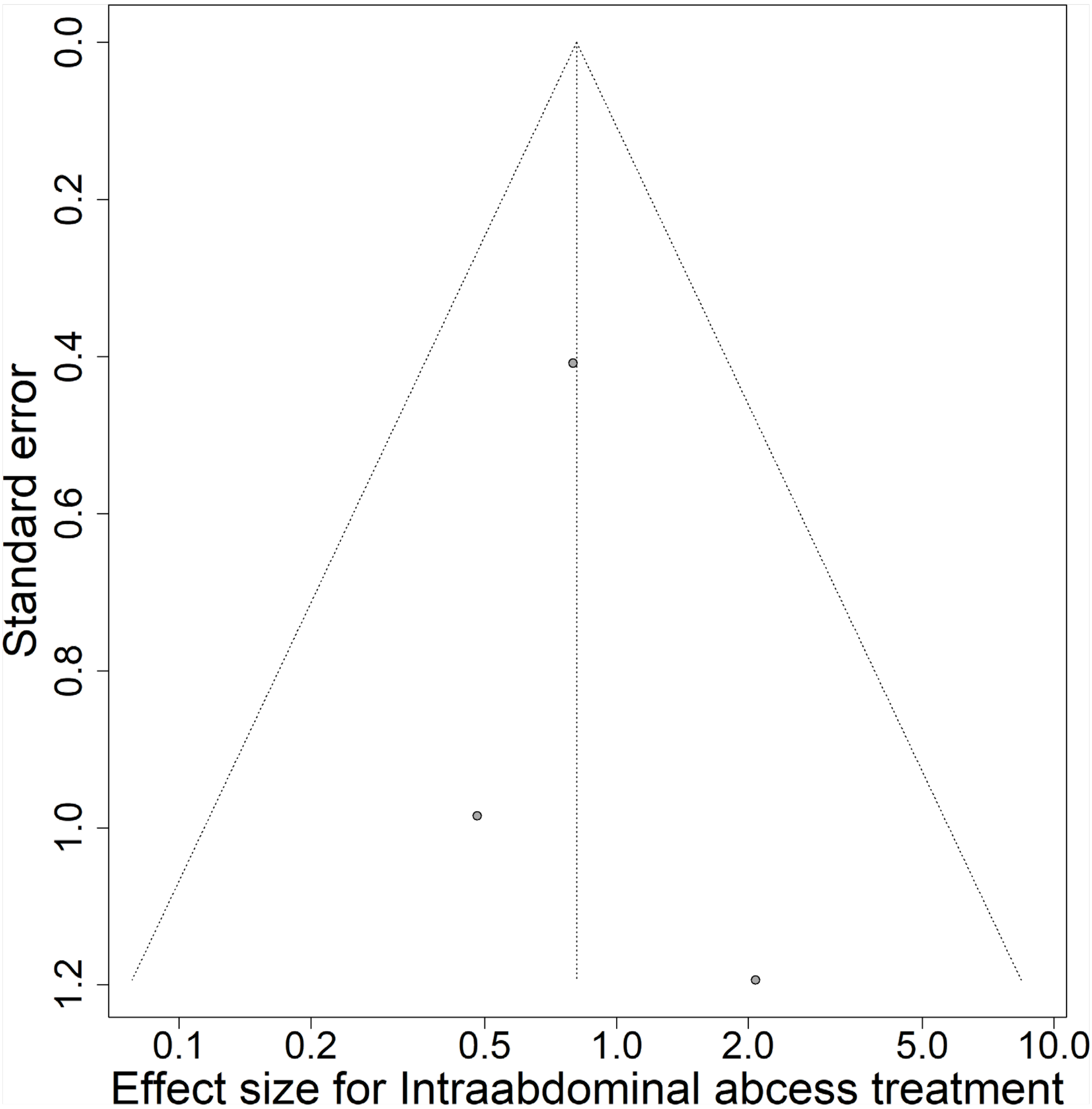

Fig. Funnel plot for Intraabdominal abcess treatment, comparing PS with SEMS

The funnel plot for Intraabdominal abcess treatment, comparing PS with SEMS is shown in figure _.

The publication bias test gave a p=0.819.

Influence studies: Omitting Tol, 2016; Omitting Cho, 2020; Omitting Roberts, 2021 - no; no; no

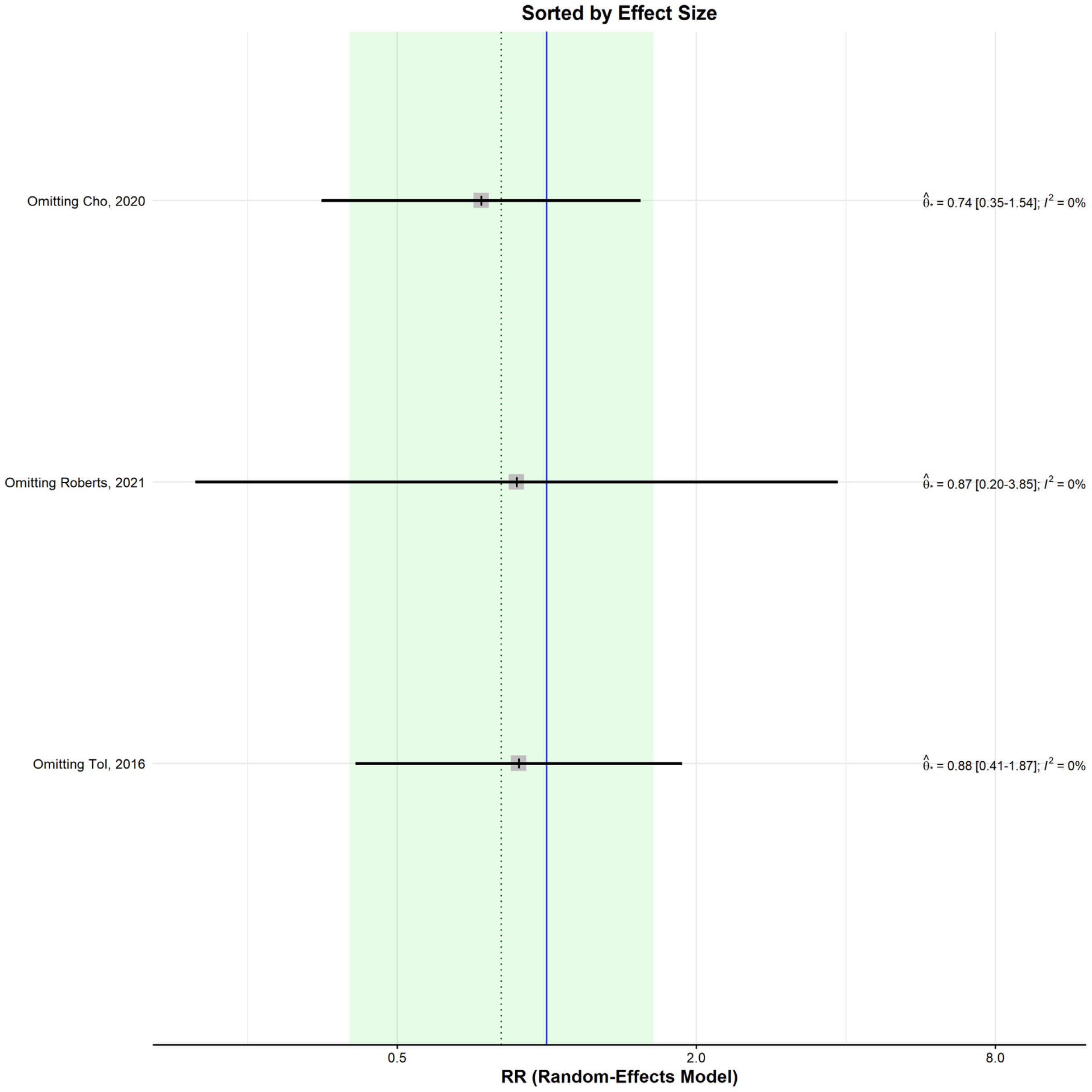

Fig. Leave-one-out sensitivity analysis plot for selected studies for Intraabdominal abcess treatment

The heterogeneity was assessed, and we found an I2 of 0% (95% CI 0% - 89.6%) and the Q test for heterogeneity gave p=0.636.

The RR value (the RR of Intraabdominal abcess treatment in the PS group compared to the SEMS group) obtained with the meta-analysis was of -0.21 (95% CI -0.92 - 0.49), p=0.559 using the model with random effects.

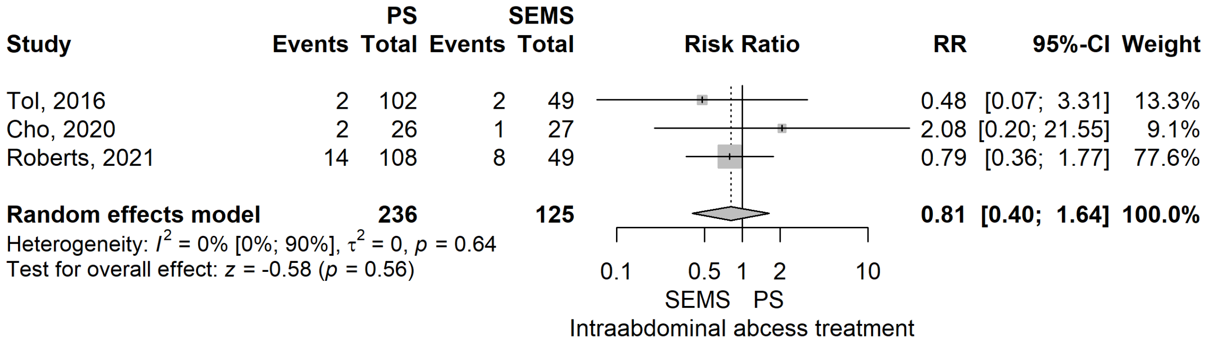

Fig. Forest plot for Intraabdominal abcess treatment, comparing PS with SEMS

Meta-analysis for Wound infections treatment, comparing PS with SEMS

	datele.Year	datele.Study.name	numberCasesPerTreatment	numberCasesTreatment	numberCasesPerControl	numberCasesControl
1	2013	Cavell, 2013	19	149	22	71
3	2016	Tol, 2016	13	102	7	49
4	2016	Song, 2016	2	43	4	43
5	2020	Latenstein, 2020	48	329	41	246
6	2020	Cho, 2020	1	26	2	27
8	2021	Roberts, 2021	14	108	11	49
9	2022	Bademci, 2022	3	17	7	31

2

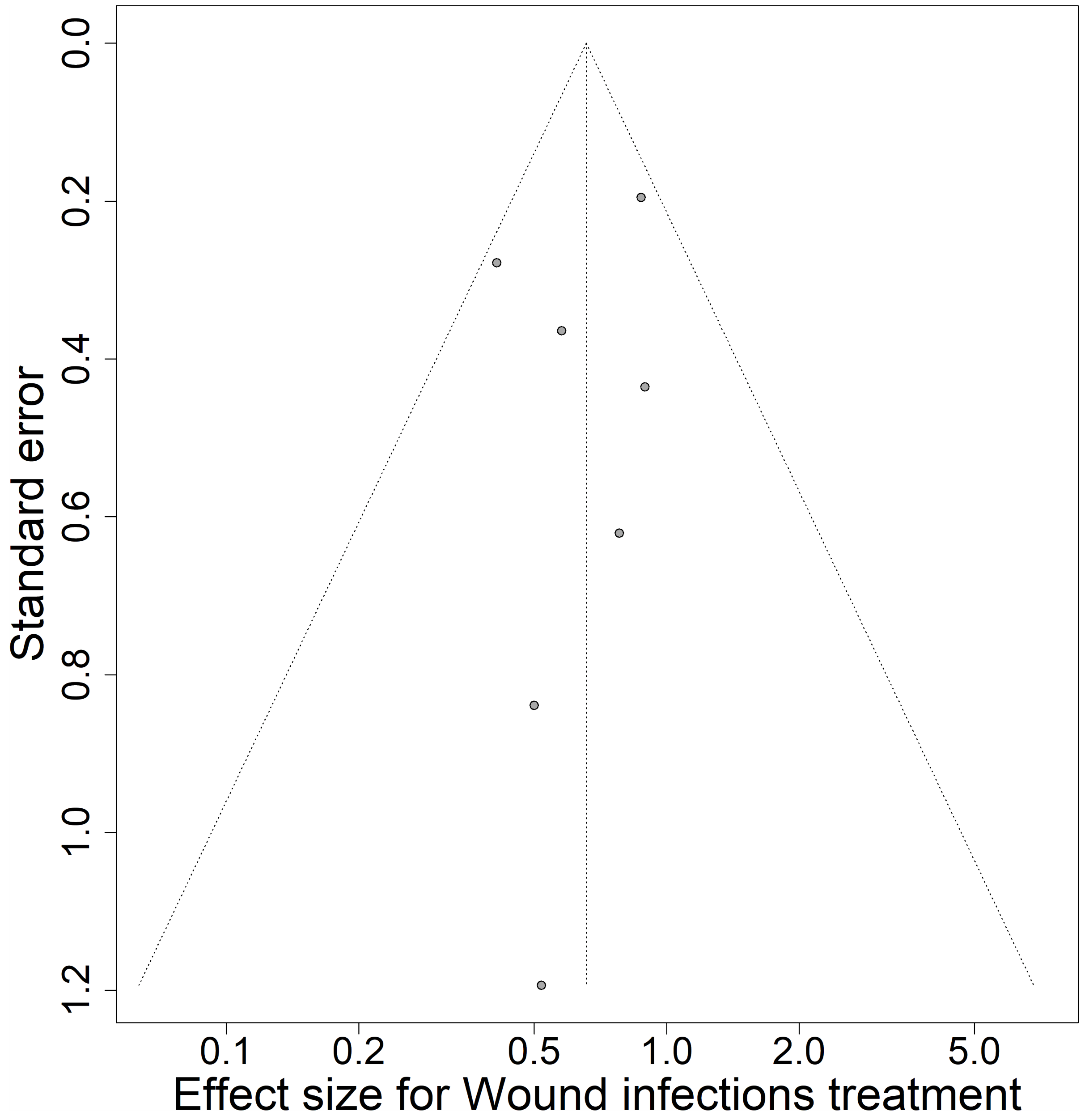

Fig. Funnel plot for Wound infections treatment, comparing PS with SEMS

The funnel plot for Wound infections treatment, comparing PS with SEMS is shown in figure _.

The publication bias test gave a p=0.612.

Influence studies: Omitting Cavell, 2013; Omitting Tol, 2016; Omitting Song, 2016; Omitting Latenstein, 2020; Omitting Cho, 2020; Omitting Roberts, 2021; Omitting Bademci, 2022 - yes; no; no; yes; no; no; no

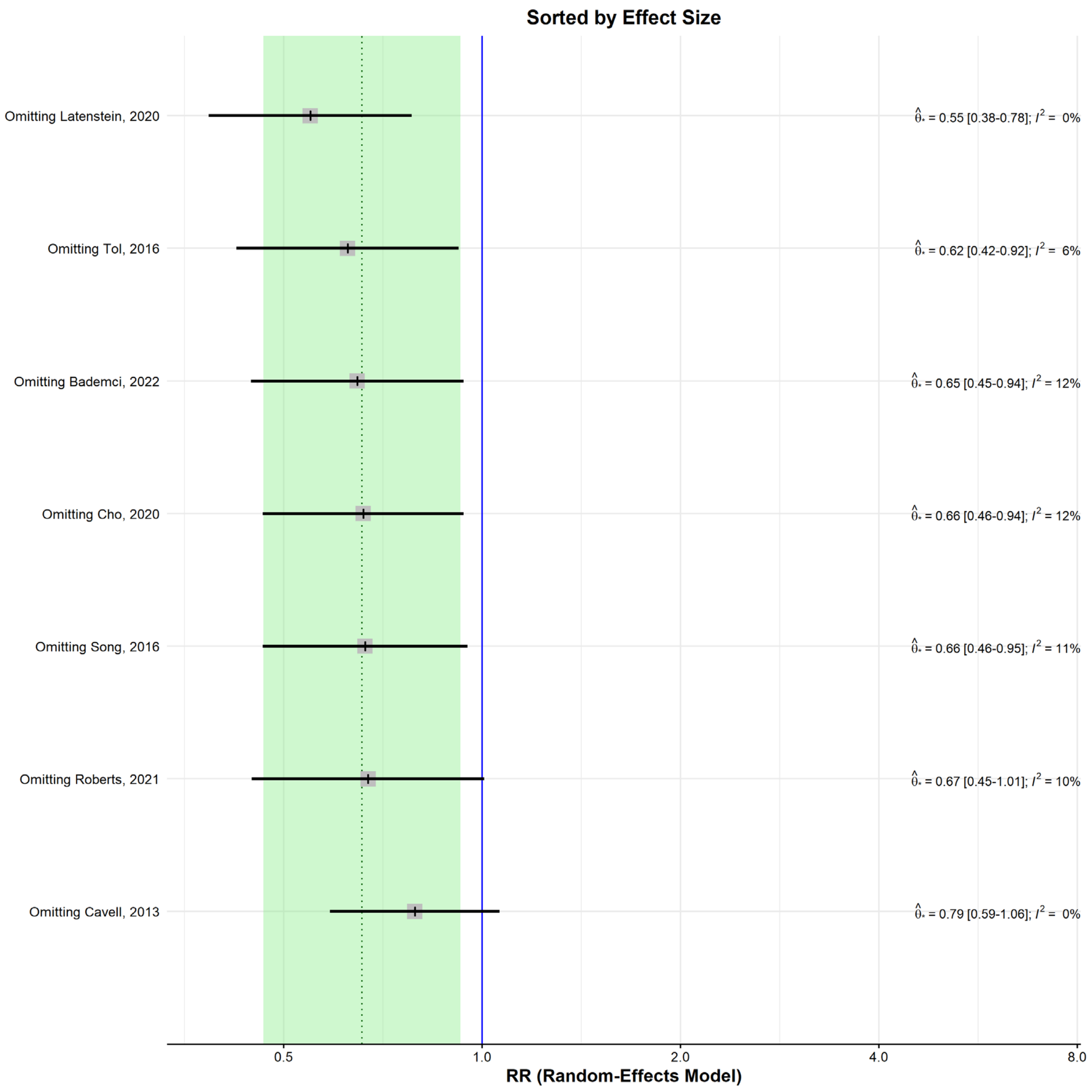

Fig. Leave-one-out sensitivity analysis plot for selected studies for Wound infections treatment

The heterogeneity was assessed, and we found an I2 of 0% (95% CI 0% - 70.8%) and the Q test for heterogeneity gave p=0.45.

The RR value (the RR of Wound infections treatment in the PS group compared to the SEMS group) obtained with the meta-analysis was of -0.42 (95% CI -0.76 - -0.08), p=0.017 using the model with random effects.

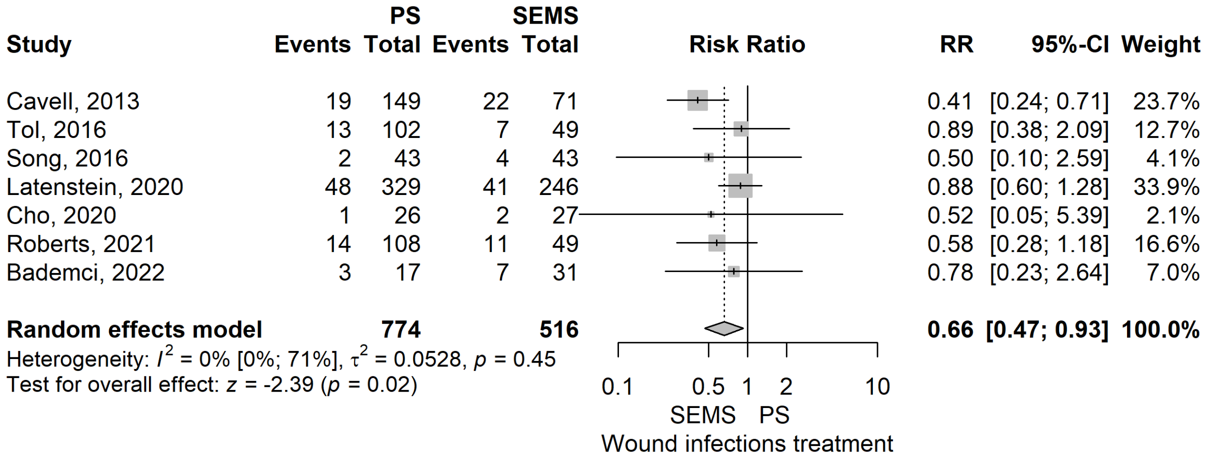

Fig. Forest plot for Wound infections treatment, comparing PS with SEMS

Meta-analysis for PPH treatment, comparing PS with SEMS

	datele.Year	datele.Study.name	numberCasesPerTreatment	numberCasesTreatment	numberCasesPerControl	numberCasesControl
3	2016	Tol, 2016	2	102	2	49
4	2016	Song, 2016	1	43	1	43
5	2020	Latenstein, 2020	23	329	11	246
6	2020	Cho, 2020	1	26	0	27

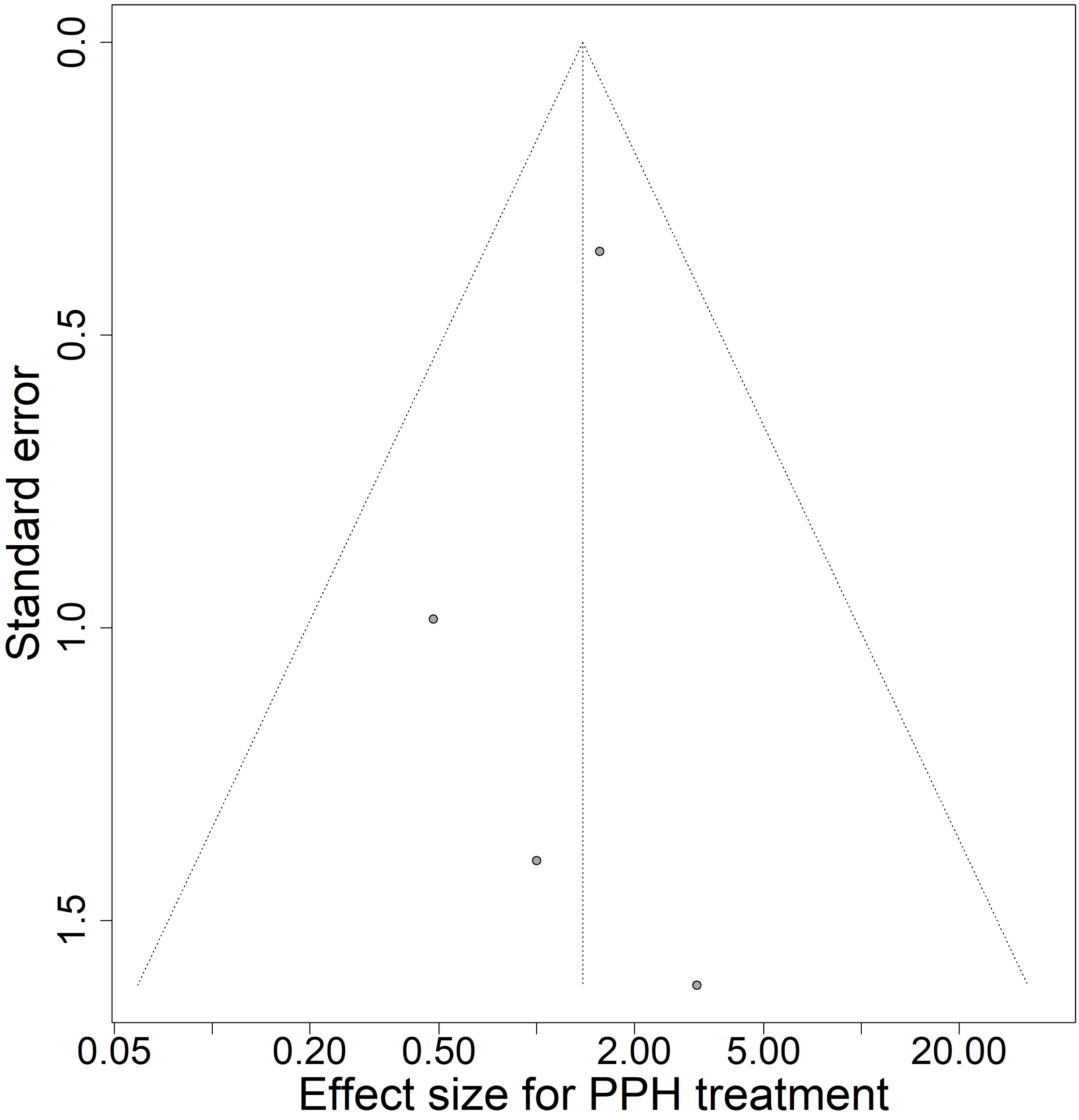

Fig. Funnel plot for PPH treatment, comparing PS with SEMS

The funnel plot for PPH treatment, comparing PS with SEMS is shown in figure _.

The publication bias test gave a p=0.671.

Influence studies: Omitting Tol, 2016; Omitting Song, 2016; Omitting Latenstein, 2020; Omitting Cho, 2020 - no; no; yes; no

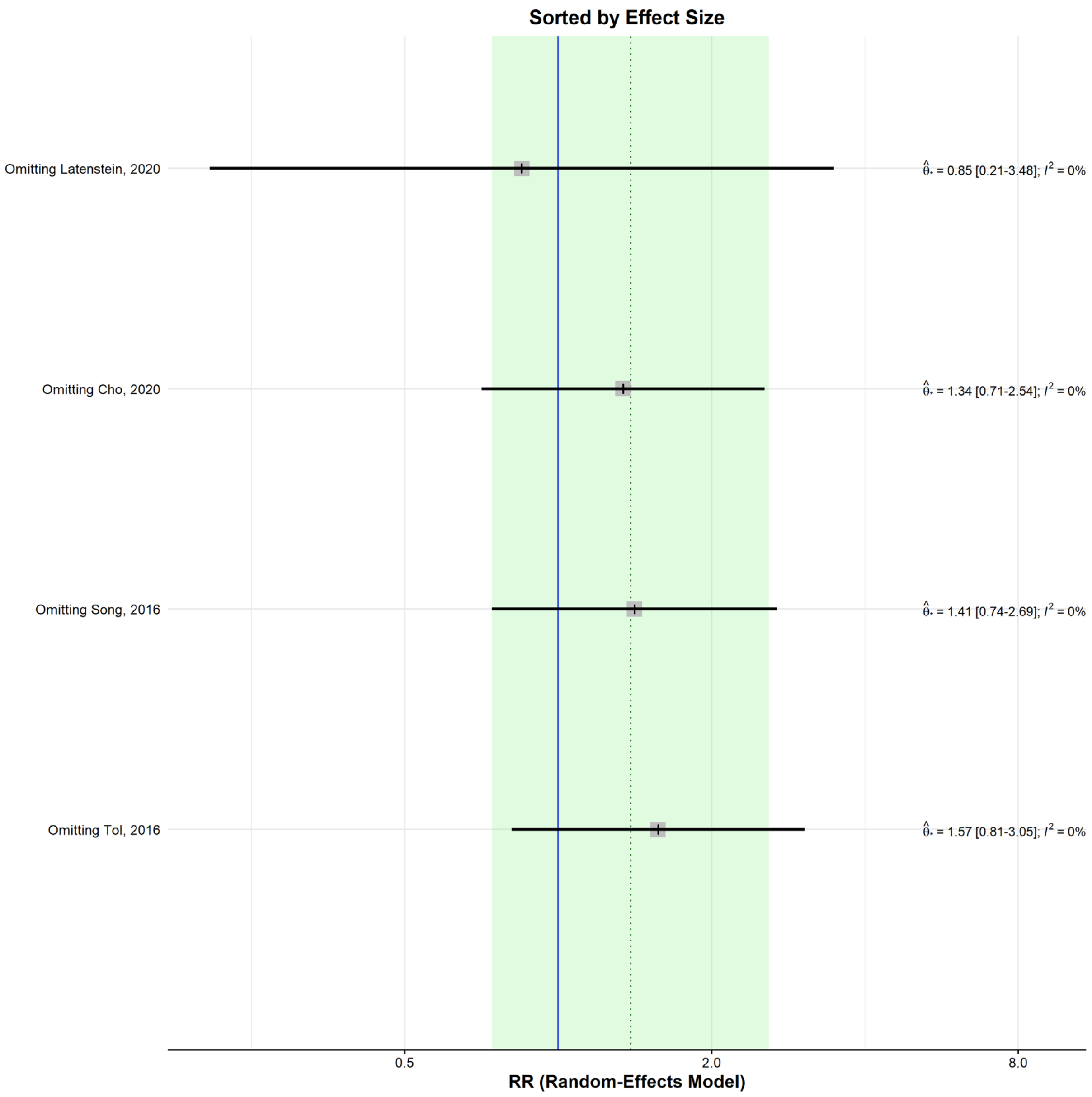

Fig. Leave-one-out sensitivity analysis plot for selected studies for PPH treatment

The heterogeneity was assessed, and we found an I2 of 0% (95% CI 0% - 84.7%) and the Q test for heterogeneity gave p=0.664.

The RR value (the RR of PPH treatment in the PS group compared to the SEMS group) obtained with the meta-analysis was of 0.33 (95% CI -0.3 - 0.95), p=0.307 using the model with random effects.

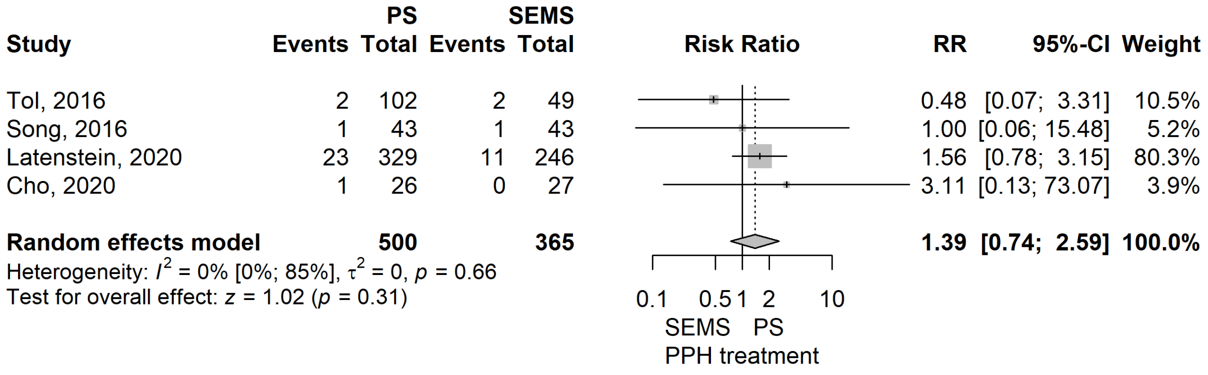

Fig. Forest plot for PPH treatment, comparing PS with SEMS

Meta-analysis for Chyle leak treatment, comparing PS with SEMS

	datele.Year	datele.Study.name	numberCasesPerTreatment	numberCasesTreatment	numberCasesPerControl	numberCasesControl
5	2020	Latenstein, 2020	25	329	18	246

2

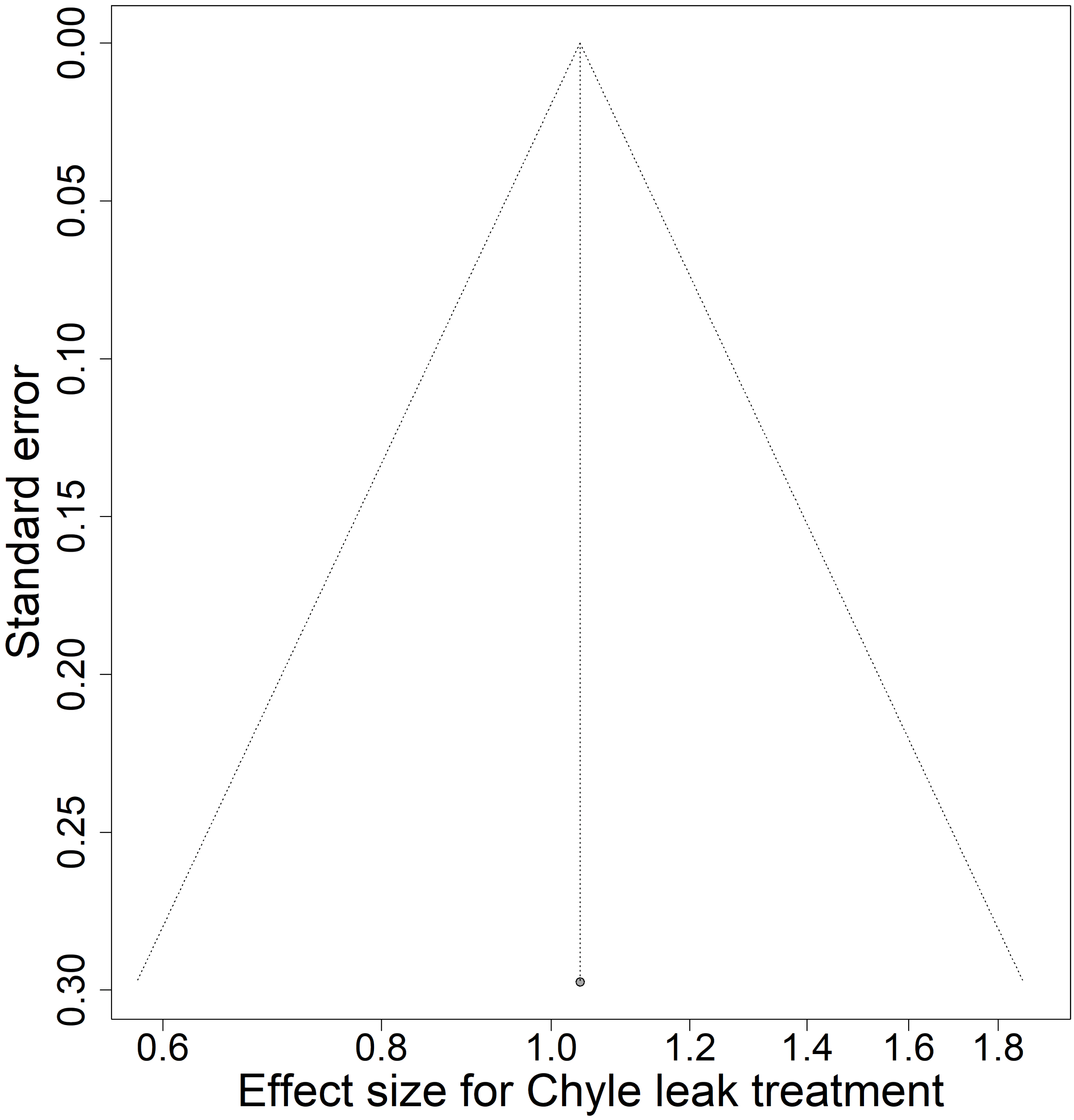

Fig. Funnel plot for Chyle leak treatment, comparing PS with SEMS

The funnel plot for Chyle leak treatment, comparing PS with SEMS is shown in figure _.

The publication bias test cannot be computed since there are not at least three studies.

The heterogeneity cannot be assessed since there are not at least two studies.

The RR value (the RR of Chyle leak treatment in the PS group compared to the SEMS group) obtained with the meta-analysis was of 0.04 (95% CI -0.55 - 0.62), p=0.899 using the model with random effects.

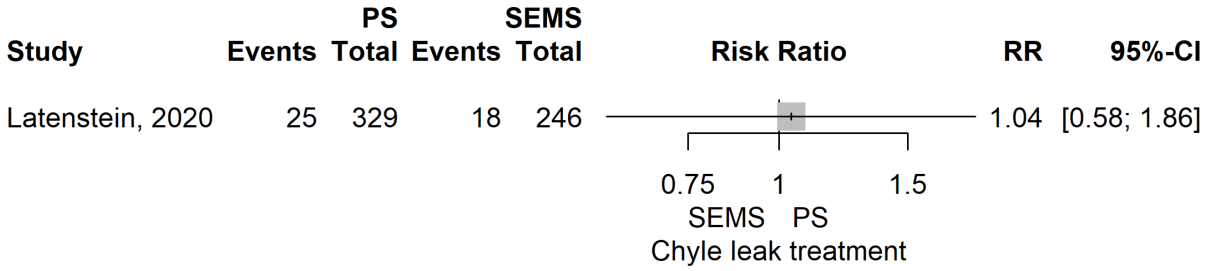

Fig. Forest plot for Chyle leak treatment, comparing PS with SEMS

Meta-analysis for POBF treatment, comparing PS with SEMS

	datele.Year	datele.Study.name	numberCasesPerTreatment	numberCasesTreatment	numberCasesPerControl	numberCasesControl
1	2013	Cavell, 2013	1	149	0	71
3	2016	Tol, 2016	1	102	1	49
5	2020	Latenstein, 2020	12	329	13	246
6	2020	Cho, 2020	2	26	0	27

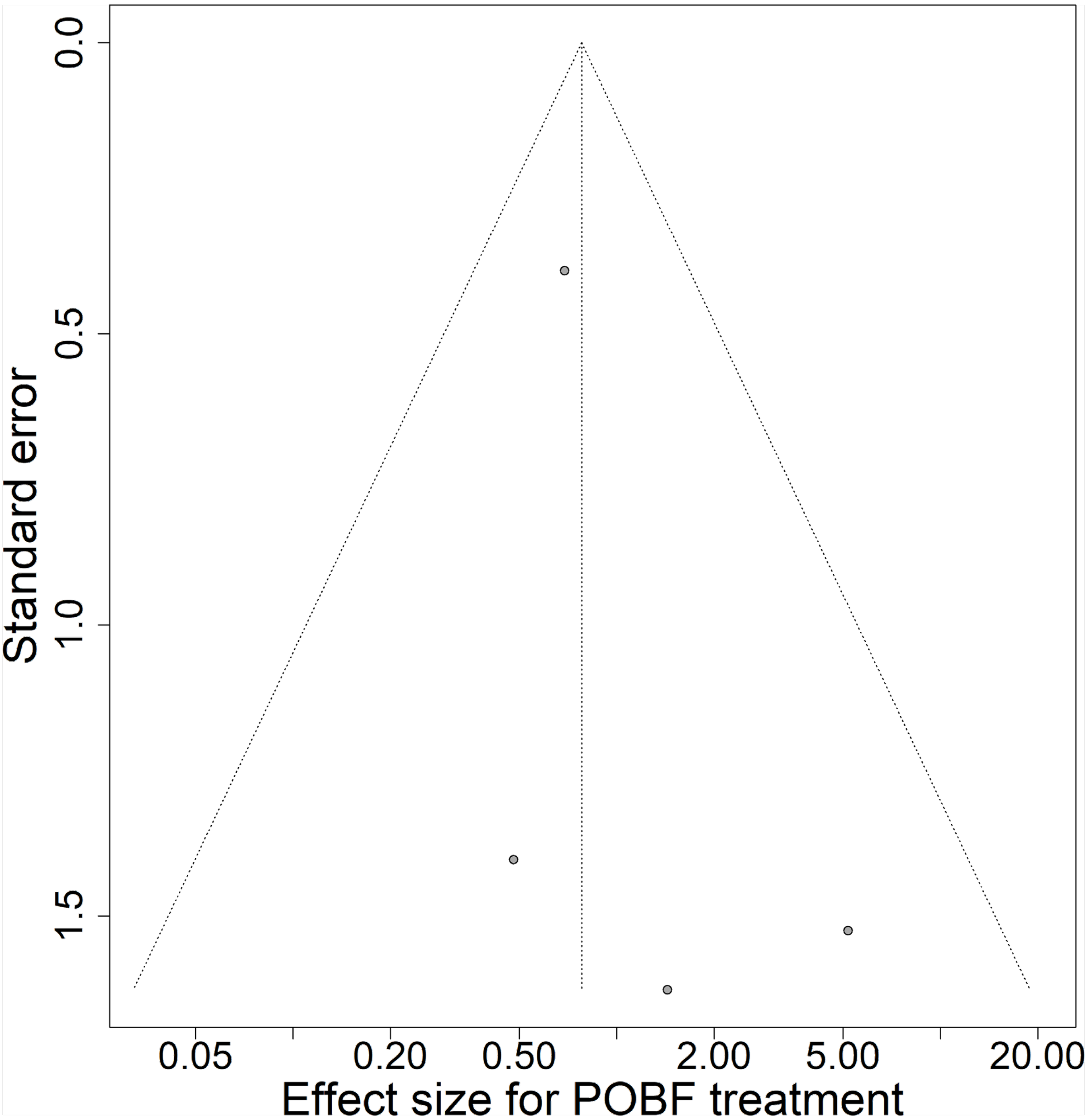

Fig. Funnel plot for POBF treatment, comparing PS with SEMS

The funnel plot for POBF treatment, comparing PS with SEMS is shown in figure _.

The publication bias test gave a p=0.407.

Influence studies: Omitting Cavell, 2013; Omitting Tol, 2016; Omitting Latenstein, 2020; Omitting Cho, 2020 - no; no; yes; no

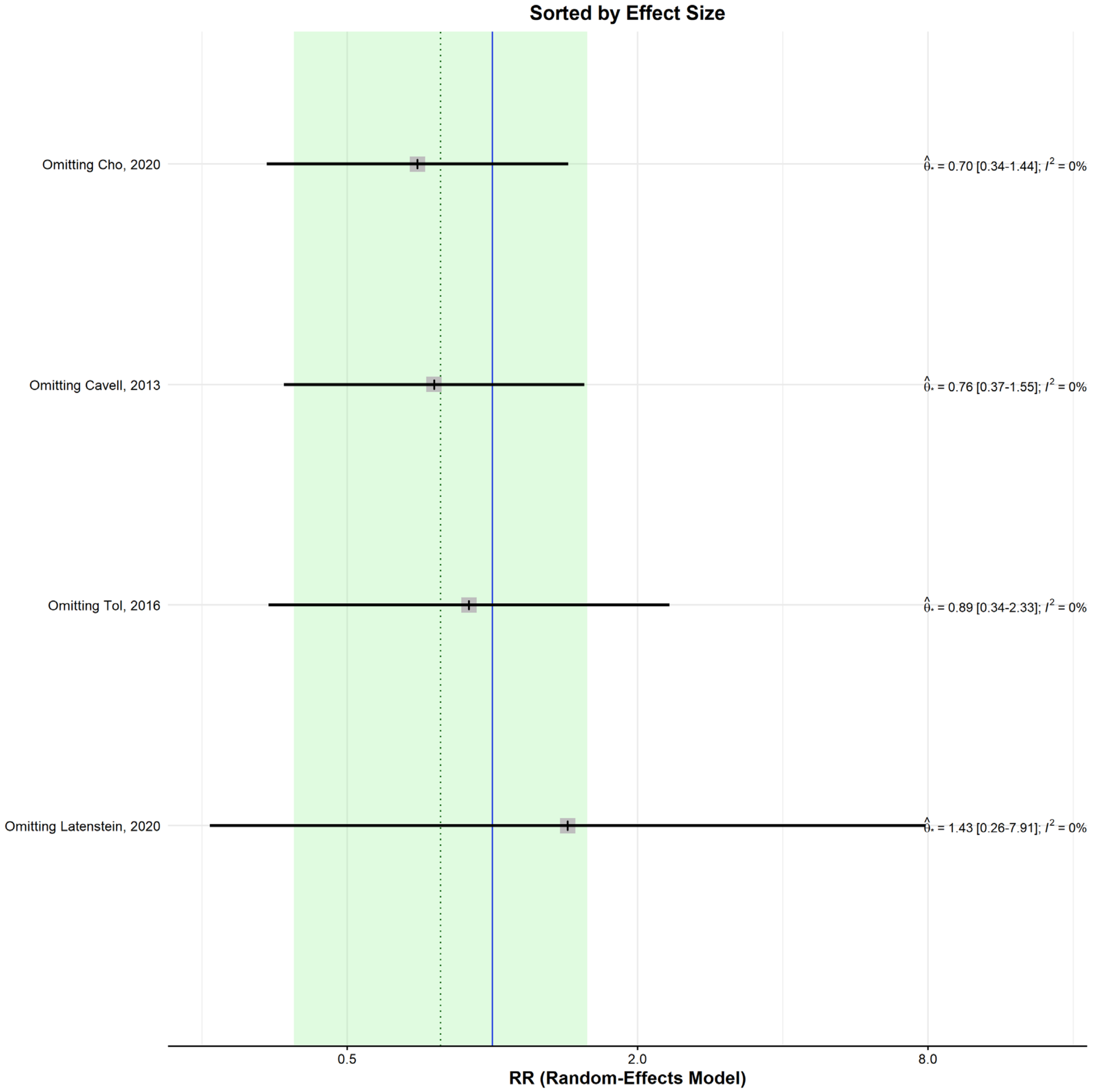

Fig. Leave-one-out sensitivity analysis plot for selected studies for POBF treatment

The heterogeneity was assessed, and we found an I2 of 0% (95% CI 0% - 84.7%) and the Q test for heterogeneity gave p=0.593.

The RR value (the RR of POBF treatment in the PS group compared to the SEMS group) obtained with the meta-analysis was of -0.25 (95% CI -0.95 - 0.45), p=0.487 using the model with random effects.

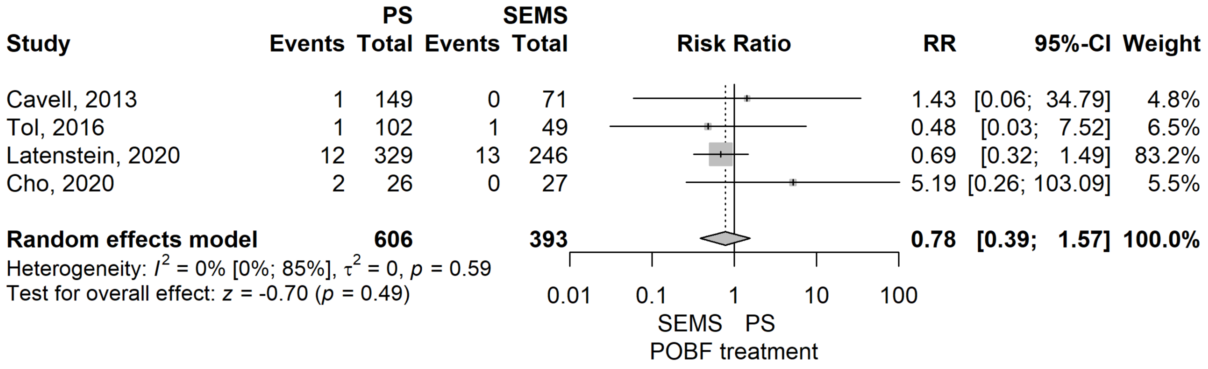

Fig. Forest plot for POBF treatment, comparing PS with SEMS

Meta-analysis for POPF treatment, comparing PS with SEMS

	datele.Year	datele.Study.name	numberCasesPerTreatment	numberCasesTreatment	numberCasesPerControl	numberCasesControl
1	2013	Cavell, 2013	19	149	5	71
3	2016	Tol, 2016	8	102	1	49
4	2016	Song, 2016	3	43	3	43
5	2020	Latenstein, 2020	61	329	24	246
6	2020	Cho, 2020	5	26	2	27
7	2020	Kuwatani, 2020	0	12	2	17
8	2021	Roberts, 2021	25	108	6	49
9	2022	Bademci, 2022	3	17	11	31

2

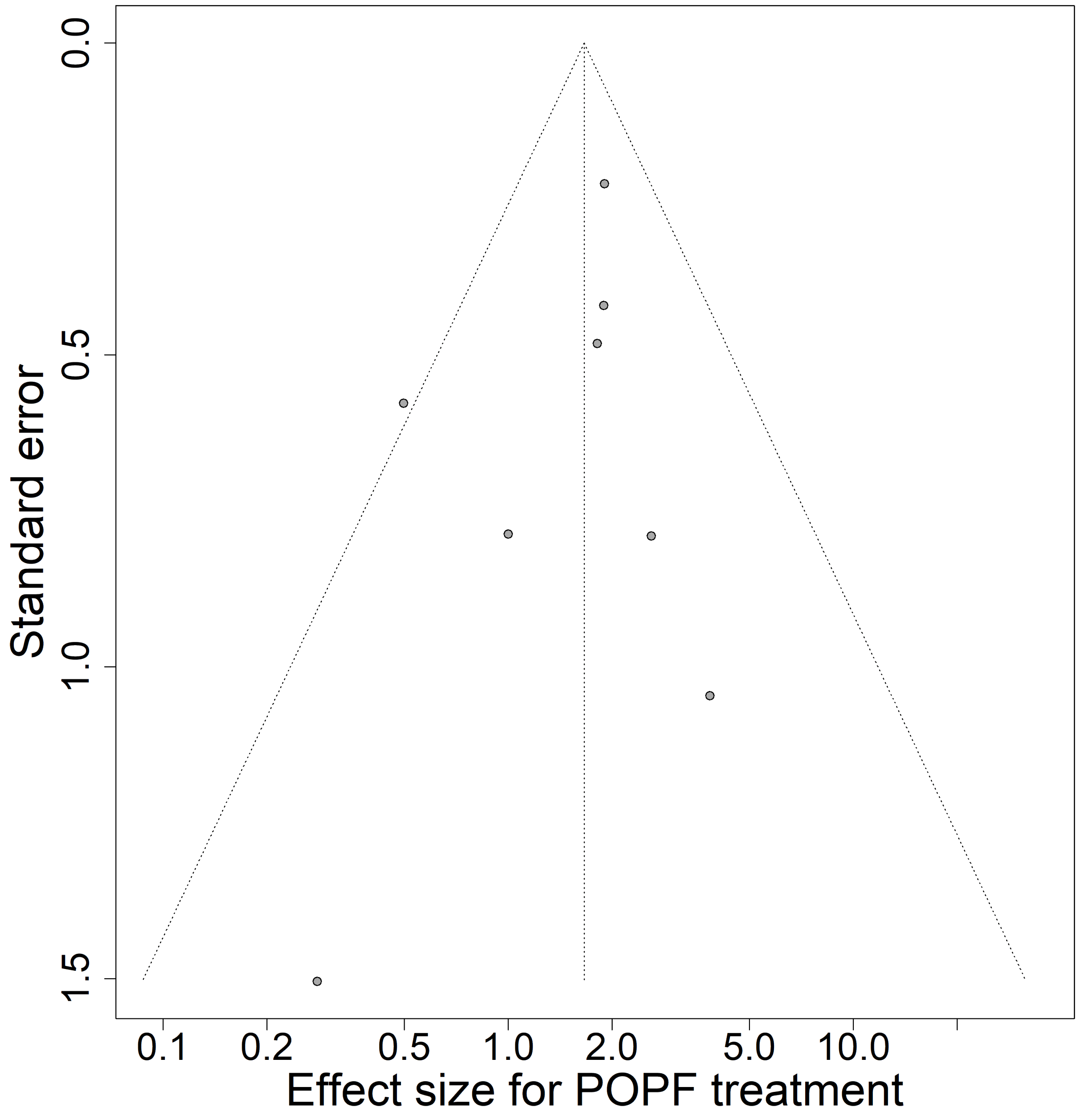

Fig. Funnel plot for POPF treatment, comparing PS with SEMS

The funnel plot for POPF treatment, comparing PS with SEMS is shown in figure _.

The publication bias test gave a p=0.375.

Influence studies: Omitting Cavell, 2013; Omitting Tol, 2016; Omitting Song, 2016; Omitting Latenstein, 2020; Omitting Cho, 2020; Omitting Kuwatani, 2020; Omitting Roberts, 2021; Omitting Bademci, 2022 - no; no; no; yes; no; no; no; no

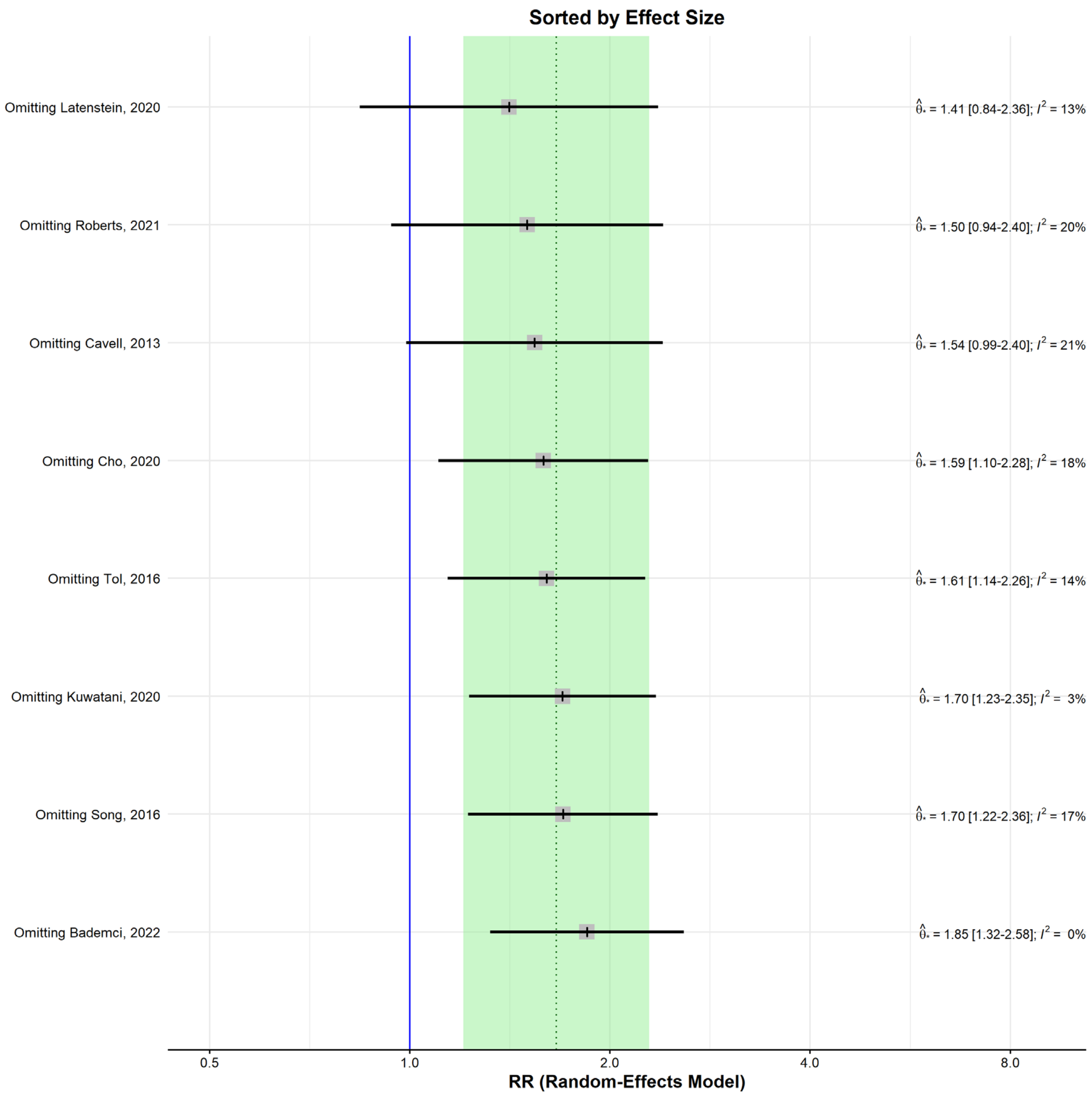

Fig. Leave-one-out sensitivity analysis plot for selected studies for POPF treatment

The heterogeneity was assessed, and we found an I2 of 8.2% (95% CI 0% - 70.2%) and the Q test for heterogeneity gave p=0.367.

The RR value (the RR of POPF treatment in the PS group compared to the SEMS group) obtained with the meta-analysis was of 0.51 (95% CI 0.19 - 0.83), p=0.002 using the model with random effects.

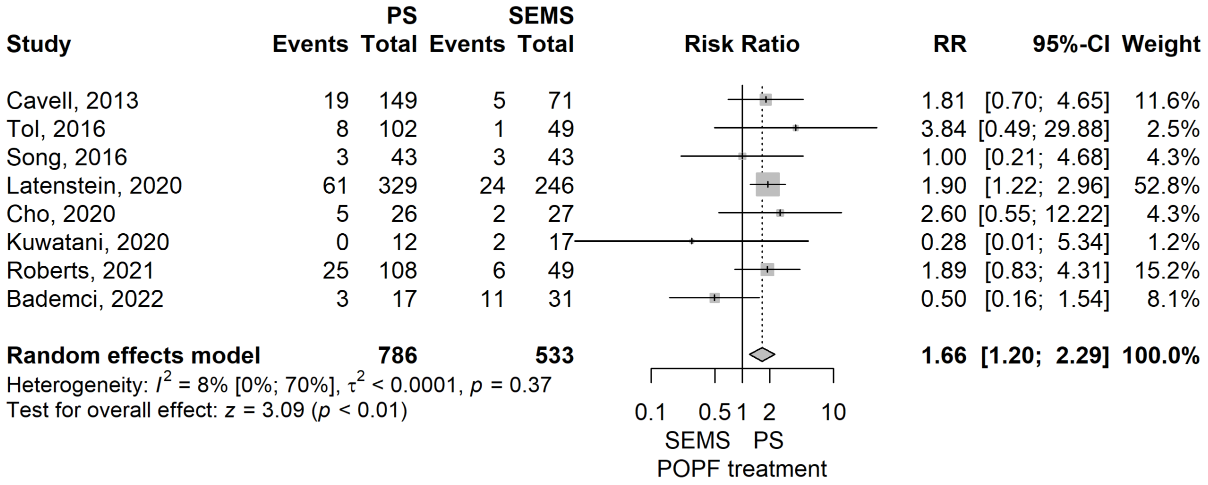

Fig. Forest plot for POPF treatment, comparing PS with SEMS

Meta-analysis for Grade 1 treatment, comparing PS with SEMS

	datele.Year	datele.Study.name	numberCasesPerTreatment	numberCasesTreatment	numberCasesPerControl	numberCasesControl
3	2016	Tol, 2016	0	102	0	49
4	2016	Song, 2016	2	43	2	43

2

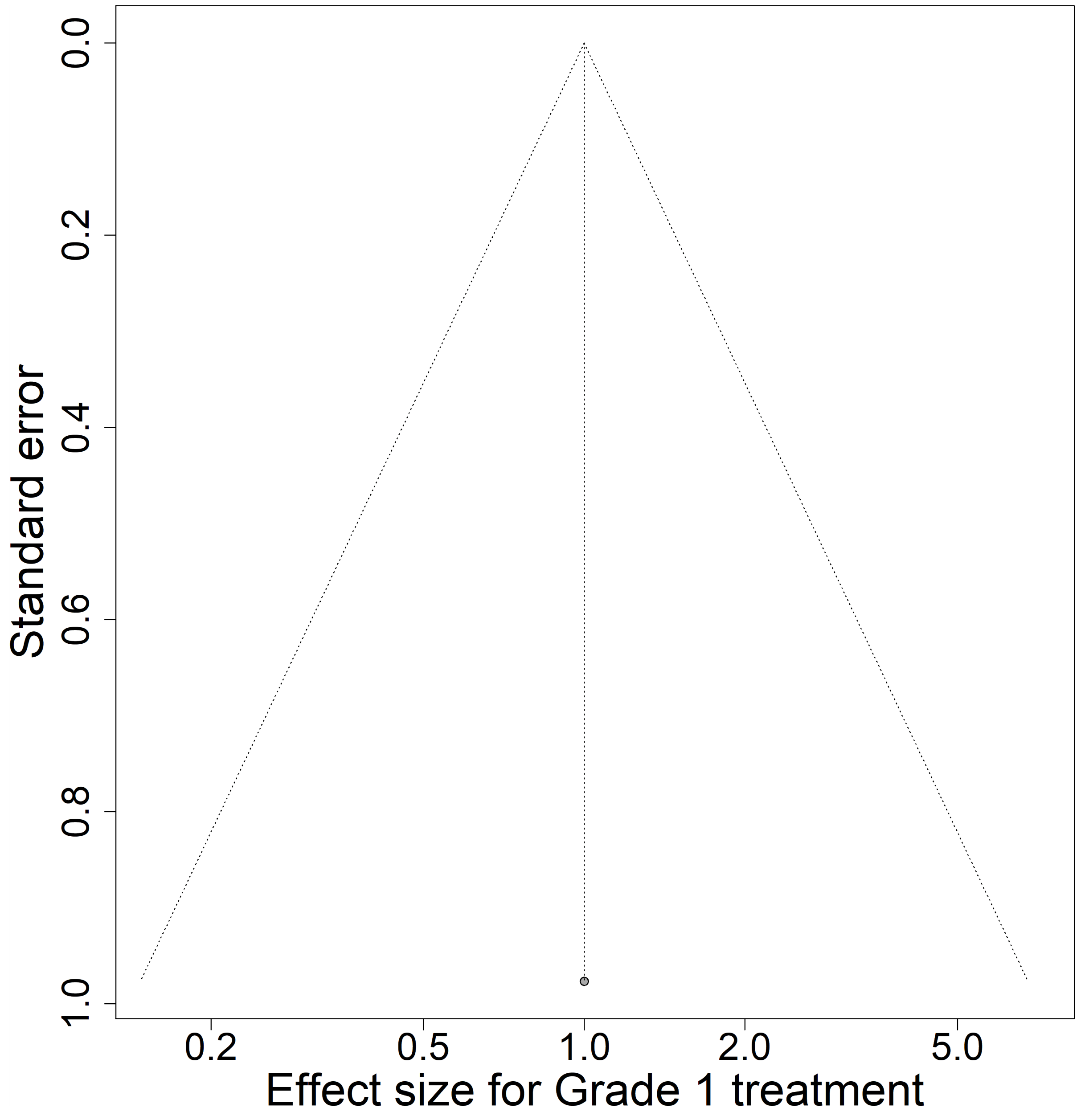

Fig. Funnel plot for Grade 1 treatment, comparing PS with SEMS

The funnel plot for Grade 1 treatment, comparing PS with SEMS is shown in figure _.

The publication bias test cannot be computed since there are not at least three studies.

Influence analysis plus chart - Algorithm did not converge

The heterogeneity was assessed, and we found an I2 of NA% (95% CI NA% - NA%) and the Q test for heterogeneity gave p=1.

The RR value (the RR of Grade 1 treatment in the PS group compared to the SEMS group) obtained with the meta-analysis was of 0 (95% CI -1.91 - 1.91), p=1 using the model with random effects.

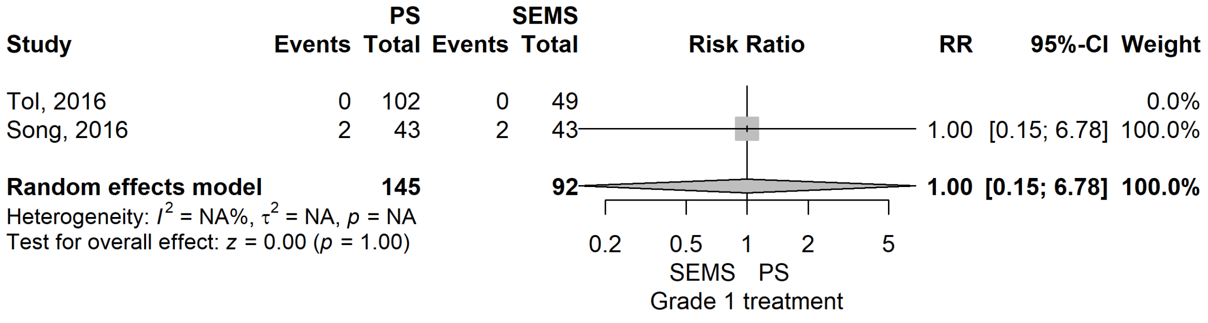

Fig. Forest plot for Grade 1 treatment, comparing PS with SEMS

Meta-analysis for Grade 2-3 treatment, comparing PS with SEMS

	datele.Year	datele.Study.name	numberCasesPerTreatment	numberCasesTreatment	numberCasesPerControl	numberCasesControl
3	2016	Tol, 2016	8	102	1	49
4	2016	Song, 2016	1	43	1	43
5	2020	Latenstein, 2020	61	329	24	246

2

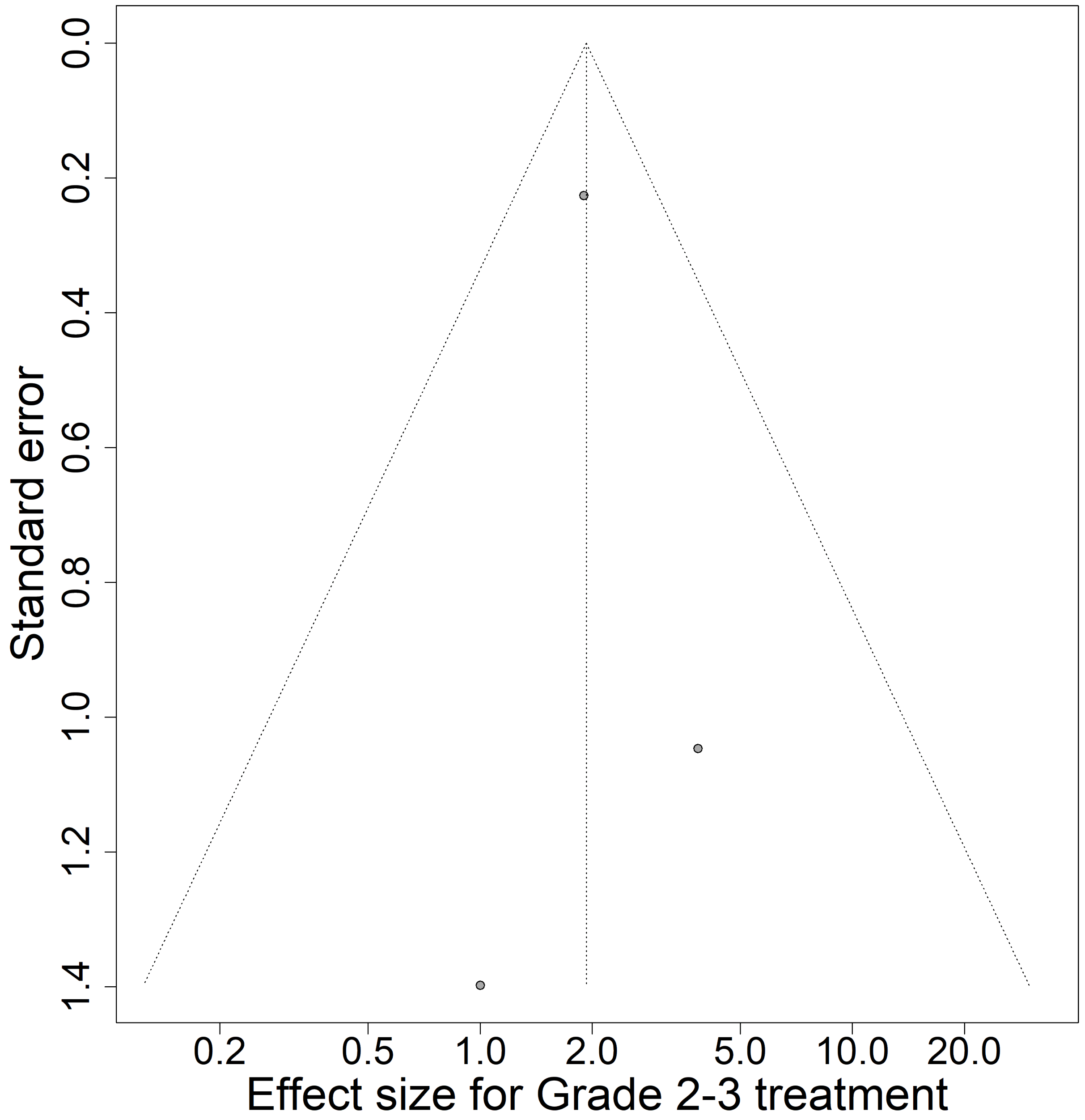

Fig. Funnel plot for Grade 2-3 treatment, comparing PS with SEMS

The funnel plot for Grade 2-3 treatment, comparing PS with SEMS is shown in figure _.

The publication bias test gave a p=0.913.

Influence studies: Omitting Tol, 2016; Omitting Song, 2016; Omitting Latenstein, 2020 - no; no; yes

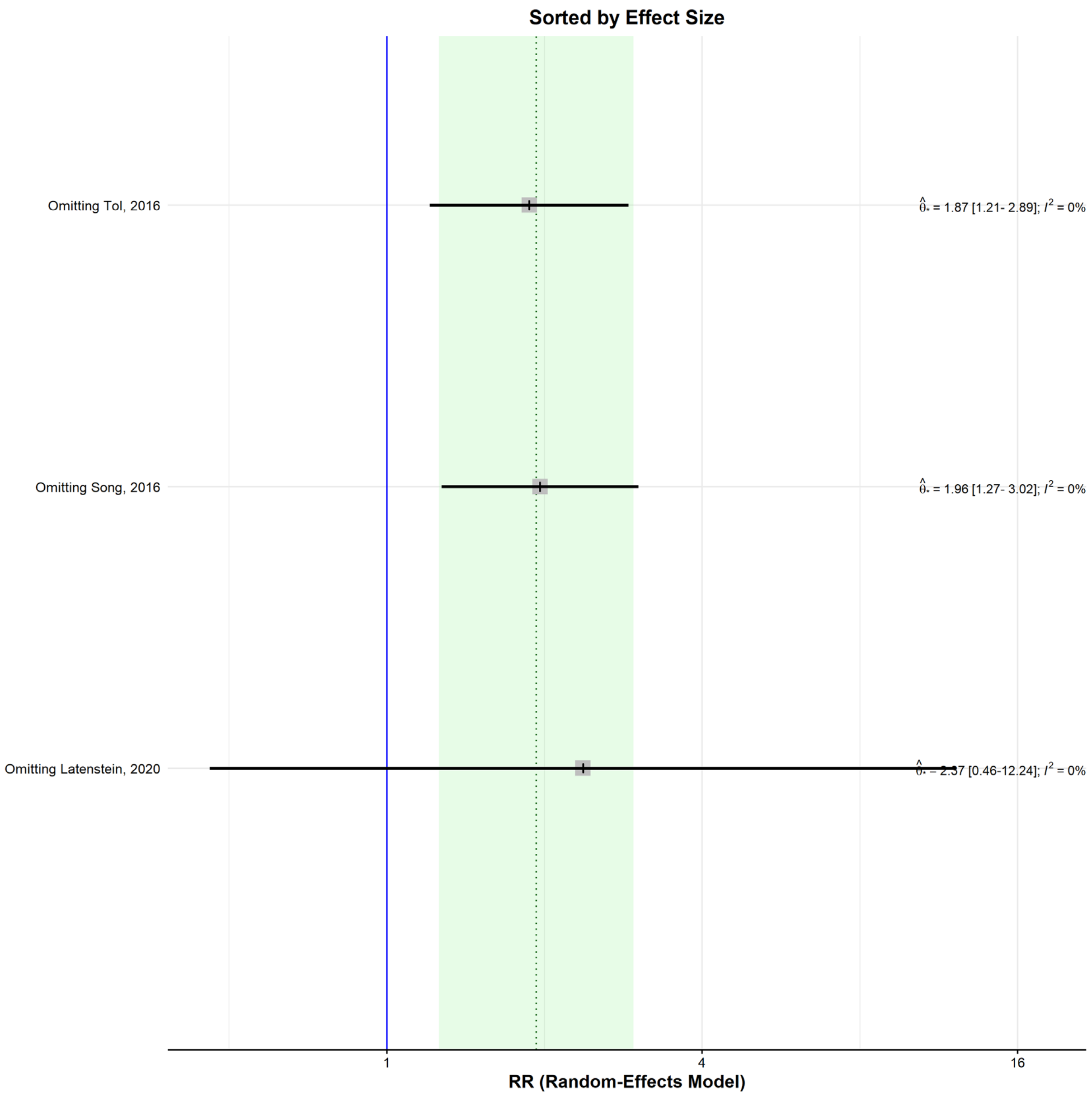

Fig. Leave-one-out sensitivity analysis plot for selected studies for Grade 2-3 treatment

The heterogeneity was assessed, and we found an I2 of 0% (95% CI 0% - 89.6%) and the Q test for heterogeneity gave p=0.719.

The RR value (the RR of Grade 2-3 treatment in the PS group compared to the SEMS group) obtained with the meta-analysis was of 0.66 (95% CI 0.23 - 1.08), p=0.003 using the model with random effects.

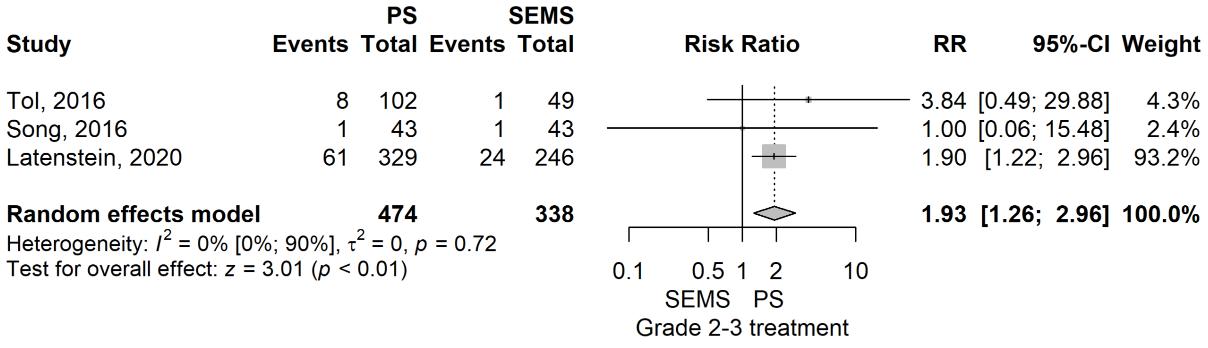

Fig. Forest plot for Grade 2-3 treatment, comparing PS with SEMS

Meta-analysis for DGE treatment, comparing PS with SEMS

	datele.Year	datele.Study.name	numberCasesPerTreatment	numberCasesTreatment	numberCasesPerControl	numberCasesControl
3	2016	Tol, 2016	18	102	6	49
4	2016	Song, 2016	3	43	2	43
5	2020	Latenstein, 2020	72	329	39	246
6	2020	Cho, 2020	1	26	3	27
9	2022	Bademci, 2022	3	17	9	31

2

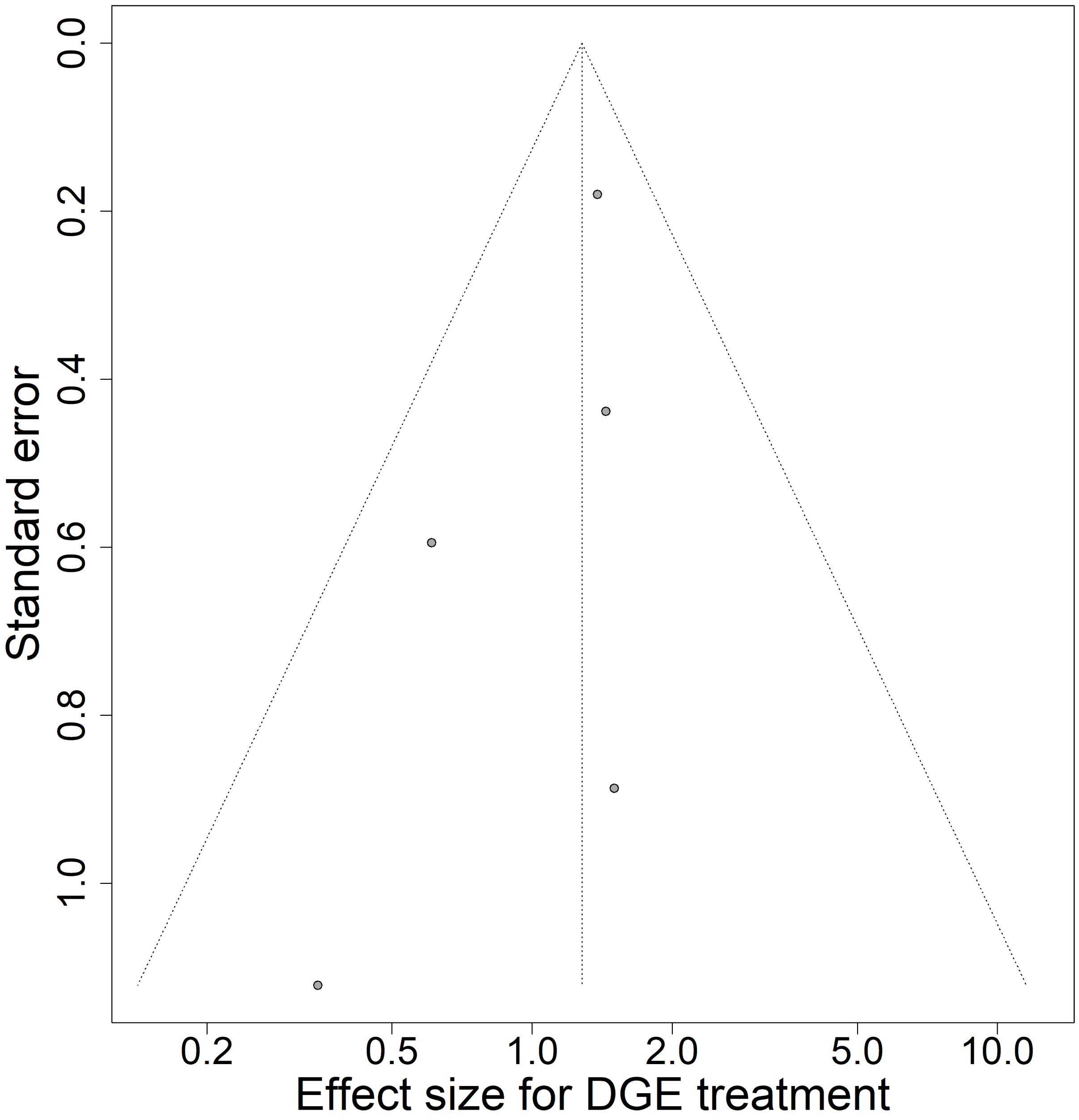

Fig. Funnel plot for DGE treatment, comparing PS with SEMS

The funnel plot for DGE treatment, comparing PS with SEMS is shown in figure _.

The publication bias test gave a p=0.231.

Influence studies: Omitting Tol, 2016; Omitting Song, 2016; Omitting Latenstein, 2020; Omitting Cho, 2020; Omitting Bademci, 2022 - yes; no; yes; no; no

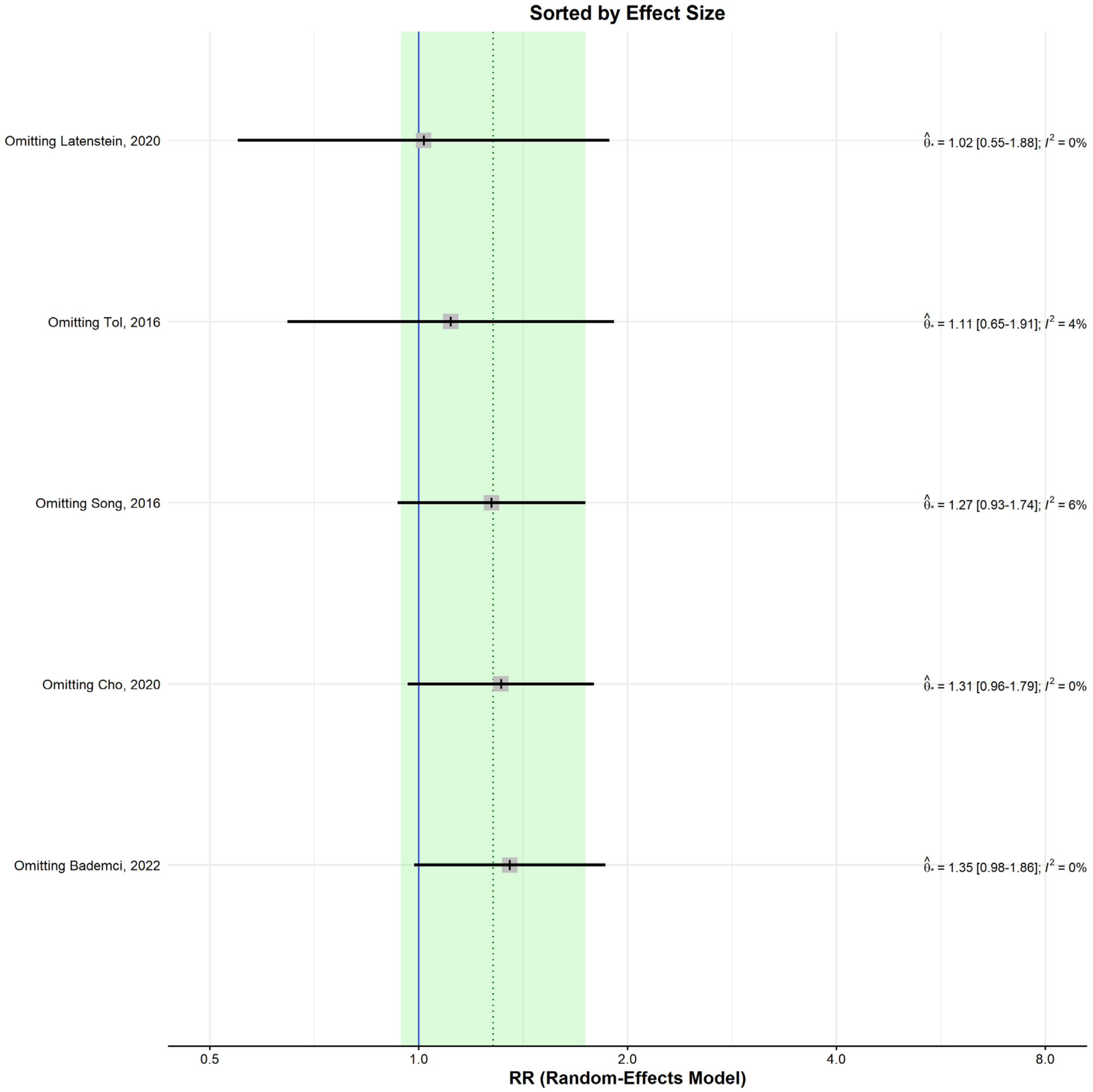

Fig. Leave-one-out sensitivity analysis plot for selected studies for DGE treatment

The heterogeneity was assessed, and we found an I2 of 0% (95% CI 0% - 79.2%) and the Q test for heterogeneity gave p=0.523.

The RR value (the RR of DGE treatment in the PS group compared to the SEMS group) obtained with the meta-analysis was of 0.25 (95% CI -0.06 - 0.55), p=0.114 using the model with random effects.

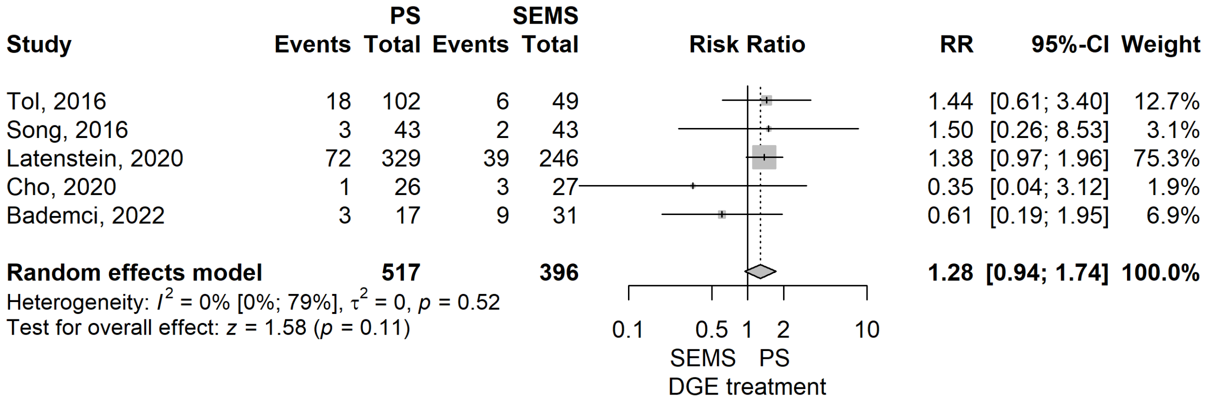

Fig. Forest plot for DGE treatment, comparing PS with SEMS

Meta-analysis for Soft pancreas treatment, comparing PS with SEMS

	datele.Year	datele.Study.name	numberCasesPerTreatment	numberCasesTreatment	numberCasesPerControl	numberCasesControl
5	2020	Latenstein, 2020	209	329	121	246

2

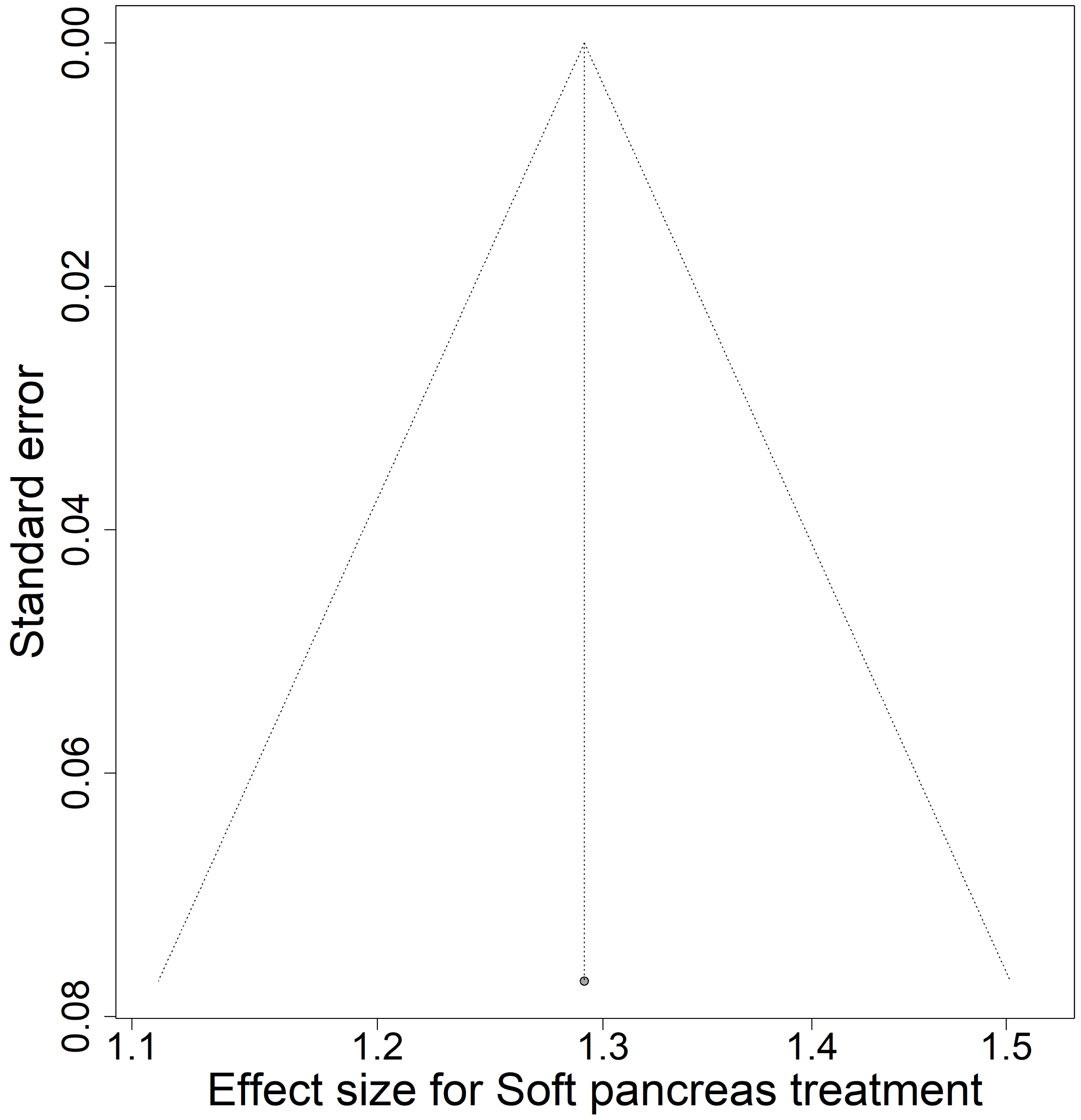

Fig. Funnel plot for Soft pancreas treatment, comparing PS with SEMS

The funnel plot for Soft pancreas treatment, comparing PS with SEMS is shown in figure _.

The publication bias test cannot be computed since there are not at least three studies.

The heterogeneity cannot be assessed since there are not at least two studies.

The RR value (the RR of Soft pancreas treatment in the PS group compared to the SEMS group) obtained with the meta-analysis was of 0.26 (95% CI 0.1 - 0.41), p=< 0.001 using the model with random effects.

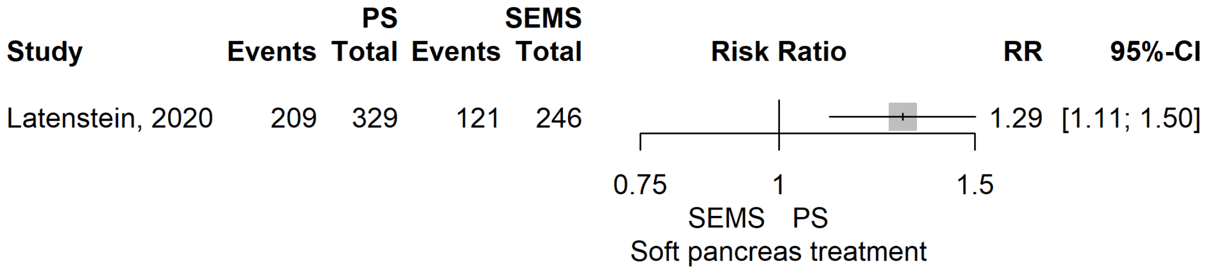

Fig. Forest plot for Soft pancreas treatment, comparing PS with SEMS

Sinteza metaanalizelor

Characteristic, effect size type	N studies	e/nt intervention	e/nt control	Effect size (95% CI)	p-value	I2 (95 % CI)	p-value	Egger test	Studies	Leave one out
Pancreatitis treatment, RR	5	43/608	43/414	-0.33 (-0.8 - 0.14)	0.165	24.9 (0 - 69.7)	0.256	0.524	Tol, 2016; Song, 2016; Latenstein , 2020; Cho, 2020; Roberts, 2021	- Tol, 2016: 0.86 (0.54-1.37), p=0.538, I2=0%; - Song, 201: 0.76 (0.49-1.19), p=0.228, I2=8%; - Latenstei: 0.61 (0.28-1.35), p=0.224, I2=35%; - Cho, 2020: 0.64 (0.33-1.21), p=0.169, I2=39%; - Roberts, : 0.65 (0.37-1.15), p=0.14, I2=36%

Cholangitis treatment, RR	5	76/608	21/414	1 (-0.02 - 2.02)	0.054	36.2 (0 - 76.1)	0.18	0.057	Tol, 2016; Song, 2016; Latenstein, 2020; Cho, 2020; Roberts, 2021	- Tol, 2016: 1.73 (0.83-3.6), p=0.143, I2=0%; - Song, 201: 2.65 (0.86-8.18), p=0.09, I2=48%; - Latenstei: 4.71 (1.56-14.2), p=0.006, I2=0%; - Cho, 2020: 2.88 (0.9-9.26), p=0.075, I2=51%; - Roberts, : 3.16 (0.74-13.61), p=0.122, I2=44%
Perforation treatment, RR	3	15/539	4/344	0.91 (-0.19 - 2.02)	0.104	0 (NA - NA)	0.374	NC	Tol, 2016; Latenstein, 2020; Roberts, 2021	- Tol, 2016: 3.24 (0.93-11.25), p=0.064, I2=NA%; - Latenstei: 0.96 (0.09-10.34), p=0.974, I2=NA%
Hemorrhage treatment, RR	4	20/565	8/371	0.45 (-0.31 - 1.21)	0.246	0 (0 - 84.7)	0.943	0.022	Tol, 2016; Latenstein, 2020; Cho, 2020; Roberts, 2021	- Tol, 2016: 1.52 (0.69-3.34), p=0.294, I2=0%; -

									Latenstei: 2.56 (0.44-15.02), p=0.296, I2=0%; - Cho, 2020: 1.5 (0.69-3.29), p=0.308, I2=0%; - Roberts, : 1.53 (0.7-3.35), p=0.29, I2=0%
Occlusion treatment, RR	5	59/608	17/414	0.79 (0.25 - 1.33)	0.004	0 (0 - 79.2)	0.795	0.482	Tol, 2016; Song, 2016; Latenstein , 2020; Cho, 2020; Roberts, 2021	- Tol, 2016: 2.03 (1.13-3.63), p=0.018, I2=0%; - Song, 201: 2.16 (1.22-3.85), p=0.009, I2=0%; - Latenstei: 2.99 (1.23-7.29), p=0.016, I2=0%; - Cho, 2020: 2.27 (1.31-3.94), p=0.004, I2=0%; - Roberts, : 2.06 (1.18-3.62), p=0.011, I2=0%

Catheter exchange treatment, RR	4	70/500	30/365	0.61 (-0.48 - 1.7)	0.273	69.5 (0 - 91.1)	0.038	0.407	Tol, 2016; Song, 2016; Latenstein, 2020; Cho, 2020	- Tol, 2016: 1.1 (0.7-1.75), p=0.674, I2=0%; - Song, 201: 2.54 (0.39-16.54), p=0.329, I2=85%; - Latenstei: 2.76 (0.45-16.92), p=0.272, I2=78%
Mortality treatment, RR	6	15/666	9/467	0.24 (-0.8 - 1.28)	0.654	3.5 (0 - 79.9)	0.386	0.685	Cavell, 2013; Tol, 2016; Song, 2016; Latenstein, 2020; Cho, 2020; Bademci, 2022	- Cavell, 2013: 1.27 (0.45-3.6), p=0.654, I2=4%; - Tol, 2016: 0.86 (0.29-2.55), p=0.788, I2=0%; - Song, 2016: 1.17 (0.36-3.79), p=0.798, I2=21%; - Latenstein, : 1.91 (0.46-7.94), p=0.373, I2=5%; - Cho, 2020: 1.16 (0.36-3.76), p=0.805, I2=21%

Reoperation treatment, RR	3	43/594	21/323	0.03 (-0.78 - 0.83)	0.951	48.9 (0 - 85.1)	0.141	0.727	Haapamaki, 2015; Tol, 2016; Latenstein, 2020	- Haapamaki, 2015: 1.36 (0.78-2.36), p=0.272, I2=0%; - Tol, 2016: 0.75 (0.22-2.58), p=0.65, I2=66%; - Latenstein, 202: 0.83 (0.15-4.48), p=0.828, I2=72%
Overall complication rate (Clavien - Dindo) treatment, RR	7	418/920	267/513	-0.05 (-0.15 - 0.05)	0.359	0 (0 - 70.8)	0.448	0.038	Cavell, 2013; Haapamaki, 2015; Tol, 2016; Song, 2016; Latenstein, 2020; Cho, 2020; Roberts, 2021	- Cavell, 2013: 1 (0.87-1.15), p=0.968, I2=0%; - Haapamaki, 2: 0.95 (0.86-1.05), p=0.334, I2=0%; - Tol, 2016: 0.94 (0.85-1.04), p=0.238, I2=0%; - Song, 2016: 0.95 (0.86-1.05), p=0.293, I2=1%; - Latenstein, : 1.01

										(0.83-1.23), p=0.914, I2=10%;<br>- Cho, 2020: 0.95 (0.86-1.05), p=0.291, I2=0%;<br>- Roberts, 202: 0.96 (0.86-1.06), p=0.405, I2=13%
Grade 1-2 treatment, RR	3	122/358	129/294	-0.24 (-0.43 - -0.04)	0.017	0 (0 - 89.6)	0.749	0.63	Latenstein, 2020; Kuwatani, 2020; Bademci, 2022	- Latenstein, 2020: 0.78 (0.45-1.36), p=0.387, I2=0%;<br>- Kuwatani, 2020: 0.8 (0.66-0.97), p=0.024, I2=0%;<br>- Bademci, 2022: 0.78 (0.64-0.96), p=0.019, I2=0%
Grade >=3 treatment, RR	4	127/507	74/365	0.22 (-0.04 - 0.47)	0.096	0 (0 - 84.7)	0.757	0.478	Cavell, 2013; Latenstein, 2020; Kuwatani, 2020; Bademci, 2022	- Cavell, 2013: 1.25 (0.95-1.66), p=0.116, I2=0%;<br>- Latenstein, : 1.3 (0.77-2.18),

									p=0.323, I2=0%; - Kuwatani, 20: 1.21 (0.94-1.57), p=0.143, I2=0%; - Bademci, 202: 1.25 (0.96-1.62), p=0.095, I2=0%
Infectious complications treatment, RR	1	23/163	5/28	-0.24 (-1.12 - 0.64)	0.6	NC	NC	Haapamaki, 2015	NC
Sepsis treatment, RR	1	3/17	8/31	-0.38 (-1.57 - 0.81)	0.531	NC	NC	Bademci, 2022	NC
Intraabdominal abcess treatment, RR	3	18/236	11/125	-0.21 (-0.92 - 0.49)	0.559	0 (0 - 89.6)	0.636	0.819	Tol, 2016; Cho, 2020; Roberts, 2021	- Tol, 2016: 0.88 (0.41-1.87), p=0.737, I2=0%; - Cho, 2020: 0.74 (0.35-1.54), p=0.42, I2=0%; - Roberts, : 0.87 (0.2-3.85), p=0.854, I2=0%
Wound infections treatment, RR	7	100/774	94/516	-0.42 (-0.76 - -0.08)	0.017	0 (0 - 70.8)	0.45	0.612	Cavell, 2013; Tol, 2016; Song, 2016; Latenstein	- Cavell, 2013: 0.79 (0.59-1.06), p=0.119, I2=0%;

									, 2020; Cho, 2020; Roberts, 2021; Bademci, 2022	- Tol, 2016: 0.62 (0.42-0.92), p=0.018, I2=6%;<br>- Song, 2016: 0.66 (0.46-0.95), p=0.025, I2=11%;<br>- Latenstein, : 0.55 (0.38-0.78), p=< 0.001, I2=0%;<br>- Cho, 2020: 0.66 (0.46-0.94), p=0.02, I2=12%;<br>- Roberts, 202: 0.67 (0.45-1.01), p=0.054, I2=10%;<br>- Bademci, 202: 0.65 (0.45-0.94), p=0.021, I2=12%
PPH treatment, RR	4	27/500	14/365	0.33 (-0.3 - 0.95)	0.307	0 (0 - 84.7)	0.664	0.671	Tol, 2016; Song, 2016; Latenstein , 2020; Cho, 2020	- Tol, 2016: 1.57 (0.81-3.05), p=0.182, I2=0%;<br>- Song, 201: 1.41 (0.74-2.69), p=0.294,

										I2=0%; - Latenstei: 0.85 (0.21-3.48), p=0.819, I2=0%; - Cho, 2020: 1.34 (0.71-2.54), p=0.369, I2=0%
Chyle leak treatment, RR	1	25/329	18/246	0.04 (-0.55 - 0.62)	0.899	NC		NC	Latenstein, 2020	NC
POBF treatment, RR	4	16/606	14/393	-0.25 (-0.95 - 0.45)	0.487	0 (0 - 84.7)	0.593	0.407	Cavell, 2013; Tol, 2016; Latenstein, 2020; Cho, 2020	- Cavell, 2013: 0.76 (0.37-1.55), p=0.445, I2=0%; - Tol, 2016: 0.89 (0.34-2.33), p=0.817, I2=0%; - Latenstein, : 1.43 (0.26-7.91), p=0.681, I2=0%; - Cho, 2020: 0.7 (0.34-1.44), p=0.329, I2=0%
POPF treatment, RR	8	124/786	54/533	0.51 (0.19 - 0.83)	0.002	8.2 (0 - 70.2)	0.367	0.375	Cavell, 2013; Tol, 2016; Song, 2016;	- Cavell, 2013: 1.54 (0.99-2.4), p=0.057, I2=21%;

								Latenstein, 2020; Cho, 2020; Kuwatani, 2020; Roberts, 2021; Bademci, 2022	- Tol, 2016: 1.61 (1.14-2.26), p=0.007, I2=14%; - Song, 2016: 1.7 (1.22-2.36), p=0.002, I2=17%; - Latenstein, : 1.41 (0.84-2.36), p=0.192, I2=13%; - Cho, 2020: 1.59 (1.1-2.28), p=0.013, I2=18%; - Kuwatani, 20: 1.7 (1.23-2.35), p=0.001, I2=3%; - Roberts, 202: 1.5 (0.94-2.4), p=0.09, I2=20%; - Bademci, 202: 1.85 (1.32-2.58), p=<0.001, I2=0%
Grade 1 treatment, RR	2	2/145	2/92	0 (-1.91 - 1.91)	1	NA (NA - NA)	1	NC	Tol, 2016; Song, 2016	- Tol, 2016: 1 (0.15-6.78), p=1, I2=NA%

Grade 2-3 treatment, RR	3	70/474	26/338	0.66 (0.23 - 1.08)	0.003	0 (0 - 89.6)	0.719	0.913	Tol, 2016; Song, 2016; Latenstein, 2020	- Tol, 2016: 1.87 (1.21-2.89), p=0.005, I2=0%; - Song, 201: 1.96 (1.27-3.02), p=0.002, I2=0%; - Latenstei: 2.37 (0.46-12.24), p=0.303, I2=0%
DGE treatment, RR	5	97/517	59/396	0.25 (-0.06 - 0.55)	0.114	0 (0 - 79.2)	0.523	0.231	Tol, 2016; Song, 2016; Latenstein, 2020; Cho, 2020; Bademci, 2022	- Tol, 2016: 1.11 (0.65-1.91), p=0.7, I2=4%; - Song, 201: 1.27 (0.93-1.74), p=0.128, I2=6%; - Latenstei: 1.02 (0.55-1.88), p=0.958, I2=0%; - Cho, 2020: 1.31 (0.96-1.79), p=0.084, I2=0%; - Bademci, : 1.35 (0.98-1.86),

									p=0.062, I2=0%
Soft pancreas treatment, RR	1	209/329	121/246	0.26 (0.1 - 0.41)	< 0.001	NC	NC	Latenstein, 2020	NC
