# Supplementary material for: A Systematic Review and Meta-Analysis of Preoperative Biliary Drainage Methods in Periampullary Tumors
Source: J Clin Med. 2025 Oct 8;14(19):7097. doi: 10.3390/jcm14197097 (PMC12524691; doi:10.3390/jcm14197097)
Supplement: Supplementary file 1 [file jcm-14-07097-s001.zip › Supplementary material 6-Quantitative variables - Mean Differences.pdf]

Contents

Selectie: ERBD - ENBD/PTBD 1

Meta-analysis for MBD diameter (mm), comparing ERBD with ENBD/PTBD 1

Meta-analysis for Duration of drainage (days), comparing ERBD with ENBD/PTBD 4

Meta-analysis for Operative time (min), comparing ERBD with ENBD/PTBD 7

Meta-analysis for Blood loss (mL), comparing ERBD with ENBD/PTBD 10

Meta-analysis for Hospital stay (days), comparing ERBD with ENBD/PTBD 13

Sinteza metaanalizelor 16

Selectie: PS - SEMS 22

Meta-analysis for MBD diameter (mm), comparing PS with SEMS 22

Meta-analysis for Duration of drainage (days), comparing PS with SEMS 23

Meta-analysis for Operative time (min), comparing PS with SEMS 26

Meta-analysis for Blood loss (mL), comparing PS with SEMS 29

Meta-analysis for Hospital stay (days), comparing PS with SEMS 31

Sinteza metaanalizelor 34

Selectie: ERBD - ENBD/PTBD

Meta-analysis for MBD diameter (mm), comparing ERBD with ENBD/PTBD

	datele.Year	datele.Study.name	MeanTreatment	SDTreatment	numberCasesTreatment	MeanControl	SDControl	numberCasesControl
3	2015	Fujii, 2015	4.90	3.10	72	4.30	2.20	50
6	2017	Zhang, 2017	17.80	5.20	51	17.30	4.50	102
14	2022	Satoh, 2022	4.38	1.55	117	4.94	1.77	39

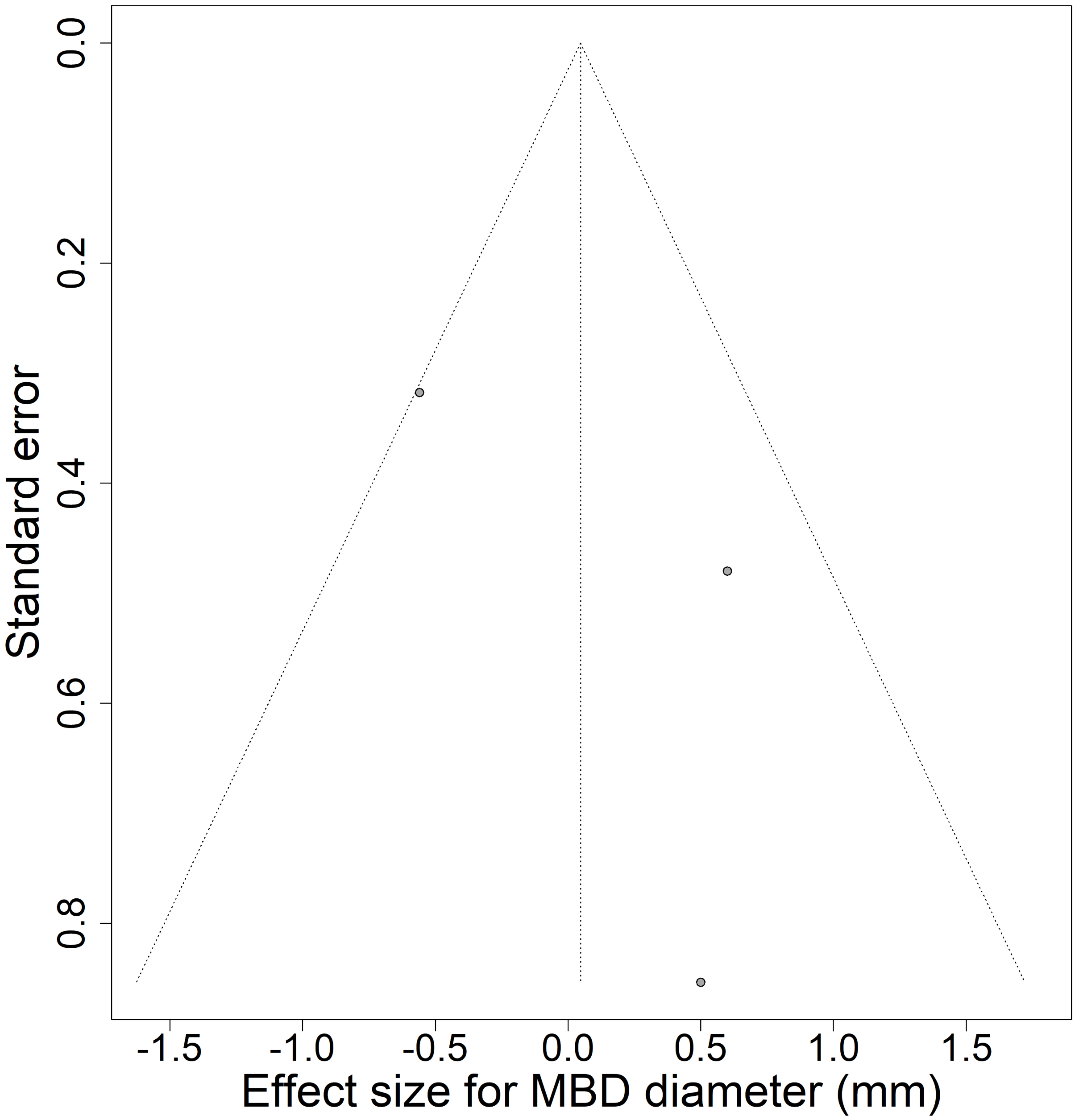

Fig. Funnel plot for MBD diameter (mm), comparing ERBD with ENBD/PTBD

The funnel plot for MBD diameter (mm), comparing ERBD with ENBD/PTBD is shown in figure _.

The publication bias test gave a p=0.458.

Influence studies: Omitting Fujii, 2015; Omitting Zhang, 2017; Omitting Satoh, 2022 - yes; no; yes

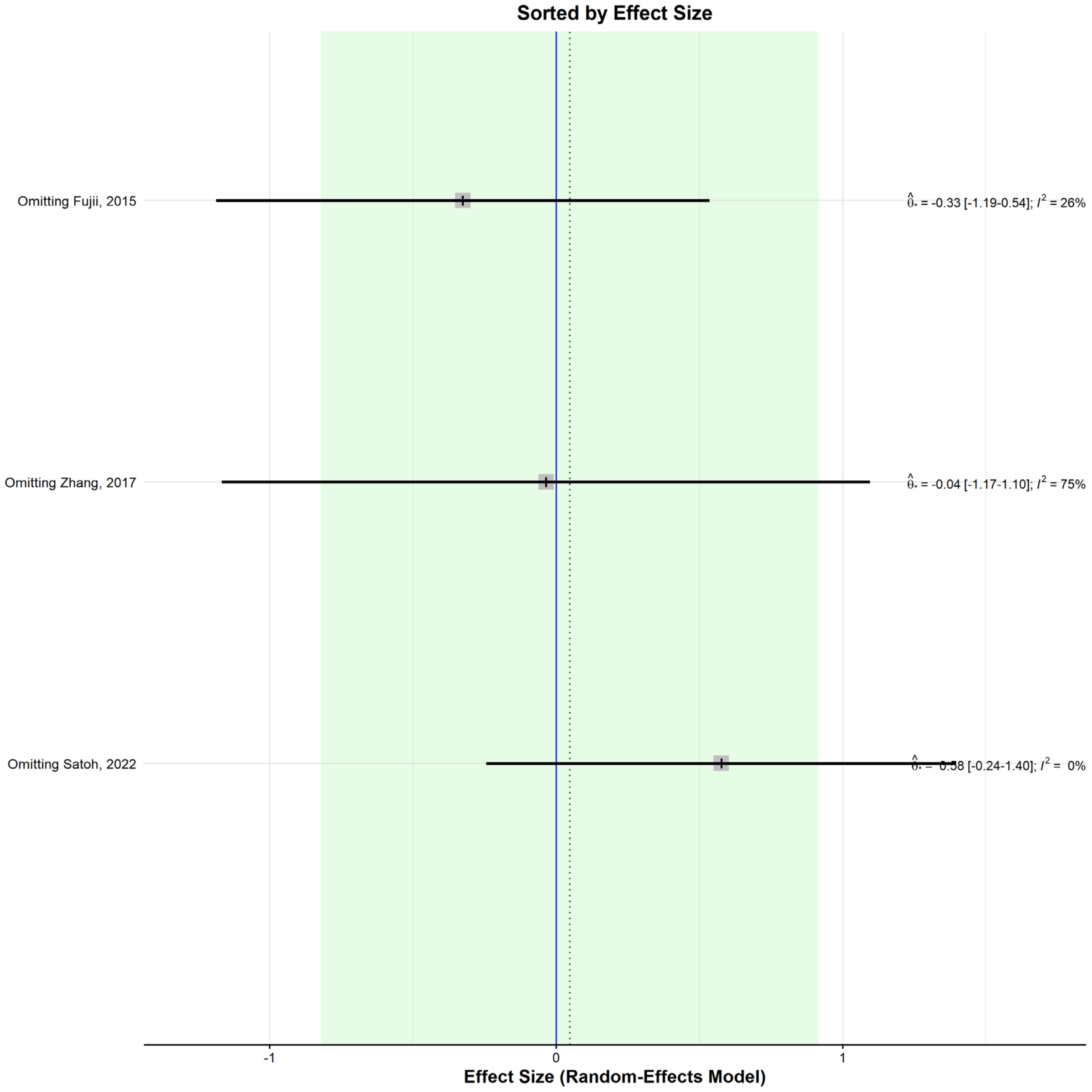

Fig. Leave-one-out sensitivity analysis plot for selected studies for MBD diameter (mm)

The heterogeneity was assessed, and we found an I2 of 57.3% (95% CI 0% - 87.8%) and the Q test for heterogeneity gave p=0.096.

The MD value (the MD of MBD diameter (mm) in the ERBD group compared to the ENBD/PTBD group) obtained with the meta-analysis was of 0.05 (95% CI -0.82 - 0.92), p=0.915 using the model with random effects.

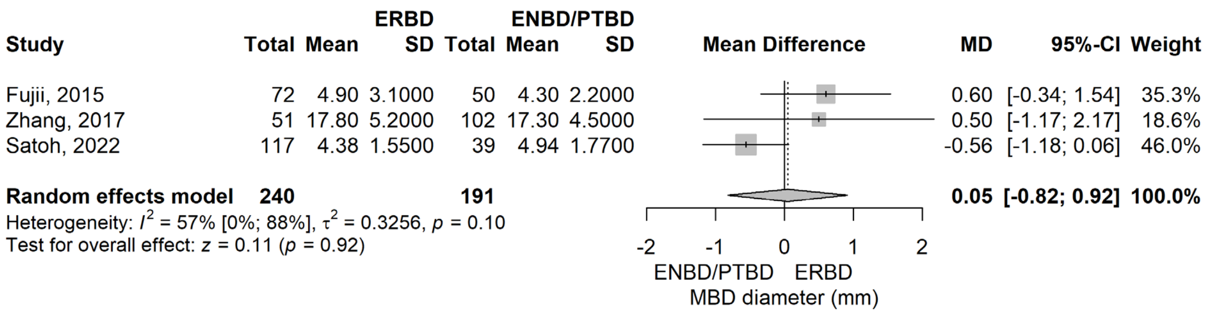

Fig. Forest plot for MBD diameter (mm), comparing ERBD with ENBD/PTBD

Meta-analysis for Duration of drainage (days), comparing ERBD with ENBD/PTBD

	datele.Year	datele.Study.name	MeanTreatment	SDTreatment	numberCasesTreatment	MeanControl	SDControl	numberCasesControl
1	2011	Park, 2011	20.81	8.61	34	13.05	4.79	43
2	2014	Kitahata, 2014	34.11	14.57	67	29.42	13.80	60
3	2015	Fujii, 2015	39.40	23.90	72	47.30	40.80	50
5	2015	Huang, 2015	45.20	59.90	37	24.11	21.18	63
6	2017	Zhang, 2017	13.55	7.60	51	11.05	4.87	102
12	2021	El-Haddad, 2021	15.00	6.10	34	16.60	5.80	30
13	2021	Suenaga, 2021	58.33	49.98	40	44.00	22.34	38
14	2022	Satoh, 2022	128.81	78.84	117	61.89	37.55	39

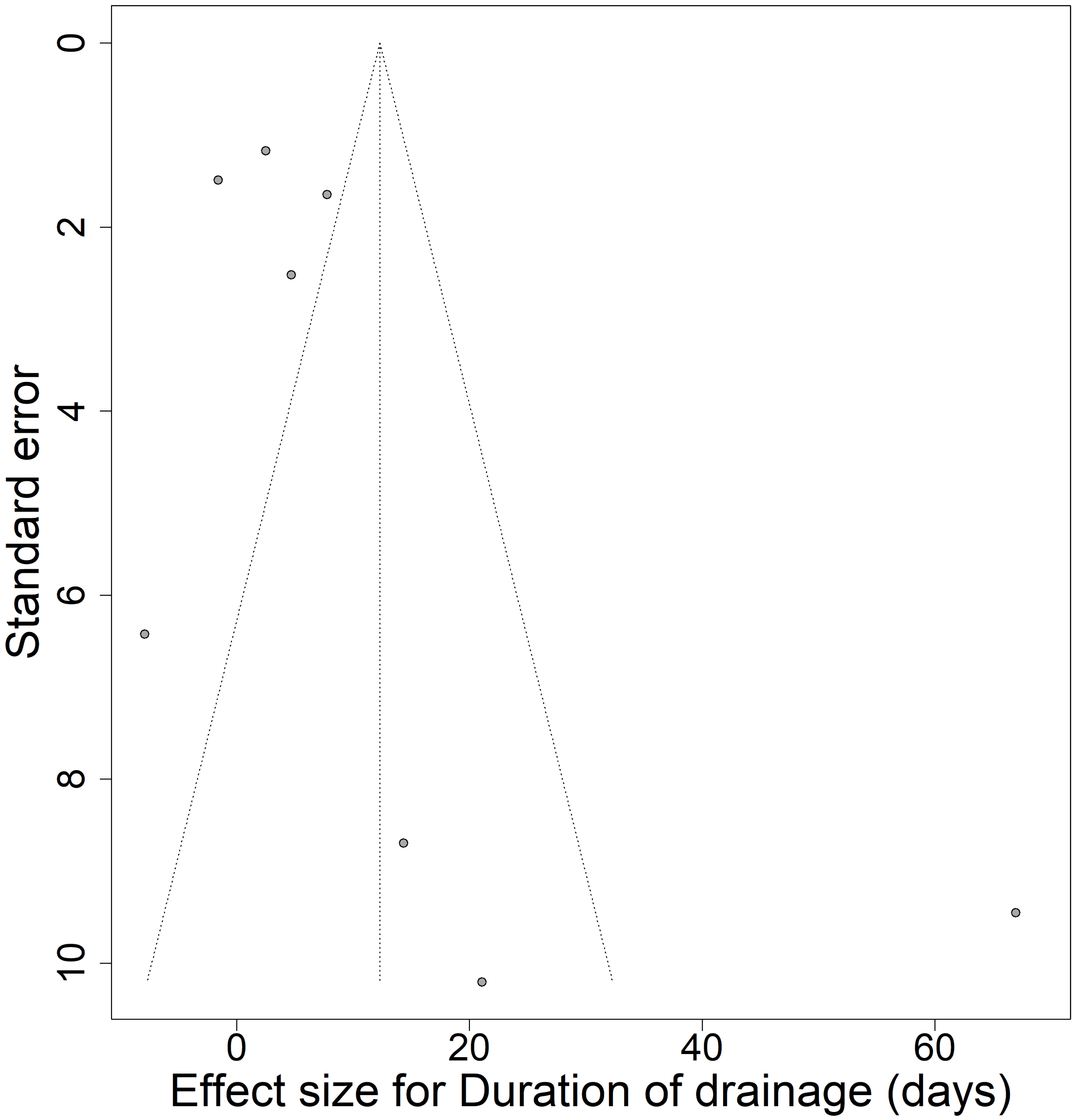

Fig. Funnel plot for Duration of drainage (days), comparing ERBD with ENBD/PTBD

The funnel plot for Duration of drainage (days), comparing ERBD with ENBD/PTBD is shown in figure _.

The publication bias test gave a p=0.18.

Influence studies: Omitting Park, 2011; Omitting Kitahata, 2014; Omitting Fujii, 2015; Omitting Huang, 2015; Omitting Zhang, 2017; Omitting El-Haddad, 2021; Omitting Suenaga, 2021; Omitting Satoh, 2022 - no; no; no; no; no; no; no; yes

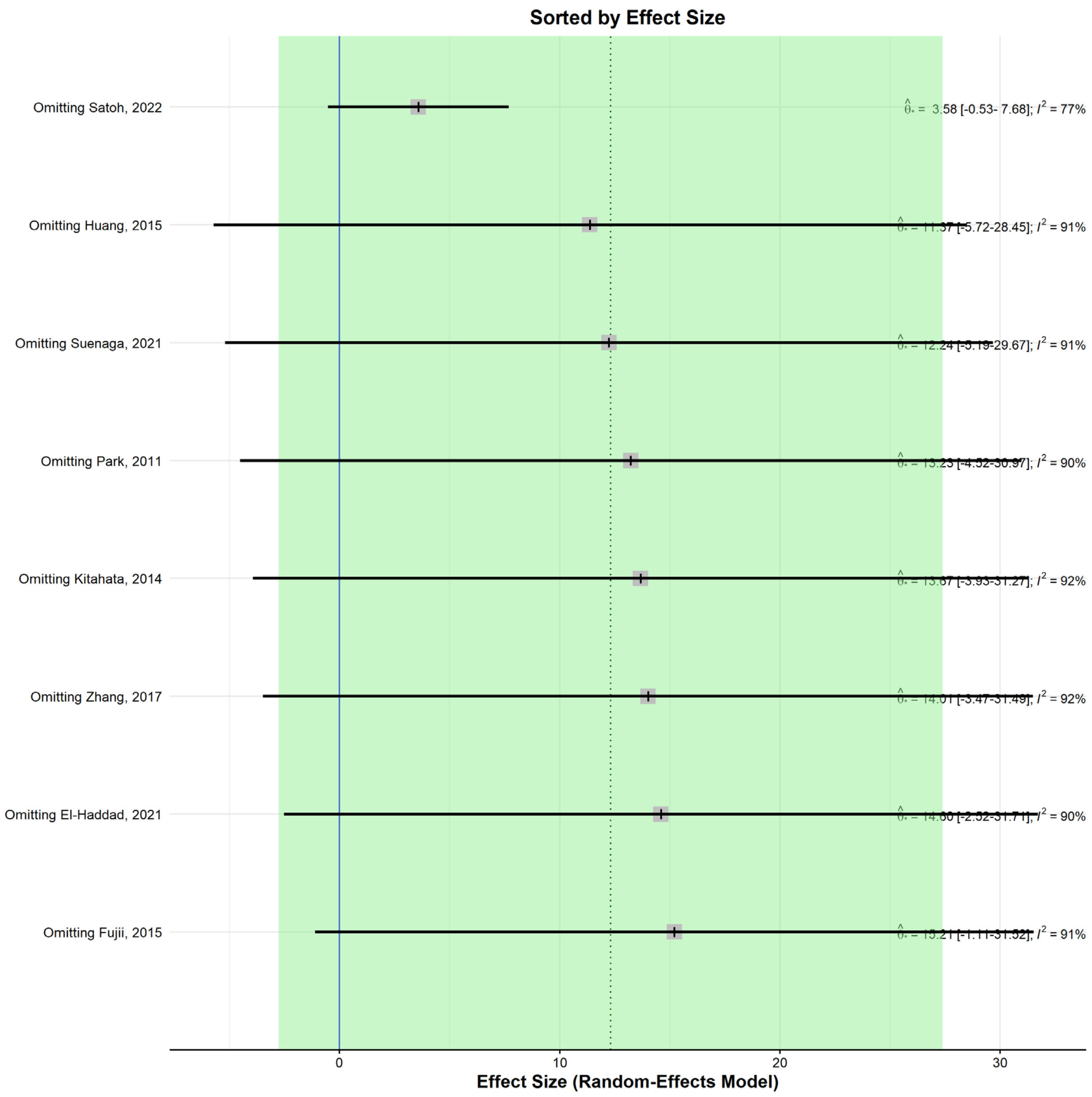

Fig. Leave-one-out sensitivity analysis plot for selected studies for Duration of drainage (days)

The heterogeneity was assessed, and we found an I2 of 90.3% (95% CI 83.2% - 94.4%) and the Q test for heterogeneity gave p=< 0.001.

The MD value (the MD of Duration of drainage (days) in the ERBD group compared to the ENBD/PTBD group) obtained with the meta-analysis was of 12.31 (95% CI -2.76 - 27.39), p=0.109 using the model with random effects.

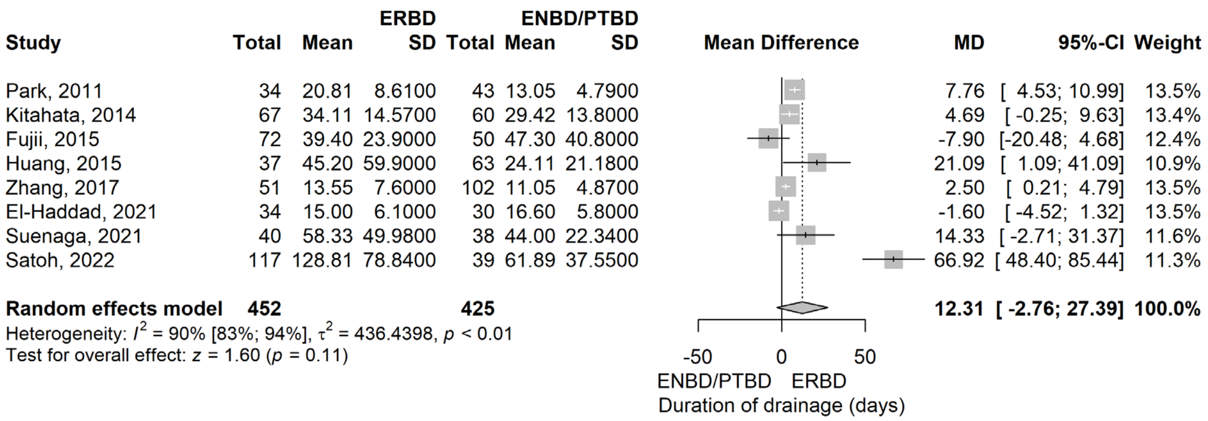

Fig. Forest plot for Duration of drainage (days), comparing ERBD with ENBD/PTBD

Meta-analysis for Operative time (min), comparing ERBD with ENBD/PTBD

	datele.Year	datele.Study.name	MeanTreatment	SDTreatment	numberCasesTreatment	MeanControl	SDControl	numberCasesControl
1	2011	Park, 2011	413.40	107.50	34	492.40	83.80	43
2	2014	Kitahata, 2014	385.98	71.91	67	426.10	92.83	60
3	2015	Fujii, 2015	456.00	106.00	72	459.00	113.00	50
4	2015	Uemura, 2015	551.18	125.21	407	568.80	148.23	166
5	2015	Huang, 2015	370.90	91.30	37	369.85	100.03	63
6	2017	Zhang, 2017	375.49	105.66	51	358.97	84.21	102
7	2018	Lee, 2018	317.20	89.60	335	318.80	102.40	234
10	2021	Han, 2021	418.89	75.92	42	454.04	92.55	46
11	2021	Byun, 2021	304.66	79.66	106	314.00	74.40	61
13	2021	Suenaga, 2021	457.66	127.64	40	477.00	133.28	38
14	2022	Satoh, 2022	531.72	87.65	117	514.17	117.13	39
15	2022	Subasi, 2022	362.90	142.60	42	358.80	109.80	33

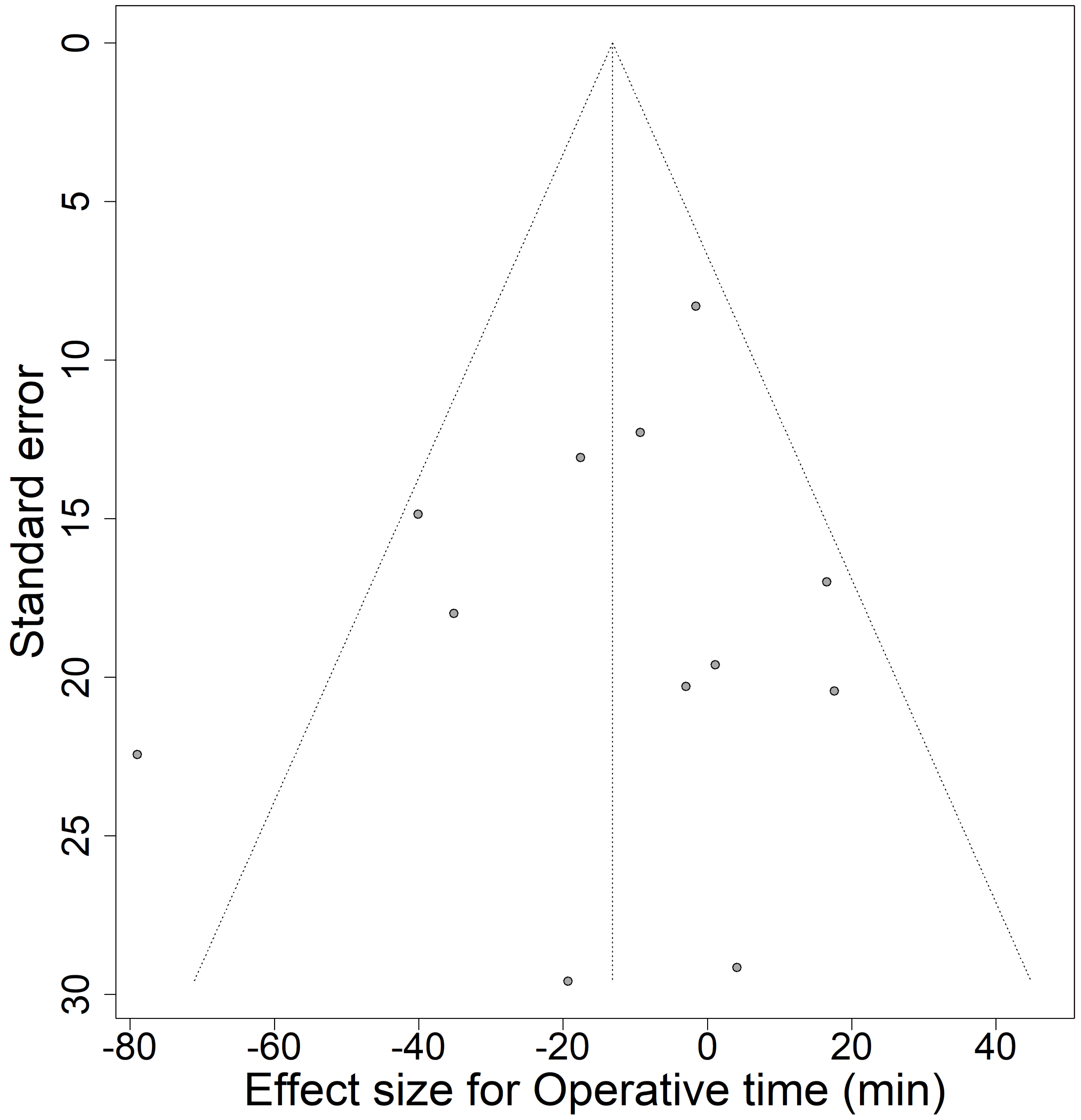

Fig. Funnel plot for Operative time (min), comparing ERBD with ENBD/PTBD

The funnel plot for Operative time (min), comparing ERBD with ENBD/PTBD is shown in figure _.

The publication bias test gave a p=0.531.

Influence studies: Omitting Park, 2011; Omitting Kitahata, 2014; Omitting Fujii, 2015; Omitting Uemura, 2015; Omitting Huang, 2015; Omitting Zhang, 2017; Omitting Lee, 2018; Omitting Han, 2021; Omitting Byun, 2021; Omitting Suenaga, 2021; Omitting Satoh, 2022; Omitting Subasi, 2022 - no; no; no; no; no; no; no; no; no; no; no; no

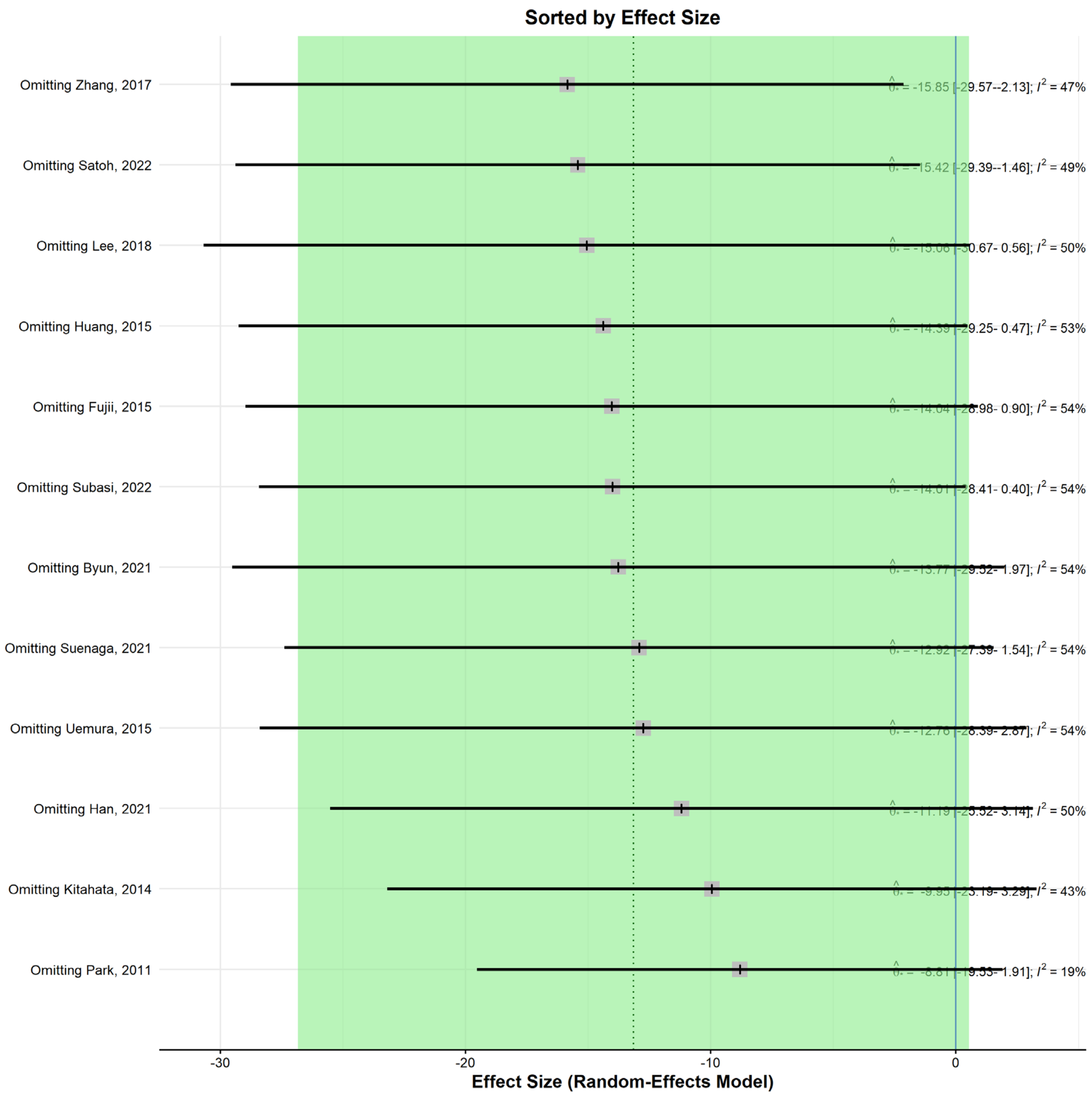

Fig. Leave-one-out sensitivity analysis plot for selected studies for Operative time (min)

The heterogeneity was assessed, and we found an I2 of 49.7% (95% CI 2.3% - 74.1%) and the Q test for heterogeneity gave p=0.026.

The MD value (the MD of Operative time (min) in the ERBD group compared to the ENBD/PTBD group) obtained with the meta-analysis was of -13.14 (95% CI -26.83 - 0.54), p=0.06 using the model with random effects.

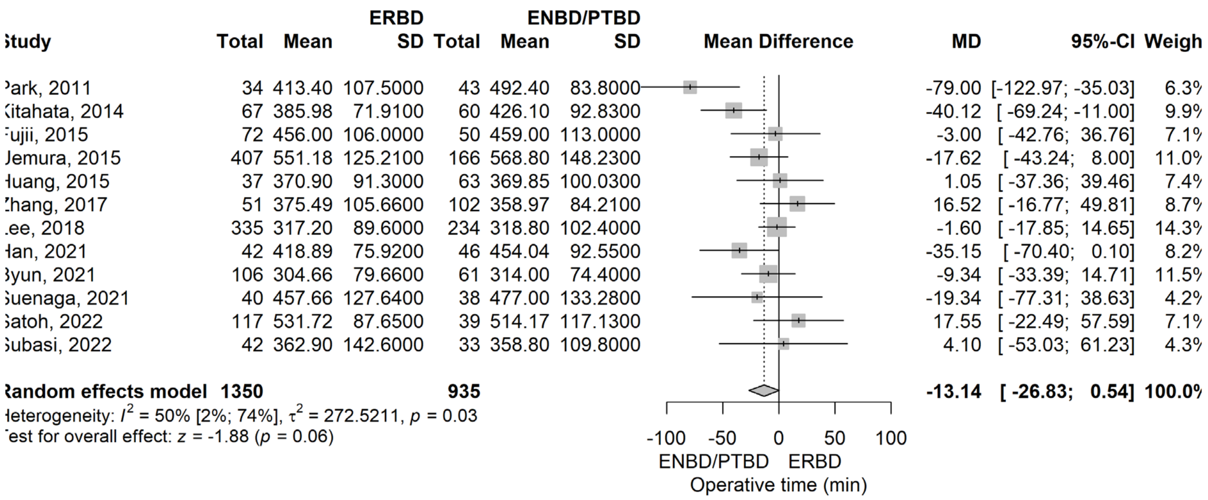

Fig. Forest plot for Operative time (min), comparing ERBD with ENBD/PTBD

Subgroup analyses

Algorithm did not converge for subgroup analisis of

Algorithm did not converge for subgroup analisis of

Meta-analysis for Blood loss (mL), comparing ERBD with ENBD/PTBD

	datele.Year	datele.Study.name	MeanTreatment	SDTreatment	numberCasesTreatment	MeanControl	SDControl	numberCasesControl
2	2014	Kitahata, 2014	2151.31	1429.34	67	1984.06	1255.41	60
3	2015	Fujii, 2015	1203.00	924.00	72	1162.00	1006.00	50
4	2015	Uemura, 2015	7107.14	4170.93	407	2196.70	1091.55	166
5	2015	Huang, 2015	494.60	293.60	37	426.50	236.13	63
6	2017	Zhang, 2017	488.04	306.31	51	475.49	274.47	102
7	2018	Lee, 2018	398.90	322.40	335	465.40	419.60	234
10	2021	Han, 2021	1032.74	485.07	42	1463.04	811.14	46
11	2021	Byun, 2021	300.00	225.47	106	316.66	265.74	61
12	2021	El-Haddad, 2021	1705.00	405.00	34	1158.00	190.00	30

13	2021	Suenaga, 2021	696.00	606.68	40	797.66	553.16	38
14	2022	Satoh, 2022	1925.88	1086.47	117	1145.90	633.69	39
2								

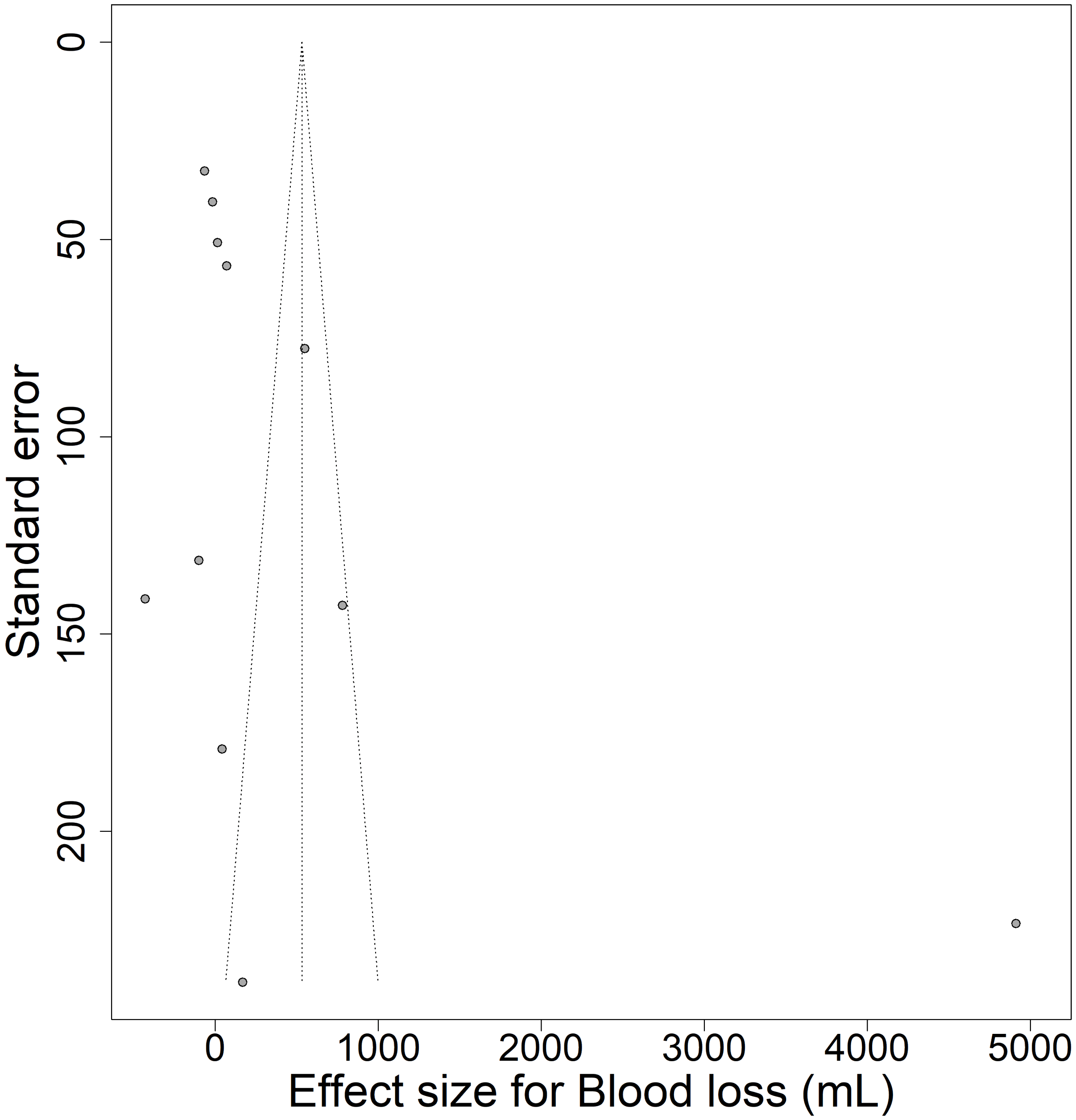

Fig. Funnel plot for Blood loss (mL), comparing ERBD with ENBD/PTBD

The funnel plot for Blood loss (mL), comparing ERBD with ENBD/PTBD is shown in figure _.

The publication bias test gave a p=0.115.

Influence studies: Omitting Kitahata, 2014; Omitting Fujii, 2015; Omitting Uemura, 2015; Omitting Huang, 2015; Omitting Zhang, 2017; Omitting Lee, 2018; Omitting Han, 2021; Omitting Byun, 2021; Omitting El-Haddad, 2021; Omitting Suenaga, 2021; Omitting Satoh, 2022 - no; no; yes; no; no; no; no; no; no; no; no

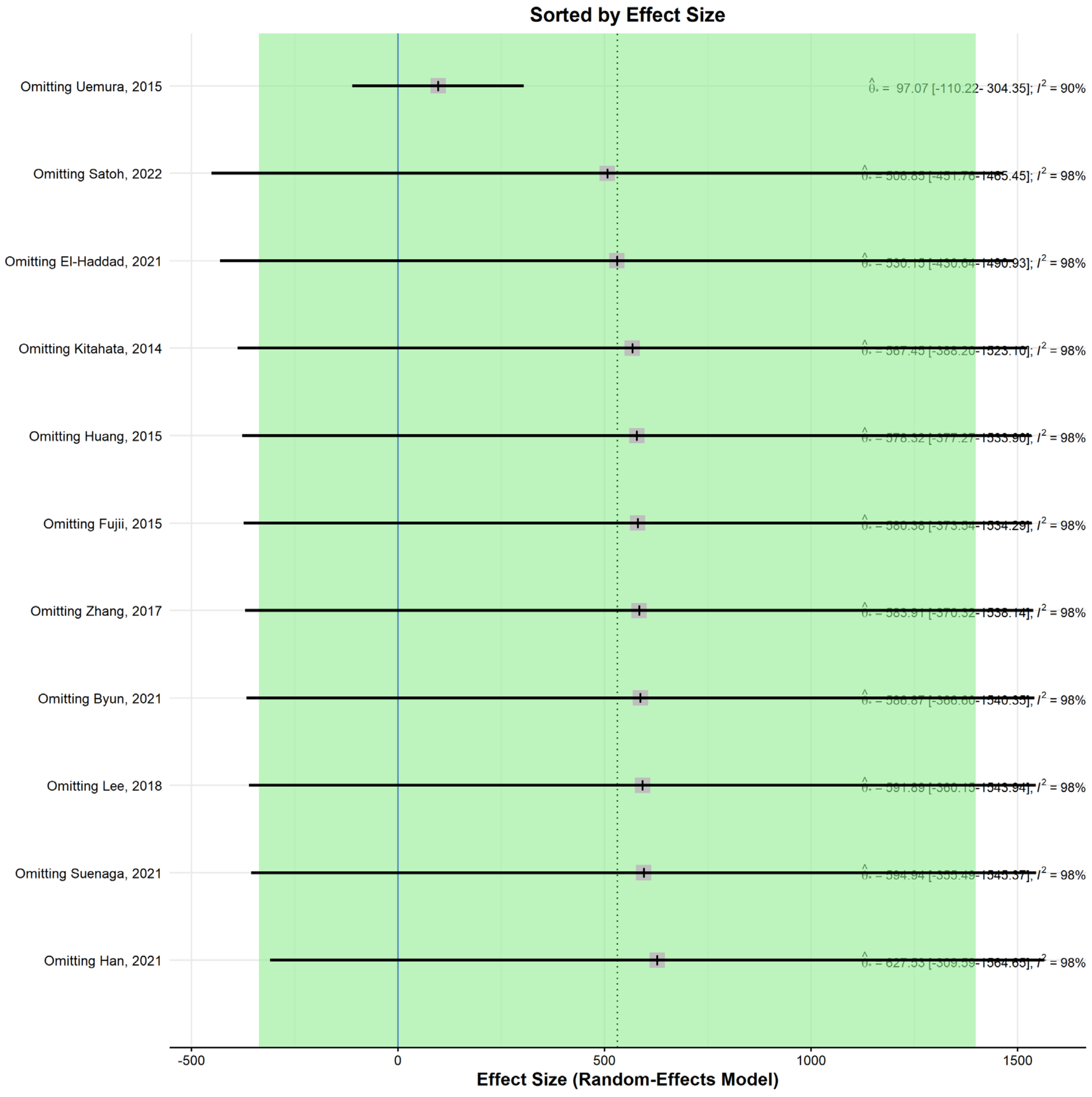

Fig. Leave-one-out sensitivity analysis plot for selected studies for Blood loss (mL)

The heterogeneity was assessed, and we found an I2 of 98.2% (95% CI 97.7% - 98.7%) and the Q test for heterogeneity gave p=< 0.001.

The MD value (the MD of Blood loss (mL) in the ERBD group compared to the ENBD/PTBD group) obtained with the meta-analysis was of 531.04 (95% CI - 336.48 - 1398.57), p=0.23 using the model with random effects.

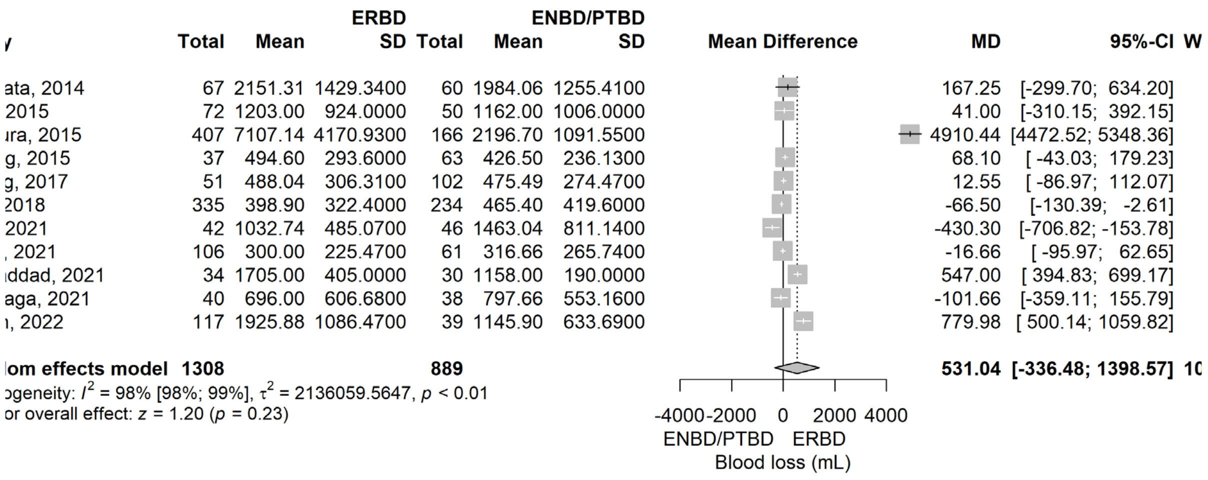

Fig. Forest plot for Blood loss (mL), comparing ERBD with ENBD/PTBD

Subgroup analyses

Algorithm did not converge for subgroup analisis of

Algorithm did not converge for subgroup analisis of

Meta-analysis for Hospital stay (days), comparing ERBD with ENBD/PTBD

	datele.Year	datele.Study.name	MeanTreatment	SDTreatment	numberCasesTreatment	MeanControl	SDControl	numberCasesControl
2	2014	Kitahata, 2014	67.97	42.35	67	41.79	21.60	60
5	2015	Huang, 2015	28.80	25.30	37	22.64	10.52	63
6	2017	Zhang, 2017	29.84	10.03	51	31.85	10.35	102
7	2018	Lee, 2018	25.70	84.30	335	20.60	13.00	234
10	2021	Han, 2021	36.79	17.29	42	39.41	22.87	46
12	2021	El-Haddad, 2021	28.30	13.40	34	19.90	6.40	30
14	2022	Satoh, 2022	59.73	26.77	117	34.64	9.81	39
15	2022	Subasi, 2022	19.90	9.10	42	23.30	12.20	33

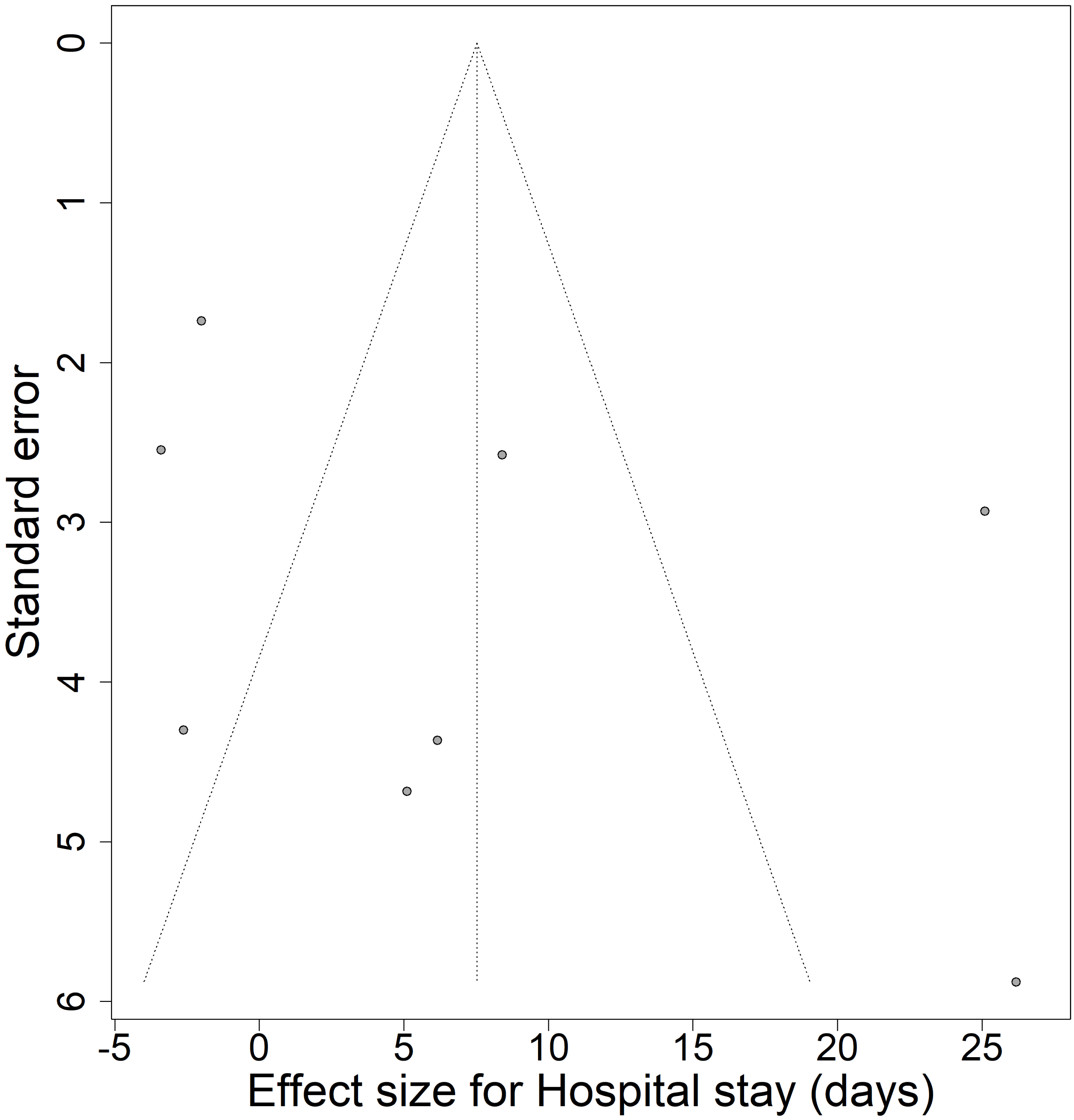

Fig. Funnel plot for Hospital stay (days), comparing ERBD with ENBD/PTBD

The funnel plot for Hospital stay (days), comparing ERBD with ENBD/PTBD is shown in figure _.

The publication bias test gave a p=0.281.

Influence studies: Omitting Kitahata, 2014; Omitting Huang, 2015; Omitting Zhang, 2017; Omitting Lee, 2018; Omitting Han, 2021; Omitting El-Haddad, 2021; Omitting Satoh, 2022; Omitting Subasi, 2022 - no; no; no; no; no; no; yes; no

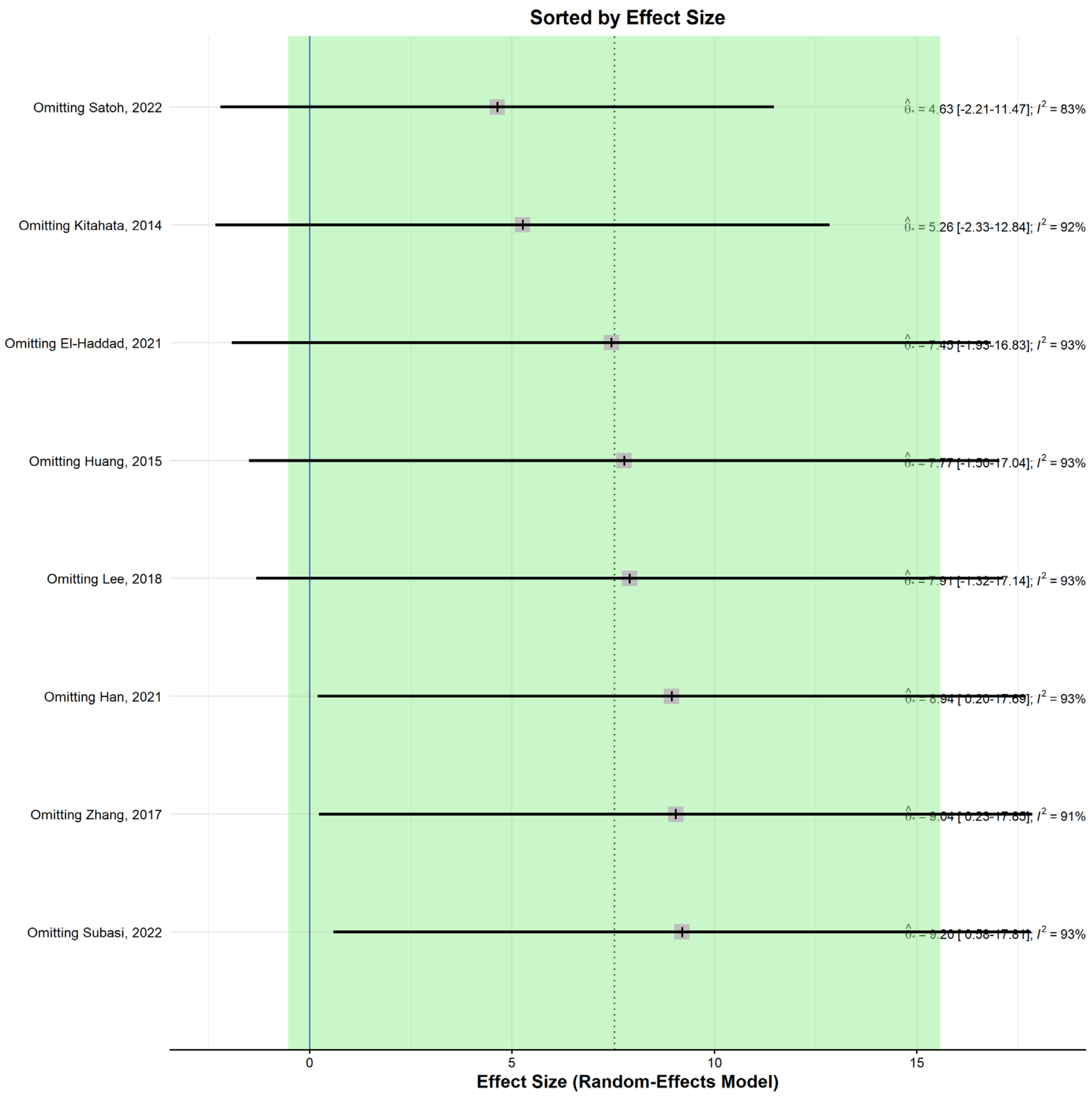

Fig. Leave-one-out sensitivity analysis plot for selected studies for Hospital stay (days)

The heterogeneity was assessed, and we found an I2 of 92.4% (95% CI 87.3% - 95.4%) and the Q test for heterogeneity gave p=< 0.001.

The MD value (the MD of Hospital stay (days) in the ERBD group compared to the ENBD/PTBD group) obtained with the meta-analysis was of 7.53 (95% CI -0.52 - 15.58), p=0.067 using the model with random effects.

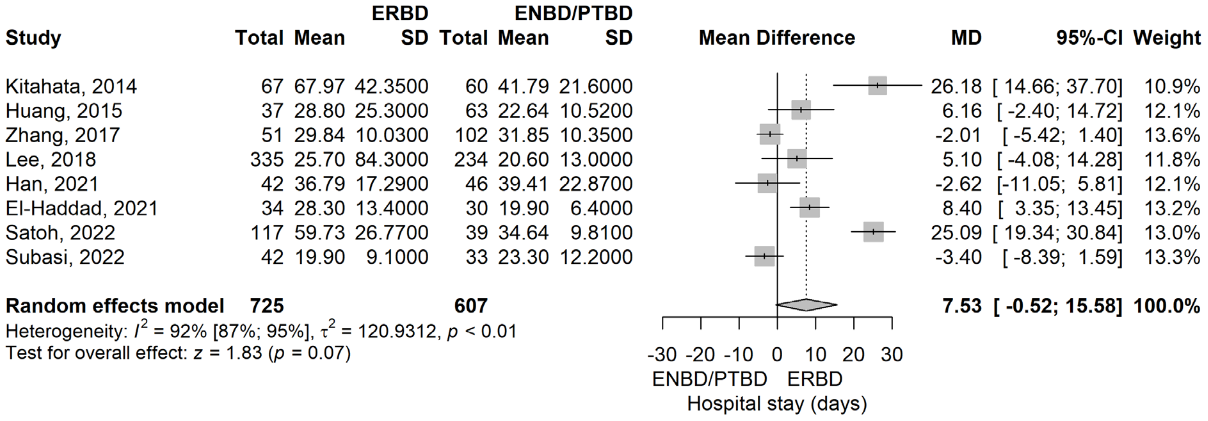

Fig. Forest plot for Hospital stay (days), comparing ERBD with ENBD/PTBD

Sinteza metaanalizelor

Characteristic, effect size type	N studies	e/nt intervention	e/nt control	Effect size (95% CI)	p-value	I2 (95% CI)	p-value	Egger test	Studies	Leave one out
MBD diameter (mm), MD	3	240	191	0.05 (-0.82 - 0.92)	0.915	57.3 (0 - 87.8)	0.096	0.458	Fujii, 2015; Zhang, 2017; Satoh, 2022	- Fujii, 2015: -0.33 (-1.19-0.54), p=0.458, I2=26%; - Zhang, 2017: -0.04 (-1.17-1.1), p=0.951, I2=75%; - Satoh, 2022: 0.58 (-0.24-1.4), p=0.169, I2=0%
Duration of drainage (days), MD	8	452	425	12.31 (-2.76 - 27.39)	0.109	90.3 (83.2 - 94.4)	< 0.001	0.18	Park, 2011; Kitahata, 2014; Fujii, 2015; Huang, 2015;	- Park, 2011: 13.23 (-4.52-30.97), p=0.144, I2=90%; -

	Zhang, 2017; El-Haddad, 2021; Suenaga, 2021; Satoh, 2022	Kitahata, : 13.67 (-3.93-31.27), p=0.128, I2=92%; - Fujii, 201: 15.21 (-1.11-31.52), p=0.068, I2=91%; - Huang, 201: 11.37 (-5.72-28.45), p=0.192, I2=91%; - Zhang, 201: 14.01 (-3.47-31.49), p=0.116, I2=92%; - El-Haddad, : 14.6 (-2.52-31.71), p=0.095, I2=90%; - Suenaga, 2: 12.24 (-5.19-29.67), p=0.169, I2=91%; - Satoh, 202: 3.58 (-0.53-7.68), p=0.088, I2=77%

Operative time (min), MD	12	1350	935	-13.14 (-26.83 - 0.54)	0.06	49.7 (2.3 - 74.1)	0.026	0.531	Park, 2011; Kitahata , 2014; Fujii, 2015; Uemura , 2015; Huang, 2015; Zhang, 2017; Lee, 2018; Han, 2021; Byun, 2021; Suenaga , 2021; Satoh, 2022; Subasi, 2022	- Park, 2011: - 8.81 (- 19.53- 1.91), p=0.107, I2=19%; - Kitahata , : -9.95 (-23.19- 3.29), p=0.141, I2=43%; - Fujii, 201: - 14.04 (- 28.98- 0.9), p=0.066, I2=54%; - Uemura, 20: - 12.76 (- 28.39- 2.87), p=0.11, I2=54%; - Huang, 201: - 14.39 (- 29.25- 0.47), p=0.058, I2=53%; - Zhang, 201: - 15.85 (- 29.57-- 2.13), p=0.024, I2=47%; - Lee, 2018: - 15.06 (- 30.67- 0.56), p=0.059, I2=50%;

										- Han, 2021: -11.19 (-25.52-3.14), p=0.126, I2=50%; - Byun, 2021: -13.77 (-29.52-1.97), p=0.086, I2=54%; - Suenaga, 2: -12.92 (-27.39-1.54), p=0.08, I2=54%; - Satoh, 202: -15.42 (-29.39--1.46), p=0.03, I2=49%; - Subasi, 20: -14.01 (-28.41-0.4), p=0.057, I2=54%
Blood loss (mL), MD	11	1308	889	531.04 (-336.48 - 1398.57)	0.23	98.2 (97.7 - 98.7)	< 0.001	0.115	Kitahata, 2014; Fujii, 2015; Uemura, 2015; Huang, 2015; Zhang, 2017; Lee, 2018; Han,	- Kitahata, 2014: 567.45 (-388.2-1523.1), p=0.245, I2=98%; - Fujii, 2015: 580.38 (-373.54-

	2021; Byun, 2021; El-Haddad, 2021; Suenaga, 2021; Satoh, 2022	1534.29), p=0.233, I2=98%;<br>- Uemura, 2015: 97.07 (-110.22-304.35), p=0.359, I2=90%;<br>- Huang, 2015: 578.32 (-377.27-1533.9), p=0.236, I2=98%;<br>- Zhang, 2017: 583.91 (-370.32-1538.14), p=0.23, I2=98%;<br>- Lee, 2018: 591.89 (-360.15-1543.94), p=0.223, I2=98%;<br>- Han, 2021: 627.53 (-309.59-1564.65), p=0.189, I2=98%;<br>- Byun, 2021: 586.87 (-366.6-

										1540.35), p=0.228, I2=98%; - El-Haddad, 202: 530.15 (-430.64-1490.93), p=0.279, I2=98%; - Suenaga, 2021: 594.94 (-355.49-1545.37), p=0.22, I2=98%; - Satoh, 2022: 506.85 (-451.76-1465.45), p=0.3, I2=98%
Hospital stay (days), MD	8	725	607	7.53 (-0.52 - 15.58)	0.067	92.4 (87.3 - 95.4)	< 0.001	0.281	Kitahata, 2014; Huang, 2015; Zhang, 2017; Lee, 2018; Han, 2021; El-Haddad, 2021; Satoh, 2022; Subasi, 2022	- Kitahata, 2014: 5.26 (-2.33-12.84), p=0.174, I2=92%; - Huang, 2015: 7.77 (-1.5-17.04), p=0.1, I2=93%; - Zhang, 2017: 9.04

	(0.23-17.85), p=0.044, I2=91%;<br>- Lee, 2018: 7.91 (-1.32-17.14), p=0.093, I2=93%;<br>- Han, 2021: 8.94 (0.2-17.69), p=0.045, I2=93%;<br>- El-Haddad, 202: 7.45 (-1.93-16.83), p=0.119, I2=93%;<br>- Satoh, 2022: 4.63 (-2.21-11.47), p=0.184, I2=83%;<br>- Subasi, 2022: 9.2 (0.58-17.81), p=0.036, I2=93%

Selectie: PS - SEMS

Meta-analysis for MBD diameter (mm), comparing PS with SEMS

No studies to combine for this characteristic

Meta-analysis for Duration of drainage (days), comparing PS with SEMS

	datele.Year	datele.Study.name	MeanTreatment	SDTreatment	numberCasesTreatment	MeanControl	SDControl	numberCasesControl
4	2016	Song, 2016	14.20	8.30	43	12.30	6.90	43
5	2020	Latenstein, 2020	38.50	20.88	329	29.66	14.95	246
6	2020	Cho, 2020	11.33	5.49	26	11.33	4.69	27
8	2021	Roberts, 2021	47.20	7.20	108	50.10	9.60	49
				2				

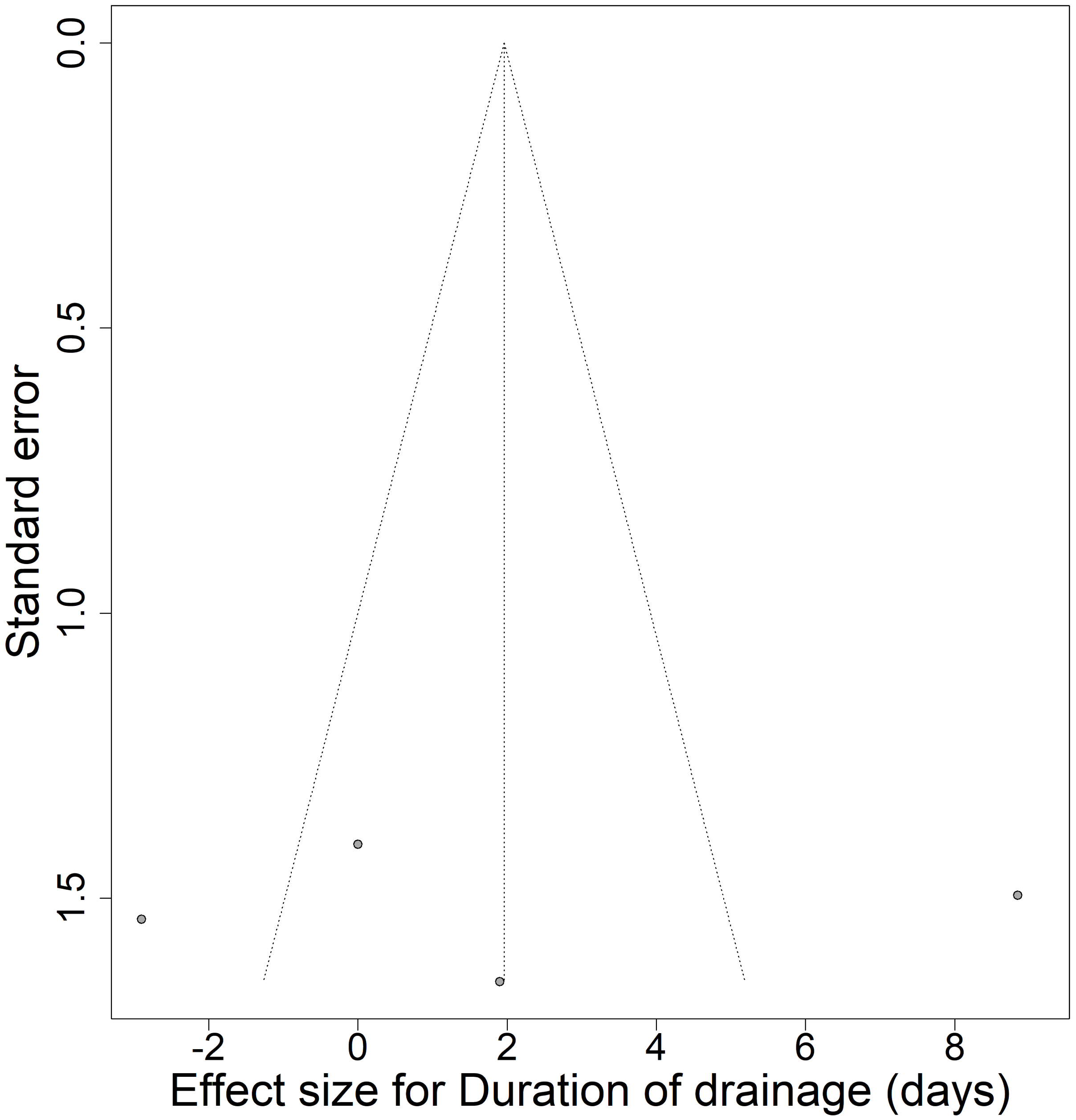

Fig. Funnel plot for Duration of drainage (days), comparing PS with SEMS

The funnel plot for Duration of drainage (days), comparing PS with SEMS is shown in figure _.

The publication bias test gave a p=0.999.

Influence studies: Omitting Song, 2016; Omitting Latenstein, 2020; Omitting Cho, 2020; Omitting Roberts, 2021 - no; yes; no; no

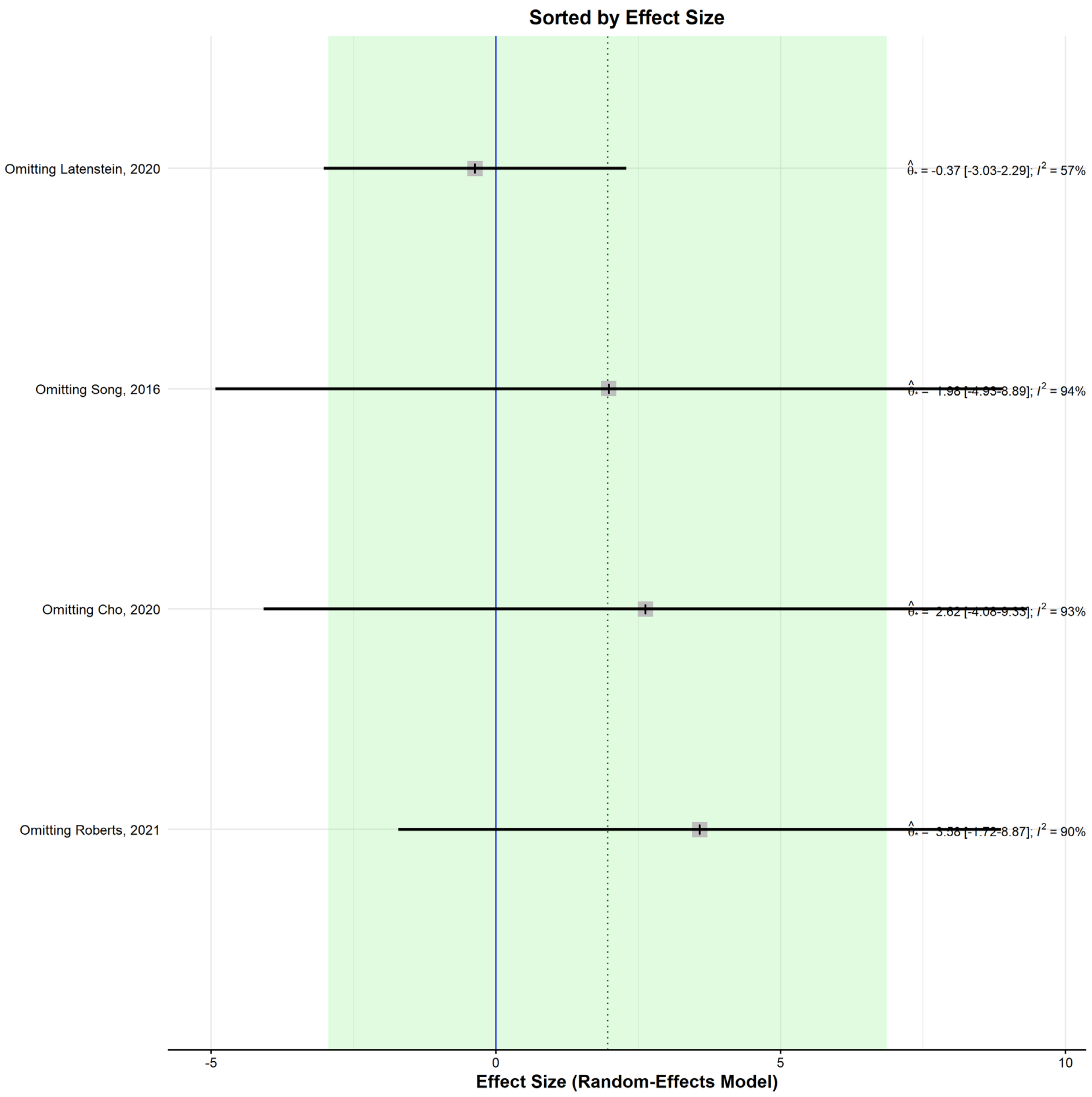

Fig. Leave-one-out sensitivity analysis plot for selected studies for Duration of drainage (days)

The heterogeneity was assessed, and we found an I2 of 90.9% (95% CI 79.9% - 95.9%) and the Q test for heterogeneity gave p=< 0.001.

The MD value (the MD of Duration of drainage (days) in the PS group compared to the SEMS group) obtained with the meta-analysis was of 1.96 (95% CI -2.94 - 6.87), p=0.433 using the model with random effects.

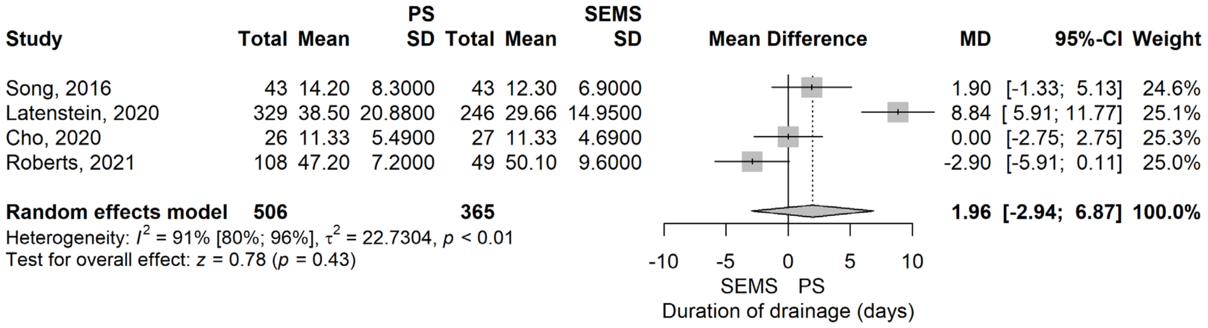

Fig. Forest plot for Duration of drainage (days), comparing PS with SEMS

Meta-analysis for Operative time (min), comparing PS with SEMS

	datele.Year	datele.Study.name	MeanTreatment	SDTreatment	numberCasesTreatment	MeanControl	SDControl	numberCasesControl
1	2013	Cavell, 2013	301.32	88.40	149	301.81	71.14	71
7	2020	Kuwatani, 2020	482.66	131.66	12	604.00	162.48	17

2

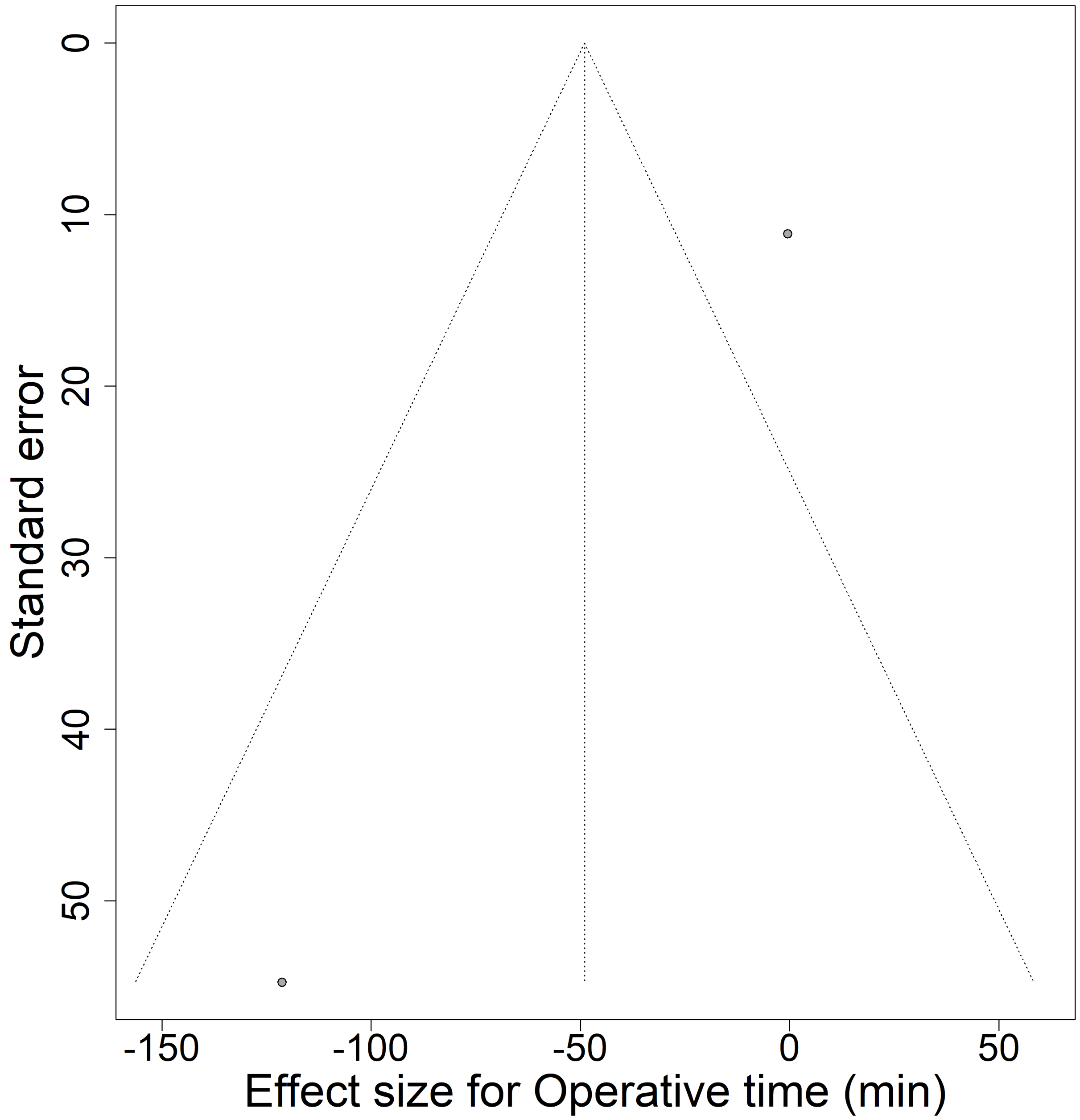

Fig. Funnel plot for Operative time (min), comparing PS with SEMS

The funnel plot for Operative time (min), comparing PS with SEMS is shown in figure _.

The publication bias test cannot be computed since there are not at least three studies.

Influence studies: Omitting Cavell, 2013; Omitting Kuwatani, 2020 - yes; yes

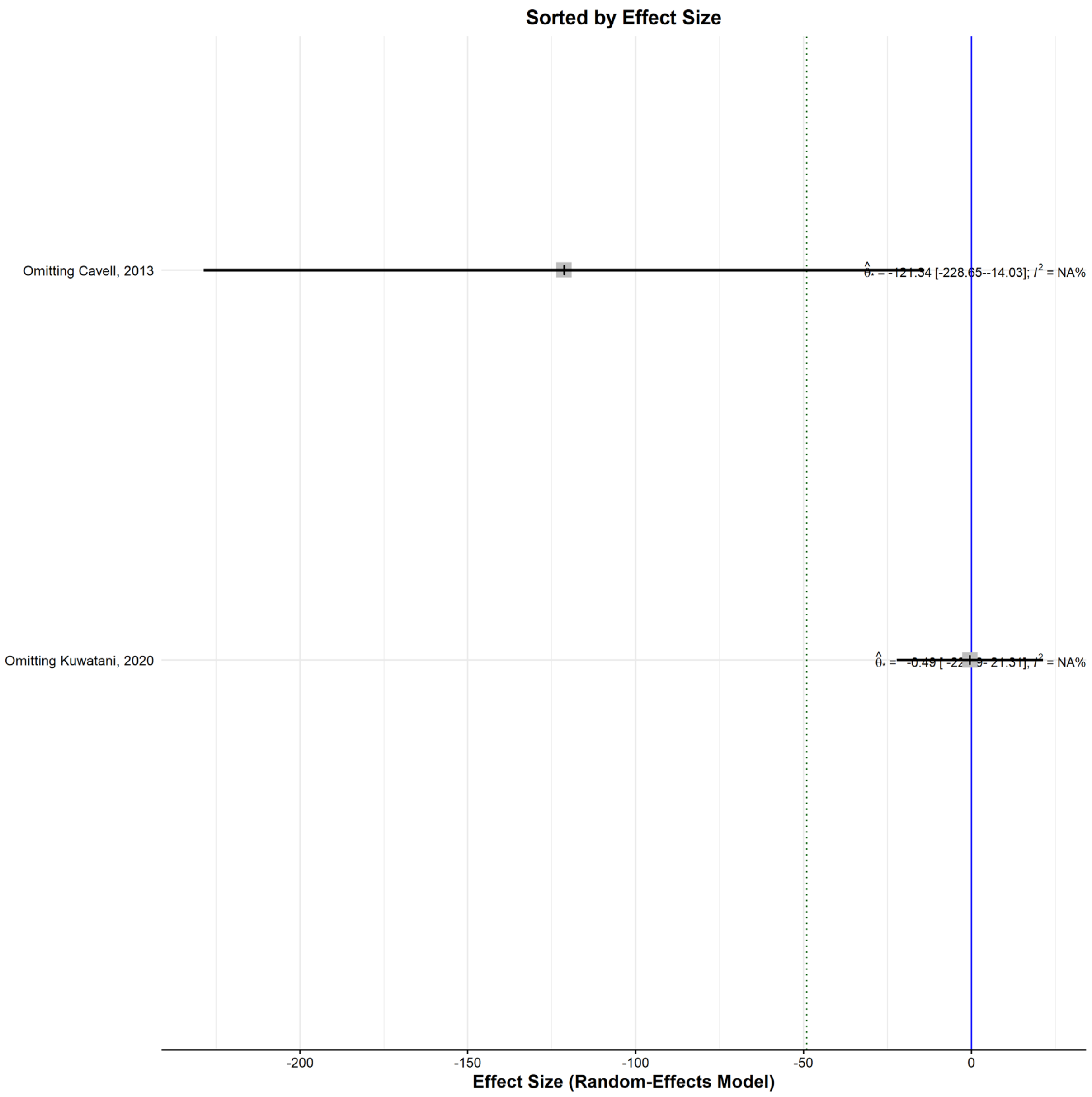

Fig. Leave-one-out sensitivity analysis plot for selected studies for Operative time (min)

The heterogeneity was assessed, and we found an I2 of 78.6% (95% CI 7.2% - 95.1%) and the Q test for heterogeneity gave p=0.031.

The MD value (the MD of Operative time (min) in the PS group compared to the SEMS group) obtained with the meta-analysis was of -49.03 (95% CI -165.14 - 67.09), p=0.408 using the model with random effects.

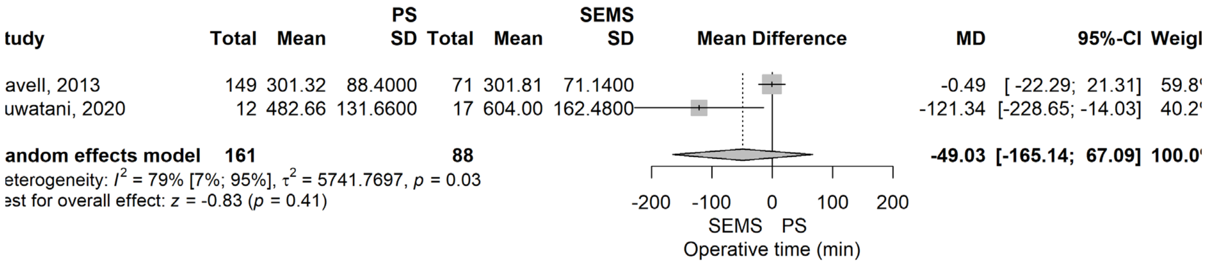

Fig. Forest plot for Operative time (min), comparing PS with SEMS

Meta-analysis for Blood loss (mL), comparing PS with SEMS

	datele.Year	datele.Study.name	MeanTreatment	SDTreatment	numberCasesTreatment	MeanControl	SDControl	numberCasesControl
1	2013	Cavell, 2013	1519.30	882.22	149	905.63	469.10	71
7	2020	Kuwatani, 2020	625.66	524.96	12	766.33	582.03	17
2								

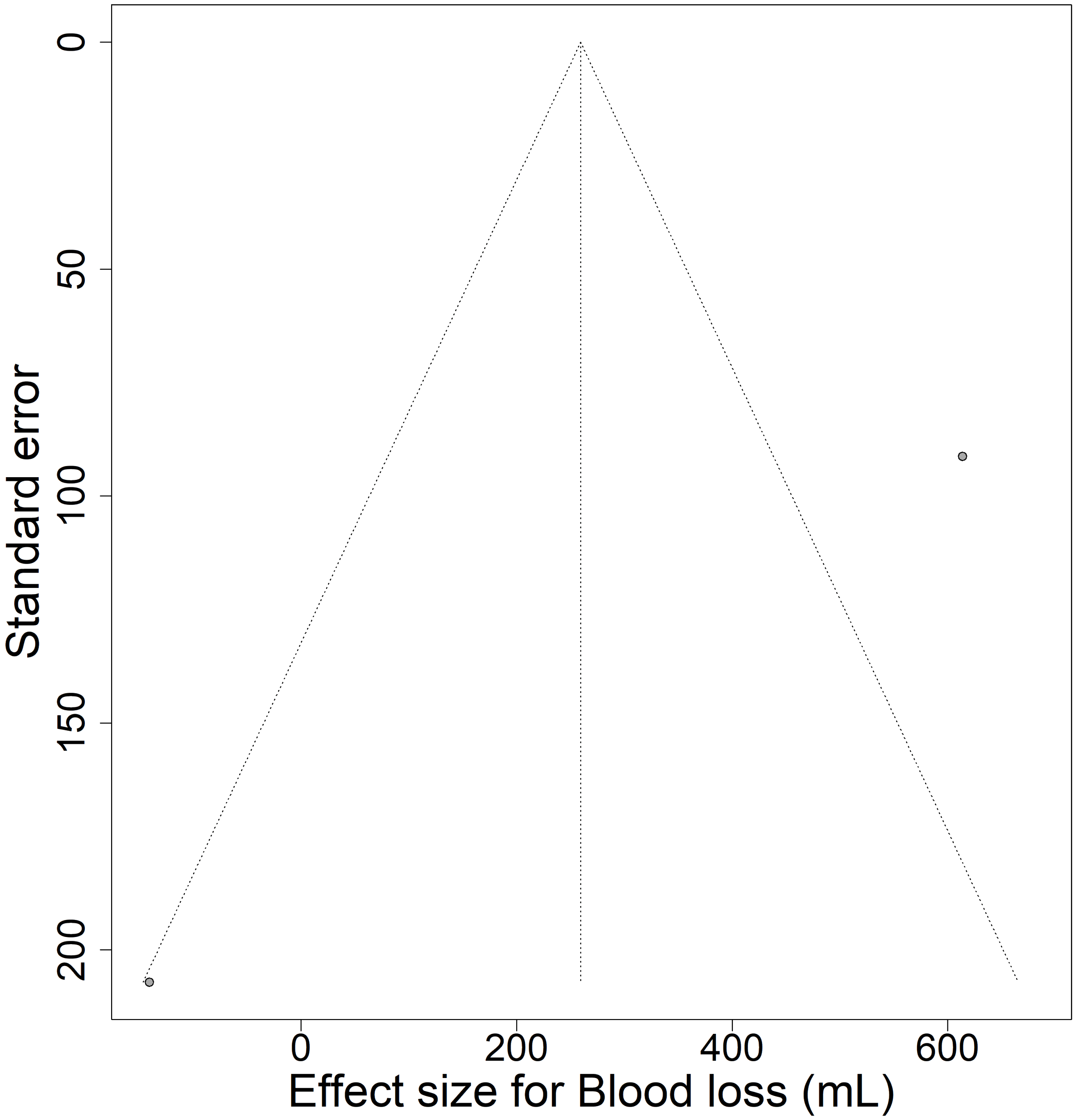

Fig. Funnel plot for Blood loss (mL), comparing PS with SEMS

The funnel plot for Blood loss (mL), comparing PS with SEMS is shown in figure _.

The publication bias test cannot be computed since there are not at least three studies.

Influence studies: Omitting Cavell, 2013; Omitting Kuwatani, 2020 - yes; yes

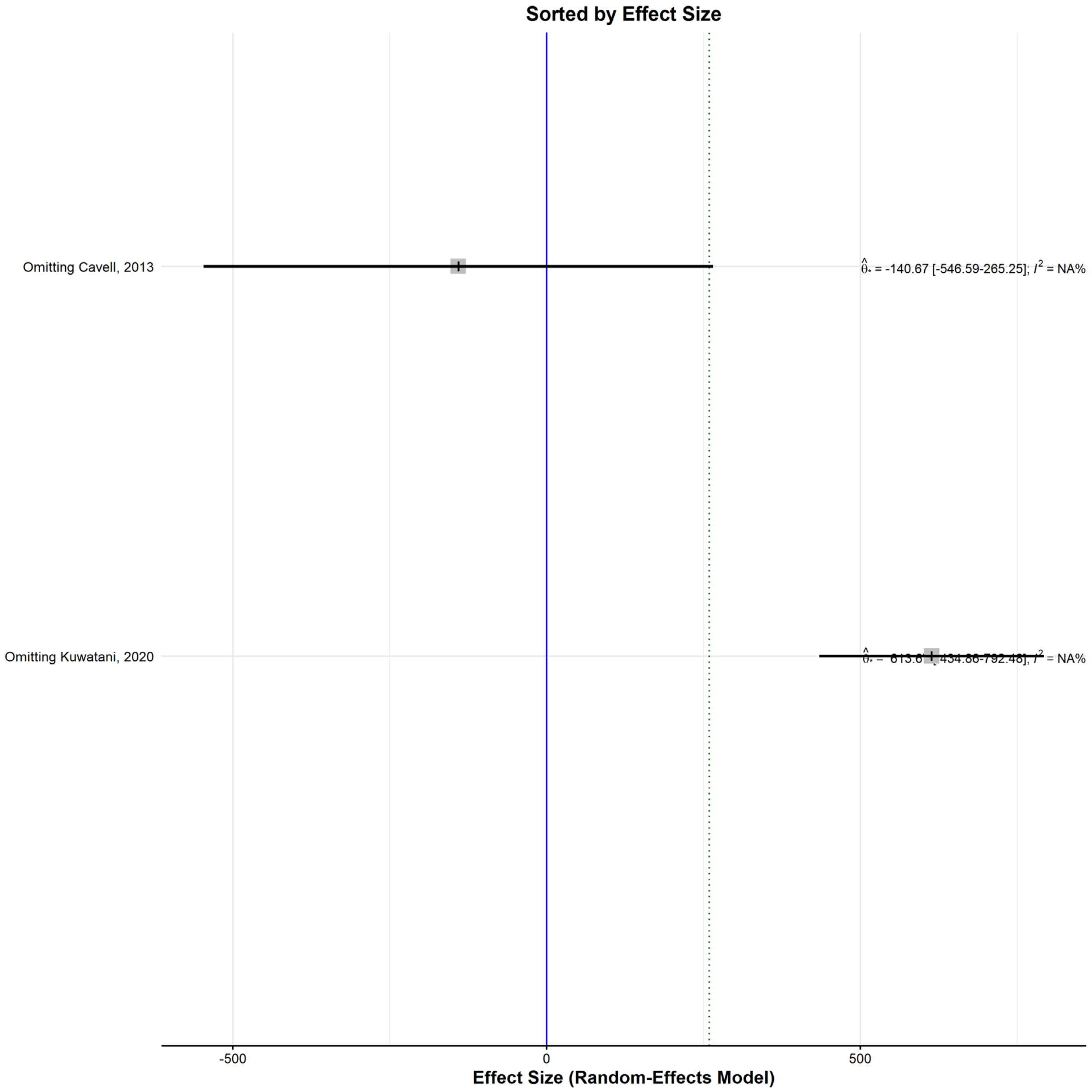

Fig. Leave-one-out sensitivity analysis plot for selected studies for Blood loss (mL)

The heterogeneity was assessed, and we found an I2 of 91% (95% CI 67.9% - 97.5%) and the Q test for heterogeneity gave p=< 0.001.

The MD value (the MD of Blood loss (mL) in the PS group compared to the SEMS group) obtained with the meta-analysis was of 259.41 (95% CI -478.46 - 997.29), p=0.491 using the model with random effects.

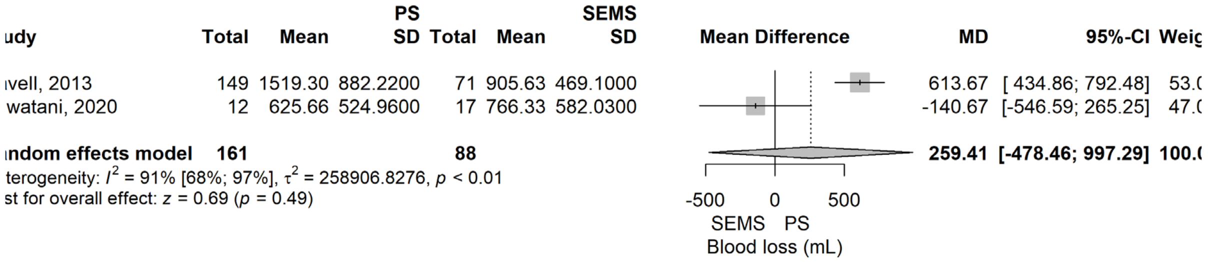

Fig. Forest plot for Blood loss (mL), comparing PS with SEMS

Meta-analysis for Hospital stay (days), comparing PS with SEMS

	datele.Year	datele.Study.name	MeanTreatment	SDTreatment	numberCasesTreatment	MeanControl	SDControl	numberCasesControl
1	2013	Cavell, 2013	20.83	10.51	149	21.18	11.33	71
3	2016	Tol, 2016	16.33	8.27	102	12.66	7.63	49
5	2020	Latenstein, 2020	17.40	18.14	329	14.00	9.41	246
6	2020	Cho, 2020	15.33	8.62	26	11.83	5.08	27
9	2022	Bademci, 2022	23.00	14.70	17	27.00	18.30	31
				2				

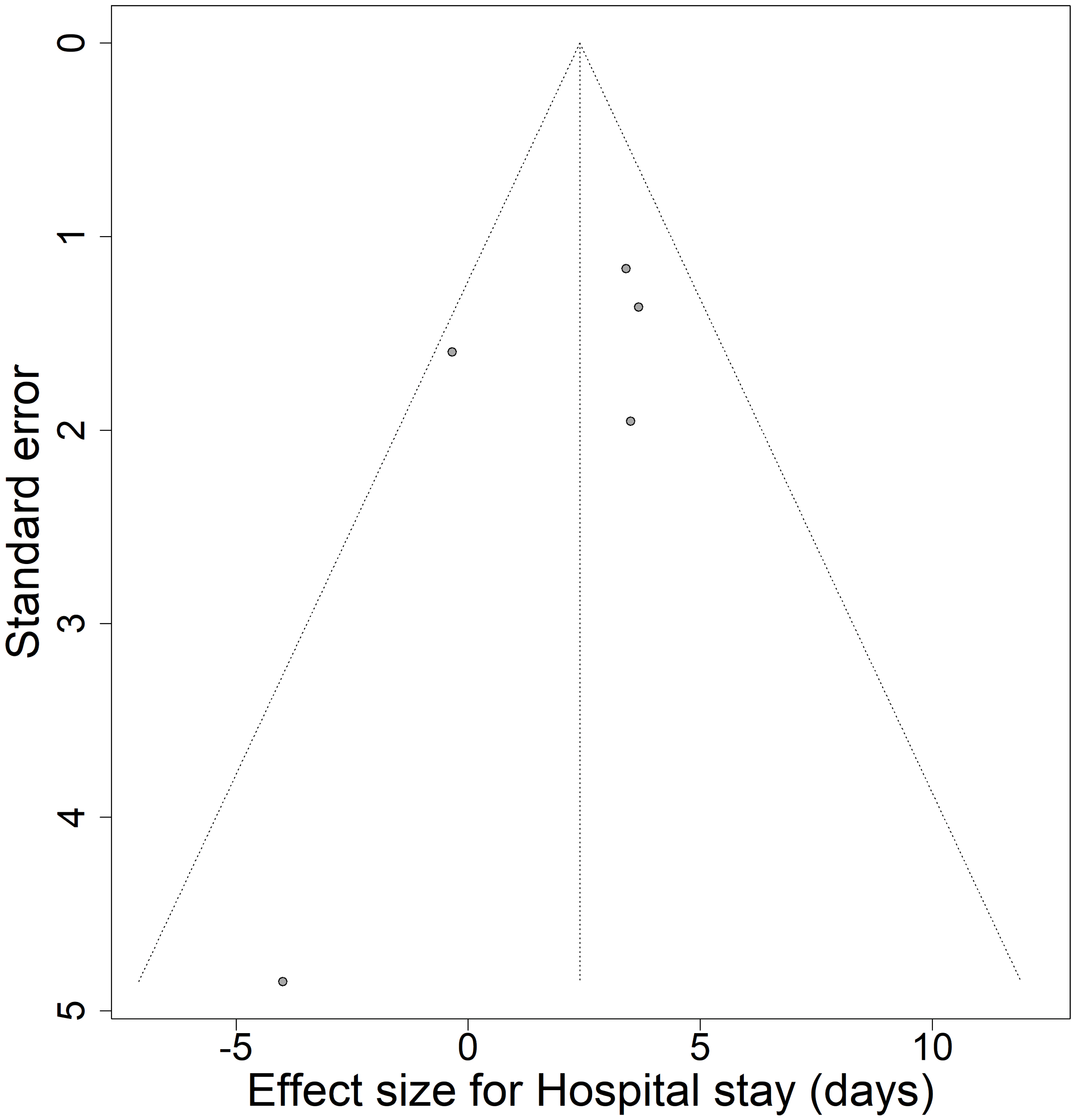

Fig. Funnel plot for Hospital stay (days), comparing PS with SEMS

The funnel plot for Hospital stay (days), comparing PS with SEMS is shown in figure _.

The publication bias test gave a p=0.283.

Influence studies: Omitting Cavell, 2013; Omitting Tol, 2016; Omitting Latenstein, 2020; Omitting Cho, 2020; Omitting Bademci, 2022 - yes; no; no; no; no

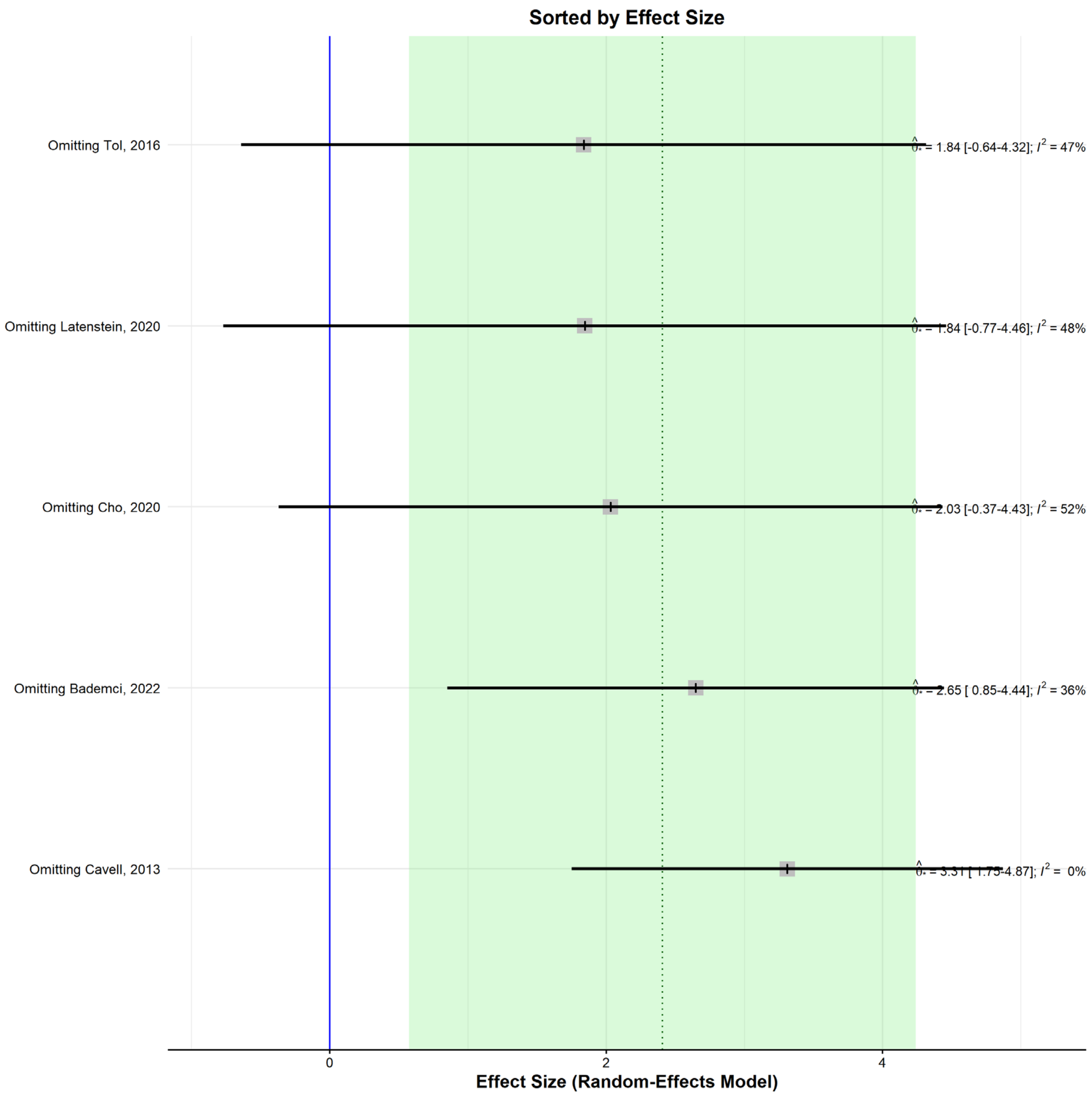

Fig. Leave-one-out sensitivity analysis plot for selected studies for Hospital stay (days)

The heterogeneity was assessed, and we found an I2 of 39.1% (95% CI 0% - 77.4%) and the Q test for heterogeneity gave p=0.161.

The MD value (the MD of Hospital stay (days) in the PS group compared to the SEMS group) obtained with the meta-analysis was of 2.41 (95% CI 0.57 - 4.24), p=0.01 using the model with random effects.

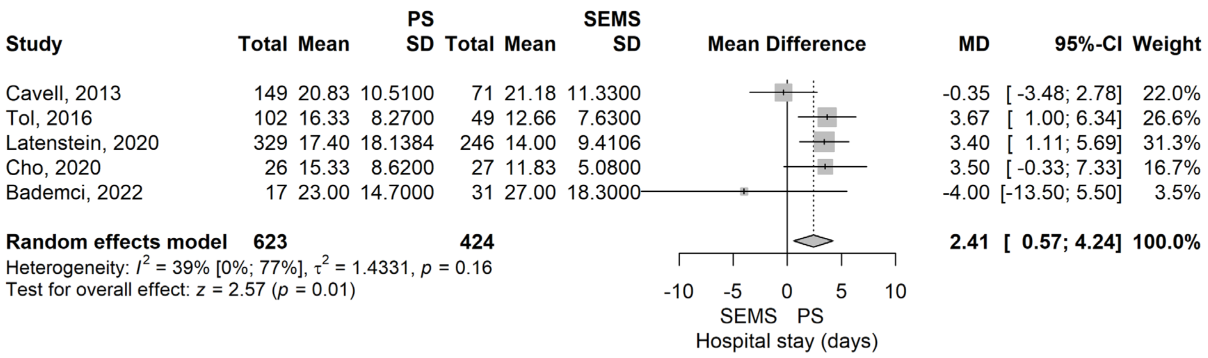

Fig. Forest plot for Hospital stay (days), comparing PS with SEMS

Sinteza metaanalizelor

Characteristic, effect size type	N studies	e/nt intervention	e/nt control	Effect size (95% CI)	p-value	I2 (95% CI)	p-value	Egger test	Studies	Leave one out
MBD diameter (mm), MD	0	-	-	-	-	-	-	-	-	-
Duration of drainage (days), MD	4	506	365	1.96 (-2.94 - 6.87)	0.433	90.9 (79.9 - 95.9)	< 0.001	0.999	Song, 2016; Latenstein, 2020; Cho, 2020; Roberts, 2021	- Song, 2016: 1.98 (-4.93-8.89), p=0.574, I2=94%; - Latenstein: -0.37 (-3.03-2.29), p=0.785, I2=57%; - Cho, 2020: 2.62 (-4.08-9.33), p=0.443, I2=93%; - Roberts, 2: 3.58 (-1.72-8.87), p=0.185, I2=90%

Operative time (min), MD	2	161	88	-49.03 (-165.14 - 67.09)	0.408	78.6 (7.2 - 95.1)	0.031	NC	Cavell, 2013; Kuwatani, 2020	- Cavell, 2013: -121.34 (-228.65--14.03), p=0.027, I2=NA%; - Kuwatani, 20: -0.49 (-22.29-21.31), p=0.965, I2=NA%
Blood loss (mL), MD	2	161	88	259.41 (-478.46 - 997.29)	0.491	91 (67.9 - 97.5)	< 0.001	NC	Cavell, 2013; Kuwatani, 2020	- Cavell, 2013: -140.67 (-546.59-265.25), p=0.497, I2=NA%; - Kuwatani, 20: 613.67 (434.86-792.48), p=< 0.001, I2=NA%
Hospital stay (days), MD	5	623	424	2.41 (0.57 - 4.24)	0.01	39.1 (0 - 77.4)	0.161	0.283	Cavell, 2013; Tol, 2016; Latenstein, 2020; Cho, 2020; Bademci, 2022	- Cavell, 2013: 3.31 (1.75-4.87), p=< 0.001, I2=0%; - Tol, 2016: 1.84 (-0.64-4.32), p=0.146, I2=47%; - Latenstein, : 1.84

	(-0.77-4.46), p=0.167, I2=48%;<br>- Cho, 2020: 2.03 (-0.37-4.43), p=0.097, I2=52%;<br>- Bademci, 202: 2.65 (0.85-4.44), p=0.004, I2=36%
